# Scalable Total Synthesis of Bastimolide A Enabled by Asymmetric Allylborations Catalyzed by Chiral Brønsted Acids

**DOI:** 10.1021/jacsau.5c00630

**Published:** 2025-07-08

**Authors:** Shigenobu Umemiya, Naoya Shinagawa, Aisuke Fujimoto, Masahiro Terada

**Affiliations:** † Research and Analytical Center for Giant Molecules, Graduate School of Science, Tohoku University, 6-3 Aramaki Aza Aoba, Aoba-ku, Sendai 980-8578, Japan; ‡ Department of Chemistry, Graduate School of Science, 208508 Tohoku University, Sendai 980-8578, Japan

**Keywords:** scalable synthesis, chiral Brønsted acid, macrolide, organocatalysis, asymmetric allylation

## Abstract

The efficient and scalable synthesis of natural products, such as polyketides, has paved the way for the discovery of pharmaceuticals, saving millions of lives and improving people’s health. We report the catalytic total synthesis of bastimolide A, a potent antimalarial, in 21 steps in a total yield of 15.4%, employing various catalytic reactions without using stoichiometric asymmetric reactions or excess amounts of heavy metal reagents. Our efficient and scalable synthesis can catalytically forge seven of 10 stereogenic centers in bastimolide A through chiral Brønsted acid-catalyzed enantio- and diastereoselective allylborations and Corey–Bakshi–Shibata reduction as key steps.

## Graphical abstract

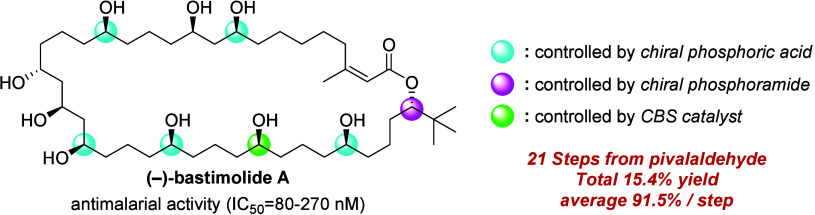

The efficient synthesis of natural products and pharmaceuticals is critical in organic synthesis.^1–6^ Tremendous effort has been devoted to the efficient and scalable syntheses of natural products for drug discovery over the past decades.^7,8^ Biological and structure–activity relationship (SAR) studies and clinical trials have been performed, thanks to the abundance of compounds produced through sophisticated synthetic routes. These efforts have paved the way for discovering a wide range of pharmaceuticals, saving millions of lives and improving human health. However, despite such great enterprise, significant challenges, such as minimizing synthetic costs and chemical wastes, have remained in modern chemical synthesis because global inflation and environmental problems have worsened in recent years. Efficient synthetic processes that utilize catalytic reactions are required to reduce synthetic costs and preserve the environment.^9,10^ Thus, developing new methodologies and catalysts to pursue ideal synthesis is essential in organic synthesis and organic synthetic chemistry.

Asymmetric catalysis has continued to fascinate researchers over the past few decades. It shows excellent synergy with the efficient synthesis of enantioenriched natural products and their derivatives because it can produce large quantities of chiral building blocks from a few chiral resources, providing access to every possible stereoisomer. Under these circumstances, we have focused our research on chiral Brønsted acid catalysts,^11–15^ specifically chiral phosphoric acids (CPAs)^16–19^ and chiral phosphoramides.^20^ These chiral Brønsted acid catalysts are environmentally benign and sustainable, because they do not contain heavy metals. Additionally, they are stable under various reaction conditions, allowing for the reduction of the catalyst loading and easy recovery. In 2010, the Antilla group reported the first CPA-catalyzed enantioselective allylboration of aldehyde.^21^ We have achieved a short synthesis of fostriecin and a scalable synthesis of leucascandrolide A macrolactone through the development of an enantioselective allylboration of acetylenic aldehydes in a CPA/transition metal cooperative system (Figure 1, eq 1) and a CPA-catalyzed stereoselective allylboration of a functionalized aldehyde including a stereogenic center (Figure 1, eq 2).^22,23^ More recently, we have established an enantioselective allylboration of sterically hindered aldehydes based on an “interaction strategy”,^24–28^ which is difficult to achieve with conventional CPA catalysts (Figure 1, eq 3).^24^ The high enantioselectivity of the chiral phosphoramide-catalyzed allylboration was accomplished through the efficiently stabilized transition state of the major reaction pathway, utilizing not the high acidity of the catalyst but the unique multiple interactions between the phosphoramide and substrates.

**Figure 1:**
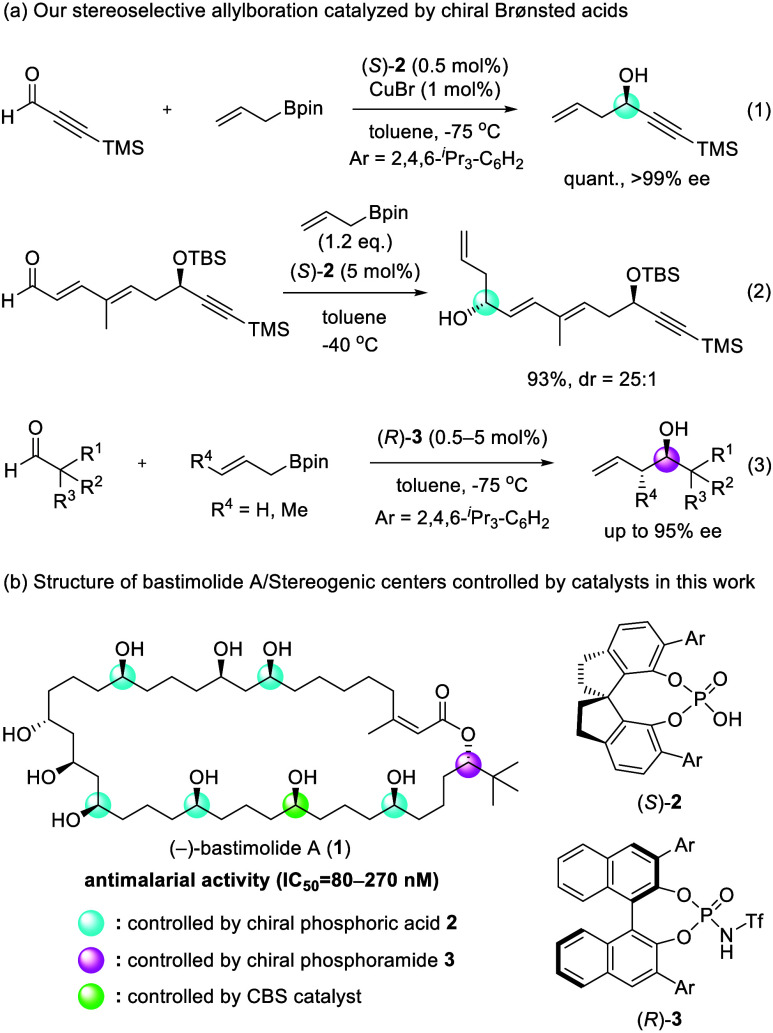
Introduction of chiral Brønsted acids and our concept of enantioselective total synthesis of bastimolide A (**1**).

Bastimolide A (**1**) is a 40-membered-ring macrolide isolated by Gerwick and co-workers in 2015. It has potent antimalarial activity (IC_50_ = 80–270 nM)^29–31^ and is characterized by an α,β-unsaturated macrolactone with isomerizable *Z*-geometry, a 1,3,5-triol moiety, and a 1,5-polyol structure. Its complicated structure has presented a synthetic challenge.^32,33^ The promising biological activities and unique structures of related natural products have attracted the interest of synthetic chemists, and synthetic studies^34–36^ and a total synthesis by Smith’s group^37^ have been reported. Smith’s elegant synthesis furnished this complex molecule in only 20 steps from enantioenriched epoxides in 0.65% total yield. Although they established a short-step synthesis of **1** through a convergent approach, they used excess amounts of toxic heavy metals such as mercury reagents, and most of their transformations were not catalytic reactions. Resolving these issues would decrease chemical waste and synthetic costs. In this regard, using chiral Brønsted acid-catalyzed carbon–carbon bond forming reactions with predictable stereoselectivities to forge bastimolide A (**1**) would be an ideal strategy, in terms of efficiency and access to its stereoisomers. Herein, we report an efficient and enantioselective total synthesis of bastimolide A (**1**) over the longest linear sequence (LLS) of 21 steps in a total yield of 15.4% without using stoichiometric asymmetric reactions. The synthesis was enabled by a novel enantioselective allylboration using multiple interactions of a phosphoramide catalyst^24^ and various catalytic carbon–carbon bond forming reactions, including our refined stereoselective allylation reactions using a CPA and copper cocatalyst system.^22^ Our synthetic route constructed all 10 stereogenic centers in bastimolide A (**1**) in a scalable manner: seven stereogenic centers were controlled by asymmetric catalysis, whereas the remaining three were forged by asymmetric inductions without any chiral reagents.

Our retrosynthetic analysis is shown in Figure 2. We envisioned that the intramolecular Suzuki–Miyaura coupling reaction under mild conditions would be suitable for constructing the macrolactone ring while minimizing the isomerization of the *Z*-unsaturated lactone moiety. The stereogenic center at C19 would be forged at the late stage of the synthesis through a 1,5-*anti*-aldol reaction utilizing the C23 stereocenter. C4–C19 fragment **4** would be efficiently synthesized through a ring-opening reaction of epoxide **7** with alkyne **6**, while C20–C43 fragment **5** would be prepared by a cross-metathesis reaction of enone **8** with terminal olefin **9** and a CPA-catalyzed diastereoselective allylation reaction. Through careful retrosynthesis, bastimolide A (**1**) would be derived from simple enantioenriched subunits **6**, **7**, **8**, and **9**, all of which would be concisely synthesized by taking advantage of chiral Brøsted acid-catalyzed enantioselective allylation reactions.

**Figure 2:**
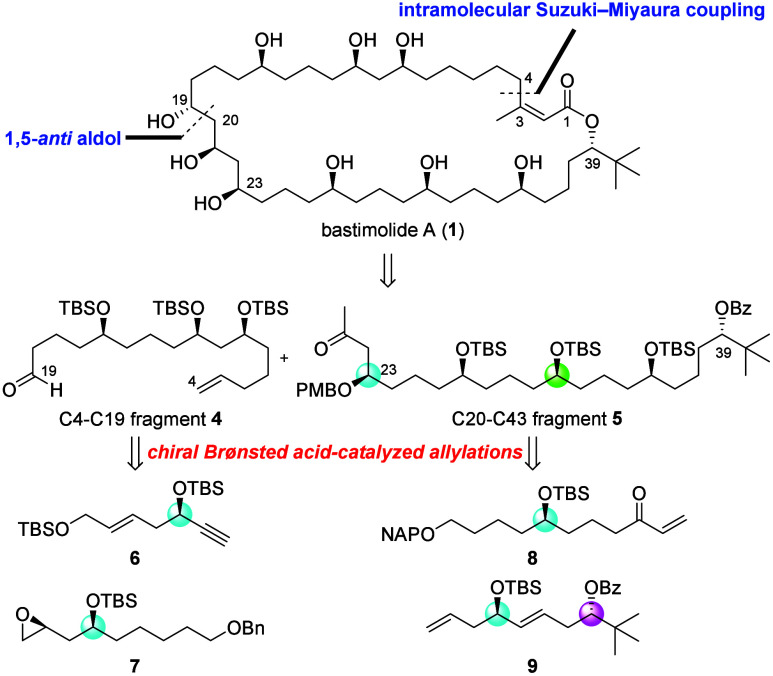
Synthetic plan for bastimolide A.

Our synthesis commenced with the CPA/CuBr-catalyzed enantioselective allylboration of TMS-substituted acetylenic aldehyde **10** (Figure 3a).^22^ We obtained enantioenriched propargylic alcohol **12** in quantitative yield with perfect enantioselectivity using only 0.5 mol % of CPA. Furthermore, CPA (*S*)-**2** used in this reaction could be recovered in 98% yield through a simple workup using *N*-methyl-2-aminoethanol to trap boron compounds. The recovered catalyst promoted the allylboration without losing chemical yield and enantioselectivity.^38^ Subsequent protection of the secondary hydroxyl group by a TBS group afforded terminal olefin **13**. A three-step operation involving olefin metathesis with methyl acrylate, ester reduction by DIBAL-H, and protection of the primary hydroxyl group accompanied by deprotection of the TMS group at the alkyne terminus produced terminal alkyne **6** in a good yield. As shown in Figure 3b, epoxide subunit **7** could be readily prepared from aldehyde **16** in a four-step sequence, including the enantioselective CPA-catalyzed allylboration and the Bartlett–Smith epoxide synthesis.^39,40^ Next, deprotonation of the terminal alkyne by *n*-BuLi, followed by the addition of enantioenriched epoxide **7** in the presence of BF_3_·Et_2_O,^41^ promoted the ring-opening reaction of the epoxide, affording the coupling product in excellent yield. The protection of the generated secondary hydroxyl group and the reduction of the double and triple bonds, accompanied by benzyl group removal under mild hydrogenation conditions, gave the primary alcohol **15**. A terminal olefin was introduced by the Grieco–Nishizawa olefination^42^ of **15**. The selective deprotection of the primary TBS group and the subsequent TEMPO oxidation furnished the C4–C19 fragment, namely, aldehyde **4**, from commercially available 3-(trimethylsilyl)-2-propynal (**10**) in 11 steps and a total yield of 41.2%.

**Figure 3:**
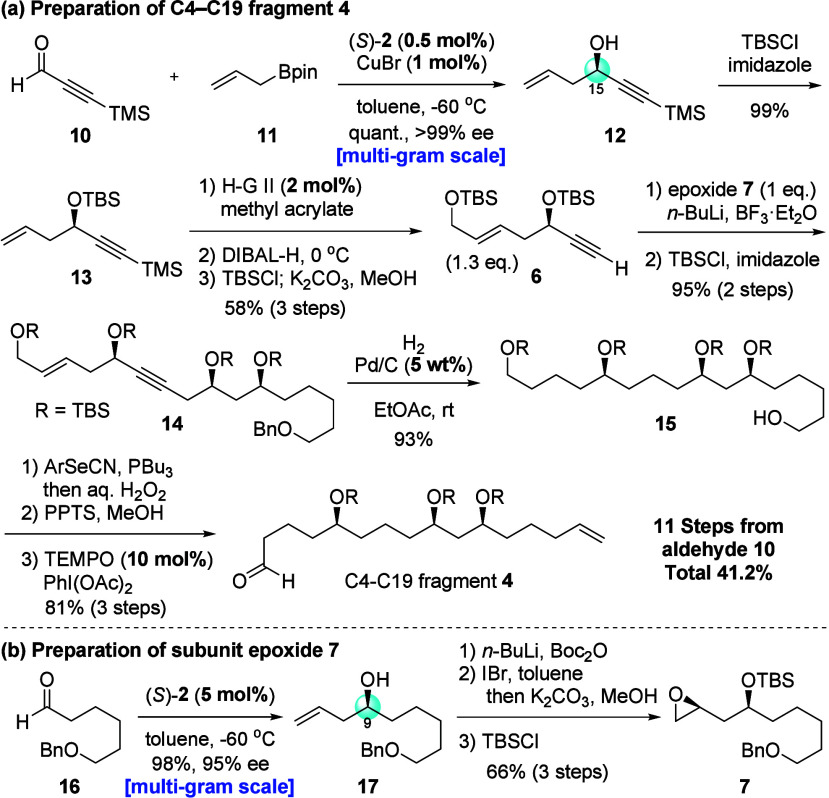
Preparation of C4–C19 fragment **4** and subunit epoxide **7**.

Next, we synthesized the C20–C43 fragment **5** (Figure 4a). The construction of **5** started with a chiral phosphoramide-catalyzed enantioselective allylation of pivalaldehyde (**18**) with allylboronic reagent **11**. In our initial attempt, we used SPINOL-derived catalyst (*R*)-**2**, which generally gives the best results in the conventional enantioselective allylboration reaction with alkyl aldehydes,^21,43^ for the reaction with pivalaldehyde. The desired product was obtained in good yield but with poor enantioselectivity (40% ee).^44^ On the other hand, when we used phosphoramide catalyst (*S*)-**3**, which can promote multiple interactions with substrates for this reaction, the desired product was obtained in quantitative yield with excellent enantioselectivity.^24^ The reaction proceeded smoothly with only 0.5 mol % of catalyst in pentane, and the catalyst could be recovered in 99% yield and used again in the same reaction to give the desired product without any loss of chemical yield and enantioselectivity. Finally, more than 2.5 g (20 mmol) of enantioenriched homoallylic alcohol **19** was prepared using only 44 mg of the catalyst. Treatment of alcohol **19** with BzCl and pyridine, followed by cross-metathesis with crotonaldehyde using Hoveyda–Grubbs II catalyst, gave the corresponding α,β-unsaturated aldehyde in good yield. The obtained aldehyde was converted into homoallylic alcohol **9** via an asymmetric allylboration using (*S*)-**2** in a highly diastereoselective fashion without the influence of the Lewis basic benzoate moiety and the stereogenic center at C39.

**Figure 4:**
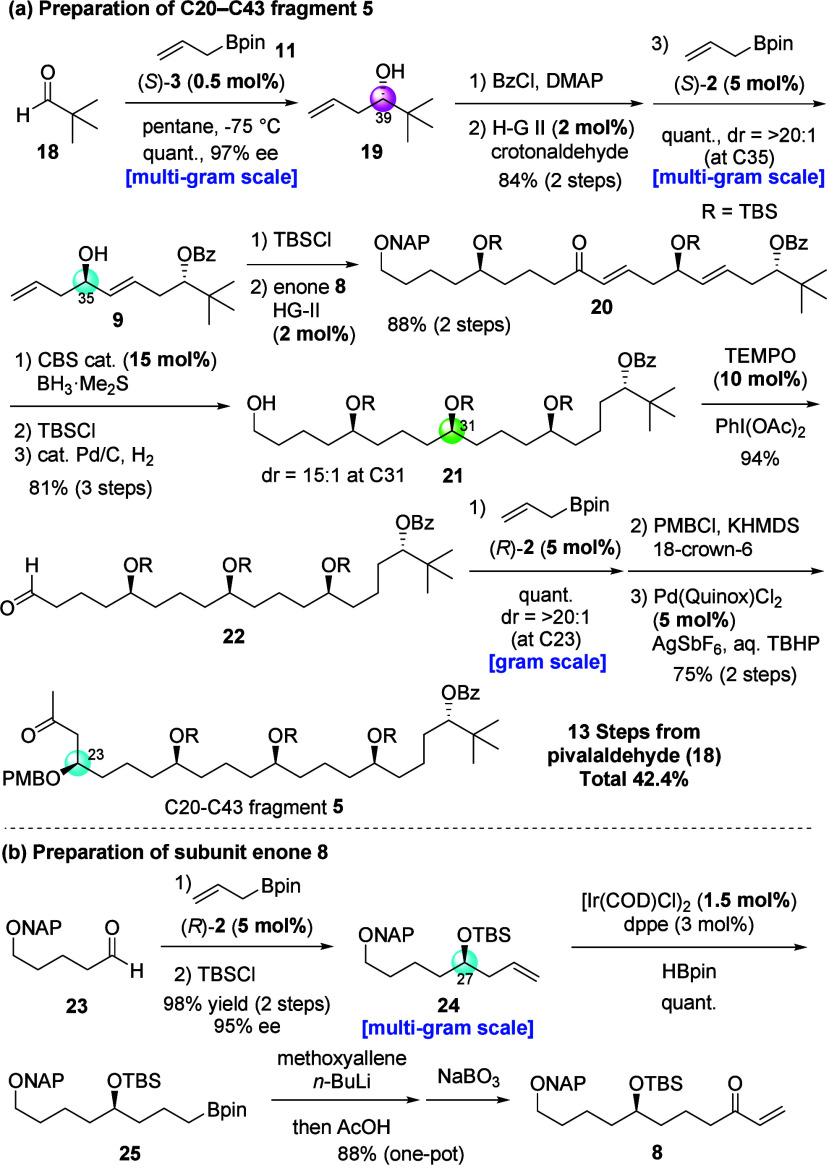
Preparation of C20–C43 fragment **5** and subunit enone **8**.

Next, we synthesized subunit enone **8** from readily available NAP-protected aldehyde **23** (Figure 4b). CPA-catalyzed allylboration followed by TBS protection gave silyl ether **24** in 98% yield with 95% ee. The Ir-catalyzed hydroboration of **24** furnished alkylboronic ester **25**, which was subsequently used in Aggarwal’s three-carbon homologation^45^ reaction to provide subunit **8** on a gram scale. Silylated subunit **9** was united with subunit enone **8** via olefin cross-metathesis using Hoveyda–Grubbs II catalyst to afford desired enone **20** in high yield. Exposing this enone to BH_3_·Me_2_S in the presence of (*S*)-oxazaborolidine (CBS catalyst) resulted in the formation of the stereocenter at C31 with high diastereoselectivity (dr = 15:1). Protection of the generated secondary alcohol by a TBS group followed by hydrogenation of olefins involving deprotection by the NAP group afforded primary alcohol **21**. After the TEMPO oxidation of **21**, generated aldehyde **22** was converted into C20–C43 fragment **5** through a three-step transformation: CPA-catalyzed asymmetric allylboration, PMB protection, and the modified Wacker oxidation.^46^ To our delight, the CPA-catalyzed allylation reaction of functionalized aldehyde **22** gave the corresponding product with excellent diastereoselectivity, despite having a benzoyl group that might interact with CPA and four stereocenters that might promote mismatch interactions with the catalyst. The overall yield for preparing C20–C43 fragment **5** was 42.4% in 13 steps from pivalaldehyde (**18**).

With ample quantities of C4–C19 fragment **4** and C20–C43 fragment **5**, we assembled these fragments through a diastereoselective aldol reaction (Figure 5). Treatment of **5** with Cy_2_BCl and Et_3_N furnished the corresponding boryl enolate. Adding aldehyde **4** gave the desired aldol adduct in 94% yield with excellent diastereoselectivity (dr = >20:1).^47–49^ Reduction of ketone **26** under Evans–Saksena conditions gave a 1,3-*anti* diol in good yield. The diol was subjected to protection using TBSOTf and lutidine to afford important intermediate **27** having complete stereogenic centers in gram quantities. Selective removal of the Bz group by reacting with DIBAL-H at −78 °C, followed by installation of the C1–C3 unit by using acid anhydride **28** under mild basic conditions, provided ester **29** having all of the carbons of bastimolide A (**1**). We proceeded with the construction of the 40-membered-ring system of **1** through an intramolecular Suzuki–Miyaura coupling reaction. We initially attempted to use Aggarwal’s conditions^50^ for the synthesis of bastimolide B but obtained a low yield (ca. 10%) because the iodide was eliminated, generating the corresponding triple bond competitively (see SI for details). After carefully investigating the reaction conditions, we obtained macrolactone **30** in 61% yield without any isomerization from *Z*-olefin to *E*-olefin under the modified conditions.^51,52^ Finally, the global deprotection of **30** was performed by treating with DDQ in pH 7 phosphate buffer and exposing the resulting mono alcohol to aqueous HF to furnish bastimolide A (**1**) in 80% yield (two steps). The ^1^H NMR, ^13^C NMR, HRMS, and optical rotation data of synthesized **1** are identical to those of the natural product and Smith’s synthetic **1**.^37^ Our synthetic route furnished this molecule with the longest linear sequence of 21 steps in 15.4% total yield (91.5% average yield). This scalable route produced >200 mg of bastimolide A (**1**).

**Figure 5:**
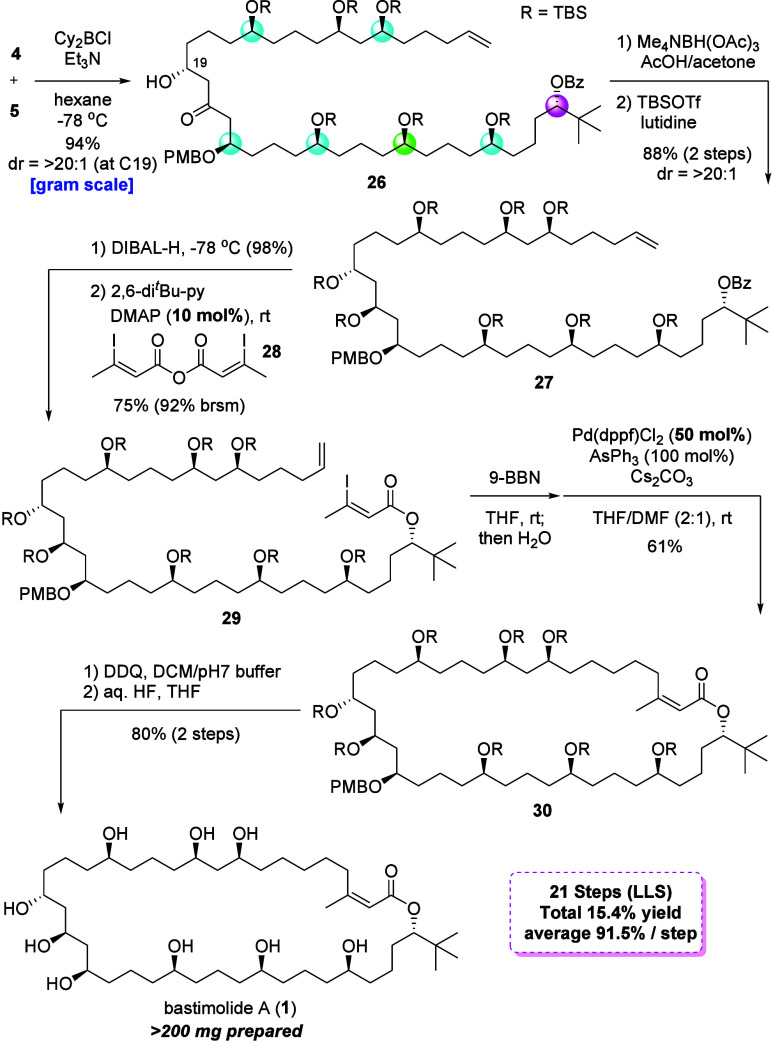
Total synthesis of bastimolide A (**1**).

In summary, we have achieved a total synthesis of bastimolide A in 21 steps (LLS) in a total yield of 15.4% from inexpensive starting materials, utilizing chiral Brønsted acid-catalyzed enantioselective and diastereoselective allylborations as the key steps. By establishing a method for recovering phosphoramide catalyst (*S*)-**3**, only 44 mg of (*S*)-**3** was required for synthesizing bastimolide A. As this method was also applicable to chiral phosphoric acid (*S*)-**2**, 360 mg of (*S*)-**2** was used to provide four chiral subunits **6**–**9** on a multigram scale, and 295 mg of (*S*)-**2** was recovered in pure form. These results indicated that the robustness of the chiral Brønsted acid catalysts and our protocol for recycling the catalysts contributed to scalability, as well as sustainability in the synthesis of bastimolide A. We also demonstrated that the CPA-catalyzed allylboration of functionalized substrates, such as **22**, gave the desired product in almost quantitative yield with excellent stereoselectivity. Careful optimization of the synthetic route enabled us to incorporate catalytic reactions in 10 of the 21 steps, reducing chemical waste and eliminating the need for reactions not only with excess amounts of heavy metals but also with stoichiometric amounts of chiral sources. Moreover, our synthetic route can catalytically construct 7 of the 10 stereogenic centers in bastimolide A, contributing to the synthesis of derivatives having different stereochemistry and substituents for biological and SAR studies. Further studies of the divergent total synthesis of bastimolide families and other related macrolides having 1,3,5-stereotriols and 1,5-stereodiols through chiral Brønsted acid-catalyzed enantioselective transformations are underway in our laboratory.

## Supplementary Material



## Notes

Experimental procedure and characterization data (PDF)

## ABBREVIATIONS:

LLS: longest linear sequence
DMAP: 4-dimethylaminopyridine
COD: 1,5-cyclooctadiene
NAP: 2-naphthylmethyl ether
brsm: based on recovered starting material
DDQ: 2,3-dichloro-5,6-dicyano-1,4-benzoquinone.
