# Supplementary material for: Scalable Total Synthesis of Bastimolide A Enabled by Asymmetric Allylborations Catalyzed by Chiral Brønsted Acids
Source: JACS Au. 2025 Jul 8;5(7):3052–7. doi: 10.1021/jacsau.5c00630 (PMC12308400; doi:10.1021/jacsau.5c00630)

Scalable Total Synthesis of Bastimolide A Enabled byAsymmetric Allylborations Catalyzed by Chiral Brønsted Acids

Shigenobu Umemiya,[1] Naoya Shinagawa,[2] Aisuke Fujimoto[2] and Masahiro Terada[2]*

[1]Research and Analytical Center for Giant Molecules, Graduate School of Science, Tohoku University, 6-3 Aramaki Aza Aoba, Aoba-ku, Sendai 980-8578, Japan

[2]Department of Chemistry, Graduate School of Science, Tohoku University 6-3 Aramaki-Aza Aoba-ku, Sendai, Miyagi 980-8578, Japan

Mail: mterada@tohoku.ac.jp

SUPPORTING INFORMATION

Table of Contents

1. General Information S2
2. Experimental Procedure S3
3. ^{1}H and ^{13}C NMR Comparison of Bastimolide A S35
4. Determination of the Stereochemistry (at C23, C27, C31, and C35) S37
5. Procedure of Recycle of Brønsted Acid Catalysts in Asymmetric Allylborations S41
6. Investigation of macrolactonization conditions S45
7. References S46
8. NMR Charts S47

1. General Information

All reactions were carried out under nitrogen atmosphere in flame-dried glassware. Dichloromethane (DCM), diethyl ether (Et_2O), and tetrahydrofuran (THF) were supplied from KANTO Chemical Co., Inc. as "Dehydrated solvent system". Other solvents and reagents were purchased from commercial suppliers and used without further purification. Purification of reaction products was carried out by flash column chromatography using silica gel 60 N (Merck 40-63 μm). Analytical thin layer chromatography (TLC) was performed on Merck precoated TLC plates (silica gel 60 GF 254, 0.25 mm). ^{1}H NMR spectra were recorded on a JEOL ECA-600 (600 MHz) spectrometer. Chemical shifts are reported in ppm from tetramethylsilane or solvent resonance as the internal standard ($CDCl_3$: 7.26 ppm, TMS: 0.00 ppm). ^{13}C NMR spectra were recorded on a JEOL ECA-600 (151 MHz) spectrometer with complete proton decoupling. Chemical shifts are reported in ppm from the solvent resonance as the internal standard ($CDCl_3$: 77.0 ppm). Infrared spectra were recorded on a Jasco FT/IR-4100 spectrometer. Chiral stationary phase HPLC analysis was performed on a Jasco LC-2000 Plus Series system with DAICEL chiral analytical column (4.6 mmΦ* 250 mm length). Optical rotations were measured on a Jasco P1020 digital polarimeter with a sodium lamp and reported as follows; $[\alpha]^{T\,°C}_{D}$ (c = g/100 mL, solvent). Mass spectra analysis using ESI ionization method was performed on a Bruker Daltonics solariX 9.4T spectrometer at the Research and Analytical Center for Giant Molecules, Graduate School of Science, Tohoku University.

2. Experimental Procedure

Alcohol 12

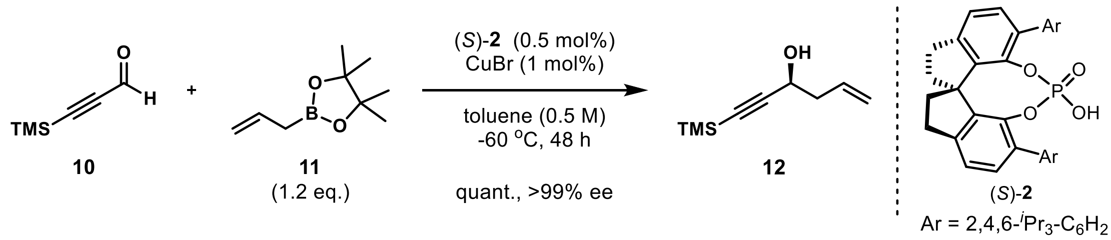

To a solution of aldehyde **10** (2.5 g, 20 mmol), (*S*)-**2** (72 mg, 0.10 mmol, 0.5 mol%) and CuBr (29 mg, 0.20 mmol, 1.0 mol%) in toluene (40 mL, 0.5 M) was added allylboronic acid pinacol ester (4.53 mL, 24 mmol, 1.2 eq.) at -60 °C under an atmosphere of nitrogen and the solution was stirred at this temperature for 48 h. The reaction mixture was quenched with aq. $NaHCO_3$ and then diluted with EtOAc. The aqueous phase was extracted with EtOAc (3 x 5 mL), the organic fractions were combined, washed with brine, dried over Na_2SO_4, filtered and concentrated *in vacuo*. The resultant residue was purified by column chromatography on silica gel (Hexane/EtOAc = 40/1 to 20/1) to give **12** (3.4 g, 20 mmol) in quantitative yield as a colorless oil. All spectroscopic data for **12** (^{1}H NMR, ^{13}C NMR, IR, HRMS, and $[\alpha]_D$ were identical to the known compound.[1]

Silyl ether 13

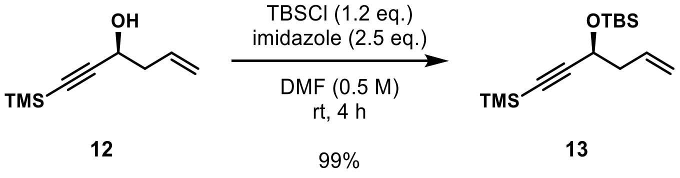

To alcohol **12** (2.52 g, 15 mmol) and imidazole (2.55 g, 37.5 mmol, 2.5 eq.) in DMF (30 mL, 0.5 M) was added TBSCl (2.71 g, 18 mmol, 1.2 eq.) at 0 °C under an atmosphere of nitrogen and the reaction was stirred at room temperature for 4 h. The reaction mixture was diluted with Et_2O and then quenched with aq. $NaHCO_3$. The phases were separated and the aqueous phase was extracted with Et_2O (3 x 20 mL). The organic fractions were combined, washed with water then brine, dried over $MgSO_4$, filtered and concentrated *in vacuo*. The resultant residue was purified by flash column chromatography on silica gel (Hexane/EtOAc = 100/1 to 50/1) to give **13** (4.20 g, 14.9 mmol) in 99% yield as a colorless oil. All spectroscopic data for **00** (^{1}H NMR, ^{13}C NMR, IR, and HRMS) were identical to the known compound.[1]

Methyl ester S1

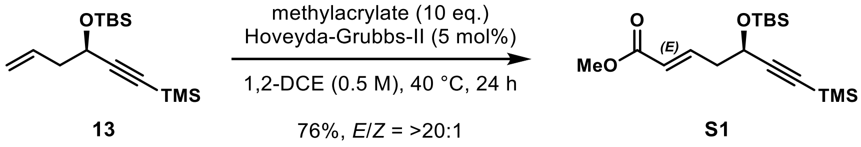

To a solution of alkene **13** (4.23 g, 15 mmol) and methyl acrylate (12.9 mL, 150 mmol, 10 eq.) in 1,2-DCE (30 mL, 0.5 M) was added Hoveyda-Grubbs 2nd generation catalyst (470 mg, 0.75 mmol, 5.0 mol%) under an atmosphere of argon at 40 °C. The reaction mixture was stirred for 24 h at this temperature. The resulting mixture was concentrated under reduced pressure and the residue was purified by flush column chromatography (Hexane/EtOAc =200/1 to 50/1) to afford methyl ester **S1** (3.88 g, 11.4 mmol) in 76% yield, *E*/*Z* = >20:1 as a colorless oil.

R_f = 0.66 (Hexane/EtOAc = 5/1).
$[\alpha]_D^{26}$: +31.9 (c = 0.59, $CHCl_3$).
^{1}H NMR (600 MHz, $CDCl_3$) δ 6.96 (dt, *J* = 15.6, 7.2 Hz, 1H), 5.96 (dt, *J* = 15.6, 1.8 Hz, 1H), 4.34 (t, *J* = 6.0 Hz, 1H), 3.74 (s, 3H), 2.58-2.52 (m, 2H), 0.89 (s, 9H), 0.16 (s, 9H), 0.13 (s, 3H), 0.11 (s, 3H).
^{13}C NMR ($CDCl_3$, 151 MHz) δ 166.7, 144.5, 123.4, 106.2, 89.8, 62.2, 51.5, 41.3, 25.7 (3C), 18.2, 0.27 (3C), -4.5, -5.0.
IR (ATR): 2956, 2176, 1729, 1251, 1167, 1088, 837, 778, 758 cm^{-1}.
HRMS (ESI) m/z: $[M+Na]^+$ Calcd for $C_{17}H_{32}NaO_3Si_2$ 363.17822; Found 363.17822.

Allylic alcohol S2

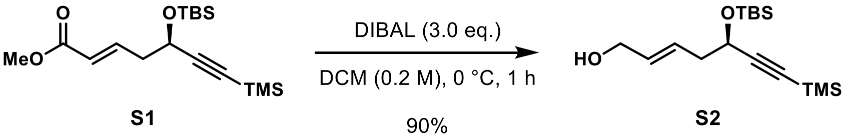

To a solution of methyl ester **S1** (3.41 g, 10 mmol) in DCM (50 mL, 0.2 M) was added DIBAL-H in hexanes (30 mL, 30 mmol 3.0 eq.) at 0 °C under an atmosphere of argon. The reaction mixture was stirred at this temperature for 20 min and quenched with EtOAc (20 mL) at 0 °C. The resulting mixture was stirred for 20 min at room temperature and sat. Rochelle's salt (20 mL) was added. The reaction mixture was stirred vigorously at room temperature for 12 h. Then the aqueous phase was separated and extracted with EtOAc (3 x 20 mL). The organic fractions were combined, washed with brine, dried over Na_2SO_4, filtered and concentrated *in vacuo*. The residue was purified by flash colomn chromatography on silica gel (Hexane/EtOAc = 20/1 to 15/1) to furnish **S2** (2.81 g, 9.0 mmol) in 90% yield as a colorless oil.

R_f = 0.29 (Hexane/EtOAc = 5/1).
$[\alpha]_D^{26}$: +26.3 (c = 0.36, $CHCl_3$).
^{1}H NMR (600 MHz, $CDCl_3$) δ 5.77-5.70 (m, 2H), 4.34 (t, *J* = 6.0 Hz, 1H), 4.11 (brs, 2H), 2.44-2.37 (m, 2H), 1.27 (brs, 1H), 0.90 (s, 9H), 0.16 (s, 9H), 0.13 (s, 3H), 0.11 (s, 3H).
^{13}C NMR ($CDCl_3$, 151 MHz) δ 132.2, 128.1, 107.0, 89.2, 63.7, 63.2, 41.4, 25.8 (3C), 18.3, -0.18 (3C), -4.5, -4.9.
IR (ATR): 3345, 2957, 2929, 2857, 2174, 1250, 1091, 969, 835, 777, 759 cm^{-1}.
HRMS (ESI) m/z: $[M+Na]^+$ Calcd for $C_{16}H_{32}NaO_2Si_2$ 335.18330; Found 335.18329.

Alkyne 6

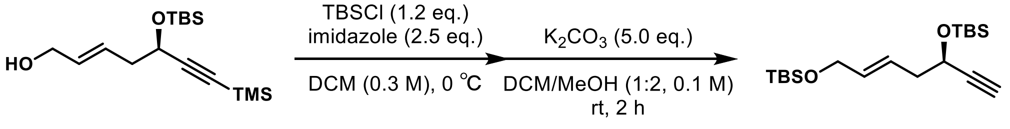

S2 85% 6

To a solution of allyl alcohol **S2** (1.56 g, 5 mmol, 1.0 eq.) and imidazole (510 mg, 7.5 mmol, 2.5 eq.) in DCM (16 mL, 0.3 M) was added TBSCl (904 mg, 6.0 mmol, 1.2 eq.) at 0 °C under an atmosphere of nitrogen. The reaction mixture was stirred at rt and monitored by TLC. After consumption of allyl alcohol, methanol (33 mL) was added at 0 °C followed by potassium carbonate (3.5 g, 25 mmol, 5.0 eq.). The reaction was allowed to warm to room temperature and stirred for 2 h. The reaction mixture was quenched with aq. $NaHCO_3$ at 0 °C and diluted with EtOAc (30 mL). The phases were separated and the aqueous phase was extracted with EtOAc (3 x 20 mL). The organic fractions were combined, washed with brine, dried over Na_2SO_4, filtered and concentrated *in vacuo*. The resultant residue was purified by flash column chromatography on silica gel (Hexane/EtOAc = 100/1 to 50/1) to give **6** (1.51 g, 4.25 mmol) in 85% yield as a colorless oil.

R_f = 0.77 (Hexane/EtOAc = 5/1).

$[\alpha]_D^{26}$: +15.9 (c = 0.58, $CHCl_3$).

^1H NMR (600 MHz, $CDCl_3$) δ 5.70 (dtt, *J* = 15.6, 6.6, 1.2 Hz, 1H), 5.64 (dt, *J* = 15.6, 4.8 Hz, 1H), 4.34 (td, *J* = 6.6, 1.8 Hz, 1H), 4.14 (dd, *J* = 4.8, 1.2 Hz, 2H), 2.42 (d, *J* = 6.6 Hz, 2H), 2.39 (d, *J* = 1.8 Hz, 1H), 0.91 (s, 9H), 0.90 (s, 9H), 0.13 (s, 3H), 0.10 (s, 3H), 0.07 (s, 6H).

^{13}C NMR ($CDCl_3$, 151 MHz) δ 132.6, 125.7, 85.2, 72.4, 63.7, 62.7, 41.5, 26.0 (3C), 25.8 (3C), 18.4, 18.2, -4.6, -5.0, -5.2 (2C).

IR (ATR): 3312, 2929, 2857, 2349, 1473, 1253, 969, 834, 775 cm^{-1}.

HRMS (ESI) m/z: $[M+Na]^+$ Calcd for $C_{19}H_{38}NaO_2Si_2$ 377.23025; Found 377.23024.

5-(benzyloxy)pentan-1-ol

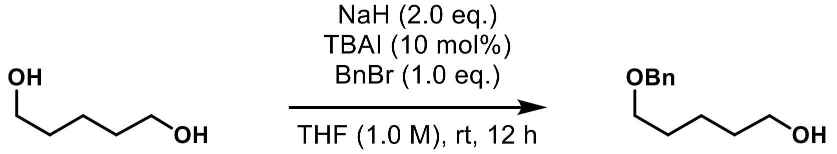

90%

To a solution of 1,6-hexanediol (2.36 g, 20 mmol, 1.0 eq.) in THF (20 mL) was added 60% NaH (1.6 g, 40 mmol, 2.0 eq.) portionwise at 0 °C under an atmosphere of nitrogen. The resulting suspension was stirred for 1 h at this temperature. Then benzyl bromide (2.38 mL, 20 mmol, 1.0 eq.) was added to the reaction mixture dropwise followed by the addition of tetrabutylammonium iodide (738 mg, 2.0 mmol, 10 mol%) in one portion. The reaction was allowed to warm to room temperature and stirred for 12 h. The reaction was then poured into sat. NH_4Cl (50 mL). The phases

were separated and the aqueous phase was extracted with EtOAc (3 x 20 mL). The organic fractions were combined, washed with brine, dried over Na_2SO_4, filtered and concentrated in vacuo. The residue was purified by column chromatography on silica gel (Hexane/EtOAc = 5/1 to 2/1) to give 5-(benzyloxy)pentan-1-ol (3.75 g, 18.4 mmol) in 90% yield as a colorless oil. All spectroscopic data for 5-(benzyloxy)pentan-1-ol (^{1}H NMR, ^{13}C NMR, IR, and HRMS) were identical to the known compound.[2]

Aldehyde 16

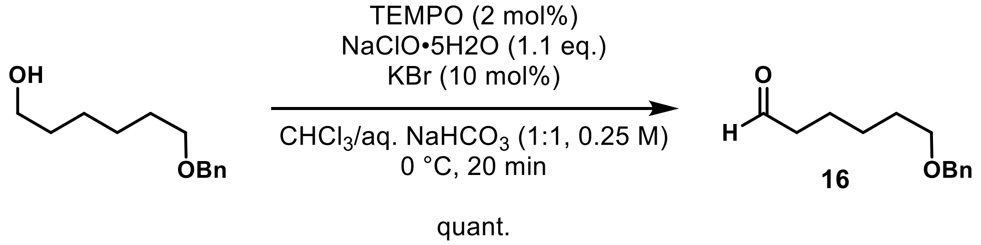

To a biphasic mixture of 5-(benzyloxy)pentan-1-ol (4.16 g, 20 mmol), 2,2,6,6-tetramethylpiperidine-1-oxyl (TEMPO) (62.6 mg, 0.40 mmol, 2.0 mol%) in $CHCl_3$ (40 mL, 0.5 M) and KBr (238 mg, 2.0 mmol, 10 mol%) in sat. $NaHCO_3$ (20 mL) was added $NaClO \cdot 5H_2O$ (3.62 g, 22 mmol, 1.1 eq.) in sat. $NaHCO_3$ (20 mL) dropwise over 5 min at 0 °C with vigorous stirring. The reaction mixture was stirred vigorously for additional 15 min at this temperature. The reaction was quenched with sat. $Na_2S_2O_3$ (40 mL) and stirred for 10 min. The phases were separated and the aqueous phase was extracted with DCM (3 x 20 mL). The organic layers were combined, dried over $MgSO_4$, filtered and concentrated *in vacuo*. The crude material was purified by flash column chromatography on silica gel (Hexane/EtOAc =20/1 to 5/1) to furnish aldehyde **16** (4.11 g, 20 mmol) in quantitative yield as a colorless oil. All spectroscopic data for **16** (^{1}H NMR, ^{13}C NMR, IR, and HRMS) were identical to the known compound.[3]

Homoallyl alcohol 17

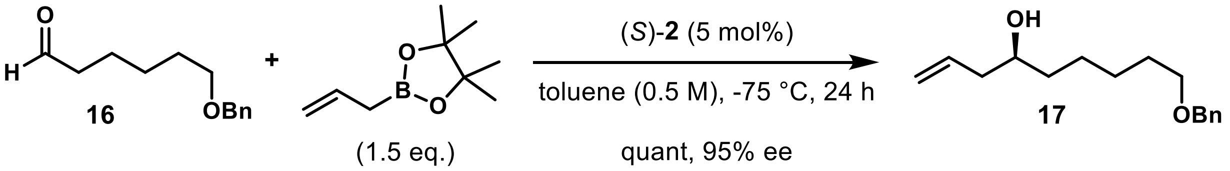

To a solution of aldehyde **16** (2.06 g, 10 mmol, 1.0 eq.) and (*S*)-**2** (359 mg, 0.5 mmol, 5 mol%) in toluene (12 mL) was added allylboronicacid pinacolester (2.52 g, 15 mmol, 1.5 eq.) in toluene (8.0 mL) dropwise at -75 °C under an Ar atmosphere. The reaction mixture was stirred for 24 h at this temperature and allowed to warm to 0 °C. *N*-methyl-2-amino-ethanol (0.96 mL, 12 mmol, 1.2 eq.) was added to the mixture. The resulting suspension was stirred for 1 h at ambient temperature. The reaction was diluted with hexane (24 mL) and filtered through a pad of celite. The remaining white solid and celite cake was rinsed with hexane (2 x 24 mL). The filtrate was washed with 1N HCl then brine, dried over Na_2SO_4, filtered and concentrated under reduced pressure. The residue was purified by flash column chromatography on silica gel (Hexane/EtOAc = 20/1 to 5/1) to obtain homoallylic alcohol **17** (2.48 g, 10 mmol) in

quantitative yield, 95% ee as a colorless oil. (*S*)-**2** was recovered as a salt by eluting Hexane/EtOAc = 4/1 to 2/1. The recovered salt was dissolved in DCM (20 mL) and washed by 6N HCl (2 x 20 mL) and dried under high-vacuum to recover (*S*)-**2** (355 mg, 0.49 mmol) in 98% yield as a white solid. All spectroscopic data for **17** (^{1}H NMR, ^{13}C NMR, IR, HRMS, and [a]$_D$ were identical to the known compound.[4]

Carbonate S3

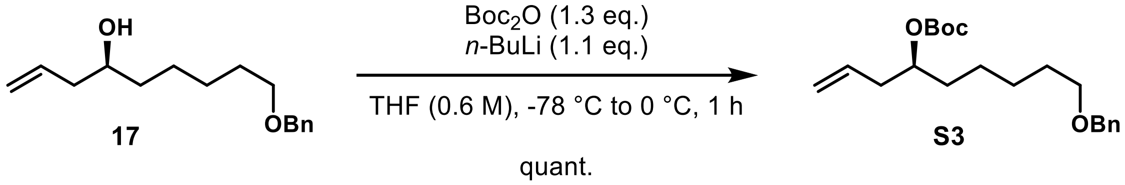

To a solution of secondary alcohol **17** (2.98 g, 12 mmol, 1.0 eq.) in THF (12 mL) was added *n*-BuLi in hexane (1.6 M, 8.25 mL, 13.2 mmol, 1.1 eq.) at -78 °C under Ar atmosphere. The reaction mixture was stirred for 10 min before di-tert-butyl dicarbonate (3.40 g, 15.6 mmol, 1.3 eq.) in THF (8.0 mL) was added dropwise. The reaction was allowed to warm to 0 °C and stirred for 1 h. The reaction was quenched with sat. NH_4Cl (20 mL) and allowed to warm to room temperature. The phases were separated and the aqueous phase was extracted with EtOAc (3 x 20 mL). The organic fractions were combined, washed with brine, dried over Na_2SO_4, filtered and concentrated in vacuo. The residue was purified by column chromatography on silica gel (Hexane/EtOAc = 30/1 to 20/1) to give **S3** (4.18 g, 12 mmol) in quantitative yield as a colorless oil.

R_f = 0.67 (Hexane/EtOAc = 5/1).
$[\alpha]_D^{21}$: −14.5 (c = 0.51, $CHCl_3$).
^{1}H NMR (600 MHz, $CDCl_3$) δ 7.36-7.26 (m, 5H), 5.81-5.74 (ddt, *J*=16.8, 10.2, 7.2 Hz, 1H), 5.11-5.05 (m, 2H), 4.70-4.66 (m, 1H), 4.49 (s, 2H), 3.45 (t, *J*= 6.6 Hz, 2H), 2.33 (t, *J*= 6.6 Hz, 2H), 1.64-1.53 (m, 4H), 1.47 (s, 9H), 1.41-1.29 (m, 4H).
^{13}C NMR ($CDCl_3$, 151 MHz) δ 153.3, 138.5, 133.5, 128.2 (2C), 127.5 (2C), 127.3, 117.6, 81.4, 76.3, 72.8, 70.1, 38.6, 33.5, 29.5, 27.7 (3C), 25.9, 25.0.
IR (ATR): 2936, 2859, 1736, 1367, 1277, 1253, 1162, 1095, 753, 697 cm^{-1}.
HRMS (ESI) m/z: $[M+Na]^+$ Calcd for $C_{21}H_{32}NaO_4$ 371.21928; Found 371.21927.

Epoxide S4

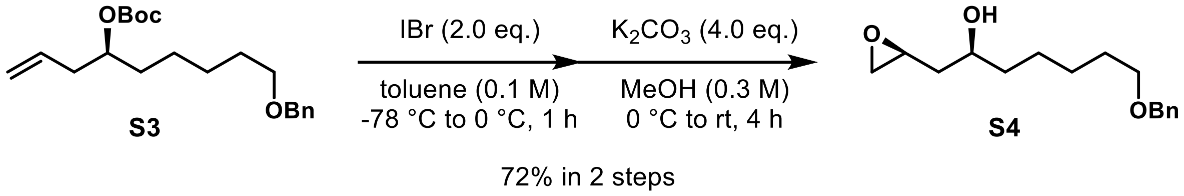

To a solution of carbonate **S3** (3.92 g, 11.2 mmol) in toluene (112 mL, 0.1 M) was added IBr in DCM (74 mL, ca.

0.3 M, 2.0 eq.) at -78 °C under Ar atmosphere. The reaction mixture was stirred for 3 h and allowed to warm to 0 °C. The resulting mixture was stirred for additional 30 min and then poured into an ice-cooled sat. $NaHCO_3$/aq. $Na_2S_2O_3$ (1:1, 200 mL). The aqueous layer was separated and extracted with ethyl acetate (1 x 200 mL). The combined organic layers were washed with brine, dried over Na_2SO_4, and concentrated to give an unstable yellow oil. This crude material was immediately subjected to the next step without further purification.

To a solution of above crude material in MeOH (56 mL) was added K_2CO_3 (6.5 g, 44.8 mmol, 4.2 eq.) at 0 °C under Ar atmosphere. The reaction mixture was stirred for 1 h at this temperature and for additional 2 hours at room temperature. The resulting mixture was quenched with aq. $NaHCO_3$/aq. $Na_2S_2O_3$ (1:1, 100 mL). The aqueous phase was extracted with Et_2O (3 x 50 mL), the organic fractions were combined, washed with brine, dried over $MgSO_4$, filtered and concentrated in vacuo. The resultant residue was purified by flash column chromatography on silica gel (Hexane/EtOAc = 4/1 to 2/1) to give **S4** (2.12 g, 8.02 mmol) in 72% yield as a colorless oil.

R_f = 0.07 (Hexane/EtOAc = 5/1).
$[\alpha]_D^{22}$: −8.12 (c = 0.53, $CHCl_3$).
^1H NMR (600 MHz, $CDCl_3$) δ 7.35-7.27 (m, 5H), 4.50 (s, 2H), 3.91-3.85 (m, 1H), 3.47 (t, *J* = 6.6 Hz, 2H), 3.08 (m, 1H), 2.78 (dd, *J* = 5.4, 4.2 Hz, 1H), 2.50 (dd, *J* = 5.4, 3.0 Hz, 1H), 2.09 (brs, 1H), 1.87-1.83 (ddd, *J* = 14.4, 4.2, 3.6 Hz, 1H), 1.66-1.61 (m, 2H), 1.53-1.34 (m, 7H).
^{13}C NMR ($CDCl_3$, 151 MHz) δ 138.6, 128.4 (2C), 127.7 (2C), 127.5, 72.9, 70.5, 70.3, 50.7, 46.6, 39.7, 37.5, 29.7, 26.2, 25.3.
IR (ATR): 3444, 2932, 2858, 1454, 1363, 1095, 1027, 736, 698 cm^{-1}.
HRMS (ESI) m/z: $[M+Na]^+$ Calcd for $C_{16}H_{24}NaO_3$ 287.16177; Found 287.16175.

Epoxide 7

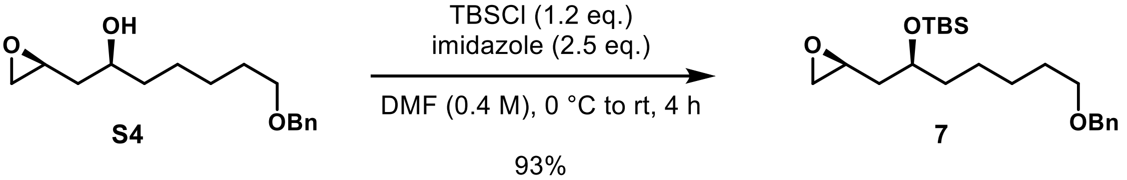

To a solution of epoxide **S4** (2.19 g, 8.24 mmol, 1.0 eq.) and imidazole (1.40 g, 20.5 mmol, 2.5 eq.) in DMF (15 mL, 0.4 M) was added TBSCl (1.50 g, 9.84 mmol, 1.2 eq.) at 0 °C. The reaction mixture was allowed to warm to room temperature and stirred for 4 h. The reaction mixture was quenched with aq. $NaHCO_3$ (20 mL) and then diluted with Et_2O (20 mL). The phases were separated and the aqueous phase was extracted with Et_2O (3 x 20 mL). The organic fractions were combined, washed with brine, dried over $MgSO_4$, filtered and concentrated *in vacuo*. The residue was purified by column chromatography on silica gel (Hexane/EtOAc= 40/1 to 25/1) to furnish epoxide **7** (2.89 g, 7.62 mmol) in 93% yield as a colorless oil.

R_f = 0.58 (Hexane/EtOAc = 5/1).

$[\alpha]_D^{21}$: −6.68 (c = 1.00, $CHCl_3$).
^1H NMR (600 MHz, $CDCl_3$) δ 7.35-7.26 (m, 5H), 4.50 (s, 2H), 3.84-3.83 (m, 1H), 3.47 (t, *J* = 6.6 Hz, 2H), 3.04-3.02 (m, 1H), 2.75 (dd, *J* = 4.8, 4.2 Hz, 1H), 2.45 (dd, *J* = 4.8, 3.0 Hz, 1H), 1.73-1.69 (ddd, *J* = 13.8, 6.0, 6.0 Hz, 1H), 1.65-1.51 (m, 5H), 1.41-1.25 (m, 4H), 0.89 (s, 9H), 0.050 (s, 3H), 0.045 (s, 3H).
^{13}C NMR ($CDCl_3$, 151 MHz) δ 138.6, 128.4 (2C), 127.6 (2C), 127.5, 72.9, 70.4, 70.3, 49.5, 46.8, 40.1, 37.1, 29.8, 26.3, 25.8 (3C), 25.3, 18.0, -4.5, -4.6.
IR (ATR): 2929, 2856, 1463, 1360, 1254, 1099, 1075, 834, 773 cm^{-1}.
HRMS (ESI) m/z: $[M+Na]^+$ Calcd for $C_{22}H_{38}NaO_3Si$ 401.24824; Found 401.24823.

Secondary alcohol S5

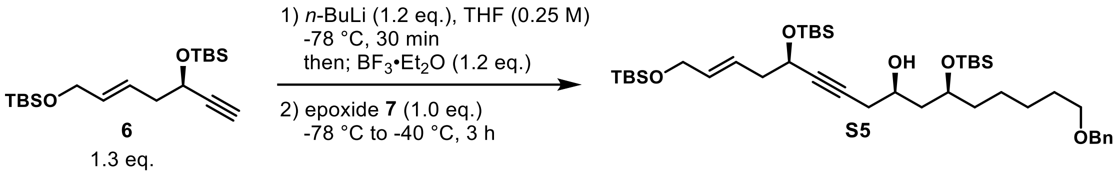

To a solution of alkyne **6** (2.31 g, 6.5 mmol, 1.3 eq.) in THF (26 mL) was added *n*-BuLi in hexane (1.6 M, 3.75 mL, 1.2 eq.) at -78 °C under Ar atmosphere. After stirring it for 30 min, $BF_3 \cdot Et_2O$ (750 μL, 6.0 mmol, 1.2 eq.) was added dropwise to the reaction. The resulting mixture was stirred for an additional 30 min at this temperature and then added epoxide **7** (1.89 g, 5.0 mmol, 1.0 eq.) in THF (16 mL). The reaction mixture was stirred for 2 h at -78 °C and additional 1 h at -40 °C. The mixture was then quenched with sat. NH_4Cl (50 mL). The phases were separated and the aqueous phase was extracted with EtOAc (3 x 20 mL), the organic fractions were combined, washed with brine, dried over Na_2SO_4, filtered and concentrated *in vacuo*. The resultant residue was purified by column chromatography on silica gel (Hexane/EtOAc = 25/1 to 15/1) to provide **S5** (3.65 g, 4.98 mmol) in 99% yield as a colorless oil.

R_f = 0.48 (Hexane/EtOAc = 5/1).
$[\alpha]_D^{24}$: +11.4 (c = 0.81, $CHCl_3$).
^1H NMR (600 MHz, $CDCl_3$) δ 7.36-7.27 (m, 5H), 5.69 (dtt, *J* = 15.6, 6.6, 1.8 Hz, 1H), 5.61 (dt, *J* = 15.6, 4.8 Hz, 1H), 4.50 (s, 2H), 4.34 (tt, *J* = 6.6, 1.8 Hz, 1H), 4.13 (dd, *J* = 4.8, 1.2 Hz, 2H), 3.93-3.89 (m, 1H), 3.86-3.82 (m, 1H), 3.47 (t, *J* = 6.6 Hz, 2H), 3.29 (brs, 1H), 2.41 (ddd, *J* = 16.8, 5.4, 1.8 Hz, 1H), 2.38 (dd, *J* = 6.6, 6.6 Hz, 2H), 2.33 (ddd, *J* = 16.8, 6.6, 1.8 Hz, 1H), 1.79 (ddd, *J* = 14.4, 4.2, 3.0 Hz, 1H), 1.63-1.49 (m, 5H), 1.40-1.27 (m, 4H), 0.90 (s, 9H), 0.89 (s, 18H), 0.11 (s, 3H), 0.10 (s, 3H), 0.09 (s, 6H), 0.06 (s, 6H).
^{13}C NMR ($CDCl_3$, 151 MHz) δ 138.6, 132.3, 128.6 (2C), 127.6 (2C), 127.5, 126.2, 83.8, 81.1, 72.9, 72.7, 70.3, 69.6, 63.7, 63.1, 41.9, 41.8, 37.8, 29.8, 27.6, 26.5, 26.0 (3C), 25.8 (6C), 24.5, 18.4, 18.3, 17.9, -4.0, -4.5, -4.7, -4.9, -5.2 (2C).
IR (ATR): 3512, 2951, 2856, 1471, 1462, 1254, 1088, 834, 774, 756 cm^{-1}.
HRMS (ESI) m/z: $[M+Na]^+$ Calcd for $C_{41}H_{76}NaO_5Si_3$ 755.48928; Found 755.48930.

Silyl ether 14

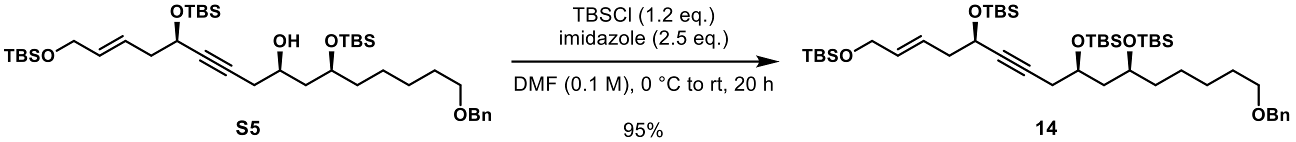

To a solution of alcohol **S5** (2.20 g, 3.0 mmol) and imidazole (511 mg, 7.5 mmol, 2.5 eq.) in DMF (30 mL, 0.1 M) was added TBSCl (543 mg, 3.6 mmol, 1.2 eq.) at 0 °C. The reaction mixture was allowed to warm to room temperature and stirred for 20 h. The reaction mixture was quenched with sat. $NaHCO_3$ (20 mL) and then diluted with Et_2O (30 mL). The phases were separated and the aqueous phase was extracted with Et_2O (3 x 20 mL). The organic fractions were combined, washed with water and brine, dried over $MgSO_4$, filtered and concentrated *in vacuo*. The residue was purified by flash column chromatography on silica gel (Hexane/EtOAc= 50/1) to furnish silyl ether **14** (2.41 g, 2.45 mmol) in 95% yield as a pale-yellow oil.

R_f = 0.76 (Hexane/EtOAc = 5/1).
$[\alpha]_D^{26}$: −3.89 (c = 0.74, $CHCl_3$).
^{1}H NMR (600 MHz, $CDCl_3$) δ 7.36-7.26 (m, 5H), 5.69 (dtt, *J* = 15.6, 7.2, 1.2 Hz, 1H), 5.62 (dt, *J* = 15.6, 5.4 Hz, 1H), 4.50 (s, 2H), 4.31 (tt, *J* = 6.6, 1.8 Hz, 1H), 4.13 (dd, *J* = 5.4, 1.2 Hz, 2H), 3.82-3.79 (m, 1H), 3.77-3.73 (m, 1H), 3.47 (t, *J* = 6.6 Hz, 2H), 2.37 (m, 2H), 2.33 (dd, *J* = 6.0, 1.8 Hz, 2H), 1.75 (ddd, *J* = 13.8, 7.2, 4.8 Hz, 1H), 1.65 (ddd, *J* = 13.8, 7.8, 6.0 Hz, 1H), 1.64-1.60 (m, 2H), 1.50-1.27 (m, 4H), 0.90 (s, 9H), 0.89 (s, 9H), 0.882 (s, 9H), 0.880 (s, 9H), 0.11 (s, 3H), 0.08 (s, 3H), 0.07 (s, 3H), 0.06 (s, 6H), 0.044 (s, 3H), 0.042 (s, 3H), 0.03 (s, 3H).
^{13}C NMR ($CDCl_3$, 151 MHz) δ 138.7, 132.1, 128.3 (2C), 127.6 (2C), 127.5, 126.5, 83.3, 81.4, 72.9, 70.5, 69.3, 68.5, 63.9, 63.1, 44.5, 42.0, 36.9, 29.8, 28.0, 26.5, 26.0 (3C), 25.9 (3C), 25.8 (3C), 25.8 (3C), 25.1, 18.4, 18.3, 18.1, 18.0, -4.23, -4.35, -4.4 (2C), -4.7, -5.0, -5.1 (2C).

IR (ATR): 2928, 2856, 2376, 2320, 1473, 1462, 1362, 1254, 1092, 833, 773, 758 cm^{-1}.

HRMS (ESI) m/z: $[M+Na]^+$ Calcd for $C_{47}H_{90}NaO_5Si_4$ 869.57575; Found 869.57578.

Primary alcohol 15

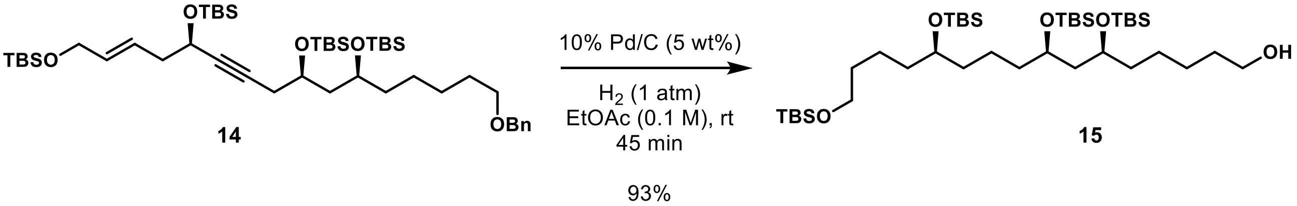

To a solution of silyl ether **14** (2.20 g, 2.6 mmol, 1.0 eq.) in EtOAc (26 mL, 0.1 M) was added 10% Pd/C (110 mg,

5 wt.%) under an atmosphere of nitrogen. The reaction vessel was parged with hydrogen for three times and stirred vigorously under hydrogen atmosphere for 45 min at ambient temperature. The reaction vessel was parged with Ar and filtered through a pad of celite. The filtrate was concentrated under reduced pressure and the residue was purified by flash column chromatography on silica gel (Hexane/EtOAc = 30/1 to 15/1) to afford primary alcohol **15** (1.85 g, 2.42 mmol) in 93% yield as a colorless oil.

R_f = 0.45 (Hexane/EtOAc = 5/1).
$[\alpha]_D^{21}$: −2.79 (c = 0.52, $CHCl_3$).
^1H NMR (600 MHz, $CDCl_3$) δ 3.75-3.70 (m, 2H), 3.64 (t, *J* = 6.6 Hz, 2H), 3.64-3.60 (m, 1H), 3.60 (t, *J* = 6.6 Hz, 2H), 1.65-1.23 (m, 22H), 0.89 (s, 9H), 0.882 (s, 18H), 0.880 (s, 9H), 0.05 (s, 6H), 0.04 (s, 6H), 0.03 (s, 12H).
^{13}C NMR ($CDCl_3$, 151 MHz) δ 72.3, 69.59, 69.55, 63.3, 63.0, 44.8, 37.7, 37.5, 37.2, 36.9, 33.1, 32.8, 26.03, 26.00 (3C), 25.95 (3C), 25.91 (6C), 24.9, 21.7, 20.8, 18.4, 18.12, 18.06 (2C), -4.26, -4.31, -4.37, -4.40, -4.42 (2C), -5.27 (2C).
IR (ATR): 3341, 2928, 2857, 1472, 1462, 1254, 1099, 1050, 833, 771 cm^{-1}.
HRMS (ESI) m/z: $[M+Na]^+$ Calcd for $C_{40}H_{90}NaO_5Si_4$ 785.57575; Found 785.57579.

Alkene S6

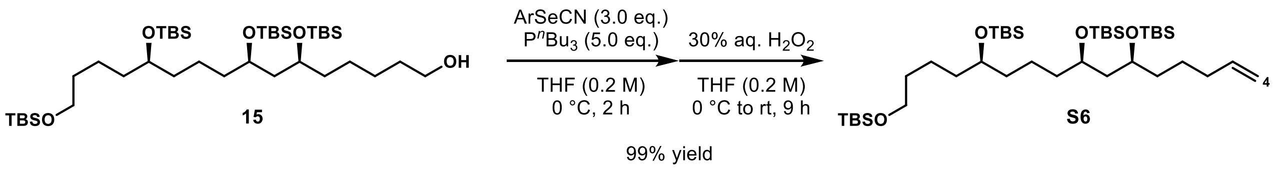

To a solution of primary alcohol **15** (1.72 g, 2.26 mmol, 1.0 eq.) in THF (22.6 mL, 0.1 M) was added 2-nitrophenyl-selenocyanate (1.54 g, 6.78 mmol, 3.0 eq.) and tributylphosphine (2.29 g, 11.3 mmol, 5.0 eq.) sequentially at 0 °C under Ar atmosphere. The reaction mixture was stirred for 30 min and aq. H_2O_2 (30%, 22.6 mL) was added. The reaction mixture was allowed to warm to rt and stirred for 9 h. The phases were separated and the aqueous phase was extracted with EtOAc (3 x 20 mL). The organic fractions were combined, washed with water (2 x 30 mL) and brine, dried over Na_2SO_4, filtered and concentrated *in vacuo*. The crude material was purified by flash column chromatography on silica gel (hexane/EtOAc =100/0 to 50/1) to give **S6** (1.67 g, 2.24 mmol) in 99% yield as a colorless oil.

R_f = 0.93 (Hexane/EtOAc = 5/1).
$[\alpha]_D^{21}$: −1.49 (c = 0.40, $CHCl_3$).
^1H NMR (600 MHz, $CDCl_3$) δ 5.80 (ddt, *J* = 17.4, 10.2, 6.6 Hz, 1H), 5.00 (ddt, *J* = 17.4, 1.8, 1.8 Hz, 1H), 4.94 (ddt, *J* = 10.2, 1.8, 1.2 Hz, 1H), 3.75-3.70 (m, 2H), 3.64-3.60 (m, 1H), 3.60 (t, *J* = 6.6 Hz, 2H), 2.04 (dt, *J* = 6.6, 6.6 Hz, 2H), 1.65-1.61 (m, 1H), 1.53-1.25 (m, 17H), 0.89 (s, 9H), 0.882 (s, 18H), 0.880 (s, 9H), 0.05 (s, 6H), 0.038 (s, 3H), 0.036 (s, 6H), 0.033 (s, 9H).

^{13}C NMR ($CDCl_3$, 151 MHz) δ 138.9, 114.4, 72.3, 69.6, 69.5, 63.3, 44.8, 37.7, 37.5, 36.9, 36.6, 34.0, 33.1, 25.99 (3C), 25.95 (3C), 25.92 (6C), 24.4, 21.7, 20.8, 18.4, 18.12, 18.06 (2C), -4.26, -4.32, -4.37, -4.40, -4.42 (2C), -5.27 (2C).
IR (ATR): 2951, 2856, 2320, 1641, 1472, 1462, 1253, 1004, 833, 806, 771 cm^{-1}.
HRMS (ESI) m/z: $[M+Na]^+$ Calcd for $C_{40}H_{88}NaO_4Si_4$ 767.56519; Found 767.56521.

Primary alcohol S7

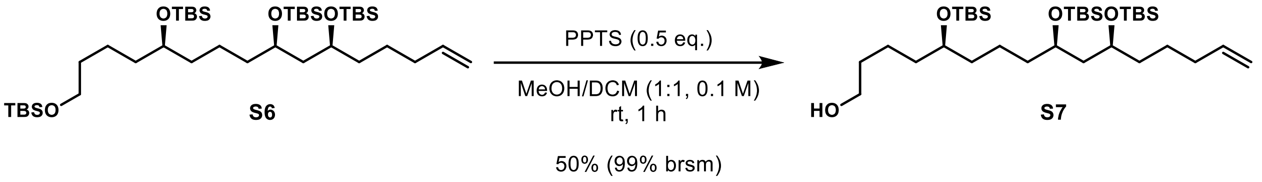

To a solution of silyl ether **S6** (2.01 g, 2.7 mmol, 1.0 eq.) in DCM/MeOH (1:1, 27 mL, 0.1 M) was added pyridinium *p*-toluenesulfonate (PPTS) (339 mg, 1.35 mmol, 50 mol%) at room temperature under Ar atmosphere. The reaction mixture was stirred for 1 h and quenched with sat. $NaHCO_3$ (30 mL). The phases were separated and the aqueous phase was extracted with EtOAc (3 x 30 mL). The organic fractions were combined, washed with brine, dried over Na_2SO_4, filtered and concentrated *in vacuo*. The crude material was purified by flash column chromatography on silica gel (hexane/EtOAc =100/1 to 25/1) to obtain **S7** (858 mg, 1.36 mmol) in 50% yield as a colorless oil.

R_f = 0.43 (Hexane/EtOAc = 5/1).
$[\alpha]_D^{22}$: −2.35 (c = 0.40, $CHCl_3$).
^1H NMR (600 MHz, $CDCl_3$) δ 5.80 (ddt, *J* = 17.4, 10.2, 6.6 Hz, 1H), 5.00 (ddt, *J* = 17.4, 1.8, 1.8 Hz, 1H), 4.95 (ddt, *J* = 10.2, 1.8, 1.2 Hz, 1H), 3.77-3.71 (m, 2H), 3.64 (t, *J* = 6.6 Hz, 2H), 3.65-3.61 (m, 1H), 2.04 (dt, *J* = 6.6, 6.6 Hz, 2H), 1.65-1.25 (m, 18H), 0.88 (s, 27H), 0.04 (s, 18H).
^{13}C NMR ($CDCl_3$, 151 MHz) δ 138.9, 114.4, 72.2, 69.6, 69.5, 63.0, 44.8, 37.7, 37.4, 36.7, 36.6, 34.0, 33.0, 25.9 (9C), 24.3, 21.4, 20.8, 18.13, 18.06 (2C), -4.26, -4.31, -4.37, -4.42 (3C).
IR (ATR): 3337, 2857, 2372, 2320, 1742, 1641, 1361, 1253, 1061, 1005, 833, 771 cm^{-1}.
HRMS (ESI) m/z: $[M+Na]^+$ Calcd for $C_{34}H_{74}NaO_4Si_3$ 653.47871; Found 653.47874.

Aldehyde 4 (C4-C19 fragment)

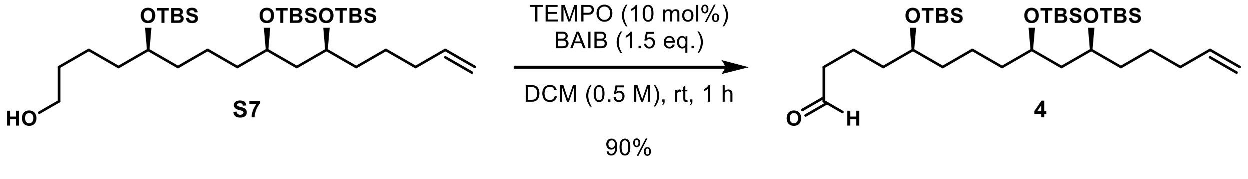

To a solution of alcohol **S7** (1.45 g, 2.30 mmol, 1.0 eq.) and iodobenzene diacetate (BAIB) (1.11 g, 3.45 mmol, 1.5 eq.) in DCM (4.6 mL, 0.5 M) was added TEMPO (36 mg, 0.23 mmol, 10 mol%) at ambient temperature under

Ar atmosphere. The reaction was stirred for 1.5 h and quenched with sat. $NaHCO_3$ (5 mL) and sat. $Na_2S_2O_3$ (5 mL) at 0 °C. The resultant was stirred vigorously at this temperature. The phases were separated and the aqueous phase was extracted with DCM (3 x 20 mL). The organic fractions were combined, washed with brine, dried over $MgSO_4$, filtered and concentrated *in vacuo*. The residue was purified by column chromatography on silica gel (Hexane/EtOAc= 100/0 to 100/1) to furnish aldehyde **4** (1.30 g, 2.07 mmol) in 90% yield as a colorless oil.

R_f = 0.72 (Hexane/EtOAc = 5/1).
$[\alpha]_D^{22}$: −0.18 (c = 1.11, $CHCl_3$).
^{1}H NMR (600 MHz, $CDCl_3$) δ 9.76 (t, *J* = 1.8 Hz, 1H), 5.80 (ddt, *J* = 17.4, 10.2, 6.6 Hz, 1H), 5.00 (ddt, *J* = 17.4, 1.8, 1.8 Hz, 1H), 4.95 (ddt, *J* = 10.2, 1.8, 1.2 Hz, 1H), 3.75-3.71 (m, 2H), 3.68-3.64 (m, 1H), 2.42 (td, *J* = 7.2, 1.8 Hz, 2H), 2.04 (dt, *J* = 6.6, 6.6 Hz, 2H), 1.73-1.59 (m, 3H), 1.52-1.23 (m, 13H), 0.88 (s, 27H), 0.040 (s, 9H), 0.036 (s, 9H).
^{13}C NMR ($CDCl_3$, 151 MHz) δ 202.6, 138.9, 114.4, 71.9, 69.53, 69.49, 44.8, 44.0, 37.6, 37.4, 36.6, 36.3, 34.0, 25.9 (9C), 24.4, 20.7, 18.10, 18.06 (2C), 17.9, -4.26, -4.31, -4.36, -4.4 (2C), -4.5.
IR (ATR): 3078, 2951, 2928, 2857, 2372, 2321, 1730, 1472, 1462, 1254, 1063, 833, 771, cm^{-1}.
HRMS (ESI) m/z: $[M+Na]^+$ Calcd for $C_{34}H_{72}NaO_4Si_3$ 651.46306; Found 651.46307.

Benzoate S8

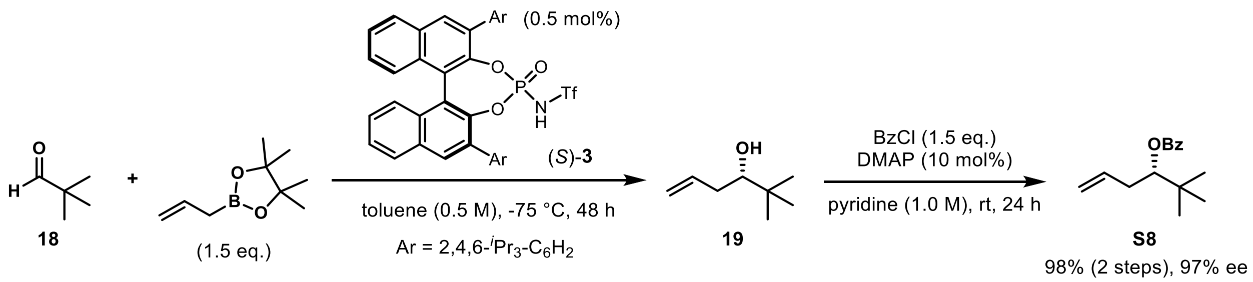

To a solution of (*S*)-**3** (44.2 mg, 0.05 mmol, 0.5 mol%) and pivalaldehyde (**18**) (990 mg, 10 mmol, 1.0 eq.) in pentane (6.0 mL) was added allylboronic acid pinacol ester (2.83 mL, 15 mmol, 1.5 eq.) in pentane (4.0 mL) at -75 °C under Ar atmosphere. The reaction was stirred for 48 h at this temperature and allowed to warm to room temperature. The resulting pink solution was added *N*-methyl-2-amino-ethanol (0.96 mL, 12 mmol, 1.2 eq.) dropwise (white precipitation was immediately formed). The resulting white suspension was stirred for additional 1 h. The reaction mixture was then filtered through a pad of celite and the celite cake was rinsed with pentane (3 x 10 mL). the filtrate was then washed with 1 N HCl (30 mL) and brine (30 mL), dried over Na_2SO_4, filtered and carefully concentrated under reduced pressure (>300 mmHg, <25 °C). The crude material was purified by flush column chromatography (pentane/Et_2O = 40:1 to 20:1) to obtain secondary alcohol **19** and (Hexane/EtOAc = 5/1 to 4/1) to recover (*S*)-**3** as a salt. **19** was directly used for the next step as a Et_2O/pentane mixture. The recovered salt of (*S*)-**3** was protonated by washing DCM solution of this salt (5 mL) with 6N HCl (5 mL) and dried under high vacuum to recover (*S*)-**3** (43.6 mg, 0.049 mmol) in 99% as a white solid. All spectroscopic data was identical to the known

compound.[4] The enantiomeric excess of **19** was determined by chiral stationary phase HPLC analysis of derivative **S28** (See page S45).

To the solution of secondary alcohol **19** in pyridine (10 mL, 1.0 M) was added BzCl (1.4 mL, 12 mmol, 1.2 eq.) and 4-dimethylamino-pyridine (DMAP) (122 mg, 1.0 mmol, 10 mol%) at room temperature. The resulting suspension was stirred for 24 h at 30 °C. The resulting mixture was poured into 1N HCl and the phases were separated. The aqueous phase was extracted with EtOAc (3 x 10 mL) and the organic layers were combined, washed with brine, dried over Na_2SO_4 and concentrated under reduced pressure. The crude product was purified by flush column chromatography (Hexane/EtOAc = 100/1) to furnish benzoate **S8** (2.31 g, 9.8 mmol) in 98% yield, 97% ee over 2 steps as a colorless oil.

Rf = 0.75 (Hexane/EtOAc = 5/1).
$[\alpha]_D^{23}$: −6.84 (c = 1.00, $CHCl_3$).
^{1}H NMR (600 MHz, $CDCl_3$) δ 8.05 (ddd, *J* = 7.8, 1.8, 1.2 Hz, 2H), 7.55 (tt, *J* = 7.8, 1.2 Hz, 1H), 7.44 (ddd, *J* = 7.8, 7.8, 1.8 Hz, 2H), 5.79 (dddd, *J* = 16.2, 10.2, 7.8, 6.0 Hz, 1H), 5.06-5.02 (m, 2H), 4.95 (ddtt, *J* = 10.2, 2.4, 1.2, 1.2 Hz, 1H), 2.49 (dddt, *J* = 14.4, 6.0, 3.0, 1.2 Hz, 1H), 2.34 (dddt, *J* = 14.4, 10.2, 7.8, 1.2 Hz, 1H),1.01 (s, 9H).
^{13}C NMR ($CDCl_3$, 151 MHz) δ 166.3, 135.1, 132.7, 130.6, 129.6 (2C), 128.3 (2C), 117.2, 80.3, 34.9, 34.7, 26.1 (3C).
IR (ATR): 2967, 1717, 1366, 1268, 1109, 914, 708 cm^{-1}.
HRMS (ESI) m/z: [M+Na]+ Calcd for $C_{15}H_{20}NaO_2$ 255.13555; Found 255.13553.

Unsaturated aldehyde S9

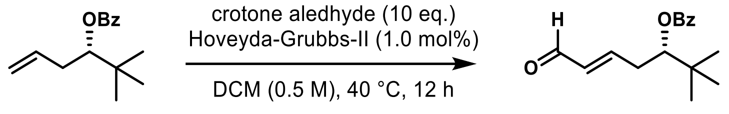

To a solution of alkene **S8** (1.82 g, 7.85 mmol) and crotonaldehyde (6.47 mL, 78.5 mmol, 10 eq.) in DCM (15.7 mL, 0.5 M) was added 2nd Generation Hoveyda-Grubbs catalyst (49.2 mg, 78.5 μmol, 1.0 mol%) under Ar atmosphere. The reaction mixture was allowed to warm to 40 ℃ by an oil bath and stirred at the same temperature for 12 h. The reaction mixture was concentrated in vacuo. The residue was purified by column chromatography on silica gel (Hexane/EtOAc= 40/1 to 30/1) to afford **S9** (1.75 g, 6.74 mmol) in 86% yield as a colorless oil.

R_f = 0.30 (Hexane/EtOAc = 5/1).
$[\alpha]_D^{25}$: −34.1 (c = 0.74, $CHCl_3$).
^{1}H NMR (600 MHz, $CDCl_3$) δ 9.43 (d, *J* = 7.8 Hz, 1H), 8.02 (ddd, *J* = 7.8, 1.8, 1.2 Hz, 2H), 7.58 (tt, *J* = 7.8, 1.2 Hz, 1H), 7.45 (ddd, *J* = 7.8, 7.8, 1.8 Hz, 2H), 6.83 (ddd, *J* = 15.6, 8.4, 6.0 Hz, 1H), 6.12 (dddd, *J* = 15.6, 7.8, 1.2, 1.2 Hz, 1H), 5.15 (dd, *J* = 10.2, 3.0 Hz, 1H), 2.76 (dddd, *J* =15.0, 6.0, 3.0, 1.2

Hz, 1H), 2.68-2.62 (dddd, *J* = 15.0, 10.2, 8.4, 1.2 Hz, 1H), 1.04 (s, 9H).

^{13}C NMR ($CDCl_3$, 151 MHz) δ 193.7, 166.2, 154.5, 134.8, 133.2, 129.9, 129.6 (2C), 128.5 (2C), 79.2, 35.1, 34.0, 26.0 (3C).

IR (ATR): 2966, 2372, 2320, 1715, 1690, 1268, 1110, 969, 709 cm^{-1}.

HRMS (ESI) m/z: $[M+Na]^+$ Calcd for $C_{16}H_{20}NaO_3$ 283.13047; Found 283.13046.

Alcohol 9

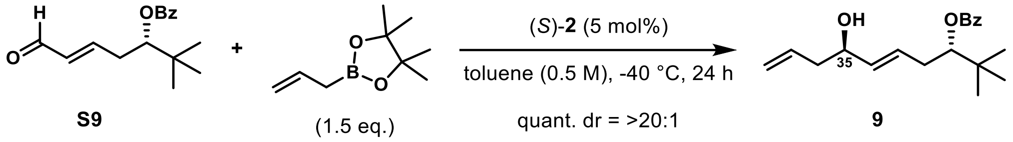

To a solution of unsaturated aldehyde **S9** (1.04 g, 4.0 mmol) and (*S*)-**2** (144 mg, 0.2 mmol, 5 mol%) in toluene (4.8 mL) was added allylboronic acid pinacol ester (1.13 mL, 6.0 mmol, 1.5 eq.) in toluene (3.2 mL) at -40 °C. The reaction mixture was stirred for 24 h at this temperature and then allowed to warm to room temperature. The resulting solution was added *N*-methyl-2-amino-ethanol (384 μL, 4.8 mmol, 1.2 eq.) dropwise (white precipitation was immediately formed). The resulting white suspension was stirred for additional 1 h and diluted with hexane (20 mL). The suspension was filtered through a pad of celite and the celite cake was rinsed with hexane (3 x 10 mL). The filtrate was washed with 1 N HCl (30 mL) then brine (30 mL), dried over Na_2SO_4, filtered and carefully concentrated under reduced pressure. The residue was purified by column chromatography on silica gel (Hexane/EtOAc= 20/1 to 10/1) to furnish alcohol **9** (1.21 g, 4.0 mmol) in quantitative yield, *dr* = >20:1 as a colorless oil. (*S*)-**2** was recovered as a salt by eluting Hexane/EtOAc = 2/1. The recovered salt was dissolved in DCM and washed by 6N HCl and dried under high-vacuum to recover (*S*)-**2** (140 mg, 0.195 mmol) in 98% yield as a white solid. The absolute configuration at C35 was determined by ^{1}H NMR analysis of MTPA ester derivative (see page S40).

R_f = 0.15 (Hexane/EtOAc = 5/1).

$[\alpha]_D^{25}$: −25.5 (c = 0.73, $CHCl_3$).

^{1}H NMR (600 MHz, $CDCl_3$) δ 8.04 (ddd, *J* = 7.8, 1.8, 1.2 Hz, 2H), 7.56 (tt, *J* = 7.8, 1.2 Hz, 1H), 7.44 (ddd, *J* = 7.8, 7.8, 1.8 Hz, 2H), 5.66 (m, 2H), 5.49 (dd, *J* = 15.6, 7.2 Hz, 1H), 5.01 (dd, *J* = 10.2, 2.4 Hz, 1H), 4.95-4.90 (m, 2H), 3.99 (td, *J* = 7.2, 5.4 Hz, 1H), 2.47 (ddd, *J* = 14.4, 6.0, 2.4 Hz, 1H), 2.30 (ddd, *J* = 14.4, 10.2, 9.0 Hz, 1H), 2.08-1.99 (m, 2H), 1.00 (s, 9H).

^{13}C NMR ($CDCl_3$, 151 MHz) δ 166.2, 135.1, 134.1, 132.8, 130.5, 129.5 (2C), 128.43, 128.36 (2C), 117.8, 80.3, 71.6, 41.5, 34.8, 33.2, 26.1 (3C).

IR (ATR): 3483, 2966, 2373, 2320, 1715, 1271, 1113, 967, 709 cm^{-1}.

HRMS (ESI) m/z: $[M+Na]^+$ Calcd for $C_{19}H_{26}NaO_3$ 325.17742; Found 325.17742.

Silyl ether S10

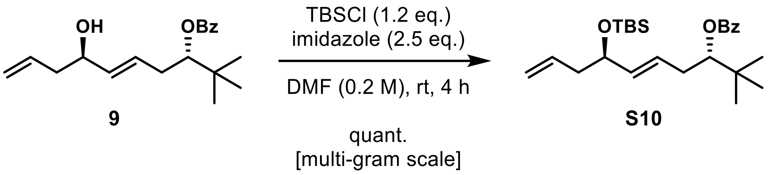

To a solution of alcohol **9** (1.81 g, 6.0 mmol) and imidazole (1.02 g, 15 mmol, 2.5 eq.) in DMF (30 mL, 0.2 M) was added TBSCl (1.08 g, 7.2 mmol, 1.2 eq.) at 0 °C. The reaction mixture was allowed to warm to room temperature and stirred for 4 h. The reaction mixture was quenched with aq. $NaHCO_3$ (20 mL) and then diluted with Et_2O (20 mL). The phases were separated and the aqueous phase was extracted with Et_2O (3 x 20 mL), the organic fractions were combined, washed with brine, dried over $MgSO_4$, filtered and concentrated *in vacuo*. The residue was purified by column chromatography on silica gel (Hexane/EtOAc= 40/1) to furnish silyl ether **S10** (2.49 g, 6.0 mmol) in quantitative yield as a colorless oil.

R_f = 0.70 (Hexane/EtOAc = 5/1).
$[\alpha]_D^{25}$: −24.5 (c = 0.67, $CHCl_3$).
^{1}H NMR (600 MHz, $CDCl_3$) δ 8.04 (ddd, *J* = 7.8, 1.8, 1.2 Hz, 2H), 7.55 (tt, *J* = 7.8, 1.2 Hz, 1H), 7.43 (ddd, *J* = 7.8, 7.8, 1.8 Hz, 2H), 5.57 (ddt, *J* = 16.8, 10.8, 7.2 Hz, 1H), 5.50 (ddd, *J* = 15.0, 8.4, 5.4 Hz, 1H), 5.41 (dd, *J* = 15.0, 6.6 Hz, 1H), 5.02 (dd, *J* = 10.2, 3.0 Hz, 1H), 4.80-4.76 (m, 2H), 3.96 (dt, *J* = 6.6, 6.0 Hz, 1H), 2.46 (ddd, *J* = 14.4, 5.4, 3.0 Hz, 1H), 2.29 (ddd, *J* = 14.4, 10.2, 8.4 Hz, 1H), 2.02-1.92 (m, 2H), 1.00 (s, 9H), 0.83 (s, 9H), -0.03 (s, 3H), -0.04 (s, 3H).
^{13}C NMR ($CDCl_3$, 151 MHz) δ 166.0, 137.0, 134.9, 132.7, 130.7, 129.6 (2C), 128.3 (2C), 126.6, 116.4, 80.1, 73.2, 42.8, 34.9, 33.2, 26.1 (3C), 25.8 (3C), 18.2, -4.4, -4.8.
IR (ATR): 2956, 2856, 2373, 2321, 1719, 1269, 1250, 1111, 1068, 834, 775, 708 cm^{-1}.
HRMS (ESI) m/z: $[M+Na]^+$ Calcd for $C_{25}H_{40}NaO_3Si$ 439.26389; Found 439.26388.

5-(naphthalen-2-ylmethoxy)pentan-1-ol

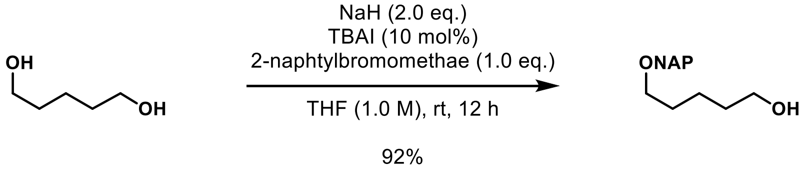

To a solution of 1,5-pentanediol (10.4 g, 100 mmol, 5 eq.) in THF (20 mL, 1.0 M) was added 60% NaH (1.6 g, 2.0 eq.) at 0 °C portion wise over 5 min under an atmosphere of nitrogen. The resulting suspension was stirred for 1 h at this temperature followed by addition of 2-naphtylbromomethane (4.42 g, 20 mmol) and tetra butyl ammonium iodide (738 mg, 2 mmol, 10 mol%). The reaction was allowed to warm to room temperature and stirred for 12 h. The reaction was then poured into sat. NH_4Cl (50 mL). The aqueous phase was extracted with EtOAc (3 x20 mL), the organic fractions were combined, washed with brine, dried over Na_2SO_4, filtered and concentrated in vacuo. The residue was purified by column chromatography on silica gel (Hexane/EtOAc= 5/1 to 2/1) to give 5-(naphthalen-2-

ylmethoxy)pentan-1-ol (4.5 g, 18.4 mmol) in 92% yield as a pale-yellow oil. All spectroscopic data for 5-(naphthalen-2-ylmethoxy)pentan-1-ol (^{1}H NMR, ^{13}C NMR, IR, and HRMS) were identical to the known compound.[5]

Aldehyde 23

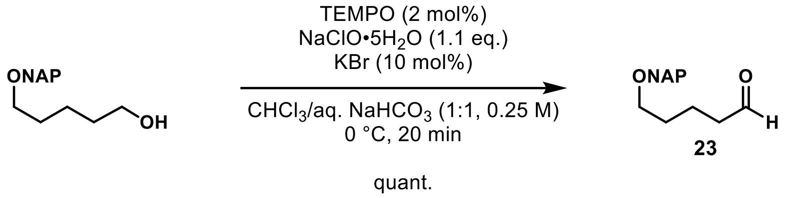

To a biphasic mixture of 5-(naphthalen-2-ylmethoxy)pentan-1-ol (2.44 g, 10 mmol), TEMPO (31.3 mg, 0.2 mmol, 2 mol%) in $CHCl_3$ (20 mL, 0.5 M) and KBr (119 mg, 1.0 mmol, 10 mol%) in sat. $NaHCO_3$ (10 mL) was added $NaClO·5H_2O$ (1.81g, 11 mmol, 1.1 eq.) in sat. $NaHCO_3$ (10 mL) dropwise over 5 min at 0 ℃ with vigorous stirring. The reaction mixture was stirred vigorously for additional 15 min at this temperature. The reaction was quenched with sat. $Na_2S_2O_3$ (10 mL) and stirred for 10 min. The phases were separated and the aqueous phase was extracted with DCM (3 x20 mL). The organic layers were combined, dried over $MgSO_4$, filtered and concentrated in *vacuo*. The crude material was purified by flash column chromatography (Hexane/EtOAc = 5/1) to furnish aldehyde **23** (2.41 g, 10 mmol) in quantitative yield as a colorless oil.

R_f = 0.24 (Hexane/EtOAc = 5/1).
^{1}H NMR (600 MHz, $CDCl_3$) δ 9.75 (t, *J* = 1.8 Hz, 1H), 7.84-7.79 (m, 3H), 7.77 (s, 1H), 7.49-7.44 (m, 3H), 4.65 (s, 2H), 3.52 (t, *J* = 6.0 Hz, 2H), 2.47-2.44 (td, *J* = 7.2, 1.8 Hz, 2H), 1.78-1.71 (m, 2H), 1.71-1.65 (m, 2H).
^{13}C NMR ($CDCl_3$, 151 MHz) δ 202.5, 135.9, 133.3, 132.9, 128.2, 127.8, 127.7, 126.3, 126.1, 125.8, 125.7, 73.0, 69.8, 43.6, 29.2, 18.9.
IR (ATR): 2940, 2863, 2722, 1720, 1123, 1092, 795 cm^{-1}.
HRMS (ESI) m/z: $[M+Na]^+$ Calcd for $C_{16}H_{18}NaO_2$ 265.11990; Found 265.11988.

Homoallyl alcohol S12

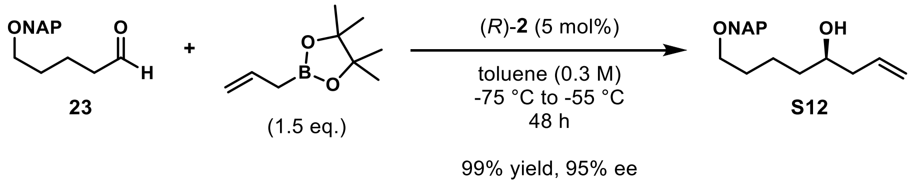

To a solution of aldehyde **23** (1.45 g, 6.0 mmol, 1.0 eq.) and (*R*)-**2** (216 mg, 0.3 mmol, 5 mol%) in toluene (12 mL) was added allylboronic acid pinacol ester (1.51 g, 9 mmol, 1.5 eq.) in toluene (8.0 mL) dropwise at -75 °C under an Ar atmosphere. The reaction mixture was stirred for 24 h at this temperature and stirred for an additional 24 h at -55 °C. *N*-methyl-2-amino-ethanol (575 μL, 7.2 mmol, 1.2 eq.) was added to the mixture. The resulting suspension was stirred for 1 h at ambient temperature. The reaction was diluted with hexane (12 mL) and filtered through a pad

of celite. The remaining white solid and celite cake was rinsed with hexane (2 x 12 mL). The filtrate was washed with 1N HCl then brine, dried over Na_2SO_4, filtered and concentrated under reduced pressure. The residue was purified by flash column chromatography on silica gel (Hexane/EtOAc = 20/1 to 4/1) to obtain homoallylic alcohol **S12** (1.69 g, 5.94 mmol) in 99% yield, 95% ee as a colorless oil. (*R*)-**2** was recovered as a salt by eluting Hexane/EtOAc = 2/1. The recovered salt was dissolved in DCM (10 mL), washed by 6N HCl (2 x 10 mL) and dried under high-vacuum to recover (*R*)-**2** (210 mg, 0.292 mmol) in 97% yield as a white solid. The ee value was determined by chiral stationary phase HPLC analysis of 24 (Chiralpack OD-3 column (Hexane/iPrOH = 98/2, 1.0 mL/min, 40 °C, 254 nm), tR major = 21.6 min, tR minor = 23.2 min; 97% ee. The absolute configuration of **S12** was determined by ^{1}H NMR analysis of MTPA ester derivative (see page S38).

R_f = 0.14 (Hexane/EtOAc = 5/1).
$[\alpha]_D^{23}$: +6.13 (c = 1.00, $CHCl_3$).
^{1}H NMR (600 MHz, $CDCl_3$) δ 7.83-7.82 (m, 3H), 7.77 (s, 1H), 7.49-7.44 (m, 3H), 5.85-5.78 (m, 1H), 5.14-5.11 (m, 2H), 4.66 (s, 2H), 3.66-3.62 (m, 1H), 3.52 (t, *J* = 6.6 Hz, 2H), 2.31-2.27 (dddt, *J* =13.8, 6.6, 4.2, 1.2 Hz, 1H), 2.15-2.10 (m, 1H), 1.72-1.64 (m, 2H), 1.63 (brs, 1H), 1.58-1.41 (m, 4H).
^{13}C NMR ($CDCl_3$, 151 MHz) δ 136.1, 134.8, 133.3, 132.9, 128.1, 127.8, 127.7, 126.3, 126.0, 125.8 (2C), 118.1, 73.0, 70.5, 70.3, 41.9, 36.5, 29.7, 22.4.
IR (ATR): 3421, 3056, 2931, 2859, 2373, 2321, 1639, 1123, 1093, 911, 816 cm^{-1}.
HRMS (ESI) m/z: $[M+Na]^+$ Calcd for $C_{19}H_{24}NaO_2$ 307.16685; Found 307.16684.

Silyl ether 24

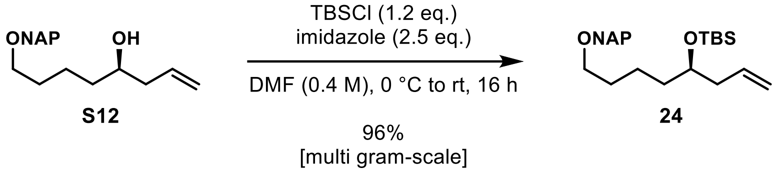

To alcohol **S12** (2.13 g, 7.5 mmol) and imidazole (1.28 g, 18.8 mmol, 2.5 eq.) in DMF (18.8 mL, 0.4 M) was added TBSCl (1.36 g, 9.0 mmol, 1.2 eq.) at 0 °C under an atmosphere of nitrogen and the solution was stirred at room temperature for 16 h. The reaction mixture was diluted with Et_2O and then quenched with aq. $NaHCO_3$. The aqueous phase was extracted with Et_2O (3 x 20 mL), the organic fractions were combined, washed with H_2O then brine, dried over $MgSO_4$, filtered and concentrated *in vacuo*. The resultant residue was purified by column chromatography on silica gel (Hexane/EtOAc = 30/1) to give **24** (2.87 g, 7.2 mmol) in 96% yield as a colorless oil.

R_f = 0.66 (Hexane/EtOAc = 5/1).
$[\alpha]_D^{25}$: +9.34 (c = 0.83, $CHCl_3$).
^{1}H NMR (600 MHz, $CDCl_3$) δ 7.87-7.83 (m, 3H), 7.78 (s, 1H), 7.49-7.45 (m, 3H), 5.81 (ddt, *J* =14.4, 10.2, 7.2 Hz, 1H), 5.04-5.00 (m, 2H), 4.66 (s, 2H), 3.70-3.67 (m, 1H), 3.51 (t, *J* = 6.6 Hz, 2H), 2.25-2.17 (m, 2H), 1.67-1.62 (m, 2H), 1.51-1.35 (m, 4H), 0.89 (s, 9H), 0.051 (s, 3H), 0.046 (s, 3H).

^{13}C NMR ($CDCl_3$, 151 MHz) δ 136.2, 135.4, 133.3, 132.9, 128.1, 127.8, 127.7, 126.2, 126.0, 125.7 (2C), 116.6, 73.0, 71.9, 70.4, 41.9, 36.6, 29.9, 25.9 (3C), 22.0, 18.1, -4.4, -4.5.
IR (ATR): 3058, 2855, 2373, 2320, 1471, 1254, 1123, 1094, 834, 773 cm^{-1}.
HRMS (ESI) m/z: $[M+Na]^+$ Calcd for $C_{25}H_{38}NaO_2Si$ 421.25333; Found 421.25333.

Boronic ester 25

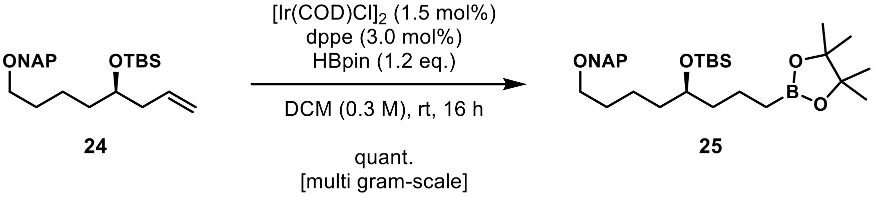

To a solution of silyl ether **24** (2.0 g, 5.0 mmol, 1.0 eq.), $[Ir(COD)Cl]_2$ (50 mg, 0.075 mmol, 1.5 mol%), and 1,2-bis(diphenylphosphino)ethane (60 mg, 0.015 mmol, 3.0 mol%) in DCM (16.7 mL) was added 4,4,5,5-tetramethyl-1,3,2-dioxaborolane (862 µL, 6.0 mmol, 1.2 eq.) dropwise under Ar atmosphere at ambient temperature. The reaction mixture was stirred for 16 h and quenched with methanol (5.0 mL). The resulting mixture was poured in to sat. $NaHCO_3$ and the phases were separated. The aqueous phase was extracted with Et_2O (3 x 20 mL), the organic fractions were combined, washed with brine, dried over $MgSO_4$, filtered and concentrated *in vacuo*. The resultant residue was purified by flash column chromatography on silica gel (Hexane/EtOAc = 20/1) to give **25** (2.28 g, 5.0 mmol) in quantitative yield as a colorless oil.

R_f = 0.56 (Hexane/EtOAc = 5/1).
$[\alpha]_D^{25}$: +3.06 (c = 1.26, $CHCl_3$).
^1H NMR (600 MHz, $CDCl_3$) δ 7.83-7.81 (m, 3H), 7.77 (s, 1H), 7.48-7.44 (m, 3H), 4.66 (s, 2H), 3.64-3.62 (m, 1H), 3.50 (t, *J* = 6.6 Hz, 2H), 1.67-1.60 (m, 2H), 1.46-1.36 (m, 8H), 1.23 (s, 12H), 0.87 (s, 9H), 0.80-0.73 (m, 2H), 0.03 (s, 3H), 0.02 (s, 3H).
^{13}C NMR ($CDCl_3$, 151 MHz) δ 136.2, 133.3, 132.9, 128.1, 127.8, 127.7, 126.2, 126.0, 125.8, 125.7, 82.9 (2C), 73.0, 72.1, 70.5, 39.9, 36.8, 30.0, 26.0 (3C), 24.8 (4C), 22.0, 19.8, 18.2, 11.2, -4.40, -4.44.
IR (ATR): 3057, 2929, 2978, 2373, 2321, 1461, 1371, 1145, 1097, 835, 795, 754 cm^{-1}.
HRMS (ESI) m/z: $[M+Na]^+$ Calcd for $C_{31}H_{51}NaBO_4Si$ 549.35419; Found 549.35420.

Vinyl ketone 8

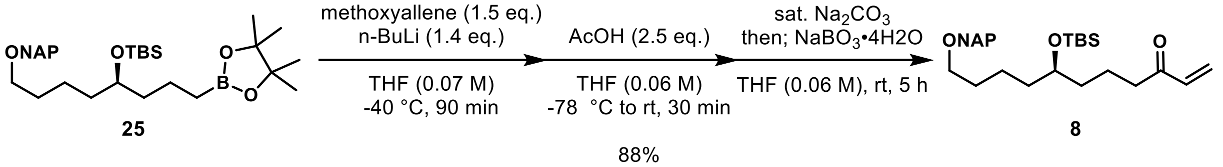

This C3 homologation reaction was performed following literature procedure.[6]

To a solution of methoxy allene (0.633 mL, 7.5 mmol, 1.5 eq.) in THF (40 mL) was added *n*-BuLi (1.6 M in Hexane, 4.52 mL, 1.4 eq.) dropwise at -40 °C. The reaction mixture was stirred for 30 min at this temperature. Then boronic ester **25** (2.28 g, 5.0 mmol, 1.0 eq.) was slowly added as a THF solution (10 mL). The reaction was stirred for an additional 90 min before further cooled to -78 °C. Freshly distilled acetic acid (0.71 mL, 12.5 mmol, 2.5 eq.) in THF (5.0 mL) was carefully added and the reaction was stirred for 5 min at this temperature. An acetone-dry ice bath was removed, and the reaction was stirred for 1 h at room temperature. Sat. Na_2CO_3 (12 mL) and sodium perborate tetrahydrate (1.54 g, 10 mmol, 2.0 eq.) were added sequentially to the reaction mixture to oxidize allyl boronic ester intermediate. The reaction mixture was stirred for 3 h at room temperature. The resulting mixture was diluted with Et_2O (30 mL) and H_2O (30 mL). The phases were separated and the aqueous phase was extracted with Et_2O (3 x 20 mL). The organic fractions were combined, washed with brine, dried over $MgSO_4$, filtered and concentrated in vacuo. The residue was purified by flush column chromatography on silica gel (Hexane/EtOAc = 20:1) to afford vinyl ketone **8** (1.99 g, 4.38 mmol) in 88% yield as a colorless oil.

R_f = 0.44 (Hexane/EtOAc = 5/1).
$[\alpha]_D^{26}$: −2.52 (c = 0.56, $CHCl_3$).
^1H NMR (600 MHz, $CDCl_3$) δ 7.83-7.81 (m, 3H), 7.78 (s, 1H), 7.49-7.43 (m, 3H), 6.34 (dd, *J* = 18.0, 10.8 Hz, 1H), 6.20 (dd, *J* = 18.0, 1.2 Hz, 1H), 6.34 (dd, *J* = 10.8, 1.2 Hz, 1H), 4.66 (s, 2H), 3.67-3.64 (m, 1H), 3.51 (t, *J* = 6.6 Hz, 2H), 2.61-2.52 (m, 2H), 1.71-1.57 (m, 4H), 1.47-1.32 (m, 6H), 0.87 (s, 9H), 0.03 (s, 6H).
^{13}C NMR ($CDCl_3$, 151 MHz) δ 200.8, 136.5, 136.2, 133.3, 132.9, 128.1, 127.9, 127.8, 127.7, 126.3, 126.0, 125.8, 125.7, 73.0, 72.0, 70.4, 39.8, 36.8, 36.4, 30.0, 25.9 (3C), 22.0, 19.8, 18.1, -4.41, -4.43.
IR (ATR): 3056, 2972, 2855, 2372, 2321, 1703, 1682, 1254, 1092, 835, 773 cm^{-1}.
HRMS (ESI) m/z: $[M+Na]^+$ Calcd for $C_{28}H_{42}NaO_3Si$ 477.27954; Found 477.27956.

Enone 20

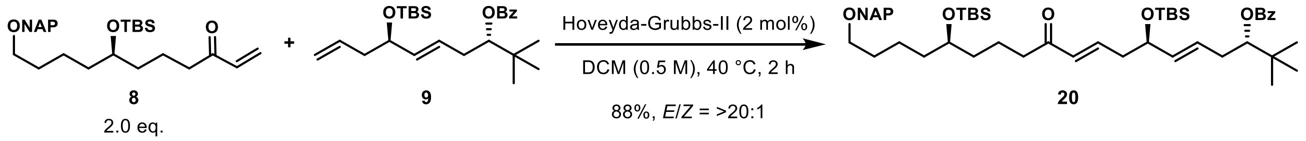

To a solution of alkene **9** (1.67 g, 4.0 mmol, 1.0 eq.) and vinyl ketone **8** (3.67 g, 8.0 mmol, 2.0 eq.) in 1,2-DCE (8.0 mL, 0.5 M) was added 2nd Generation Hoveyda-Grubbs catalyst (50.1 mg, 0.08 mmol, 2.0 mol%) under Ar atmosphere at 40 °C. The reaction mixture was stirred for 2 h at this temperature and concentrated in *vacuo*. The residue was purified by flash column chromatography on silica gel (Hexane/EtOAc = 25:1) to give enone **20** (2.96 g, 3.52 mmol) in 88% yield as a colorless oil. Unreacted vinyl ketone **8** was eluted with (Hexane/EtOAc = 30:1) to afford pure **8** (1.36 g, 3.0 mmol) in 75% yield as a yellow oil.

R_f = 0.50 (Hexane/EtOAc = 5/1).
$[\alpha]_D^{26}$: −13.8 (c = 0.67, $CHCl_3$).
^1H NMR (600 MHz, $CDCl_3$) δ 8.02 (ddd, *J* = 7.8, 1.8, 1.2 Hz, 2H), 7.83-7.81 (m, 3H), 7.77 (s, 1H), 7.54 (tt, *J* = 7.8,

1.2 Hz, 1H), 7.83-7.42 (m, 5H), 6.65 (dt, J = 16.2, 7.2 Hz, 1H), 5.85 (dt, J = 16.2, 1.2 Hz, 1H), 5.54 (ddd, J = 15.0, 8.4, 6.0 Hz, 1H), 5.41 (dd, J = 15.0, 6.6 Hz, 1H), 4.99 (dd, J = 10.2, 2.4 Hz, 1H), 4.66 (s, 2H), 4.06 (dt, J = 6.0, 6.0 Hz, 1H), 3.66-3.62 (m, 1H), 3.50 (t, J = 6.6 Hz, 2H), 2.48-2.42 (m, 1H), 2.44 (t, J = 7.2 Hz, 2H), 2.27 (ddd, J = 14.4, 10.2, 8.4 Hz, 1H), 2.08-2.01 (m, 2H), 1.65-1.52 (m, 4H), 1.47-1.32 (m, 6H), 1.00 (s, 9H), 0.87 (s, 9H), 0.81 (s, 9H), 0.03 (s, 6H), -0.05 (s, 3H), -0.06 (s, 3H).

^{13}C NMR ($CDCl_3$, 151 MHz) δ 200.4, 166.0, 143.5, 136.2, 135.5, 133.3, 132.9, 132.8, 132.3, 130.6, 129.5 (2C), 128.4 (2C), 128.1, 127.9, 127.7, 127.1, 126.3, 126.0, 125.8, 125.7, 80.0, 73.0, 72.3, 72.0, 70.4, 41.3, 39.7, 36.8, 36.6, 34.8, 33.2, 30.0, 26.1 (3C), 25.9 (3C), 25.8 (3C), 22.0, 20.0, 18.12, 18.09, -4.3, -4.4 (2C), -4.9.

IR (ATR): 3055, 2954, 2928, 2856, 1718, 1673, 1631, 1471, 1366, 1271, 1252, 1095, 889, 835, 774 cm^{-1}.

HRMS (ESI) m/z: $[M+Na]^+$ Calcd for $C_{51}H_{78}NaO_6Si_2$ 865.52291; Found 865.52290.

Allyl alcohol S13

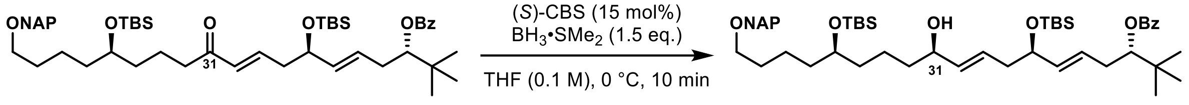

20 S13

To a solution of (*S*)-CBS catalyst (116 mg, 0.42 mmol, 15 mol%) in THF (8.4 mL) was added $BH_3·SMe_2$ (ca. 10 M in DMS, 0.42 mL, 4.2 mmol, 1.5 eq.) dropwise at 0 °C under Ar atmosphere. The reaction mixture was stirred for 30 min at this temperature before added enone **20** (2.36 g, 2.8 mmol, 1.0 eq.) in THF (19.6 mL) dropwise. The reaction was stirred for additional 10 minutes and quenched with MeOH (5.0 mL). The resultant was transferred to a separatory funnel and washed with sat. NH_4Cl (30 mL). The aqueous phase was extracted with EtOAc (3 x 20 mL). The organic fractions were combined, washed with brine, dried over Na_2SO_4, filtered and concentrated *in vacuo*. The residue was purified by column chromatography on silica gel (Hexane/EtOAc= 20/1 to 10/1) to furnish alcohol **S13** (2.36 g, 2.8 mmol) in quantitative yield, *dr* = 15:1 at C31 as a colorless oil. The relative configuration at C31 was determined by ^{1}H NMR analysis of MTPA ester derivative (see page S39).

R_f = 0.32 (Hexane/EtOAc = 5/1).

$[\alpha]_D^{26}$: −21.8 (c = 0.81, $CHCl_3$).

^{1}H NMR (600 MHz, $CDCl_3$) δ 8.03 (ddd, J = 7.8, 1.8, 1.2 Hz, 2H), 7.83-7.81 (m, 3H), 7.78 (s, 1H), 7.54 (tt, J = 7.8, 1.2 Hz, 1H), 7.48-7.42 (m, 5H), 5.49 (ddd, J = 16.2, 7.2, 6.0 Hz, 1H), 5.39 (dd, J = 15.6, 6.6 Hz, 1H), 5.38-5.33 (m, 1H), 5.29 (dd, J = 15.6, 6.6 Hz, 1H), 4.99 (dd, J = 10.8, 2.4 Hz, 1H), 4.66 (s, 2H), 4.00 (td, J = 6.6, 6.0 Hz, 1H), 3.88-3.85 (m, 1H), 3.65-3.61 (m, 1H), 3.51 (t, J = 6.6 Hz, 2H), 2.45-2.42 (m, 1H), 2.28 (ddd, J = 14.4, 10.8, 8.4 Hz, 1H), 2.02-1.92 (m, 2H), 1.67-1.57 (m, 2H), 1.49-1.22 (m, 10H), 0.99 (s, 9H), 0.87 (s, 9H), 0.80 (s, 9H), 0.024 (s, 3H), 0.022 (s, 3H), -0.04 (s, 3H), -0.06 (s, 3H).

^{13}C NMR ($CDCl_3$, 151 MHz) δ 166.2, 136.2, 135.8, 135.4, 133.3, 132.9, 132.8, 130.6, 129.6 (2C), 128.3 (2C), 128.1, 127.9, 127.7, 127.6, 126.5, 126.4, 126.3, 126.0, 125.8, 125.7, 80.5, 73.0, 72.9, 72.2, 72.1, 70.5, 40.6, 37.2, 37.1, 36.9, 34.9, 33.1, 30.0, 26.1 (3C), 25.9 (3C), 25.8 (3C), 22.0, 21.3, 18.1 (2C), -4.37, -4.40, -4.43, -4.82.

IR (ATR): 3491, 2953, 2927, 2856, 1718, 1471, 1366, 1272, 1252, 1092, 967, 835, 755 cm^{-1}.

HRMS (ESI) m/z: [M+Na]+ Calcd for $C_{51}H_{80}NaO_6Si_2$ 867.53856; Found 867.53858.

Silyl ether S14

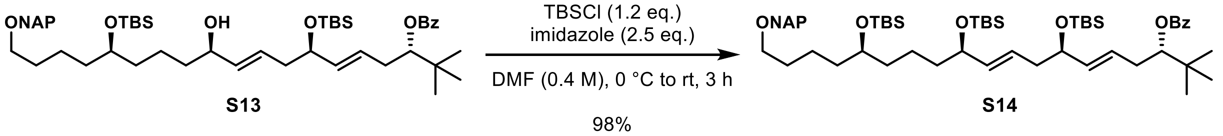

To a solution of alcohol **S13** (2.11 g, 2.5 mmol) and imidazole (426 mg, 6.25 mmol, 2.5 eq.) in DMF (6.25 mL, 0.4 M) was added TBSCl (452 mg, 3.0 mmol, 1.2 eq.) at 0 °C. The reaction mixture was allowed to warm to room temperature and stirred for 3 h. The reaction mixture was quenched with sat. $NaHCO_3$ (20 mL) and then diluted with Et_2O (20 mL). The phases were separated and the aqueous phase was extracted with Et_2O (3 x 20 mL). The organic fractions were combined, washed with brine, dried over $MgSO_4$, filtered and concentrated *in vacuo*. The residue was purified by column chromatography on silica gel (Hexane/EtOAc= 40/1) to furnish silyl ether **S14** (2.35 g, 2.45 mmol) in 98% yield as a pale yellow oil.

R_f = 0.68 (Hexane/EtOAc = 5/1).
$[\alpha]_D^{25}$: −16.4 (c = 0.60, $CHCl_3$).
^{1}H NMR (600 MHz, $CDCl_3$) δ 8.03 (ddd, *J* = 7.8, 1.8, 1.2 Hz, 2H), 7.83-7.81 (m, 3H), 7.78 (s, 1H), 7.53 (tt, *J* = 7.8, 1.2 Hz, 1H), 7.49-7.41 (m, 5H), 5.48 (ddd, *J* = 16.2, 7.2, 6.0 Hz, 1H), 5.42 (dd, *J* = 15.6, 6.6 Hz, 1H), 5.36 (dt, *J* = 15.6, 7.2 Hz, 1H), 5.19 (dd, *J* = 15.6, 6.6 Hz, 1H), 5.00 (dd, *J* = 10.2, 3.0 Hz, 1H), 4.66 (s, 2H), 3.95 (td, *J* = 6.6, 6.0 Hz, 1H), 3.91 (td, *J* = 6.6, 6.0 Hz, 1H), 3.63-3.59 (m, 1H), 3.50 (t, *J* = 6.6 Hz, 2H), 2.45-2.42 (m, 1H), 2.27 (ddd, *J* = 14.4, 10.2, 8.4 Hz, 1H), 1.92 (dd, *J* = 6.6, 6.6 Hz, 2H), 1.67-1.56 (m, 2H), 1.45-1.17 (m, 10H), 0.99 (s, 9H), 0.87 (s, 9H), 0.86 (s, 9H), 0.82 (s, 9H), 0.022 (s, 3H), 0.020 (s, 3H), 0.01 (s, 3H), -0.02 (s, 3H), -0.05 (s, 3H), -0.06 (s, 3H).
^{13}C NMR ($CDCl_3$, 151 MHz) δ 166.2, 136.2, 135.8, 135.4, 133.3, 132.9, 132.8, 130.6, 129.6 (2C), 128.3 (2C), 128.1, 127.9, 127.7, 127.4, 126.5, 126.4, 126.3, 126.0, 125.8, 125.7, 80.5, 73.0, 72.9 (2C), 72.2, 72.1, 70.5, 40.6, 37.2, 37.0, 36.9, 34.9, 33.1, 30.0, 26.1 (3C), 25.9 (6C), 25.8 (3C), 22.0, 21.2, 18.1, -4.37 (2C), -4.40 (2C), -4.43, -4.82.
IR (ATR): 2954, 2927, 2856, 1720, 1471, 1462, 1366, 1271, 1091, 967, 888, 843, 773 cm^{-1}.
HRMS (ESI) m/z: [M+Na]+ Calcd for $C_{57}H_{94}NaO_6Si_3$ 981.62504; Found 981.62509.

Primary alcohol 21

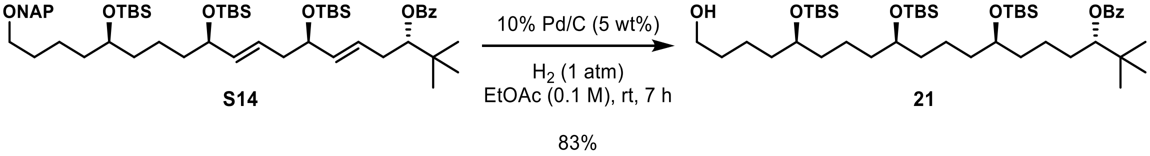

To a solution of silyl ether **S14** (2.02 g, 2.1 mmol, 1.0 eq.) in EtOAc (21 mL, 0.1 M) was added 10% Pd/C (101 mg, 5 wt.%) under an atmosphere of nitrogen. The reaction vessel was parged with H_2 three times and stirred vigorously for 7 h. The flask was then parged with Ar three times and the reaction mixture was filtered through a pad

of celite. The filtrate was concentrated under reduced pressure. The crude product was purified by flash column chromatography (Hexane/EtOAc = 30/1 to 10/1) to obtain primary alcohol **21** (1.44 g, 1.75 mmol) in 83% yield as a colorless oil.

R_f = 0.34 (Hexane/EtOAc = 5/1).
$[\alpha]_D^{19}$: −10.8 (c = 0.33, $CHCl_3$).
^{1}H NMR (600 MHz, $CDCl_3$) δ 8.06 (ddd, *J* = 7.8, 1.8, 1.2 Hz, 2H), 7.55 (tt, *J* = 7.8, 1.2 Hz, 1H), 7.44 (ddd, *J* = 7.8, 7.8, 1.8 Hz, 2H), 4.99 (dd, *J* = 8.4, 4.8 Hz, 1H), 3.65-3.61 (m, 3H), 3.60-3.54 (m, 2H), 1.63-1.18 (m, 24H), 0.97 (s, 9H), 0.88 (s, 9H), 0.87 (s, 9H), 0.80 (s, 9H), 0.031 (s, 3H), 0.029 (s, 3H), 0.013 (s, 3H), 0.008 (s, 3H), -0.022 (s, 3H), -0.036 (s, 3H).
^{13}C NMR ($CDCl_3$, 151 MHz) δ 166.4, 132.7, 130.7, 129.6 (2C), 128.3 (2C), 81.3, 72.2, 72.1, 72.0, 63.0, 37.33 (2C), 37.29, 36.99, 36.70, 34.9, 33.0, 30.0 26.1 (3C), 26.0 (6C), 25.9 (3C), 22.1, 21.4, 21.1, 21.0, 18.1 (2C), 18.0, -4.37 (2C), -4.40, -4.42 (2C), -4.50.
IR (ATR): 3412, 2952, 2928, 2857, 1719, 1471, 1366, 1272, 1253, 1090, 834, 772 cm^{-1}.
HRMS (ESI) m/z: $[M+Na]^+$ Calcd for $C_{46}H_{90}NaO_6Si_3$ 845.59374; Found 845.59371.

Aldehyde 22

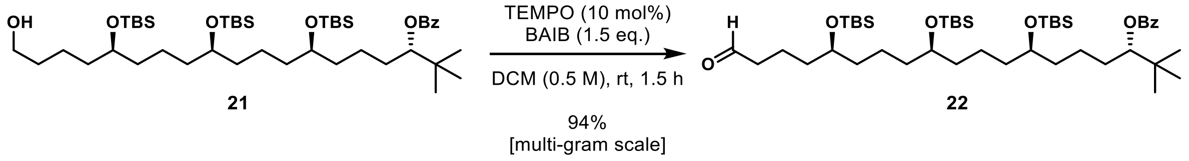

To a solution of alcohol 00 (2.14 g, 2.6 mmol, 1.0 eq.) and iodobenzene diacetate (1.26 g, 3.9 mmol, 1.5 eq.) in DCM (5.2 mL, 0.5 M) was added TEMPO (40.6 mg, 0.26 mmol, 10 mol%) at ambient temperature under Ar atmosphere. The reaction was stirred for 1.5 h and quenched with sat. $NaHCO_3$ (5 mL) and sat. $Na_2S_2O_3$ (5 mL) at 0 °C. The resultant was stirred vigorously at this temperature. The phases were separated and the aqueous phase was extracted with DCM (3 x 20 mL). The organic fractions were combined, washed with brine, dried over $MgSO_4$, filtered and concentrated *in vacuo*. The residue was purified by column chromatography on silica gel (Hexane/EtOAc= 100/1 to 50/1) to furnish aldehyde **00** (2.40 g, 2.93 mmol) in 94% yield as a colorless oil.

R_f = 0.64 (Hexane/EtOAc = 5/1).
$[\alpha]_D^{20}$: −7.63 (c = 1.26, $CHCl_3$).
^{1}H NMR (600 MHz, $CDCl_3$) δ 9.76 (t, *J* = 1.8 Hz, 1H), 8.06 (ddd, *J* = 7.8, 1.8, 1.2 Hz, 2H), 7.56 (tt, *J* = 7.8, 1.2 Hz, 1H), 7.44 (ddd, *J* = 7.8, 7.8, 1.8 Hz, 2H), 4.99 (dd, *J* = 8.4, 4.8 Hz, 1H), 3.66-3.63 (m, 1H), 3.59-3.55 (m, 2H), 2.42 (t, *J* = 1.8 Hz, 1H), 1.73-1.16 (m, 22H), 0.97 (s, 9H), 0.88 (s, 9H), 0.87 (s, 9H), 0.80 (s, 9H), 0.03 (s, 6H), 0.011 (s, 3H), 0.007 (s, 3H), -0.02 (s, 3H), -0.04 (s, 3H).
^{13}C NMR ($CDCl_3$, 151 MHz) δ 202.7, 166.4, 132.7, 130.7, 129.6 (2C), 128.3 (2C), 81.3, 72.22, 71.99, 71.8, 44.0, 37.33, 37.28 (2C), 37.24, 37.0, 36.3, 34.9, 30.0 26.1 (3C), 25.93 (3C), 25.90 (3C), 25.86 (3C), 22.1,

21.1, 21.0, 18.11, 18.09, 18.0, 17.9, -4.37 (2C), -4.41, -4.43, -4.50 (2C).

IR (ATR): 2953, 2928, 2857, 1719, 1471, 1462, 1366, 1271, 1254, 1096, 930, 834, 772, 757 cm^{-1}.

HRMS (ESI) m/z: $[M+Na]^+$ Calcd for $C_{46}H_{88}NaO_6Si_3$ 843.57809; Found 843.57806.

Secondary Alcohol S15

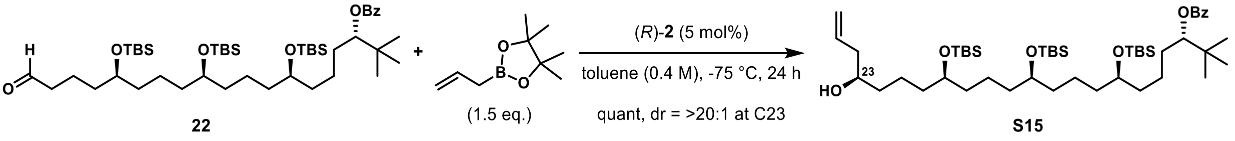

To a solution of aldehyde **22** (1.95 g, 2.40 mmol, 1.0 eq.) and (*R*)-**2** (87.4 mg, 0.12 mmol, 5 mol%) in toluene (4.0 mL) was added allylboronicacid pinacolester (605 mg, 3.6 mmol, 1.5 eq.) in toluene (2.0 mL) dropwise at -75 °C under an Ar atmosphere. The reaction mixture was stirred for 24 h at this temperature and allowed to warm to 0 °C. *N*-methyl-2-amino-ethanol (230 μL, 2.88 mmol, 1.2 eq.) was added to the mixture. The resulting suspension was stirred for 1 h at ambient temperature. The reaction was diluted with hexane (24 mL) and filtered through a pad of celite. The remaining white solid and celite cake was rinsed with hexane (2 x 24 mL). The filtrate was washed with 1N HCl and brine, dried over Na_2SO_4, filtered and concentrated under reduced pressure. The residue was purified by flash column chromatography on silica gel (Hexane/EtOAc = 20/1 to 5/1) to obtain homoallylic alcohol **S15** (2.07 g, 2.40 mmol) in quantitative yield, dr = >20:1 at C23 as a colorless oil. (*R*)-**2** was recovered as a salt by eluting Hexane/EtOAc = 2/1. The recovered salt was dissolved in DCM and washed by 6N HCl and dried under high-vacuum to recover (*R*)-**2** (84.5 mg, 0.118 mmol) in 98% yield as a white solid. The absolute configuration at C23 was determined by ^1H NMR analysis of MTPA ester derivative (see page S37).

R_f = 0.53 (Hexane/EtOAc = 5/1).

$[\alpha]_D^{20}$: −9.73 (c = 0.59, $CHCl_3$).

^1H NMR (600 MHz, $CDCl_3$) δ 8.06 (ddd, *J* = 7.8, 1.8, 1.2 Hz, 2H), 7.55 (tt, *J* = 7.8, 1.2 Hz, 1H), 7.44 (ddd, *J* = 7.8, 7.8, 1.8 Hz, 2H), 5.87-5.80 (m, 1H), 5.16-5.12 (m, 2H), 4.99 (dd, *J* = 8.4, 4.8 Hz, 1H), 3.66-3.61 (m, 2H), 3.60-3.55 (m, 2H), 2.30 (dddt, *J* = 13.8, 6.0, 5.4, 1.2 Hz, 1H), 2.14 (ddd, *J* = 13.8, 7.2, 7.2 Hz, 1H), 1.65-1.16 (m, 24H), 0.97 (s, 9H), 0.88 (s, 9H), 0.87 (s, 9H), 0.80 (s, 9H), 0.034 (s, 3H), 0.030 (s, 3H), 0.013 (s, 3H), 0.008 (s, 3H), -0.02 (s, 3H), -0.04 (s, 3H).

^{13}C NMR ($CDCl_3$, 151 MHz) δ 166.4, 134.9, 132.7, 130.7, 129.6 (2C), 128.3 (2C), 118.1, 81.3, 72.3, 72.2, 72.0, 70.6, 41.9, 37.38, 37.34, 37.30 (2C), 37.03, 37.00 (2C), 34.9, 30.0, 26.1 (3C), 26.0 (6C), 25.9 (3C), 22.1, 21.4, 21.1, 21.0, 18.1 (2C), 18.0, -4.36, -4.40 (3C), -4.43, -4.5.

IR (ATR): 3430, 2953, 2927, 2857, 1719, 1471, 1366, 1271, 1254, 1090, 928, 911, 888, 834, 772 cm^{-1}.

HRMS (ESI) m/z: $[M+Na]^+$ Calcd for $C_{49}H_{94}NaO_6Si_3$ 885.62504; Found 885.62502.

Alkene S16

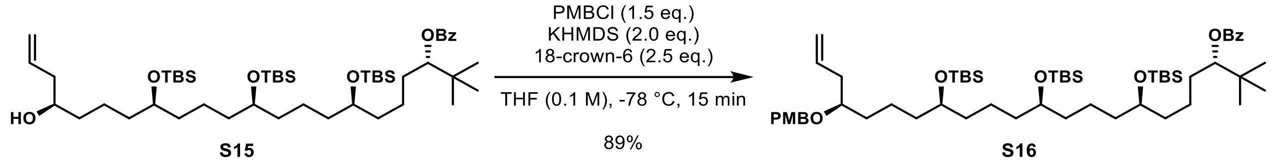

To a solution of potassium bis(trimethylsilyl) amide (4.8 mmol, 2.0 eq.) in THF/toluene (1:1, 19.2 mL, 0.25 M) was added secondary alcohol **S15** (2.07 g, 2.40 mmol, 1.0 eq.) in THF (4.8 mL) at -78 °C under Ar atmosphere. Next, *p*-methoxy benzyl chloride (564 mg, 3.6 mmol, 1.5 eq.) in THF (4.8 mL) was added and the reaction was stirred for additional 5 min. Then, 18-crown-6-ether (1.59 g, 6.0 mmol, 2.5 eq.) in THF (4.8 mL) was added to the reaction mixture. The solution was stirred for 15 min at -78 °C and quenched with sat. NH_4Cl (30 mL). The phases were separated and the aqueous phase was extracted with EtOAc (3 x 30 mL). The organic fractions were combined, washed with brine, dried over Na_2SO_4, filtered and concentrated *in vacuo*. The crude material was purified by flash column chromatography on silica gel (hexane/EtOAc =75/1 to 50/1) to obtain PMB ether **S16** (2.10 g, 2.14 mmol) in 89% yield as a colorless oil.

R_f = 0.67 (Hexane/EtOAc = 5/1).
$[\alpha]_D^{20}$: −4.49 (c = 0.38, $CHCl_3$).
^{1}H NMR (600 MHz, $CDCl_3$) δ 8.06 (ddd, *J* = 7.8, 1.8, 1.2 Hz, 2H), 7.55 (tt, *J* = 7.8, 1.2 Hz, 1H), 7.44 (ddd, *J* = 7.8, 7.8, 1.8 Hz, 2H), 7.26 (dt, *J* = 9.0, 2.4 Hz, 2H), 6.86 (dt, *J* = 9.0, 2.4 Hz, 2H), 5.84 (ddt, *J* = 16.8, 10.2, 7.2, Hz, 1H), 5.09-5.04 (m, 2H), 4.99 (dd, *J* = 8.4, 4.8 Hz, 1H), 4.49 (d, *J* = 10.8 Hz, 1H), 4.42 (d, *J* = 10.8 Hz, 1H), 3.80 (s, 3H), 3.62-3.55 (m, 3H), 3.42-3.39 (m, 1H), 2.35-2.27 (m, 2H), 1.65-1.16 (m, 24H), 0.97 (s, 9H), 0.88 (s, 9H), 0.87 (s, 9H), 0.80 (s, 9H), 0.03 (s, 6H), 0.011 (s, 3H), 0.006 (s, 3H), -0.03 (s, 3H), -0.04 (s, 3H).
^{13}C NMR ($CDCl_3$, 151 MHz) δ 166.4, 159.0, 135.1, 132.6, 131.0, 130.7, 129.6 (2C), 129.3 (2C), 128.3 (2C), 116.8, 113.7 (2C), 81.3, 78.2, 72.30, 72.27, 72.0, 70.6, 55.3, 38.3, 37.5, 37.4 (2C), 37.3, 37.2, 37.0, 34.9, 34.2, 30.0, 26.1 (3C), 26.0 (6C), 25.9 (3C), 22.1, 21.4, 21.12, 21.07, 18.1 (2C), 18.0, -4.35, -4.38 (3C), -4.4, -4.5.
IR (ATR): 2952, 2928, 2856, 1719, 1513, 1471, 1462, 1271, 1249, 1089, 1039, 834, 772, 709 cm^{-1}.
HRMS (ESI) m/z: $[M+Na]^+$ Calcd for $C_{57}H_{102}NaO_7Si_3$ 1005.6826; Found 1005.6825.

Methyl ketone 5 (C20-C43 fragment)

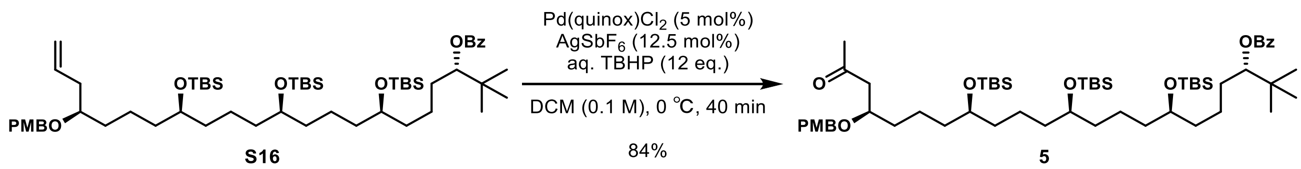

In a dry box, $AgSbF_6$ (73.9 mg, 0.215 mmol, 12.5 mol%), Pd(quinox)Cl_2 complex (32.3 mg, 0.086 mmol, 5 mol%), and a magnetic stir bar were added to a round-bottomed flask. The reaction vessel was then capped with rubber septum and taken out from a dry box. DCM (2.75 mL) was added to the reaction vessel via syringe and the mixture was stirred for 15 min at room temperature. The mixture was then diluted with DCM (2.9 mL) and aq. TBHP (70 wt.%, 2.89 mL, 20.6 mmol, 12 eq.) was added. The resulting mixture was stirred for an additional 10 min and cooled

to 0 °C. Alkene **S16** (1.69 g, 1.72 mmol, 1.0 eq.) in DCM (8.7 mL) was added to the solution dropwise. The reaction mixture was stirred for 40 min at 0 °C and quenched with aq. $Na_2S_2O_3$ (20 mL) to consume excess TBHP. The mixture was transferred to a separatory funnel and diluted with hexane (30 mL). The phases were separated and extracted with hexane (3 x 20 mL). The combined organics were washed with water and brine, dried over $MgSO_4$, filtered and concentrated under reduced pressure. The crude material was purified by flash column chromatography on silica gel (hexane/EtOAc 40/1 to 20/1) to give **5** (1.44 g, 1.44 mmol) in 84% as a colorless oil.

R_f = 0.40 (Hexane/EtOAc = 5/1).
$[\alpha]_D^{21}$: −14.0 (c = 0.44, $CHCl_3$).
^{1}H NMR (600 MHz, $CDCl_3$) δ 8.06 (ddd, *J* = 7.8, 1.8, 1.2 Hz, 2H), 7.55 (tt, *J* = 7.8, 1.2 Hz, 1H), 7.44 (ddd, *J* = 7.8, 7.8, 1.8 Hz, 2H), 7.22 (dt, *J* = 9.0, 2.4 Hz, 2H), 6.85 (dt, *J* = 9.0, 2.4 Hz, 2H), 4.99 (dd, *J* = 8.4, 4.8 Hz, 1H), 4.46 (d, *J* = 10.8 Hz, 1H), 4.41 (d, *J* = 10.8 Hz, 1H), 3.92-3.88 (m, 1H), 3.79 (s, 3H), 3.63-3.54 (m, 3H), 2.73 (dd, *J* = 16.2, 7.8 Hz, 1H), 2.49 (dd, *J* = 16.2, 7.8 Hz, 1H), 1.69-1.18 (m, 24H), 0.97 (s, 9H), 0.88 (s, 9H), 0.87 (s, 9H), 0.80 (s, 9H), 0.03 (s, 6H), 0.014 (s, 3H), 0.009 (s, 3H), -0.02 (s, 3H), -0.04 (s, 3H).
^{13}C NMR ($CDCl_3$, 151 MHz) δ 207.8, 166.4, 159.1, 132.7, 130.7, 130.6, 129.6 (2C), 129.4 (2C), 128.3 (2C), 113.7 (2C), 81.3, 75.4, 72.3, 72.2, 72.0, 71.3, 55.3, 48.6, 37.5, 37.4, 37.3 (2C), 37.1, 37.0, 34.9, 34.7, 31.2, 30.0, 26.1 (3C), 26.0 (6C), 25.9 (3C), 22.1, 21.13, 21.07 (2C), 18.1 (2C), 18.0, -4.34, -4.38 (3C), -4.4, -4.5.
IR (ATR): 2952, 2928, 2856, 2373, 2321, 1717, 1613, 1513, 1471, 1462, 1365, 1271, 1248, 1107, 1089, 1070, 834, 796, 772 cm^{-1}.
HRMS (ESI) m/z: $[M+Na]^+$ Calcd for $C_{57}H_{102}NaO_8Si_3$ 1021.6775; Found 1021.6775.

Aldol adduct 26

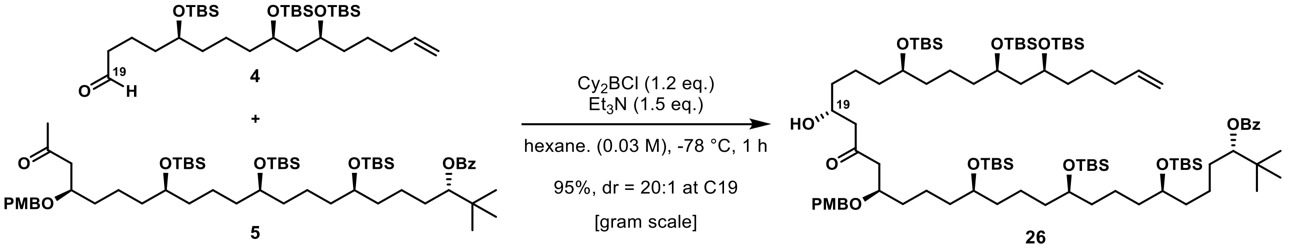

To a solution of Cy_2BCl in hexane (1.0 M, 1.8 mL, 1.2 eq.) was added Et_3N (freshly distilled over CaH_2, 250 μL, 1.8 mmol, 1.2 eq.) at 0 °C (The white precipitation was formed immediately). After stirring it for 10 min, methyl ketone **5** (1.50 g, 1.5 mmol, 1.0 eq.) in hexane (15 mL) was added dropwise to the reaction mixture. The resulting mixture was stirred for 90 min at this temperature and cooled to -78 °C by acetone-dry ice bath. Then aldehyde **4** (943 mg, 1.5 mmol, 1.0 eq.) was then added to the white suspension as a hexane solution (15 mL). The reaction was stirred for 1 h at this temperature and quenched with MeOH/pH 7 buffer (1:1). The resulting mixture was allowed to warm to 0 °C and 30% aq. H_2O_2 was added. The reaction mixture was stirred for further 1 h. The biphasic mixture was separated and the aqueous phase was extracted with Et_2O (3 x 20 mL). The organic fractions were combined, washed with brine, dried over $MgSO_4$, filtered and concentrated *in vacuo*. The residue was purified by flush column

chromatography on silica gel (Hexane/EtOAc= 20/1 to 10/1) to give aldol adduct **26** (2.32 g, 1.43 mmol) in 95% yield, dr = 20:1 at C19 as a colorless oil.

R_f = 0.53 (Hexane/EtOAc = 5/1).
$[\alpha]_D^{22}$: −13.1 (c = 0.70, $CHCl_3$).
^{1}H NMR (600 MHz, $CDCl_3$) δ 8.06 (ddd, *J* = 7.8, 1.8, 1.2 Hz, 2H), 7.55 (tt, *J* = 7.8, 1.2 Hz, 1H), 7.44 (ddd, *J* = 7.8, 7.8, 1.8 Hz, 2H), 7.22 (dt, *J* = 9.0, 2.4 Hz, 2H), 6.85 (dt, *J* = 9.0, 2.4 Hz, 2H), 5.80 (ddt, *J* = 17.4, 10.2, 6.6 Hz, 1H), 5.00 (ddt, *J* = 17.4, 1.8, 1.8 Hz, 1H), 4.99 (dd, *J* = 8.4, 4.8 Hz, 1H), 4.95 (ddt, *J* = 10.2, 1.8, 1.2 Hz, 1H), 4.46 (d, *J* = 11.4 Hz, 1H), 4.38 (d, *J* = 11.4 Hz, 1H), 4.05-3.97 (m, 1H), 3.93-3.89 (m, 1H), 3.79 (s, 3H), 3.76-3.70 (m, 2H), 3.64-3.60 (m, 2H), 3.60-3.55 (m, 2H), 3.02 (d, *J* = 3.0 Hz, 1H), 2.73 (dd, *J* = 15.6, 8.4 Hz, 1H), 2.61 (dd, *J* = 18.0, 2.4 Hz, 1H), 2.48 (dd, *J* = 18.0, 9.0 Hz, 1H), 2.45 (dd, *J* = 15.6, 4.2 Hz, 1H), 2.04 (dt, *J* = 6.6, 6.6 Hz, 2H), 1.65-1.18 (m, 42H), 0.97 (s, 9H), 0.88 (s, 36H), 0.87 (s, 9H), 0.80 (s, 9H), 0.038 (s, 6H), 0.034 (s, 12H), 0.027 (s, 6H), 0.014 (s, 3H), 0.009 (s, 3H), -0.024 (s, 3H), -0.037 (s, 3H).
^{13}C NMR ($CDCl_3$, 151 MHz) δ211.1, 166.4, 159.2, 138.9, 132.6, 130.7, 130.4, 129.6 (2C), 129.4 (2C), 128.3 (2C), 114.4, 113.8 (2C), 81.3, 75.4, 72.3 (2C), 72.1, 72.0, 71.4, 69.6, 69.5, 67.5, 55.2, 50.6, 48.4, 44.8, 37.7, 37.50, 37.48 37.3 (3C), 37.1 (2C), 37.0, 36.64, 36.60, 34.9, 34.6, 34.0, 30.0, 26.1 (3C), 25.95 (9C), 25.92 (6C), 25.87 (3C), 24.4, 22.1, 21.3, 21.12, 21.06, 21.00, 20.8, 18.1 (4C), 18.0 (2C), -4.23, -4.33 (2C), -4.37 (6C), -4.43 (2C), -4.50.
IR (ATR): 3545, 2952, 2929, 2856, 2372, 2321, 1717, 1612, 1513, 1471, 1461, 1362, 1272, 1250, 1110, 1039, 1004, 833, 805, 771, 709 cm^{-1}.
HRMS (ESI) m/z: $[M+Na]^+$ Calcd for $C_{91}H_{174}NaO_{12}Si_6$ 1650.1513; Found 1650.1515.

Diol S17

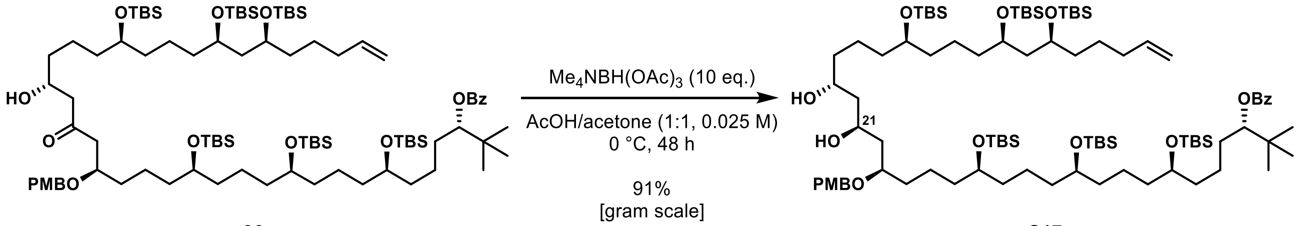

26 S17

To a solution of tetramethylammonium triacetoxy borohydride (3.21 g, 12.1 mmol, 10 eq.) in acetone/AcOH (1:2, 24.2 mL) was added β-hydroxy ketone **26** (1.98 g, 1.21 mmol, 1.0 eq.) in acetone/AcOH (2:1, 24.2 mL) dropwise at 0 °C under an Ar atmosphere. The reaction mixture was stirred for 36 h at this temperature and poured into an ice-cooled aq. K_2CO_3. The phases were separated and the aqueous phase was extracted with EtOAc (3 x 50 mL). The organic fractions were combined, washed with sat. Rochelle's salt (50 mL), sat. $NaHCO_3$ (50 mL), and brine, dried over Na_2SO_4, filtered and concentrated *in vacuo*. The crude material was purified by flash column chromatography on silica gel (hexane/EtOAc =20/1 to 10/1) to obtain 1,3-diol **S17** (1.76 g, mmol) in 90% yield, dr = 20:1 at C21 as a colorless oil.

R_f = 0.27 (Hexane/EtOAc = 5/1).

$[\alpha]_D^{24}$: −15.5 (c = 0.77, $CHCl_3$).

^{1}H NMR (600 MHz, $CDCl_3$) δ 8.06 (ddd, *J* = 7.8, 1.8, 1.2 Hz, 2H), 7.55 (tt, *J* = 7.8, 1.2 Hz, 1H), 7.44 (ddd, *J* = 7.8, 7.8, 1.8 Hz, 2H), 7.24 (dt, *J* = 9.0, 2.4 Hz, 2H), 6.87 (dt, *J* = 9.0, 2.4 Hz, 2H), 5.80 (ddt, *J* = 17.4, 10.2, 6.6 Hz, 1H), 5.01 (ddt, *J* = 17.4, 1.8, 1.8 Hz, 1H), 4.99 (dd, *J* = 8.4, 4.8 Hz, 1H), 4.94 (ddt, *J* = 10.2, 1.8, 1.2 Hz, 1H), 4.59 (d, *J* = 10.8 Hz, 1H), 4.34 (d, *J* = 10.8 Hz, 1H), 4.16 (brs, 1H), 4.14-4.10 (m, 1H), 3.91-3.86 (m, 1H), 3.80 (s, 3H), 3.76-3.67 (m, 3H), 3.64-3.60 (m, 2H), 3.60-3.55 (m, 2H), 3.01 (brs, 1H), 2.04 (dt, *J* = 6.6, 6.6 Hz, 2H), 1.87 (ddd, *J* = 15.0, 10.2, 10.2 Hz, 1H), 1.68-1.18 (m, 45H), 0.97 (s, 9H), 0.882 (s, 9H), 0.880 (s, 9H), 0.875 (s, 9H), 0.868 (s, 9H), 0.80 (s, 9H), 0.036 (s, 9H), 0.034 (s, 6H), 0.030 (s, 9H), 0.017 (s, 3H), 0.0011 (s, 3H), -0.023 (s, 3H), -0.036 (s, 3H).

^{13}C NMR ($CDCl_3$, 151 MHz) δ 166.4, 159.3, 138.9, 132.6, 130.7, 129.8, 129.6 (2C), 129.5 (2C), 128.3 (2C), 114.4, 114.0 (2C), 81.3, 80.3, 72.4, 72.3, 72.1, 72.0, 70.3, 70.1, 69.6, 69.5, 68.9, 55.3, 44.8, 42.8, 40.6, 37.9, 37.7, 37.5, 37.4, 37.32 (3C), 37.25, 37.21, 37.0, 36.6, 34.9, 34.0, 33.7, 30.0, 26.07 (3C), 25.94 (6C), 25.92 (9C), 25.86 (3C), 24.3, 22.1, 21.7, 21.1, 21.0, 20.8, 20.4, 18.12 (4C), 18.05 (2C), -4.23, -4.30, -4.33, -4.37 (3C), -4.39 (3C), -4.44 (2C), -4.50.

IR (ATR): 3447, 2951, 2928, 2856, 2372, 2322, 1719, 1613, 1514, 1471, 1461, 1272, 1251, 1089, 1039, 834, 722, 757 cm^{-1}.

HRMS (ESI) m/z: [M+Na]+ Calcd for $C_{91}H_{176}NaO_{12}Si_6$ 1652.16696; Found 1652.16699.

Silyl ether 27

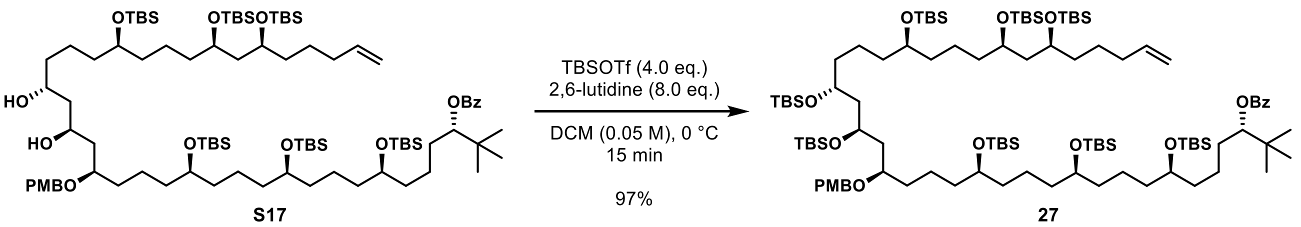

To a solution of diol **S17** (961 mg, 0.590 mmol, 1.0 eq.) and 2,6-lutidine (0.512 mL, 4.72 mmol, 8.0 eq.) in DCM (11.0 mL, 0.05 M) was added TBSOTf (0.506 mL, 2.36 mmol, 4.0 eq.) dropwise at 0 °C under an atmosphere of nitrogen. The reaction mixture was stirred for 15 min at this temperature and quenched with sat. $NaHCO_3$ (10 mL). The phases were separated and the aqueous phase was extracted with DCM (3 x 10 mL). The organic fractions were combined, washed with brine, dried over $MgSO_4$, filtered and concentrated *in vacuo*. The crude material was purified by flash column chromatography on silica gel (hexane/EtOAc = 100/1 to 50/1) to give **27** (1.07 g, 0.573mmol) in 97% yield as a colorless oil.

R_f = 0.74 (Hexane/EtOAc = 5/1).

$[\alpha]_D^{25}$: −5.49 (c = 1.26, $CHCl_3$).

^{1}H NMR (600 MHz, $CDCl_3$) δ 8.06 (ddd, *J* = 7.8, 1.8, 1.2 Hz, 2H), 7.55 (tt, *J* = 7.8, 1.2 Hz, 1H), 7.44 (ddd, *J* = 7.8, 7.8, 1.8 Hz, 2H), 7.25 (dt, *J* = 9.0, 2.4 Hz, 2H), 6.85 (dt, *J* = 9.0, 2.4 Hz, 2H), 5.80 (ddt, *J* = 17.4, 10.2, 6.6 Hz, 1H), 5.01 (ddt, *J* = 17.4, 1.8, 1.8 Hz, 1H), 4.99 (dd, *J* = 8.4, 4.8 Hz, 1H), 4.94 (ddt, *J* = 10.2, 1.8, 1.2 Hz, 1H), 4.45 (d, *J* =

11.4 Hz, 1H), 4.38 (d, J = 11.4 Hz, 1H), 3.91-3.85 (m, 1H), 3.79 (s, 3H), 3.76-3.70 (m, 3H), 3.61-3.55 (m, 4H), 3.48-3.44 (m, 1H), 2.04 (dt, J = 6.6, 6.6 Hz, 2H), 1.84 (ddd, J = 15.6, 6.6, 6.6 Hz, 1H), 1.65-1.16 (m, 45H), 0.97 (s, 9H), 0.882 (s, 18H), 0.878 (s, 18H), 0.873 (s, 9H), 0.865 (s, 9H), 0.863 (s, 9H), 0.80 (s, 9H), 0.066 (s, 3H), 0.041 (s, 9H), 0.037 (s, 9H), 0.031 (s, 9H), 0.025 (s, 6H), 0.012 (s, 3H), 0.006 (s, 3H), -0.026 (s, 3H), -0.040 (s, 3H).

^{13}C NMR ($CDCl_3$, 151 MHz) δ 166.3, 158.9, 138.9, 132.6, 131.1, 130.7, 129.6 (2C), 129.1 (2C), 128.3 (2C), 114.4, 113.6 (2C), 81.3, 76.2, 72.42, 72.40, 72.3, 72.0, 70.6, 70.1, 69.6, 69.5, 67.7, 55.2, 46.0, 44.8, 43.2, 38.4, 37.8, 37.6, 37.54, 37.48 (2C), 37.40, 37.38, 37.3, 37.0, 36.6, 34.9, 34.7, 34.0, 30.0, 26.07 (3C), 26.97 (15C), 25.92 (6C), 25.86 (3C), 24.4, 22.1, 21.5, 21.1 (2C), 20.8, 18.12 (3C), 18.07, 18.05 (2C), 18.03 (2C), -3.84, -3.91, -3.96, -4.10, -4.22, -4.35 (5C), -4.39 (3C), -4.43 (2C), -4.51.

IR (ATR): 2952, 2928, 2856, 1719, 1513, 1471, 1462, 1361, 1271, 1252, 1107, 1087, 1070, 1039, 910, 881, 834, 795, 771, 758 cm^{-1}.

HRMS (ESI) m/z: $[M+Na]^+$ Calcd for $C_{103}H_{204}NaO_{12}Si_8$ 1881.34232; Found 1881.34205.

Secondary alcohol S18

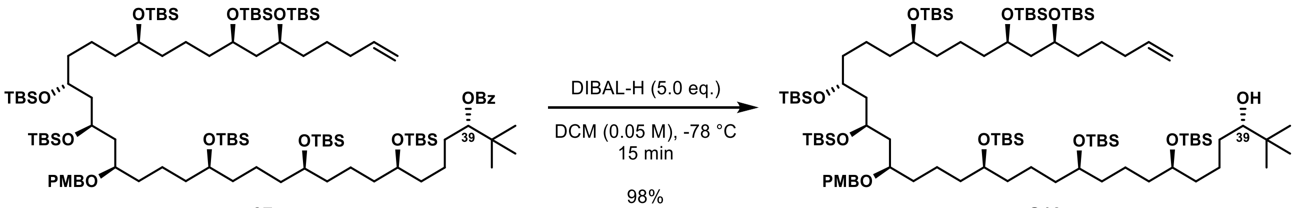

27 **S18**

To a solution of benzoate **27** (1.06 g, 0.57 mmol, 1.0 eq.) in DCM (11.4 mL, 0.05 M) was added DIBAL-H (1.0 M in hexane, 2.28 mL, 4.0 eq.) dropwise at -78 °C under Ar atmosphere. Ther action mixture was stirred for 30 min and EtOAc (10 mL) was added slowly. The resulting mixture was allowed to warm to 0 °C and added sat. Rochelle's salt (20 mL). the resulting suspension was vigorously stirred for 12 h to give clear biphasic mixture. The phases were separated and the aqueous phase was extracted with EtOAc (3 x 20 mL). The organic fractions were combined, washed with brine, dried over Na_2SO_4, filtered and concentrated *in vacuo*. The residue was purified by column chromatography on silica gel (Hexane/EtOAc= 75/1 to 30/1) to furnish alcohol **S18** (981 mg, 0.56 mmol) in 98% yield as a colorless oil.

R_f = 0.73 (Hexane/EtOAc = 5/1).

$[\alpha]_D^{24}$: −7.57 (c = 0.67, $CHCl_3$).

^{1}H NMR (600 MHz, $CDCl_3$) δ 7.25 (dt, J = 9.0, 2.4 Hz, 2H), 6.85 (dt, J = 9.0, 2.4 Hz, 2H), 5.80 (ddt, J = 17.4, 10.2, 6.6 Hz, 1H), 5.00 (ddt, J = 17.4, 1.8, 1.8 Hz, 1H), 4.94 (ddt, J = 10.2, 1.8, 1.2 Hz, 1H), 4.45 (d, J = 11.4 Hz, 1H), 4.38 (d, J = 11.4 Hz, 1H), 3.91-3.85 (m, 1H), 3.79 (s, 3H), 3.76-3.70 (m, 3H), 3.65-3.58 (m, 4H), 3.48-3.44 (m, 1H), 3.17 (d, J = 10.8 Hz, 1H), 2.04 (dt, J = 6.6, 6.6 Hz, 2H), 1.84 (ddd, J = 15.6, 6.6, 6.6 Hz, 1H), 1.65-1.20 (m, 45H), 0.889 (s, 9H), 0.882 (s, 36H), 0.878 (s, 18H), 0.874 (s, 9H), 0.863 (s, 9H), 0.067 (s, 3H), 0.041 (s, 9H), 0.036 (s, 9H), 0.033 (s, 15H), 0.031 (s, 12H).

^{13}C NMR ($CDCl_3$, 151 MHz) δ 158.9, 138.9, 131.2, 129.2 (2C), 114.4, 113.6 (2C), 79.9, 76.2, 72.43, 72.41, 72.36, 72.31, 70.6, 70.1, 69.6, 69.5, 67.8, 55.2, 46.0, 44.9, 43.2, 38.4, 37.8, 37.56 (4C), 37.49, 37.43, 37.35

37.1, 36.6, 34.9, 34.7, 34.0, 31.7, 26.0 (18C), 25.9 (6C), 25.7 (3C), 24.4, 22.9, 21.5, 21.2, 21.10, 21.06, 20.8, 18.14 (4C), 18.09 (2C), 18.05 (3C), -3.83, -3.91, -3.96, -4.10, -4.22, -4.34 (6C), -4.36 (4C), -4.44.
IR (ATR): 3522, 2951, 2929, 2856, 1514, 1472, 1462, 1362, 1251, 1111, 1041, 1104, 833, 805, 771 cm^{-1}.
HRMS (ESI) m/z: $[M+Na]^+$ Calcd for $C_{96}H_{200}NaO_{11}Si_8$ 1776.31370; Found 1776.31386.

(*Z*)-3-iodobut-2-enoic acid

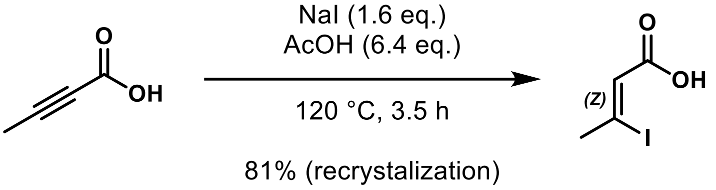

(*Z*)-3-iodobut-2-enoic acid was prepared by the known protocol.[7]

2-Butynoic acid (1.0 g, 12 mmol, 1.0 eq.) and sodium iodide (2.87 g, 19.2 mmol, 1.6 eq.) were dissolved in AcOH (4.4 mL, 76.8 mmol, 4.4 eq.) under an atmosphere of nitrogen. The reaction mixture was heated to 120 °C on an oil bath for 3.5 h. The resulting mixture was cooled to ambient temperature and diluted in Et_2O/H_2O (20 mL each). Then solid $NaHSO_3$ was added to biphasic mixture until it changes to colorless solution. The phases were separated and the aqueous phase was extracted with Et_2O (3 x 20 mL). The organic fraction was combined, dried over $MgSO_4$, filtered and concentrated under reduced pressure to give white solid. The solid was purified by recrystallization from $CHCl_3$/heptane to obtain (*Z*)-3-iodobut-2-enoic acid (2.07 g, 9.8 mmol) in 81% yield as a colorless crystal. All spectroscopic data (^1H NMR, ^{13}C NMR, IR, HRMS) was identical to the known compound.[7]

Acid anhydride 28

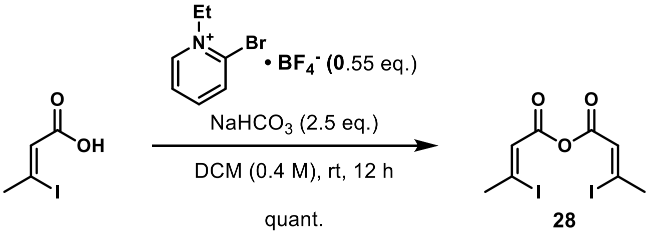

To a solution of (*Z*)-3-iodobut-2-enoic acid (848 mg, 4.0 mmol, 1.0 eq.) and $NaHCO_3$ (840 mg, 10 mmol, 2.5 eq.) in DCM (10 mL, 0.4 M) was added 1-ethyl-2-bromo-pyridinium tetrafluoroborate (602 mg, 2.2 mmol, 0.55 eq.) under an atmosphere of argon at ambient temperature. The reaction vessel was wrapped by aluminum foil and the reaction was stirred for 12 h. The reaction mixture was filtered through silica gel plug and solvent was removed under high vacuum to furnish acid anhydride **28** (807 mg, 2.0 mmol) in quantitative yield as a white solid.

Rf = 0.36 (Hexane/EtOAc = 5/1)
m.p. = 69.5-70.9 °C
^1H NMR (CDCl3, 600 MHz) δ 6.40 (q, J = 1.2 Hz, 2H), 2.81 (d, J = 1.2 Hz, 6H).
^{13}C NMR (CDCl3, 151 MHz) δ 159.1, 124.4, 120.1, 37.2.

IR (ATR); 3023, 1783, 1723, 1604, 1430, 1377, 1303, 1092, 1059, 992, 917, 847 cm^{-1}.
HRMS (ESI); m/z: $[M]^+$ Calcd for C8H8I2O3 405.8563; Found 405.8563.

Alkenyl iodide 29

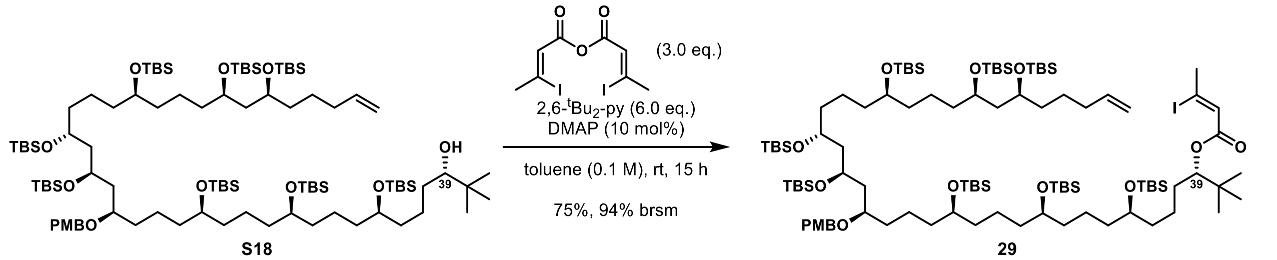

To a solution of secondary alcohol **S18** (980 mg, 0.56 mmol, 1.0 eq.), 2,6-^{t}Bu$_2$-pyridine (0.72 mL, 3.36 mmol, 6.0 eq.), and acid anhydride **28** (682 mg, 1.68 mmol, 3.0 eq.) in toluene (5.6 mL, 0.1 M) was added DMAP (6.8 mg, 0.056 mmol, 10 mol%) in toluene (0.1 mL) at 0 °C under an Ar atmosphere. The reaction mixture was warmed to ambient temperature and stirred for 15 h. The resulting mixture was diluted by DCM and filtered through a silica gel plug. The filtrate was concentrated under reduced pressure and the residue was purified by flash column chromatography (hexane/EtOAc = 70/1) to obtain **29** (818 mg, 0.42 mmol) in 75% yield (94% yield, brsm) as a clear colorless oil. Unreacted alcohol **S18** (197 mg, 0.11 mmol) was recovered in 20% (hexane/EtOAc = 40/1).

R_f = 0.81 (Hexane/EtOAc = 5/1).
$[\alpha]_D^{25}$: +0.93 (c = 1.6, $CHCl_3$).
^{1}H NMR (600 MHz, $CDCl_3$) δ 7.25 (dt, J = 9.0, 2.4 Hz, 2H), 6.85 (dt, J = 9.0, 2.4 Hz, 2H), 6.30 (q, J = 1.2 Hz, 1H), 5.80 (ddt, J = 17.4, 10.2, 6.6 Hz, 1H), 5.00 (ddt, J = 17.4, 1.8, 1.8 Hz, 1H), 4.94 (ddt, J = 10.2, 1.8, 1.2 Hz, 1H), 4.84 (dd, J = 9.6, 3.0 Hz, 1H), 4.45 (d, J = 11.4 Hz, 1H), 4.38 (d, J = 11.4 Hz, 1H), 3.91-3.85 (m, 1H), 3.79 (s, 3H), 3.76-3.70 (m, 3H), 3.65-3.58 (m, 4H), 3.48-3.44 (m, 1H), 2.73 (d, J = 1.2 Hz, 3H), 2.04 (dt, J = 6.6, 6.6 Hz, 2H), 1.84 (ddd, J = 15.6, 6.6, 6.6 Hz, 1H), 1.66-1.20 (m, 45H), 0.90 (s, 9H), 0.883 (s, 27H), 0.880 (s, 18H), 0.875 (s, 9H), 0.869 (s, 9H), 0.864 (s, 9H), 0.068 (s, 3H), 0.042 (s, 9H), 0.037 (s, 9H), 0.032 (s, 18H), 0.025 (s, 3H), 0.020 (s, 3H).
^{13}C NMR ($CDCl_3$, 151 MHz) δ 164.2, 158.9, 138.9, 131.2, 129.1 (2C), 125.9, 114.4, 113.6 (2C), 112.7, 81.1, 76.2, 72.43, 72.41, 72.3, 72.0, 70.6, 70.2, 69.6, 69.5, 67.8, 55.2, 46.0, 44.9, 43.2, 38.4, 37.8, 37.6, 37.55 (2C), 37.50, 37.46, 37.39 (2C), 36.9, 36.6, 36.5, 34.7, 34.6, 34.0, 29.8, 26.04 (3C), 25.98 (18C), 25.93 (6C), 24.4, 22.1, 21.5, 21.2, 21.1, 20.8, 18.14 (3C), 18.12 (2C), 18.09, 18.05 (3C), -3.82, -3.91, -3.95, -4.09, -4.21, -4.33 (6C), -4.36 (4C), -4.43.
IR (ATR): 2952, 2928, 2856, 1730, 1625, 1471, 1250, 1171, 1088, 1040, 885, 834, 806, 772 cm^{-1}.
HRMS (ESI) m/z: $[M+Na]^+$ Calcd for $C_{100}H_{203}INaO_{12}Si_8$ 1970.23656; Found 1970.23671.

Macrolactone 30

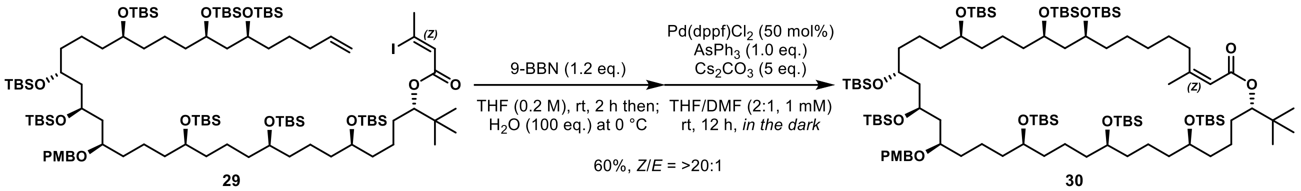

Hydroboration

To a solution of iodide **29** (195 mg, 0.10 mmol, 1.0 eq.) in THF (1.0 mL) was added 9-BBN (0.5 M in THF, 0.24 mL, 1.2 eq.) at room temperature under Ar atmosphere. The reaction was stirred for 1 h and diluted with THF (4.0 mL). The reaction was cooled to 0 °C and quenched with H_2O (0.18 mL, 10 mmol, 100 eq.). The reaction was stirred for an additional 1 h at room temperature. The resulting alkyl borane solution **A** was degassed by freeze-pump-thaw three times.

Intramolecular Suzuki coupling

A 3-neck round-bottomed flask containing magnetic stirrer bar was parged by Ar. $Pd(dppf)Cl_2$ (36.6 mg, 0.05 mmol, 50 mol%), $AsPh_3$ (30.6 mg, 0.1 mmol, 100 mol%), and Cs_2CO_3 (326 mg, 1.0 mmol, 10 eq.) was added and parged with Ar three times. The reaction vessel was wrapped with aluminum foil and THF/DMF (100 mL, 2:1, pre-degassed by Ar bubbling) was added at room temperature. The resulting mixture was stirred vigorously till it turned to yellow in color (approx. 10 min). Then, alkyl borane solution **A** was added over 1 min via syringe to the mixture. The resulting suspension was stirred for 12 h at room temperature and quenched with sat. NH_4Cl (100 mL). The phases were separated and the aqueous phase was extracted with hexane (3 x 50 mL). the organic fractions were combined, washed with water and brine, dried over $MgSO_4$, filtered through a pad of celite® and concentrated *in vacuo*. The crude product was purified by flash column chromatography (hexane/EtOAc = 80/1 to 50/1) to obtain macrolactone **30** (110 mg, 0.061 mmol) in 61%, *Z*/*E* = >99/1 as colorless oil.

R_f = 0.81 (Hexane/EtOAc = 5/1).

$[\alpha]_D^{26}$: −3.30 (c = 0.56, $CHCl_3$).

^{1}H NMR (600 MHz, $CDCl_3$) δ 7.25 (dt, *J* = 9.0, 2.4 Hz, 2H), 6.85 (dt, *J* = 9.0, 2.4 Hz, 2H), 5.66 (s, 1H), 4.75 (dd, *J* = 10.8, 1.8 Hz, 1H), 4.46 (d, *J* = 11.4 Hz, 1H), 4.38 (d, *J* = 11.4 Hz, 1H), 3.85-3.78 (m, 1H), 3.79 (s, 3H), 3.77-3.67 (m, 3H), 3.66-3.57 (m, 4H), 3.46-3.43 (m, 1H), 2.70 (ddd, *J* = 12.0, 8.4, 8.4 Hz, 1H), 2.49 (ddd, *J* = 12.0, 8.4, 8.4 Hz, 1H), 1.87 (d, *J* = 1.2 Hz, 3H), 1.82 (ddd, *J* = 13.8, 6.6, 6.6 Hz, 1H), 1.66-1.20 (m, 49H), 0.88 (brs, 54H), 0.873 (s, 9H), 0.864 (s, 18H), 0.068 (s, 3H), 0.044 (s, 6H), 0.039 (s, 6H), 0.035 (s, 9H), 0.032 (s, 18H), 0.022 (s, 3H), 0.015 (s, 3H).

^{13}C NMR ($CDCl_3$, 151 MHz) δ 166.2, 160.0, 158.9, 131.1, 129.2 (2C), 116.4, 113.6 (2C), 79.3, 76.2, 72.38, 72.33, 72.24, 72.0, 70.6, 69.7 (2C), 69.6, 67.6, 55.2, 45.9, 45.0, 43.0, 38.0, 37.8, 37.7, 37.5, 37.48, 37.44, 37.42, 37.38, 37.29, 37.20, 37.0, 34.8, 34.5, 33.6, 30.2, 29.9, 28.4, 26.02 (3C), 25.98 (12C), 25.95 (12C), 25.2, 25.15, 21.9, 21.7, 21.2, 21.1, 20.73, 20.70, 18.13 (3C), 18.08 (2C), 18.05 (2C), 18.01, -3.93, -3.95, -4.13, -4.18, -4.22, -4.26, -4.31 (2C), -4.33 (2C), -4.36 (3C), -4.38 (3C).

IR (ATR): 2952, 2927, 2856, 1716, 1513, 1741, 1462, 1378, 1251, 1088, 1042, 833, 805, 771 cm^{-1}.

HRMS (ESI) m/z: $[M+Na]^+$ Calcd for $C_{100}H_{204}NaO_{12}Si_8$ 1844.3399; Found 1844.3399.

Secondary alcohol S19

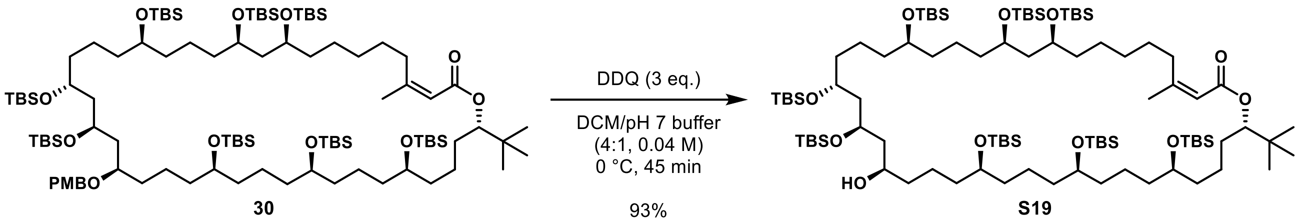

To a solution of PMB ether **00** (182 mg, 0.1 mmol, 1.0 eq.) in DCM/pH7 buffer (4:1, 2.5 mL, 0.04 M) was added DDQ (68.1 mg, 0.3 mmol, 3.0 eq.) at 0 °C under Ar atmosphere. The reaction mixture was stirred rapidly for 45 min and quenched with sat. $NaHCO_3$. The phases were separated and the aqueous phase was extracted with DCM (3 x 5 mL). The organic fractions were combined, washed with sat. $NaHCO_3$ and brine, dried over $MgSO_4$, filtered and concentrated *in vacuo*. The residue was purified by flash column chromatography on silica gel (hexane/EtOAc = 50:1) to afford secondary alcohol **00** (158 mg, 0.093 mmol) in 93% yield as a colorless oil.

R_f = 0.71 (Hexane/EtOAc = 5/1).

$[\alpha]_D^{22}$: +0.67 (c = 1.01, $CHCl_3$).

^1H NMR (600 MHz, $CDCl_3$) δ 5.66 (d, *J* = 1.2 Hz, 1H), 4.75 (dd, *J* = 10.8, 2.4 Hz, 1H), 3.92-3.87 (m, 1H), 3.77-3.66 (m, 4H), 3.66-3.57 (m, 4H), 3.05 (brs, 1H), 2.67 (ddd, *J* = 12.0, 8.4, 8.4 Hz, 1H), 2.52 (ddd, *J* = 12.0, 8.4, 8.4 Hz, 1H), 1.88 (d, *J* = 1.2 Hz, 3H), 1.66-1.20 (m, 50H), 0.89 (s, 9H), 0.884 (s, 18H), 0.881 (s, 36H), 0.878 (s, 18H), 0.866 (s, 9H), 0.113 (s, 3H), 0.110 (s, 3H), 0.060 (s, 3H), 0.054 (s, 3H), 0.038 (s, 15H), 0.034 (s, 9H), 0.030 (s, 6H), 0.022 (s, 3H), 0.018 (s, 3H).

^{13}C NMR ($CDCl_3$, 151 MHz) δ 166.2, 160.0, 116.3, 79.3, 72.3, 72.2, 72.02, 71.97, 71.88, 70.7, 69.7 (3C), 46.5, 45.0, 44.0, 37.9, 37.8, 37.54, 37.45, 37.40, 37.34, 37.30 (2C), 37.20 (2C), 37.07, 37.00, 34.6, 33.6, 30.2, 29.9, 28.4, 26.03 (3C), 25.97 (18C), 25.92 (3C), 25.83 (3C), 25.20, 25.16, 21.9, 21.23, 21.17, 21.12, 20.7, 20.5, 18.13 (2C), 18.11, 18.09, 18.06 (3C), 17.9, -3.8, -4.19, -4.22 (2C), -4.25, -4.36 (4C), -4.39 (7C).

IR (ATR): 3519, 2952, 2928, 2857, 1716, 1647, 1472, 1462, 1379, 1362, 1254, 1072, 1047, 1004, 833, 806, 771, 758 cm^{-1}.

HRMS (ESI) m/z: $[M+Na]^+$ Calcd for $C_{92}H_{196}NaO_{11}Si_8$ 1724.2824; Found 1724.2826.

Bastimolide A (1)

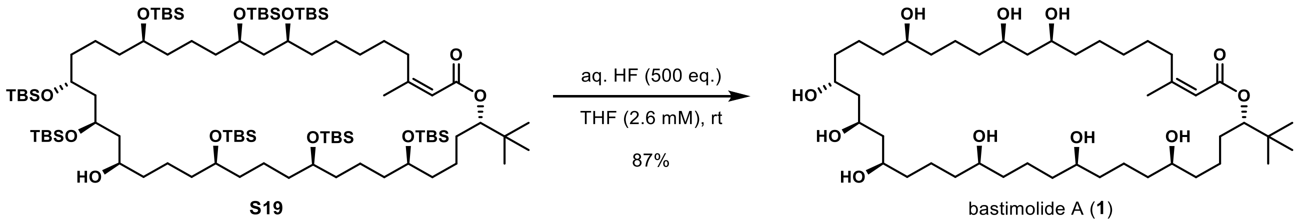

To a solution of octa silyl ether **S19** (140 mg, 0.082 mmol, 1.0 eq.) in THF (31.5 mL, 0.0026 M) was added 48%

aq. HF (1.51 mL, 41 mmol, 500 eq.) at room temperature under Ar atmosphere. The reaction was stirred for 9 h and concentrated under reduced pressure at 25 °C. The resulting mixture was purified by flash column chromatography on silica gel (EtOAc/MeOH = 50/1 to 5/1) to obtain bastimolide A (**1**) (56.3 mg, 0.071 mmol) in 87% yield as a white solid.

R_f = 0.37 (EtOAc/MeOH = 3/1).
$[\alpha]_D^{22}$: −9.78 (c = 0.79, MeOH).
^{1}H NMR (pyridine-d_5, 600 MHz) δ 6.00 (brs, 9H), 5.89 (s, 1H), 5.09 (dd, J = 10.4, 2.4 Hz, 1H), 4.78-4.74 (m, 1H), 4.47-4.43 (m, 1H), 4.30-4.26 (m, 1H), 4.24-4.21 (m, 1H), 4.18-4.15 (m, 1H), 3.997-3.88 (m, 4H), 2.96 (ddd, J = 12.0, 8.4, 8.4 Hz), 2.62 (ddd, J = 12.0, 8.4, 8.4 Hz), 2.16-2.01 (m, 5H), 1.99-1.86 (m, 8H), 1.86 – 1.60 (m, 34H), 1.59-1.48 (m, 4H), 1.47-1.36 (m, 3H), 0.95 (s, 9H).
^{13}C NMR (pyridine-d_5, 151 MHz) δ 166.9, 160.9, 117.3, 79.9, 72.1, 72.0, 71.5, 71.22, 71.19 (2C), 71.1, 69.4, 68.7, 45.4, 45.1 (2C), 39.4, 39.2 (2C), 39.1, 39.04, 38.96 (2C), 38.9, 38.8, 38.6 (3C), 35.1, 33.8, 30.6, 30.5, 29.0, 26.5 (3C), 26.3, 25.4, 23.6, 23.4, 23.3, 22.9, 22.7, 22.5.
IR (ATR): 3299, 2922, 2858, 1716, 1697, 1648, 1455, 1223, 1145, 1122, 1030, 794 cm^{-1}.
HRMS (ESI) m/z: $[M+Na]^+$ Calcd for $C_{44}H_{84}NaO_{11}$ 811.59058; Found 811.59060.

3. ^{13}C NMR Comparison of Bastimolide A (1) in pyridine-d$_5$ / ^{1}H and ^{13}C NMR Comparison of Bastimolide A (1) in methanol-d$_4$

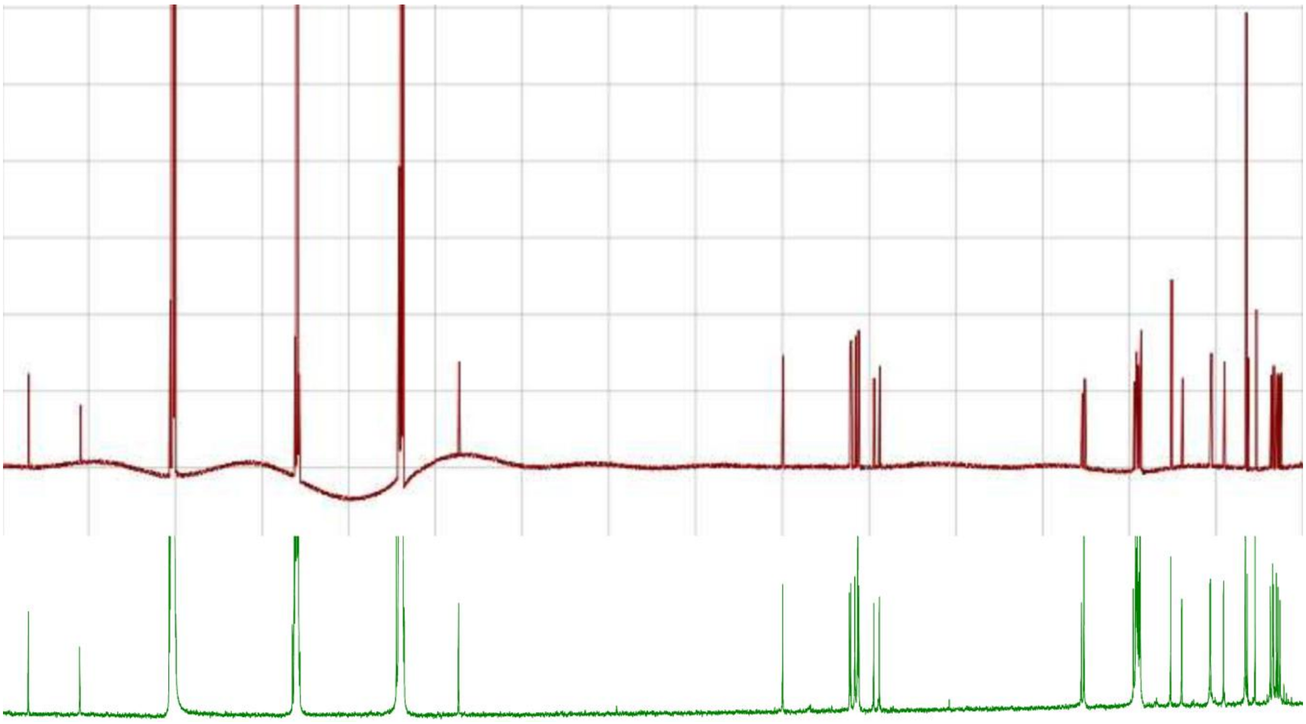

Figure 1. ^{13}C NMR overlay of natural (top, red, 125 MHz) and synthetic (bottom, green, 151 MHz) **1** in pyridine-d$_5$

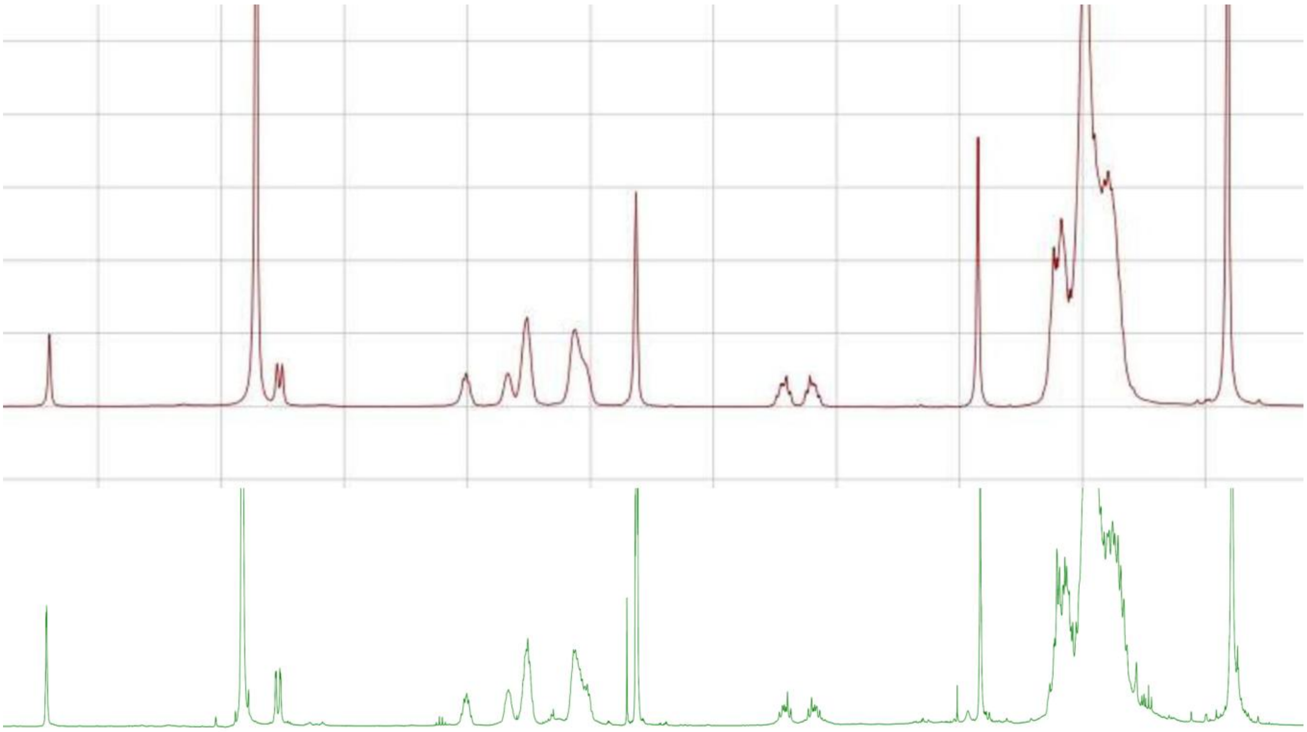

Figure 2. ^{1}H NMR overlay of natural (top, red, 500 MHz) and synthetic (bottom, green, 600 MHz) **1** in methanol-d_4

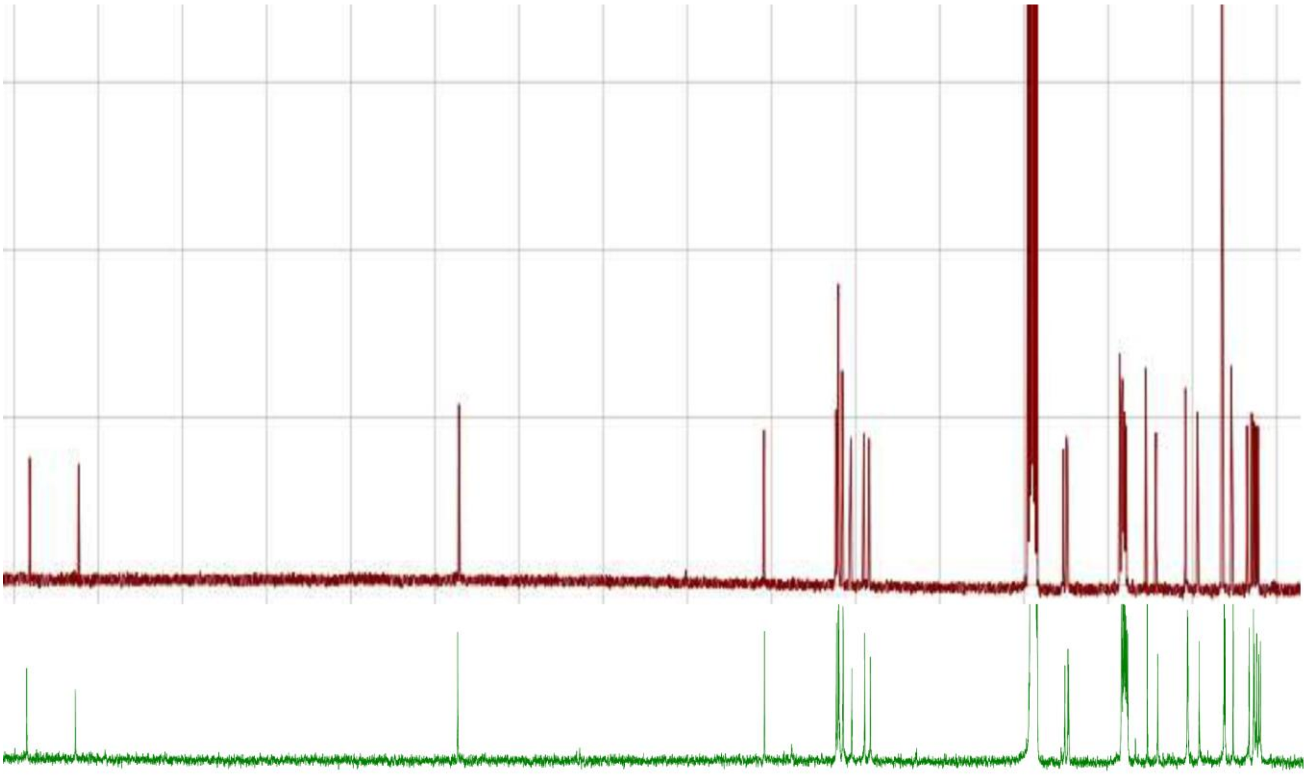

Figure 3. ^{13}C NMR overlay of natural (top, red, 125 MHz) and synthetic (bottom, green, 151 MHz) **1** in methanol-d_4

4. Determination of the Stereochemistry (at C23, C27, C31 and C35)

The relative or absolute configurations at C23, C27, C31, and C35 were confirmed by ^{1}H NMR analysis of (*R*)- and (*S*)-MTPA ester derived from corresponding secondary alcohols.

・The relative configuration at C23 (compound **S15**)

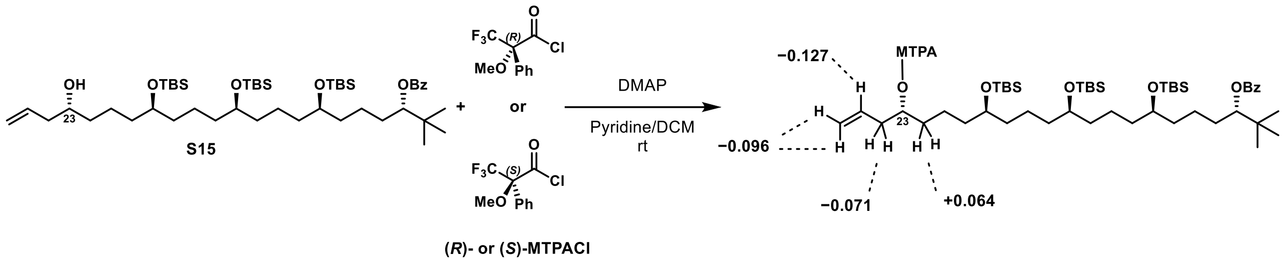

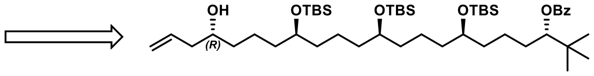

The absolute configuration at C23 was (*R*).

To a solution of alcohol **S15** (43.1 mg, 0.05 mmol) in pyridine (200 µL, 0.25 M) was added (*S*)-(+)-α-methoxy-α-(trifluoromethyl)phenylacetyl chloride (MTPACl, 18.9 mg, 0.075 mmol) at room temperature and stirred until alcohol **S15** was completely consumed. The reaction mixture was diluted in DCM (1 mL) and quenched with aq. $NaHCO_3$ (1 mL). The phases were separated and the organic layer was washed with aq. HCl (1 M) and brine, dried over $MgSO_4$, filtered and concentrated in vacuo. The residue was purified by column chromatography to give (*R*)-MTPA ester **S20** (54.0 mg, 0.05 mmol) in quantitative yield as a colorless oil. (*S*)-MTPA **S21** ester was prepared from (*R*)-MTPACl by the same method.

(*R*)-MTPA ester **S20**

^{1}H NMR (600 MHz, $CDCl_3$) δ 8.06 (ddd, *J* = 7.8, 1.8, 1.2 Hz, 2H), 7.56-7.53 (m, 3H), 7.44 (ddd, *J* = 7.8, 7.8, 1.8 Hz, 2H), 7.41-7.36 (m, 3H), 5.75 (ddt, *J* = 16.8, 10.2, 7.2 Hz, 1H), 5.17-5.13 (m, 1H), 5.12-5.09 (m, 2H), 4.99 (dd, *J* = 8.4, 4.8 Hz, 1H), 3.59-3.54 (m, 3H), 3.54 (s, 3H), 2.44-2.40 (m, 2H), 1.68-1.16 (m, 24H), 0.97 (s, 9H), 0.87 (s, 9H), 0.86 (s, 9H), 0.80 (s, 9H), 0.011 (s, 6H), 0.007 (s, 3H), 0.005 (s, 3H), -0.025 (s, 3H), -0.038 (s, 3H).

(*S*)-MTPA ester **S21**

^{1}H NMR (600 MHz, $CDCl_3$) δ 8.06 (ddd, *J* = 7.8, 1.8, 1.2 Hz, 2H), 7.56-7.53 (m, 3H), 7.44 (ddd, *J* = 7.8, 7.8, 1.8 Hz, 2H), 7.41-7.36 (m, 3H), 5.63 (ddt, *J* = 16.8, 10.2, 7.2 Hz, 1H), 5.16-5.11 (m, 1H), 5.02-4.98 (m, 2H), 4.99 (dd, *J* = 8.4, 4.8 Hz, 1H), 3.60-3.54 (m, 3H), 3.54 (s, 3H), 2.39-2.30 (m, 2H), 1.70-1.18 (m, 24H), 0.97 (s, 9H), 0.87 (s, 9H), 0.86 (s, 9H), 0.80 (s, 9H), 0.016 (s, 3H), 0.010 (s, 3H), 0.007 (s, 6H), -0.025 (s, 3H), -0.037 (s, 3H).

・The absolute configuration at C27 (compound **S12**)

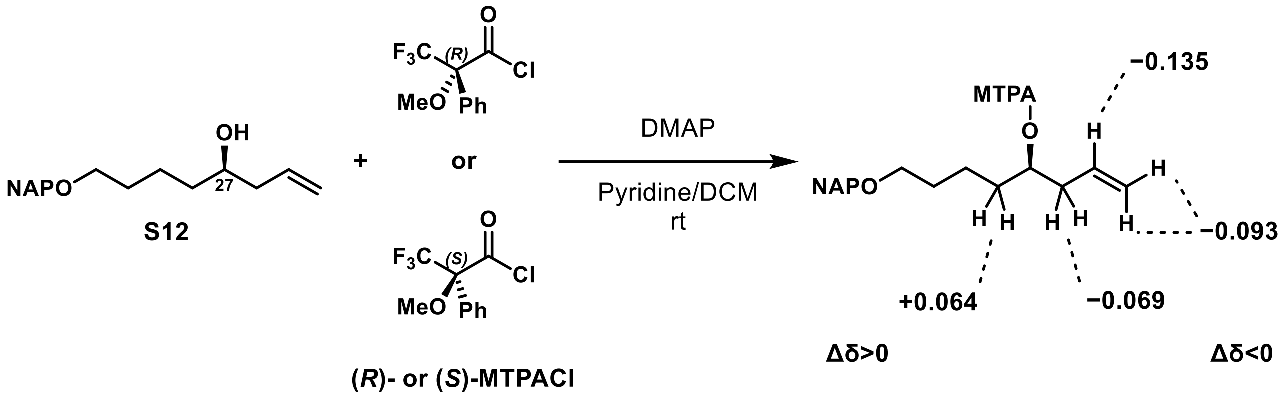

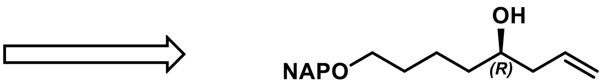

The absolute configuration at C27 was **(*R*)**.

To a solution of alcohol **S12** (14.2 mg, 0.05 mmol) in pyridine (200 µL, 0.25 M) was added (*S*)-(+)-α-methoxy-α-(trifluoromethyl)phenylacetyl chloride (MTPACl, 18.9 mg, 0.075 mmol) at room temperature and stirred until alcohol **S12** was completely consumed. The reaction mixture was diluted in DCM (1 mL) and quenched with aq. $NaHCO_3$ (1 mL). The phases were separated and the organic layer was washed with aq. HCl (1 M) and brine, dried over $MgSO_4$, filtered and concentrated in vacuo. The residue was purified by column chromatography to give (*R*)-MTPA ester **S22** (25.0 mg, 0.05 mmol) in quantitative yield as a colorless oil. (*S*)-MTPA **S23** ester was prepared from (*R*)-MTPACl by the same method.

(*R*)-MTPA ester **S22**

^{1}H NMR (600 MHz, $CDCl_3$) δ 7.84-7.82 (m, 3H), 7.76 (s, 1H),7.54-7.52 (m, 2H) 7.50-7.44 (m, 3H), 7.36-7.29 (m, 3H), 5.74 (ddt, *J* =14.4, 10.2, 7.2 Hz, 1H), 5.16 (m, 1H), 5.11-5.07 (m, 2H), 4.63 (s, 2H), 3.54 (s, 3H), 3.41 (t, *J* = 6.6 Hz, 2H), 2.44-2.38 (m, 2H), 1.65-1.51 (m, 4H), 1.34-1.22 (m, 2H).

(*S*)-MTPA ester **S23**

^{1}H NMR (600 MHz, $CDCl_3$) δ 7.84-7.81 (m, 3H), 7.76 (s, 1H),7.53-7.51 (m, 2H) 7.49-7.43 (m, 3H), 7.37-7.32 (m, 3H), 5.61 (ddt, *J* =14.4, 10.2, 7.2 Hz, 1H), 5.14 (m, 1H), 5.02-4.98 (m, 2H), 4.65 (s, 2H), 3.52 (s, 3H), 3.48 (t, *J* = 6.6 Hz, 2H), 2.34-2.30 (m, 2H), 1.71-1.58 (m, 4H), 1.50-1.37 (m, 2H).

・The absolute configuration at C31 (compound **S13**)

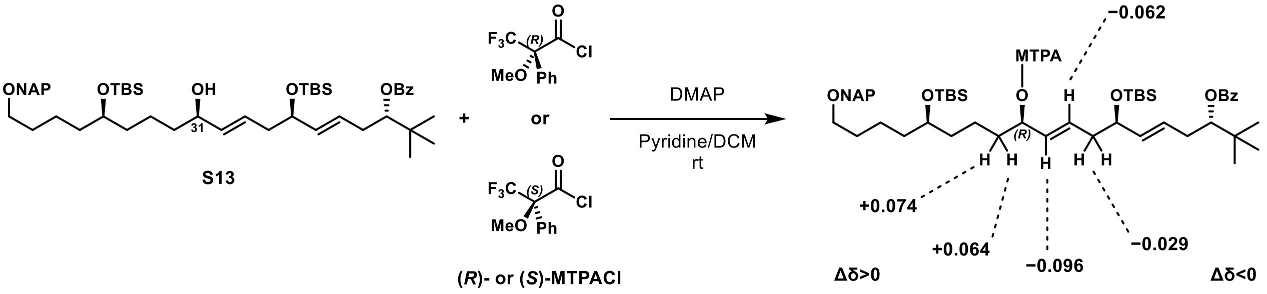

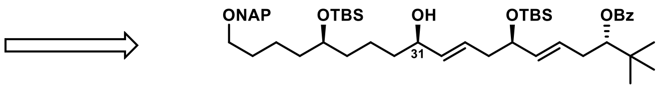

The absolute configuration at C31 was (*R*).

To a solution of alcohol **00** (42.3 mg, 0.05 mmol) in pyridine (200 μL, 0.25 M) was added (*S*)-(+)-α-methoxy-α-(trifluoromethyl)phenylacetyl chloride (MTPACl, 18.9 mg, 0.075 mmol) at room temperature and stirred until alcohol **S13** was completely consumed. The reaction mixture was diluted in DCM (1 mL) and quenched with aq. $NaHCO_3$ (1 mL). The phases were separated and the organic layer was washed with aq. HCl (1 M) and brine, dried over $MgSO_4$, filtered and concentrated in vacuo. The residue was purified by column chromatography to give (*R*)-MTPA ester **S24** (53.0 mg, 0.05 mmol) in quantitative yield as a colorless oil. (*S*)-MTPA **S25** ester was prepared from (*R*)-MTPACl by the same method.

(*R*)-MTPA ester **S24**

^{1}H NMR (600 MHz, $CDCl_3$) δ 8.02 (ddd, *J* = 7.8, 1.8, 1.2 Hz, 2H), 7.83-7.81 (m, 3H), 7.78 (s, 1H), 7.52-7.43 (m, 6H), 7.41-7.34 (m, 5H), 5.68 (td, *J* = 7.8, 7.2 Hz, 1H), 5.49 (ddd, *J* = 14.4, 7.8, 6.0 Hz, 1H), 5.38 (dd, *J* = 15.0, 6.6 Hz, 1H), 5.32 (td, *J* = 7.8, 7.2 Hz, 1H), 5.22 (dd, *J* = 15.6, 7.8 Hz, 1H), 4.99 (dd, *J* = 10.8, 2.4 Hz, 1H), 4.67 (s, 2H), 3.96 (td, *J* = 6.0, 6.0 Hz, 1H), 3.55-3.52 (m, 1H), 3.52 (s, 3H), 3.50 (t, *J* = 6.6 Hz, 2H), 2.44-2.40 (m, 1H), 2.24 (ddd, *J* = 14.4, 10.8, 8.4 Hz, 1H), 1.94-1.92 (m, 2H), 1.65-1.09 (m, 12H), 0.99 (s, 9H), 0.85 (s, 9H), 0.81 (s, 9H), 0.04 (s, 3H), -0.018 (s, 3H), -0.061 (s, 3H), -0.064 (s, 3H).

(*S*)-MTPA ester **S25**

^{1}H NMR (600 MHz, $CDCl_3$) δ 8.02 (ddd, *J* = 7.8, 1.8, 1.2 Hz, 2H), 7.83-7.81 (m, 3H), 7.77 (s, 1H), 7.51 (tt, *J* = 7.8, 1.2 Hz, 1H),7.49-7.44 (m, 5H), 7.41 (ddd, *J* = 7.8, 7.8, 1.8 Hz, 2H), 7.39-7.33 (m, 3H), 5.62 (td, *J* = 7.8, 7.2 Hz, 1H), 5.49 (ddd, *J* = 14.4, 7.8, 6.0 Hz, 1H), 5.37 (dd, *J* = 15.0, 6.6 Hz, 1H), 5.31 (td, *J* = 7.8, 7.2 Hz, 1H), 5.12 (dd, *J* = 15.6, 7.8 Hz, 1H), 4.99 (dd, *J* = 10.8, 2.4 Hz, 1H), 4.66 (s, 2H), 3.91 (td, *J* = 6.0, 6.0 Hz, 1H), 3.61-3.58 (m, 1H), 3.53 (s, 3H), 3.50 (t, *J* = 6.6 Hz, 2H), 2.45-2.41 (m, 1H), 2.25 (ddd, *J* = 14.4, 10.8, 8.4 Hz, 1H), 1.91-1.89 (m, 2H), 1.68-1.20 (m, 12H), 0.99 (s, 9H), 0.86 (s, 9H), 0.81 (s, 9H), 0.016 (s, 3H), 0.002 (s, 3H), -0.074 (s, 3H), -0.077 (s, 3H).

・The absolute configuration at C35 (compound **9**)

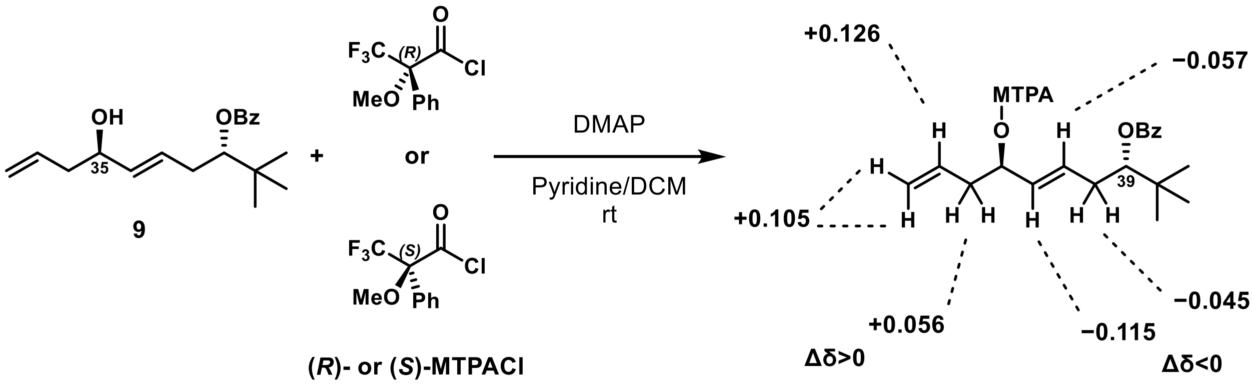

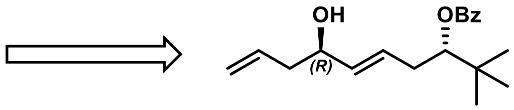

The absolute configuration at C35 was (*R*).

To a solution of alcohol **9** (15.1 mg, 0.05 mmol) in pyridine (200 μL, 0.25 M) was added (*S*)-(+)-α-methoxy-α-(trifluoromethyl)phenylacetyl chloride (MTPACl, 18.9 mg, 0.075 mmol) at room temperature and stirred until alcohol **9** was completely consumed. The reaction mixture was diluted in DCM (1 mL) and quenched with aq. $NaHCO_3$ (1 mL). The phases were separated and the organic layer was washed with aq. HCl (1 M) and brine, dried over $MgSO_4$, filtered and concentrated in vacuo. The residue was purified by column chromatography to give (*R*)-MTPA ester **S26** (25.9 mg, 0.05 mmol) in quantitative yield as a colorless oil. (*S*)-MTPA **S27** ester was prepared from (*R*)-MTPACl by the same method.

(*R*)-MTPA ester **S26**

^{1}H NMR (600 MHz, $CDCl_3$) δ 8.02 (ddd, *J* = 7.8, 1.8, 1.2 Hz, 2H), 7.55 (tt, *J* = 7.8, 1.2 Hz, 1H), 7.48-7.46 (m, 2H), 7.44 (ddd, *J* = 7.8, 7.8, 1.8 Hz, 2H), 7.39-7.35 (m, 3H), 5.84 (ddd, *J* = 15.0, 9.0, 6.0 Hz, 1H), 5.46 (dd, *J* = 15.0, 7.8 Hz, 1H), 5.40-5.33 (m, 2H), 5.02 (dd, *J* = 10.2, 2.4 Hz, 1H), 4.75-4.72 (m, 2H), 3.47 (s, 3H), 2.51 (ddd, *J* = 14.4, 6.0, 2.4 Hz, 1H), 2.31 (ddd, *J* = 14.4, 10.2, 9.0 Hz, 1H), 2.14-2.06 (m, 2H). 1.00 (s, 9H).

(*S*)-MTPA ester **S27**

^{1}H NMR (600 MHz, $CDCl_3$) δ 8.02 (ddd, *J* = 7.8, 1.8, 1.2 Hz, 2H), 7.55 (tt, *J* = 7.8, 1.2 Hz, 1H), 7.48-7.46 (m, 2H), 7.43 (ddd, *J* = 7.8, 7.8, 1.8 Hz, 2H), 7.39-7.35 (m, 3H), 5.78 (ddd, *J* = 14.4, 9.0, 6.0 Hz, 1H), 5.49 (ddt, *J* = 16.8, 10.8, 7.2 Hz, 1H), 5.40-5.32 (m, 2H), 5.00 (dd, *J* = 10.2, 2.4 Hz, 1H), 4.86-4.84 (m, 1H), 4.84-4.82 (m, 1H), 3.48 (s, 3H), 2.48 (ddd, *J* = 14.4, 6.0, 2.4 Hz, 1H), 2.26 (ddd, *J* = 14.4, 10.2, 9.0 Hz, 1H), 2.20-2.11 (m, 2H), 0.99 (s, 9H).

5. Procedure of Recycle of Brønsted Acid Catalysts in Asymmetric Allylborations

・Phosphoric acid catalyst (*R*)-**2**

First cycle

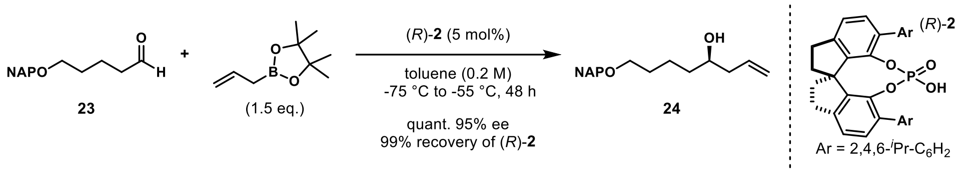

To a solution of aldehyde **23** (1.45 g, 6.0 mmol, 1.0 eq.) and (*R*)-**2** (216 mg, 0.3 mmol, 5 mol%) in toluene (12 mL) was added allylboronicacid pinacolester (1.51 g, 9 mmol, 1.5 eq.) in toluene (8.0 mL) dropwise at -75 °C under an Ar atmosphere. The reaction mixture was stirred for 24 h at this temperature and stirred for an additional 24 h at -55 °C. *N*-methyl-2-amino-ethanol (575 µL, 7.2 mmol, 1.2 eq.) was added to the mixture. The resulting suspension was stirred for 1 h at ambient temperature. The reaction was diluted with hexane (12 mL) and filtered through a pad of celite. The remaining white solid and celite cake was rinsed with hexane (2 x 12 mL). The filtrate was washed with 1N HCl then brine, dried over Na_2SO_4, filtered and concentrated under reduced pressure. The residue was purified by flash column chromatography on silica gel (Hexane/EtOAc = 20/1 to 4/1) to obtain homoallylic alcohol **24** (1.69 g, 5.94 mmol) in 99% yield, 95% ee as a colorless oil. (*R*)-**2** was recovered as a salt by eluting Hexane/EtOAc = 2/1. The recovered salt was dissolved in DCM (10 mL), washed by 6N HCl (2 x 10 mL) and dried under high-vacuum to recover (*R*)-**2** (213 mg, 0.292 mmol) in 98% yield as a white solid.

Second cycle

To a solution of aldehyde **23** (1.45 g, 6.0 mmol, 1.0 eq.) and recovered (*R*)-**2** (213 mg, 0.3 mmol, 5 mol%) in toluene (12 mL) was added allylboronicacid pinacolester (1.51 g, 9 mmol, 1.5 eq.) in toluene (8.0 mL) dropwise at -75 °C under an Ar atmosphere. The reaction mixture was stirred for 24 h at this temperature and stirred for an additional 24 h at -55 °C. *N*-methyl-2-amino-ethanol (575 µL, 7.2 mmol, 1.2 eq.) was added to the mixture. The resulting suspension was stirred for 1 h at ambient temperature. The reaction was diluted with hexane (12 mL) and filtered through a pad of celite. The remaining white solid and celite cake was rinsed with hexane (2 x 12 mL). The filtrate was washed with 1N HCl then brine, dried over Na_2SO_4, filtered and concentrated under reduced pressure. The residue was purified by flash column chromatography on silica gel (Hexane/EtOAc = 20/1 to 4/1) to obtain homoallylic alcohol **24** (1.69 g, 5.94 mmol) in 99% yield, 95% ee as a colorless oil. (*R*)-**2** was recovered as a salt by eluting Hexane/EtOAc = 2/1. The recovered salt was dissolved in DCM (10 mL), washed by 6N HCl (2 x 10 mL) and dried under high-vacuum to recover (*R*)-**2** (210 mg, 0.292 mmol) in 97% yield as a white solid. The ee value was determined by chiral stationary phase HPLC analysis of **24** (Chiralpack OD-3 column (Hexane/iPrOH = 98/2, 1.0 mL/min, 40 °C, 254 nm), tR major = 21.6 min, tR minor = 23.2 min; 95% ee.

HPLC chart of compound **S12**

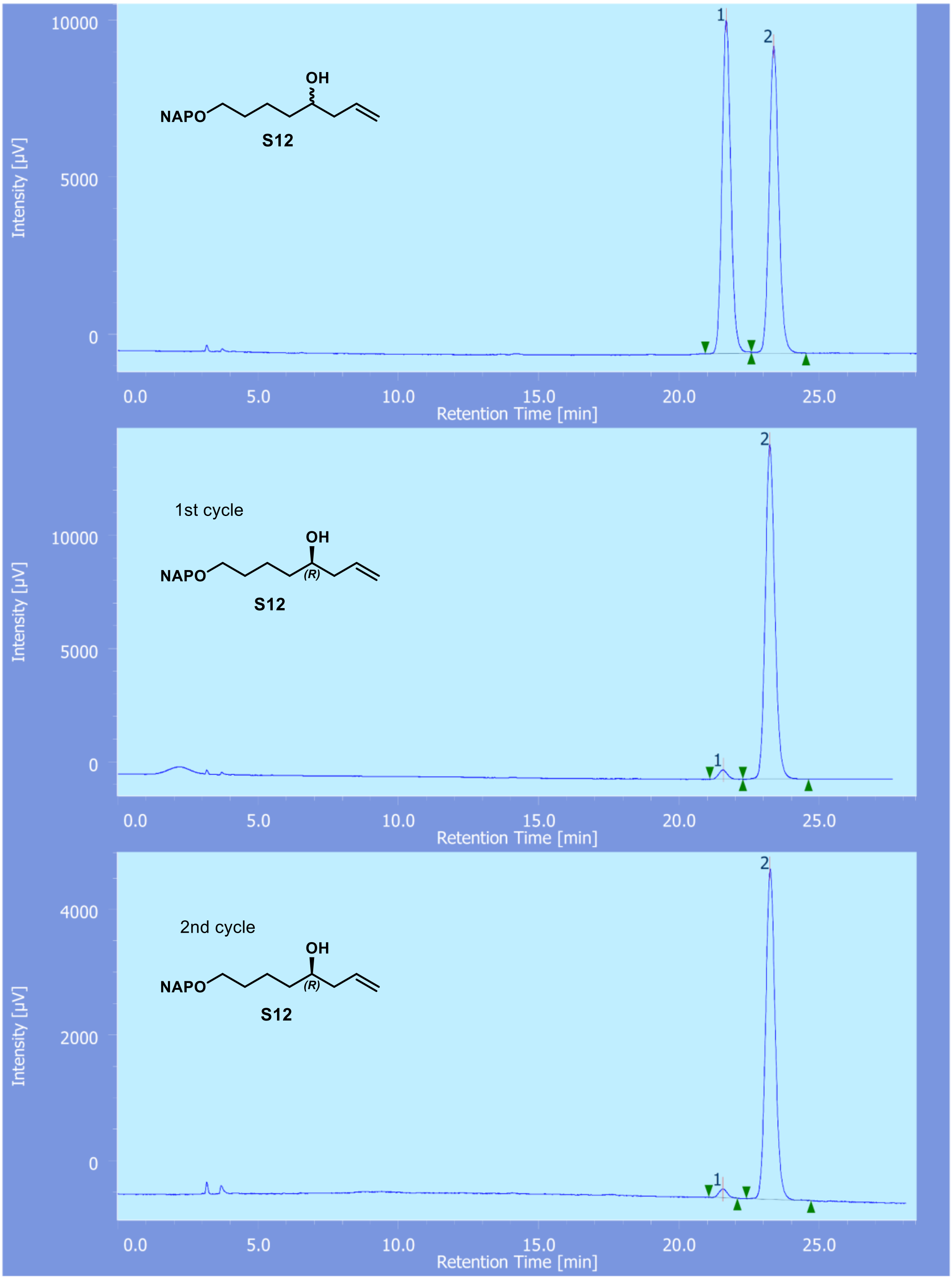

	Retention time (1)	Retention time (2)	% area (1)	% area (2)
rac-**S12**	21.667	23.358	49.960	50.040
(*R*)-**S12** (1st cycle)	21.558	23.217	2.437	97.563
(*R*)-**S12** (2nd cycle)	21.550	23.233	2.341	97.659

・Phosphoramide catalyst (*S*)-**3**

First cycle

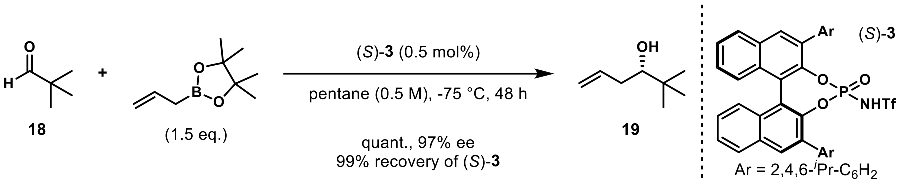

To a solution of (*S*)-**3** (44.2 mg, 0.05 mmol, 0.5 mol%) and pivalaldehyde (**18**) (990 mg, 10 mmol, 1.0 eq.) in pentane (6.0 mL) was added allylboronic acid pinacol ester (2.83 mL, 15 mmol, 1.5 eq.) in pentane (4.0 mL) at -75 °C under Ar atmosphere. The reaction was stirred for 48 h at this temperature and allowed to warm to room temperature. The resulting pink solution was added *N*-methyl-2-amino-ethanol (0.96 mL, 12 mmol, 1.2 eq.) dropwise (white precipitation was immediately formed). The resulting white suspension was stirred for additional 1 h. The reaction mixture was then filtered through a pad of celite and the celite cake was rinsed with pentane (3 x 10 mL). The filtrate was then washed with 1 N HCl (30 mL) and brine (30 mL), dried over Na_2SO_4, filtered and carefully concentrated under reduced pressure (>300 mmHg, <25 °C). The crude material was purified by flush column chromatography (pentane/Et2O = 40:1 to 20:1) to obtain secondary alcohol **19** (1.28 g, 10 mmol) in quantitative yield with 97% ee. Phosphoramide (*S*)-**3** was also recovered as a salt by flush column chromatography (Hexane/EtOAc = 5/1 to 4/1). The recovered salt of (*S*)-**3** was protonated by washing DCM solution of this salt (5 mL) with 6N HCl (5 mL) and dried under high vacuum to recover (*S*)-**3** (43.6 mg, 0.0495 mmol) in 99% as a white solid.

Second cycle

To a solution of recovered (*S*)-**3** (43.6 mg, 0.05 mmol) and pivalaldehyde **18** (990 mg, 10 mmol, 1.0 eq.) in pentane (6.0 mL) was added allylboronic acid pinacol ester (2.83 mL, 15 mmol, 1.5 eq.) in pentane (4.0 mL) at -75 °C under Ar atmosphere. The reaction was stirred for 48 h at this temperature and allowed to warm to room temperature. The resulting pink solution was added N-methyl-2-amino-ethanol (0.96 mL, 12 mmol, 1.2 eq.) dropwise (white precipitation was immediately formed). The resulting white suspension was stirred for additional 1 h. The reaction mixture was then filtered through a pad of celite and the celite cake was rinsed with pentane (3 x 10 mL). The filtrate was then washed with 1 N HCl (30 mL) and brine (30 mL), dried over Na_2SO_4, filtered and carefully concentrated under reduced pressure (>300 mmHg, <25 °C). The crude material was purified by flush column chromatography (pentane/Et_2O = 40:1 to 20:1) to obtain secondary alcohol **19** (1.28 g, 10 mmol) in quantitative yield with 97% ee. Phosphoramide (*S*)-**3** was also recovered as a salt by flush column chromatography (Hexane/EtOAc = 5/1 to 4/1). The recovered salt of (*S*)-**3** was protonated by washing DCM solution of this salt (5 mL) with 6N HCl (5 mL) and dried under high vacuum to recover (*S*)-**3** (43.5 mg, 0.0495 mmol) in >99% as a white solid.

Determination of ee value of alcohol **19**

The ee value was determined by chiral stationary phase HPLC analysis of primary alcohol derivative **S28** (Chiralpack AD-3 column (Hexane/*i*PrOH = 97/3, 1.0 mL/min, 40 °C, 254 nm), tR major = 16.1 min, tR minor = 17.4 min; 97% ee. Compound **S28** was prepared according to the following scheme.

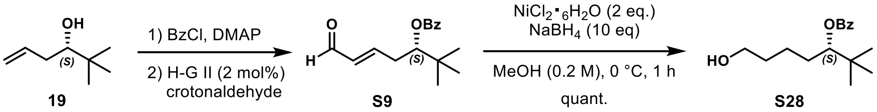

HPLC chart of compound **S28**

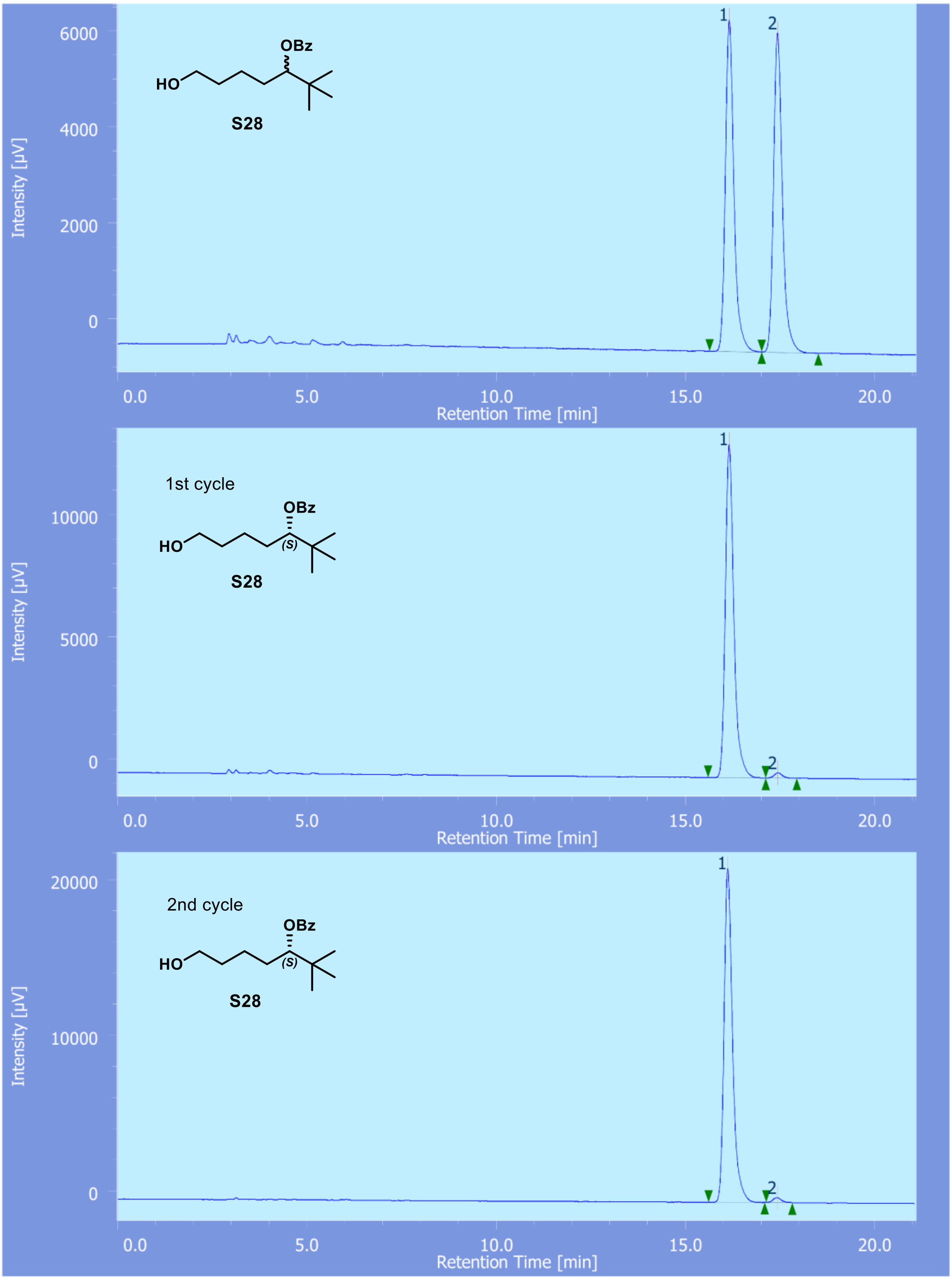

	Retention time (1)	Retention time (2)	% area (1)	% area (2)
rac-**S28**	16.150	17.425	50.150	49.850
(*S*)-**S28** (1st cycle)	16.142	17.433	98.548	1.452
(*S*)-**S28** (2nd cycle)	16.108	17.408	98.600	1.400

6. Investigation of macrolactonization conditions

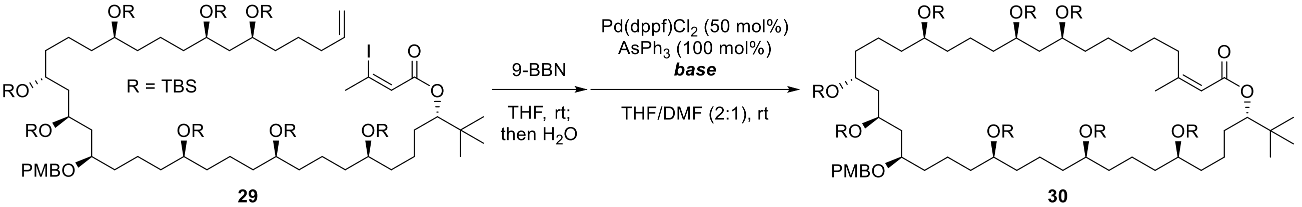

29 **30**

Table S1. Screening of intramolecular Suzuki-Miyaura Coupling reaction conditions

				yield /%[a]				
entry	base	temp. / °C	time / h	**30 (desired)**	**29 (S.M.)**	**A1**	**A2**	**A3**
1	Cs_2CO_3 (10 eq.)	25	12	61	n.d.	20	12	5
2	K_3PO_4 (10 eq.)	60	30	trace	5	trace	50	trace
3	Ag_2O (1.5 eq.)	60	30	n.d.	10	trace	28	trace
4	Cs_2CO_3 (10 eq.) Ag_2O (1.5 eq.)	28	18	35	n.d.	15	25	17
5[b]	Cs_2CO_3 (10 eq.)	25	16	40	n.d.	15	15	8
6[c]	Cs_2CO_3 (10 eq.)	25	16	50	n.d.	15	17	12
7[d]	Cs_2CO_3 (10 eq.)	70	12	11	n.d.	52	6	n.d

[a]Isolated yield. [b]Pd(dppf)Cl_2 (20 mol%) and $AsPh_3$ (40 mol%) were used. [c]The reaction was performed without $AsPh_3$. [d]Aggarwal's condition. n.d. = not determined

side products

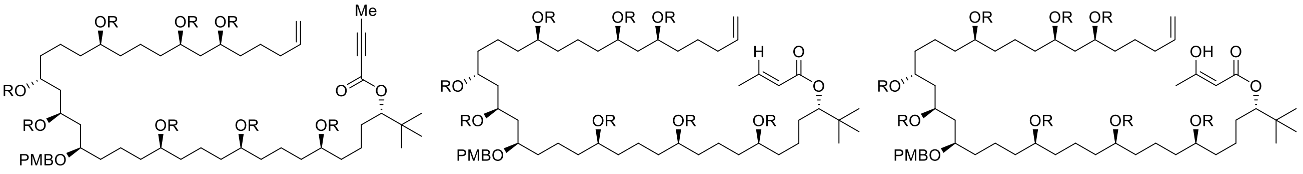

A1 (E2 elimination) **A2** **A3**

Another synthetic route

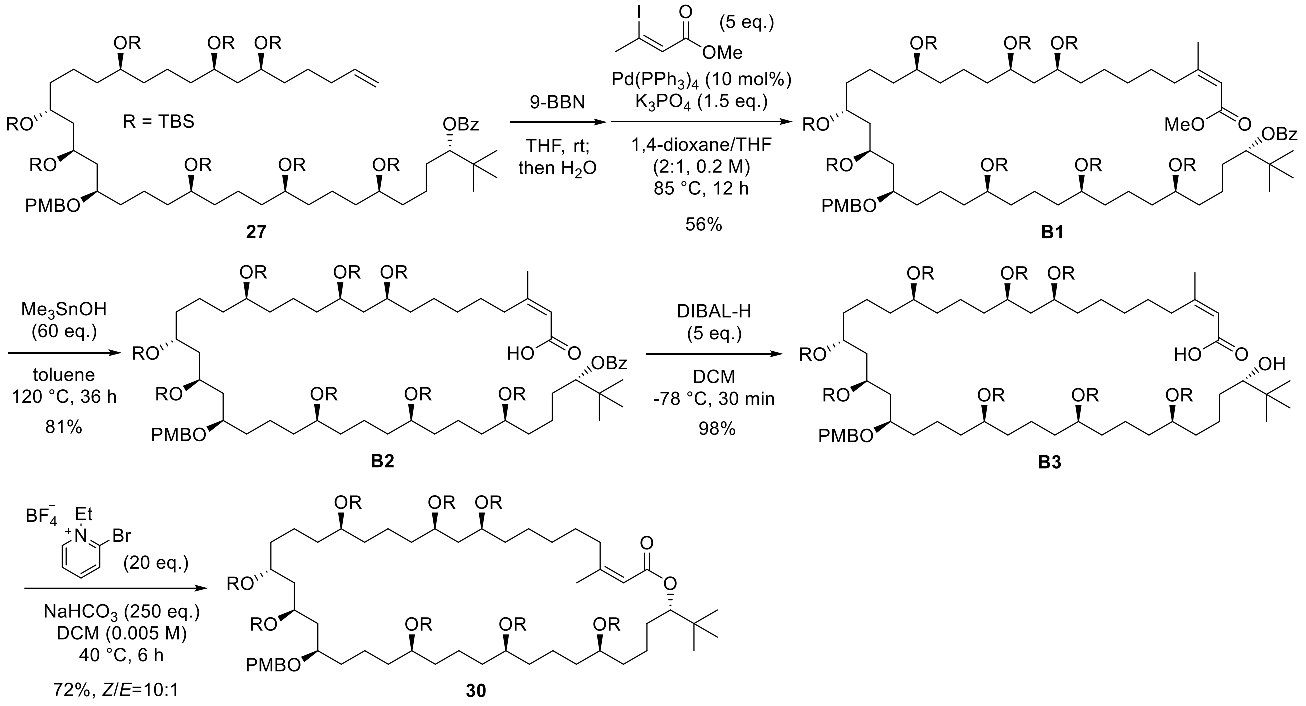

Note: This route utilized macrolactonization with Mukaiyama's reagent[8]. The main problems were increasing number of steps and the need for an excessive amount of tin reagent (Me_3SnOH). Thus, we finally adopted a route using Suzuki-Miyaura coupling.

7. References

[1] S. Umemiya, N. Shinagawa, M. Terada, *Org. Lett.* **2023**, *25*, 1924–1928.

[2] R. S. Narayan, B. Borhan *J. Org. Chem.* **2006**, *71*, 1416–1429.

[3] S. E. Denmark, W.-J. Chung, *J. Org. Chem.* **2008**, *73*, 4582–4595.

[4] S. Umemiya, S. Osaka, N. Shinagawa, T. Hirata, M. Terada, *Chem. Sci.* **2025**, *16*, 3865–3871.

[5] H. S. Overkleeft *et al.*, *J. Med. Chem.* **2014**, *57*, 9096−9104.

[6] V. K. Aggarwal *et al.*, *Angew. Chem. Int. Ed.* **2023**, *62*, e202312054.

[7] H. Tran, P. McGee, L. Barriault, *Chem. Eur. J.* **2023**, *29*, e202301640.

[8] a) T. Mukaiyama, M. Usui, K. Saigo, *Chem. Lett.* **1976**, *5*, 49–50; b) D. A. Evans, J. T. Starr, *J. Am. Chem. Soc.* **2003**, *125*,13531–13540.

8. NMR charts

^{1}H NMR (600 MHz, $CDCl_3$) and ^{13}C NMR (151 MHz, $CDCl_3$) spectra of **12**

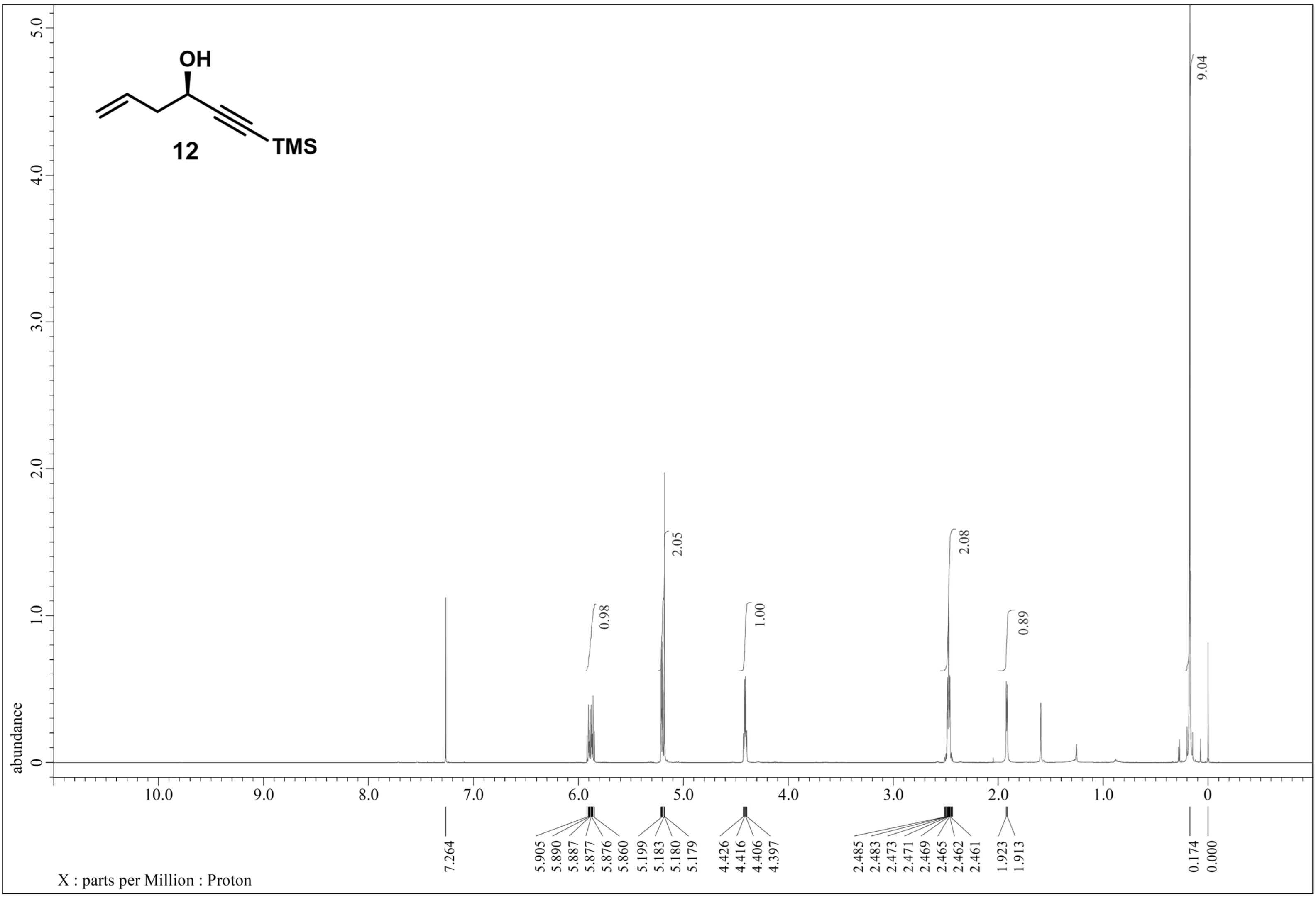

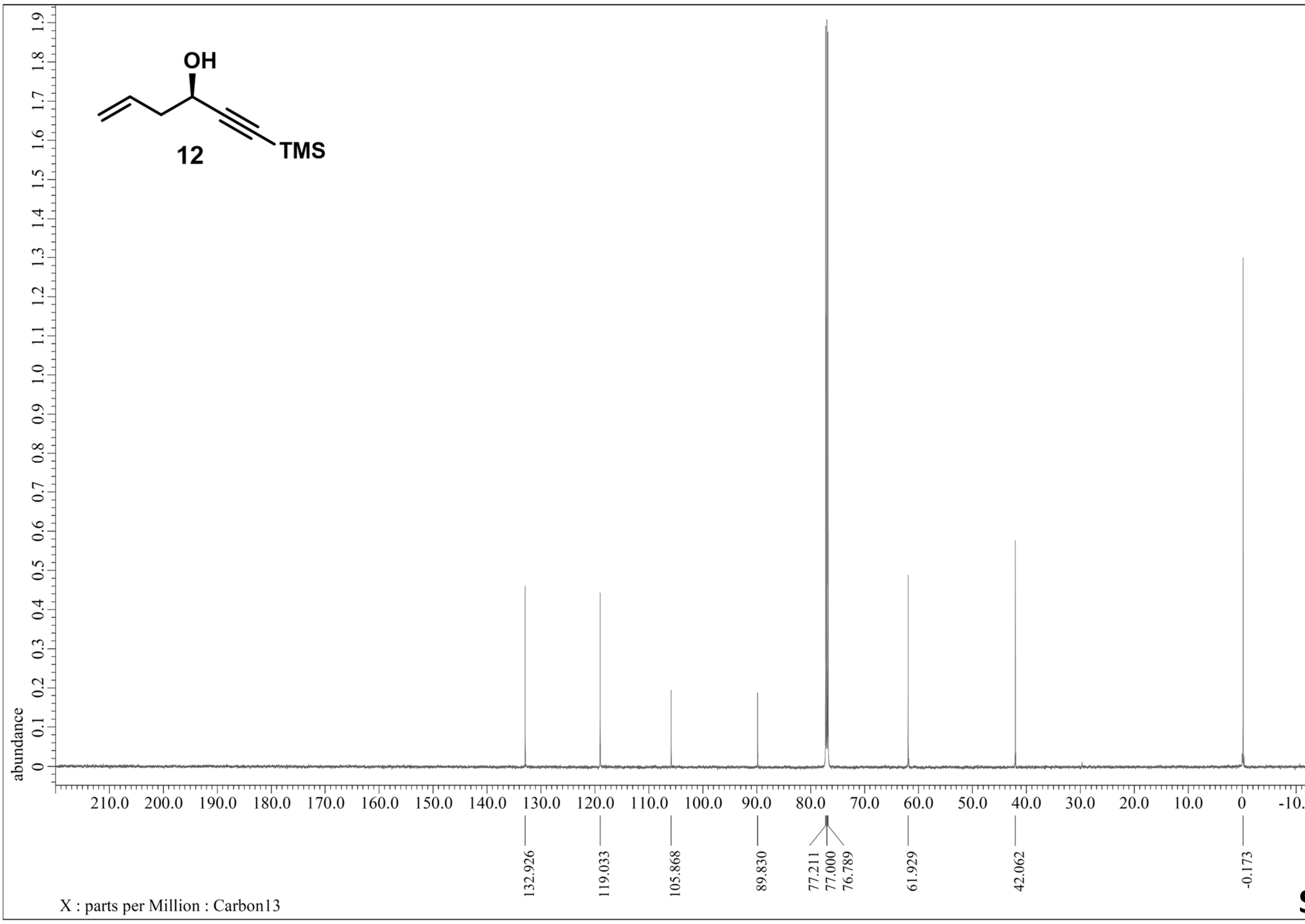

^{1}H NMR (600 MHz, $CDCl_3$) and ^{13}C NMR (151 MHz, $CDCl_3$) spectra of **13**

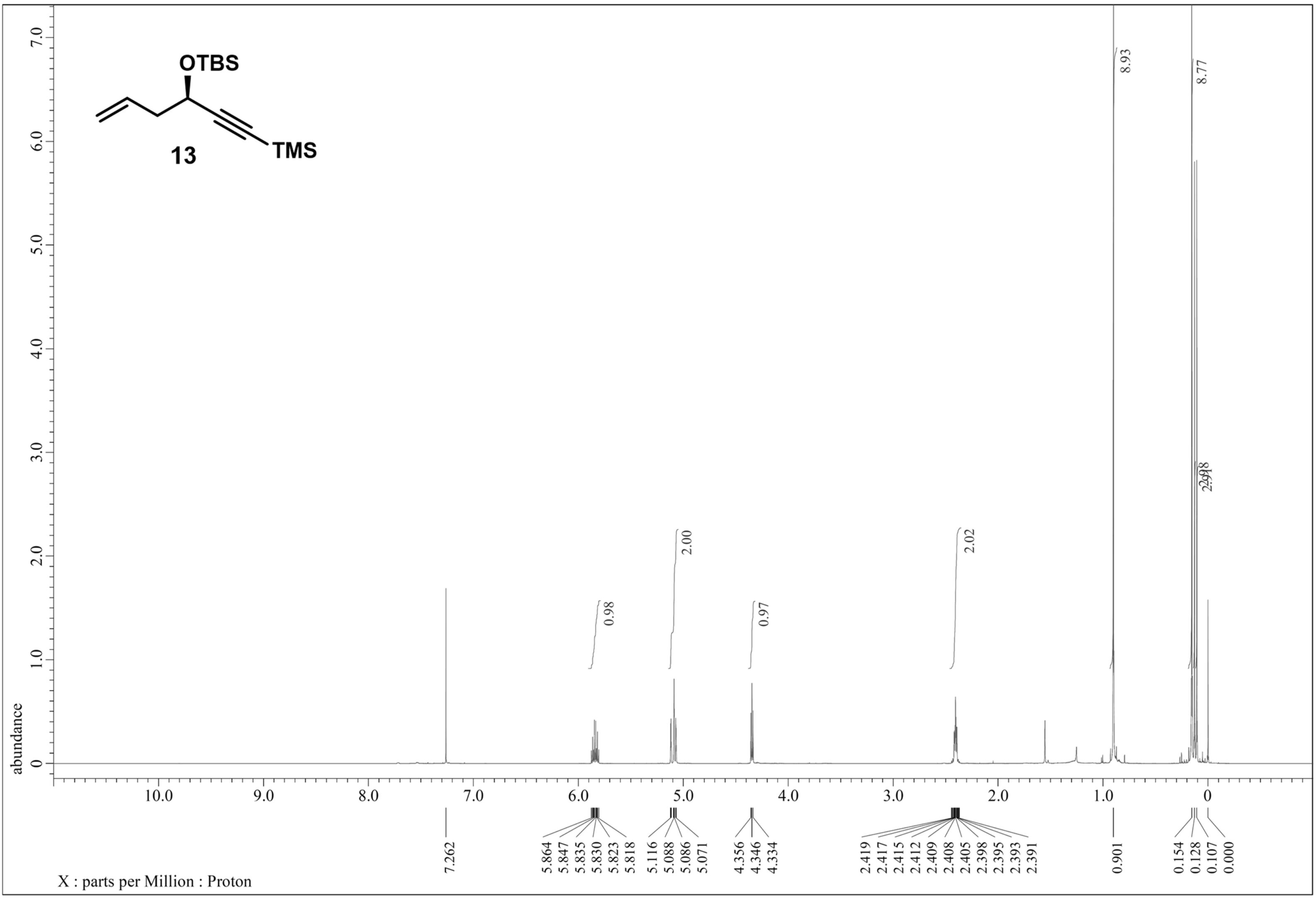

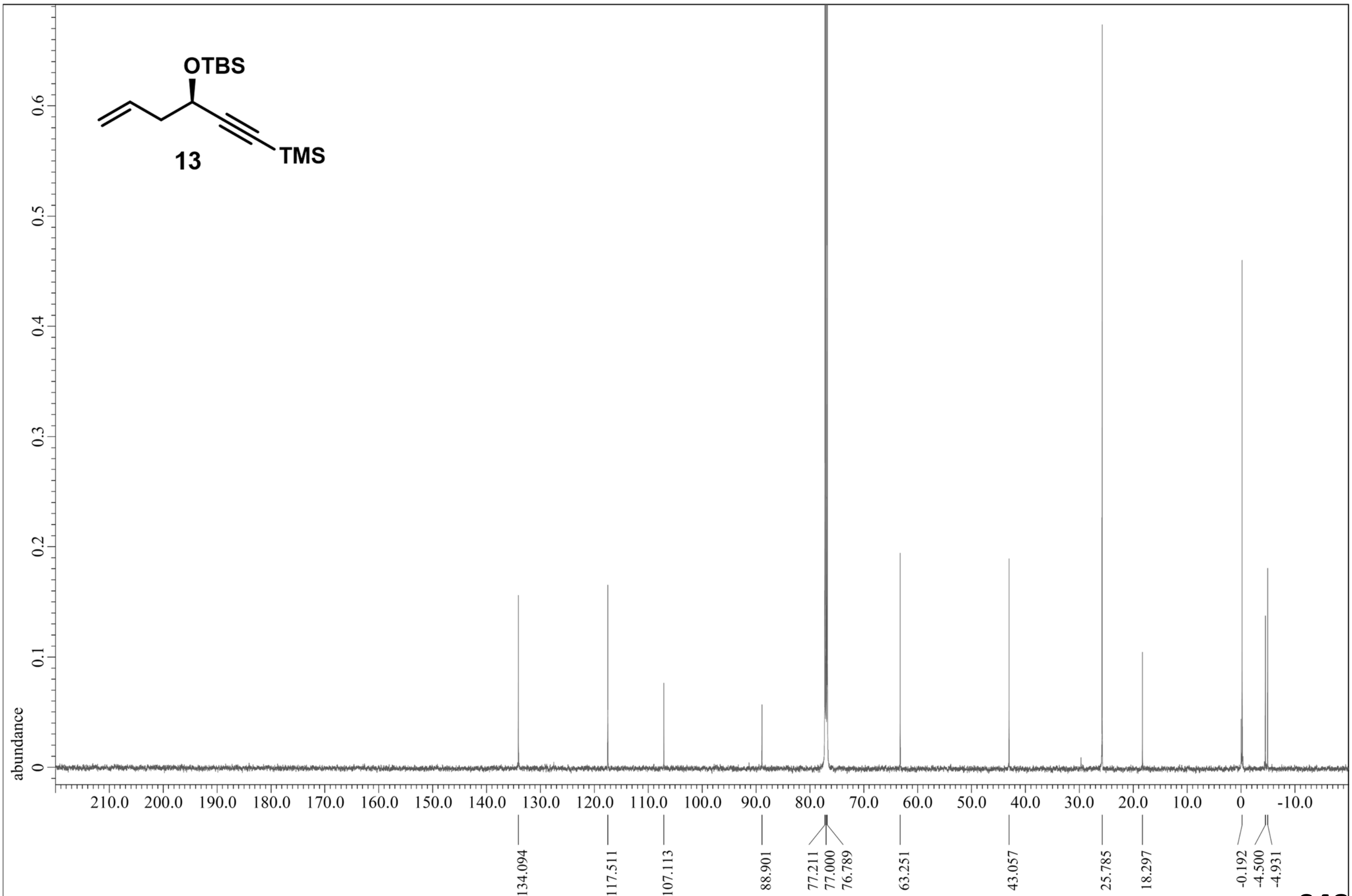

^{1}H NMR (600 MHz, $CDCl_3$) and ^{13}C NMR (151 MHz, $CDCl_3$) spectra of **S1**

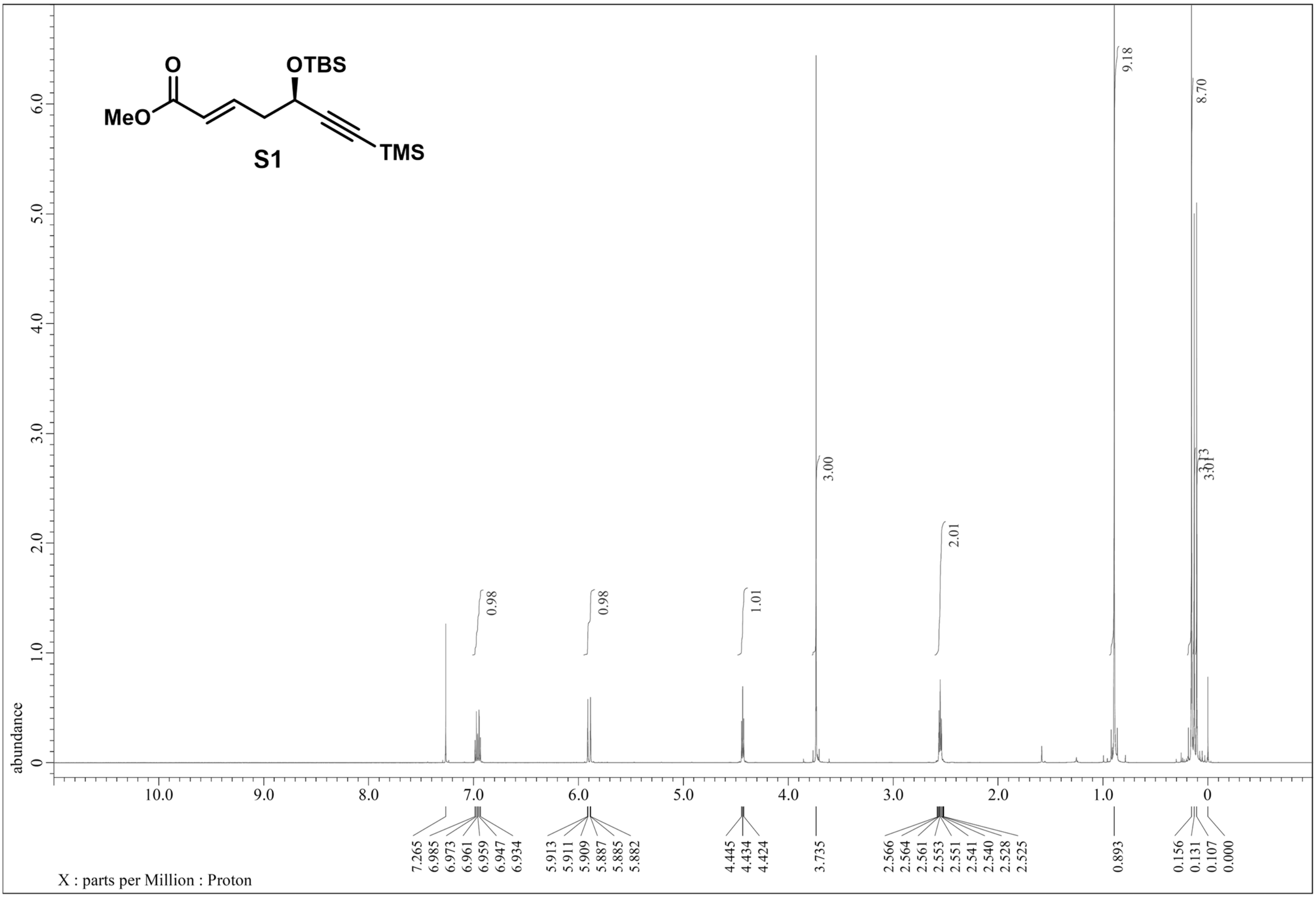

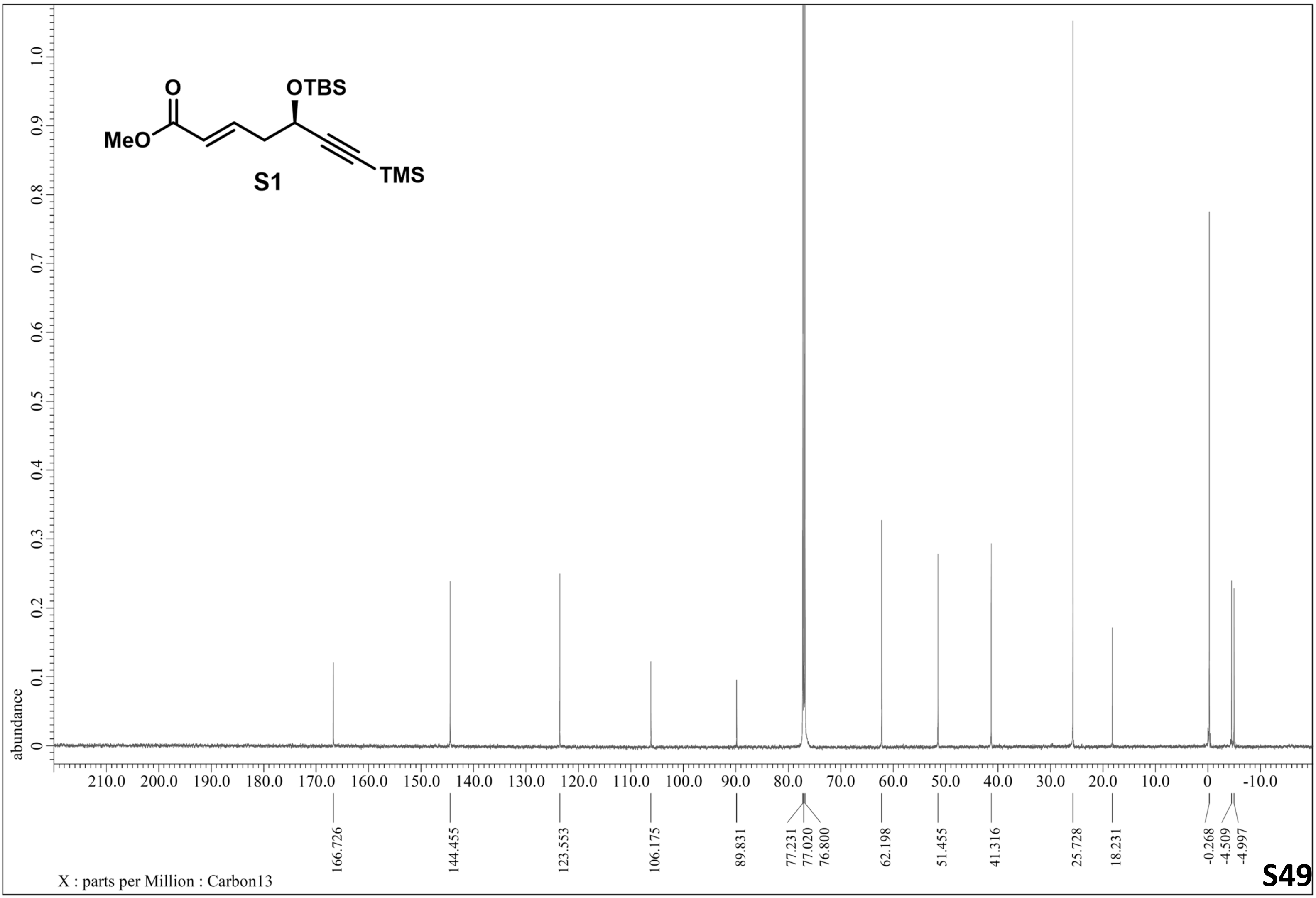

^{1}H NMR (600 MHz, $CDCl_3$) and ^{13}C NMR (151 MHz, $CDCl_3$) spectra of **S2**

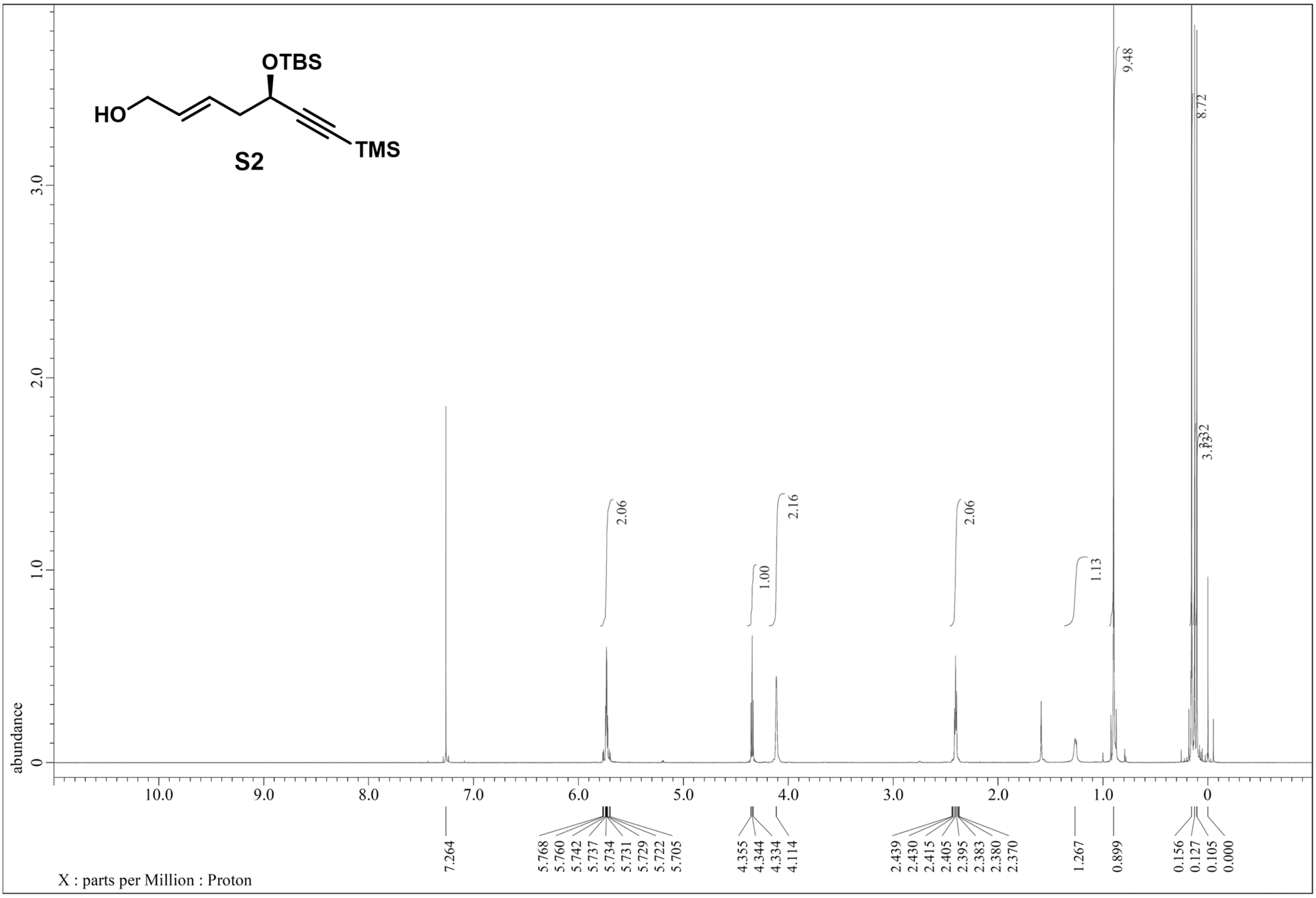

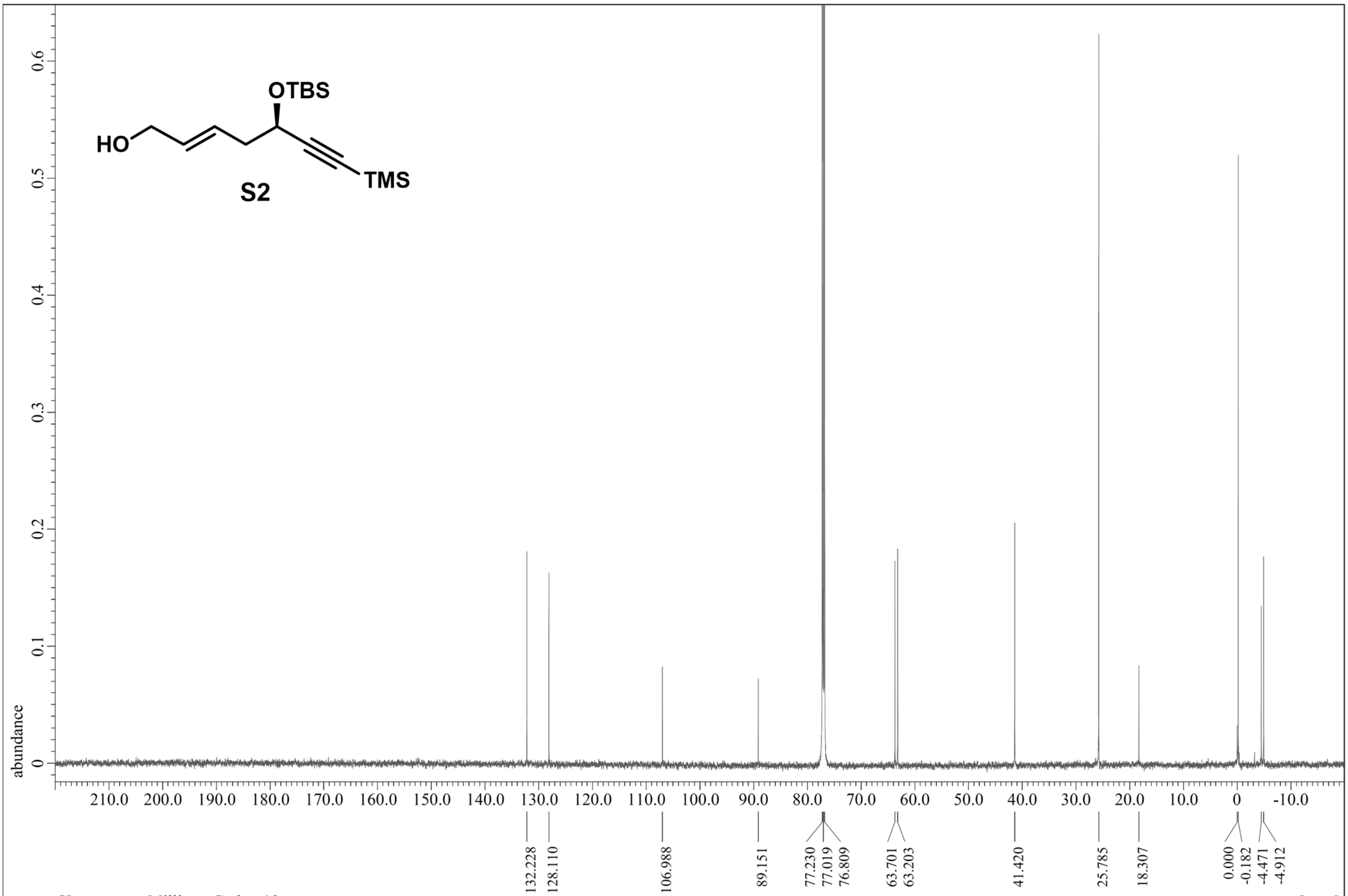

^{1}H NMR (600 MHz, $CDCl_3$) and ^{13}C NMR (151 MHz, $CDCl_3$) spectra of **6**

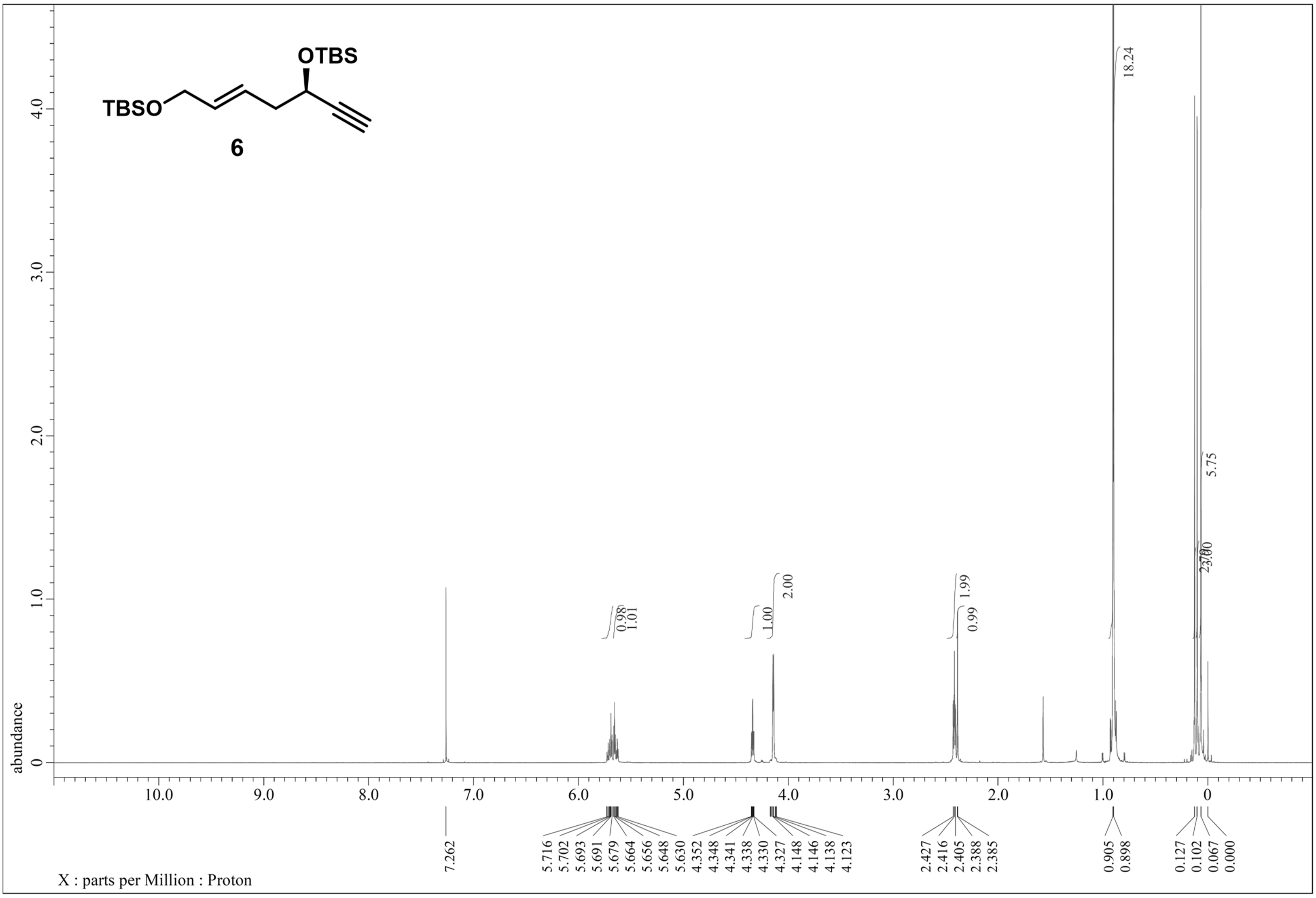

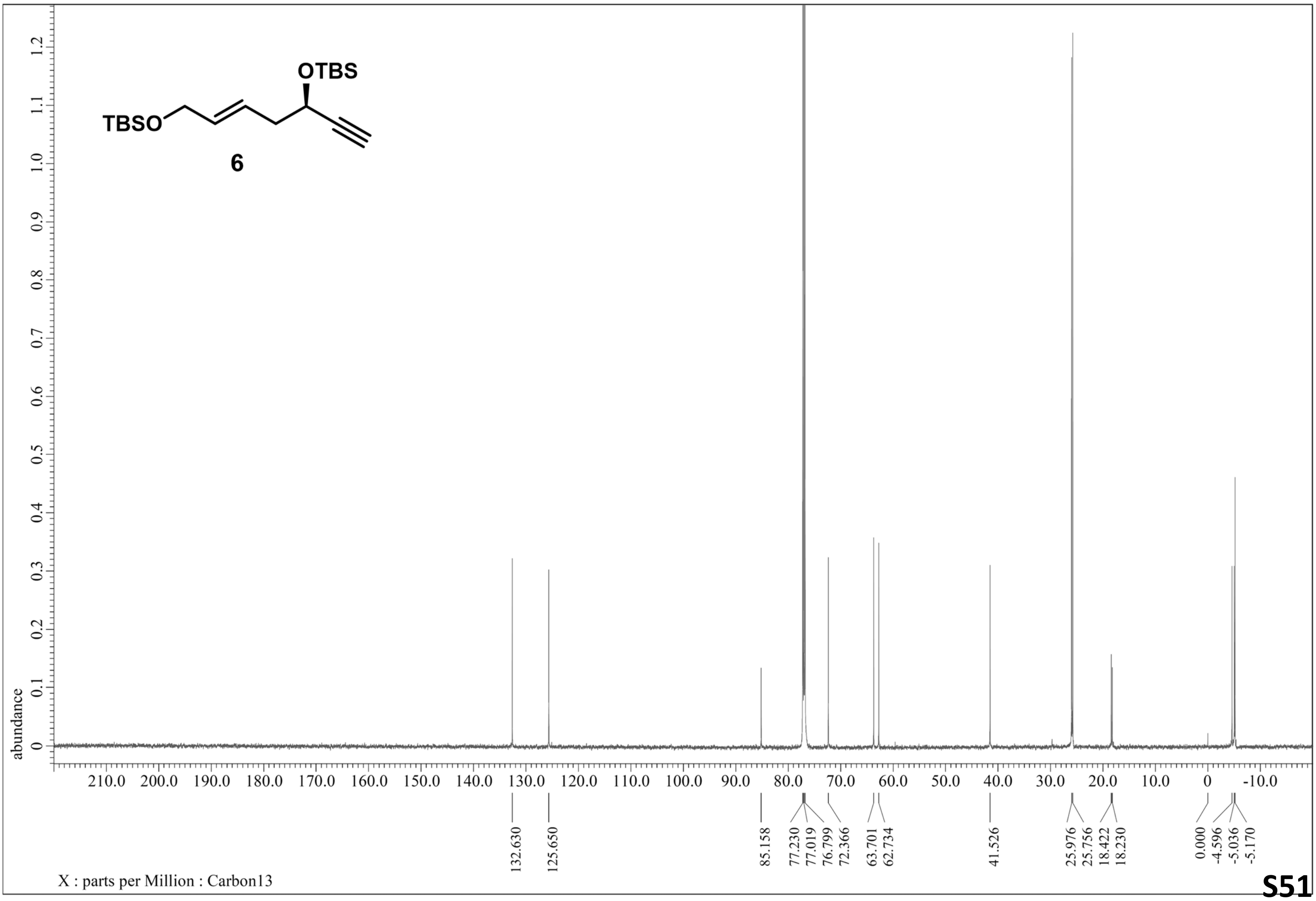

^{1}H NMR (600 MHz, $CDCl_3$) and ^{13}C NMR (151 MHz, $CDCl_3$) spectra of **16**

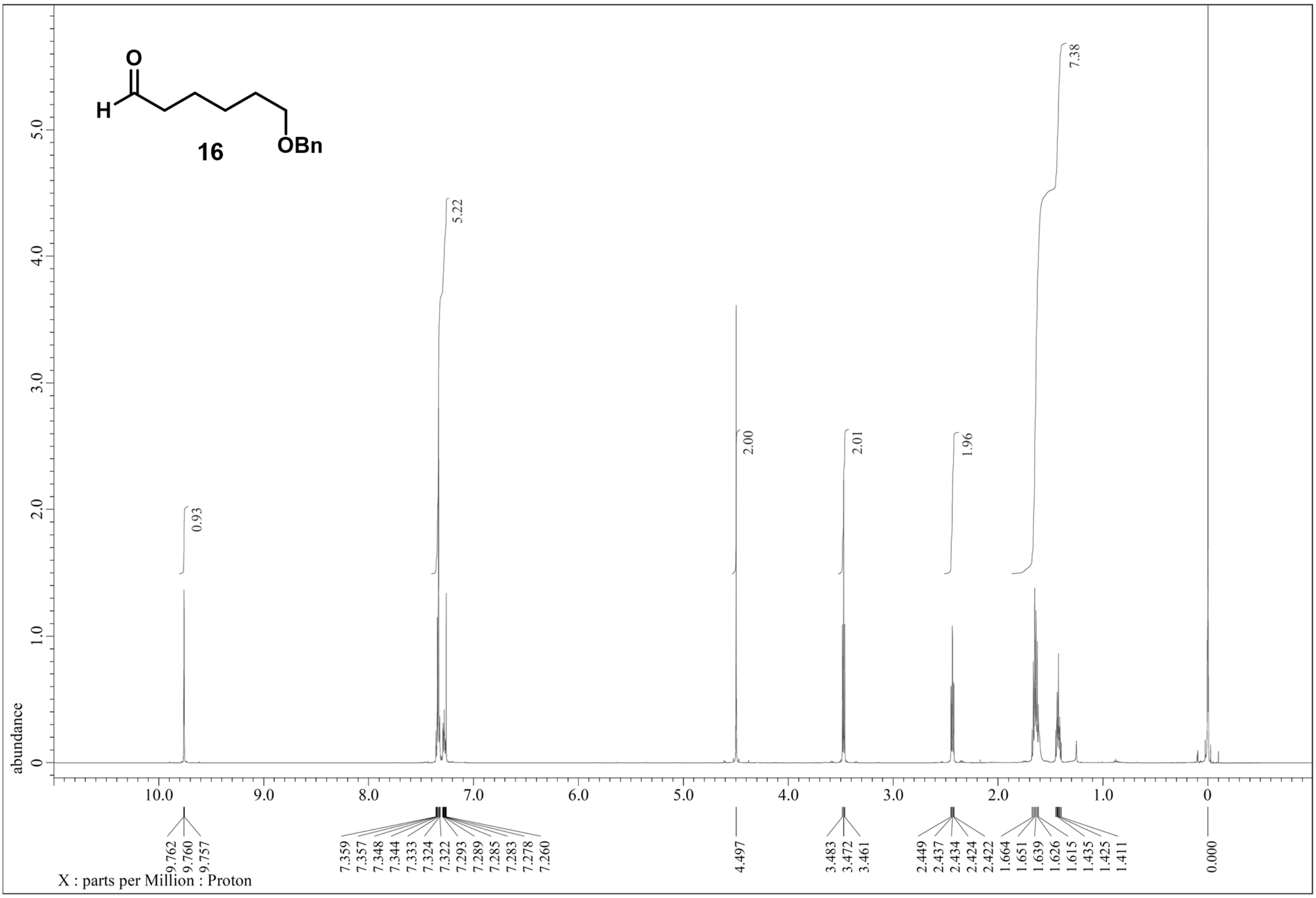

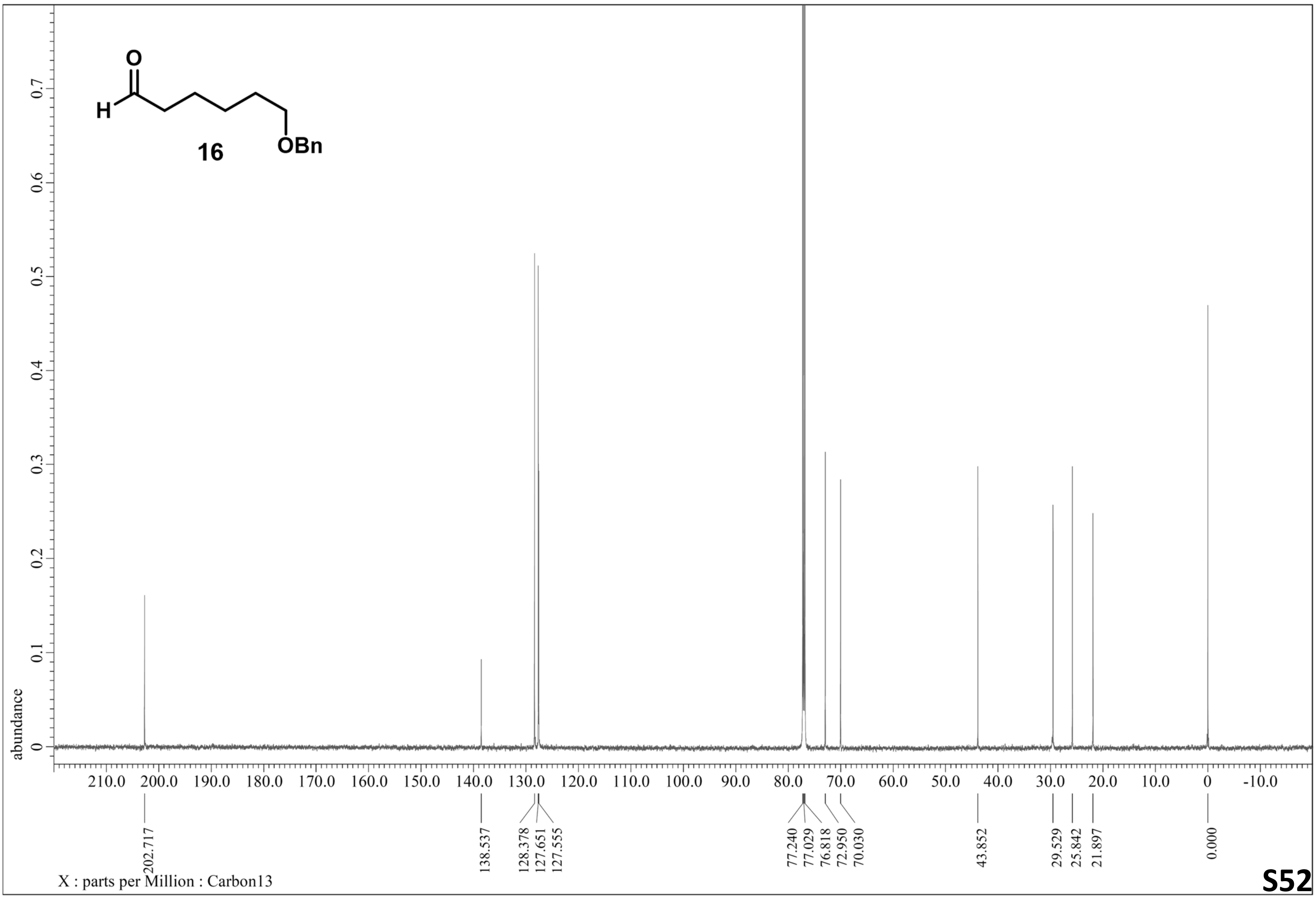

^1H NMR (600 MHz, $CDCl_3$) and ^{13}C NMR (151 MHz, $CDCl_3$) spectra of **17**

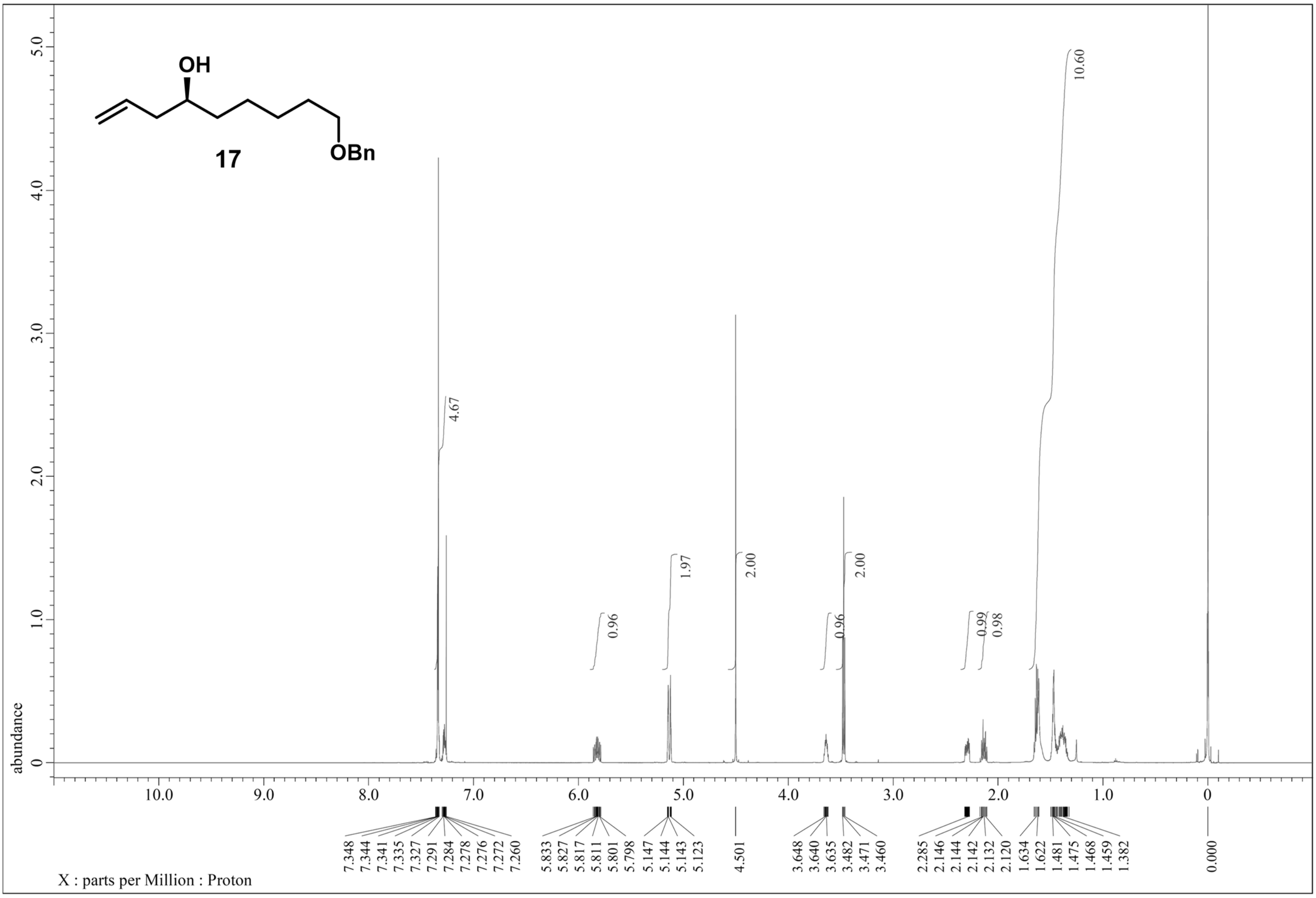

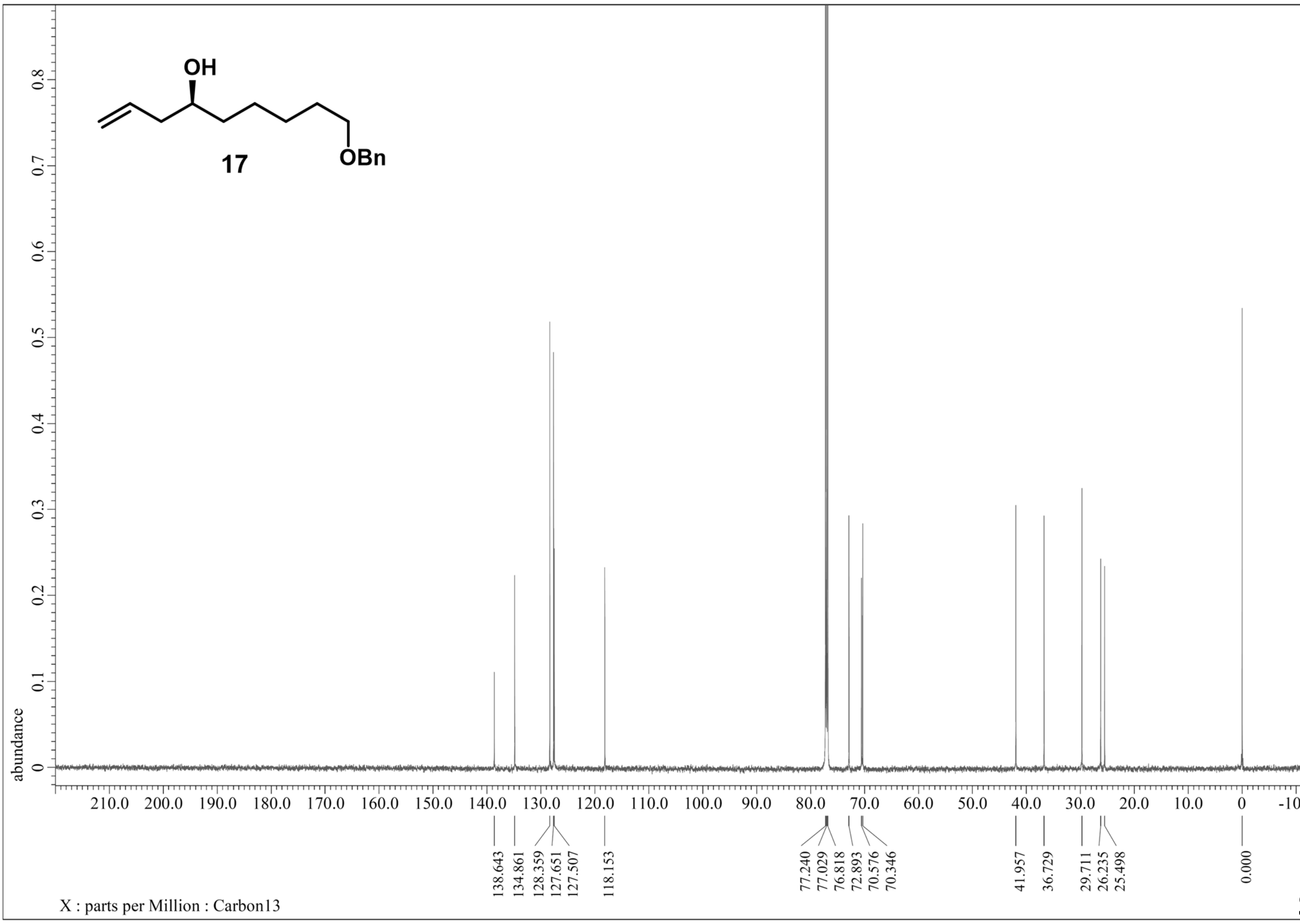

^1H NMR (600 MHz, $CDCl_3$) and ^{13}C NMR (151 MHz, $CDCl_3$) spectra of **S3**

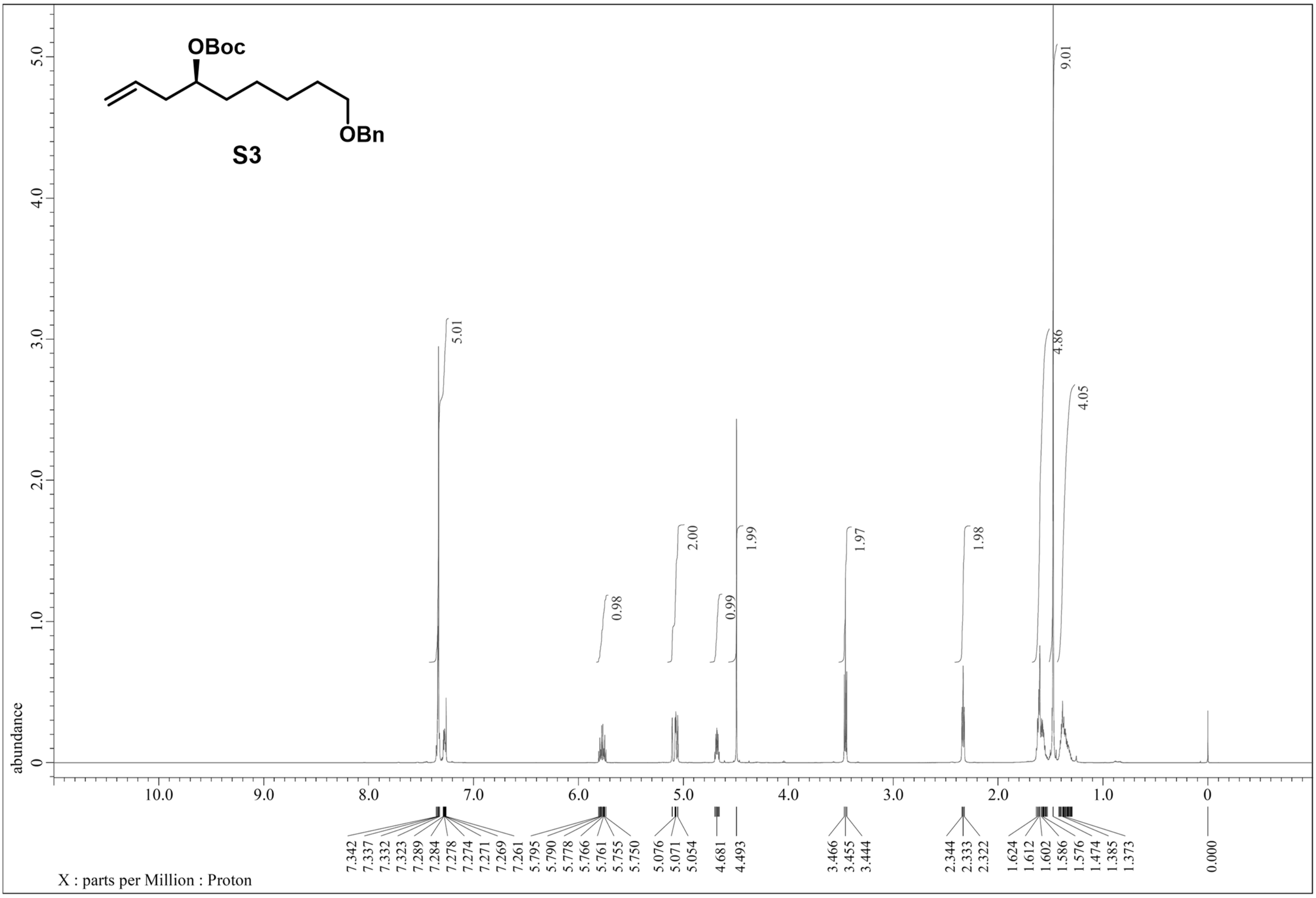

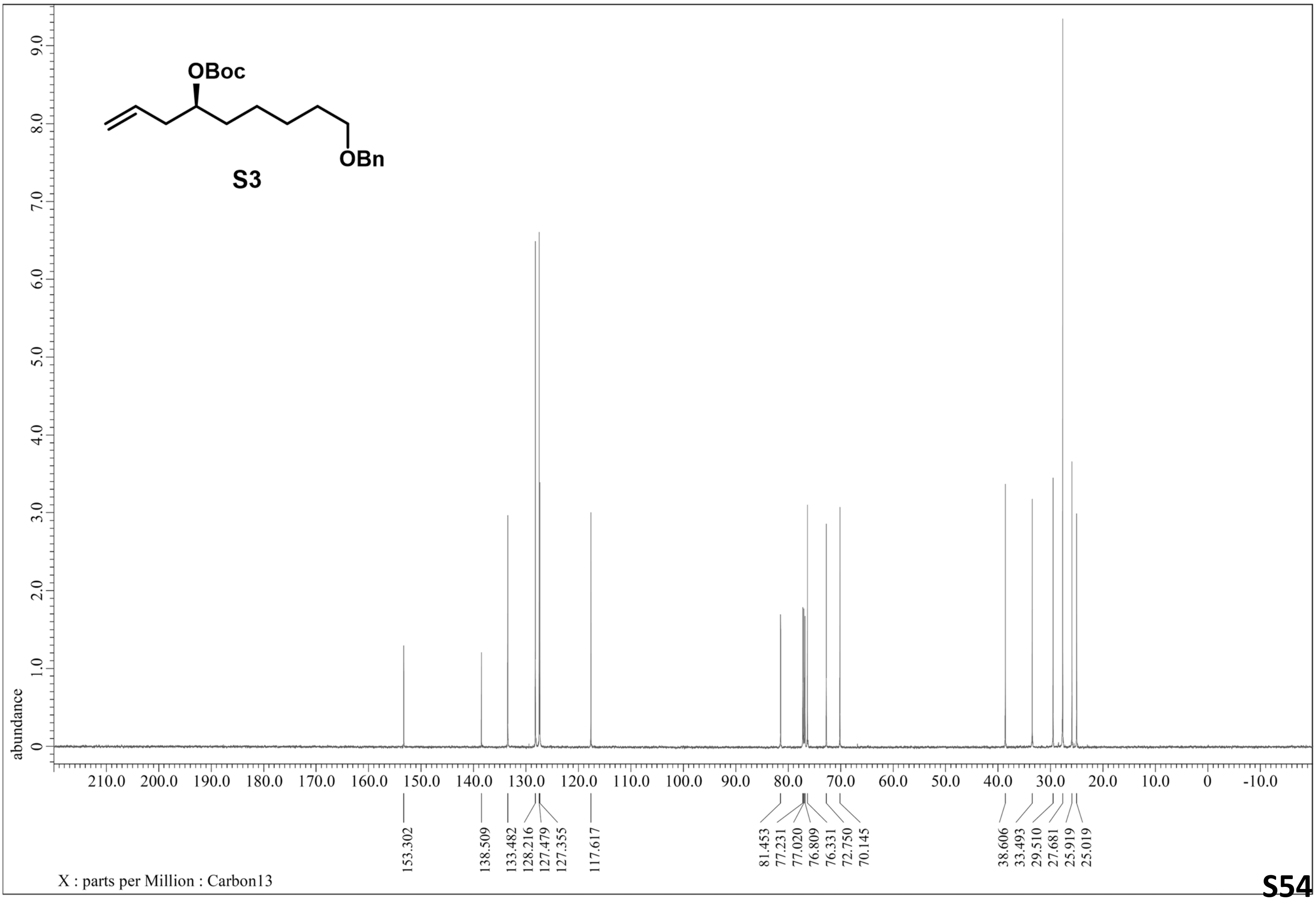

^{1}H NMR (600 MHz, $CDCl_3$) and ^{13}C NMR (151 MHz, $CDCl_3$) spectra of **S4**

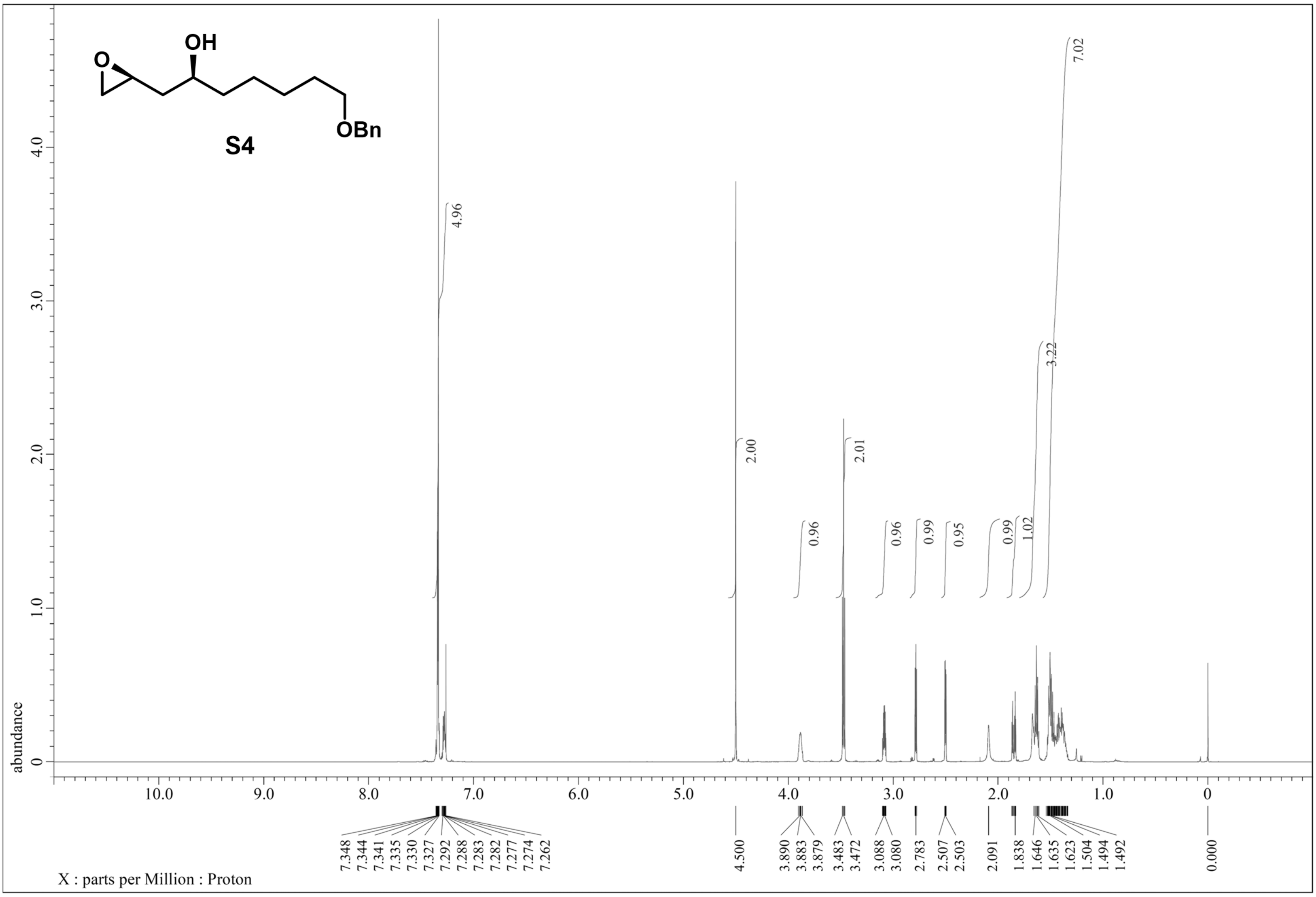

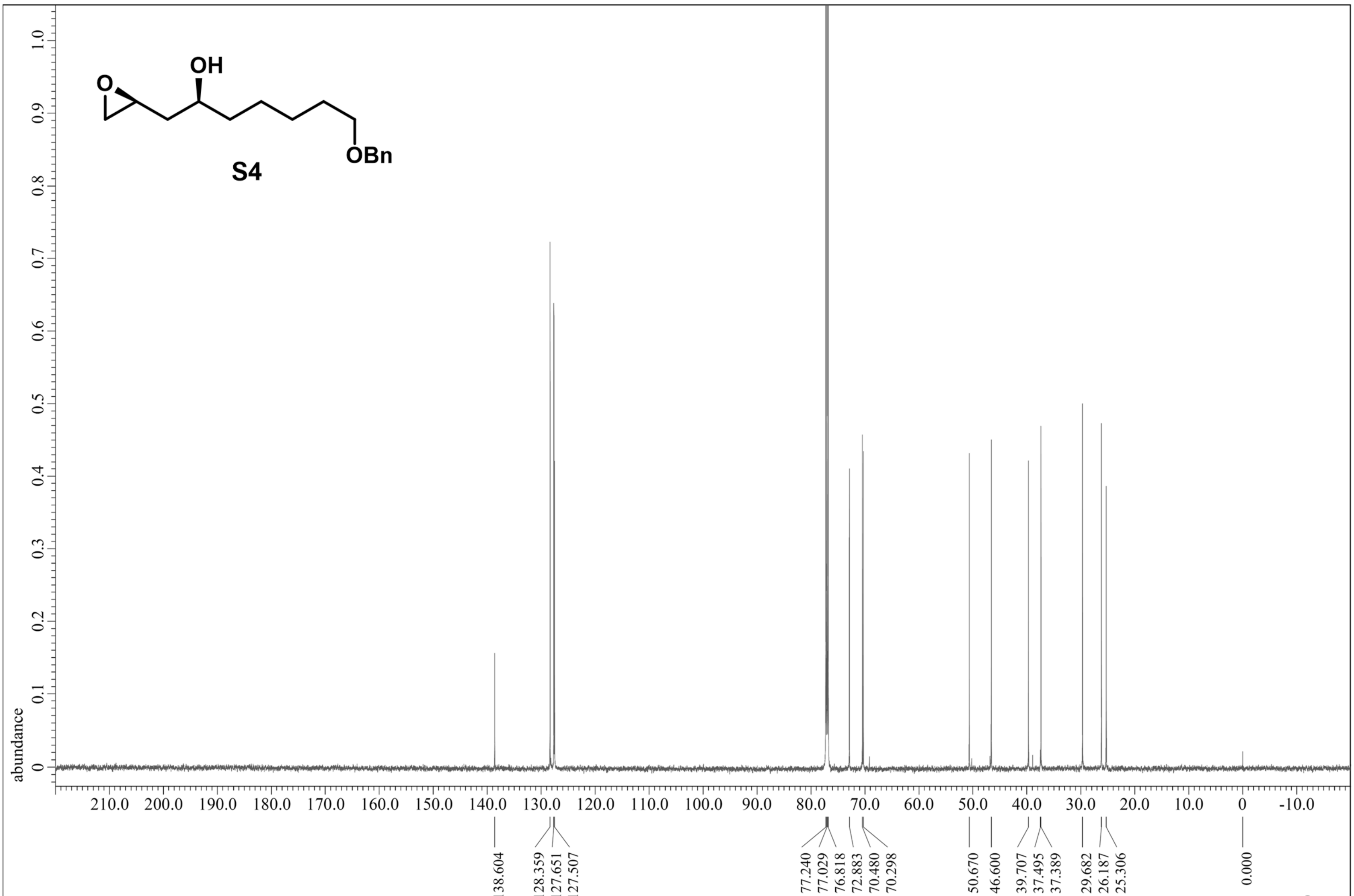

^{1}H NMR (600 MHz, $CDCl_3$) and ^{13}C NMR (151 MHz, $CDCl_3$) spectra of **7**

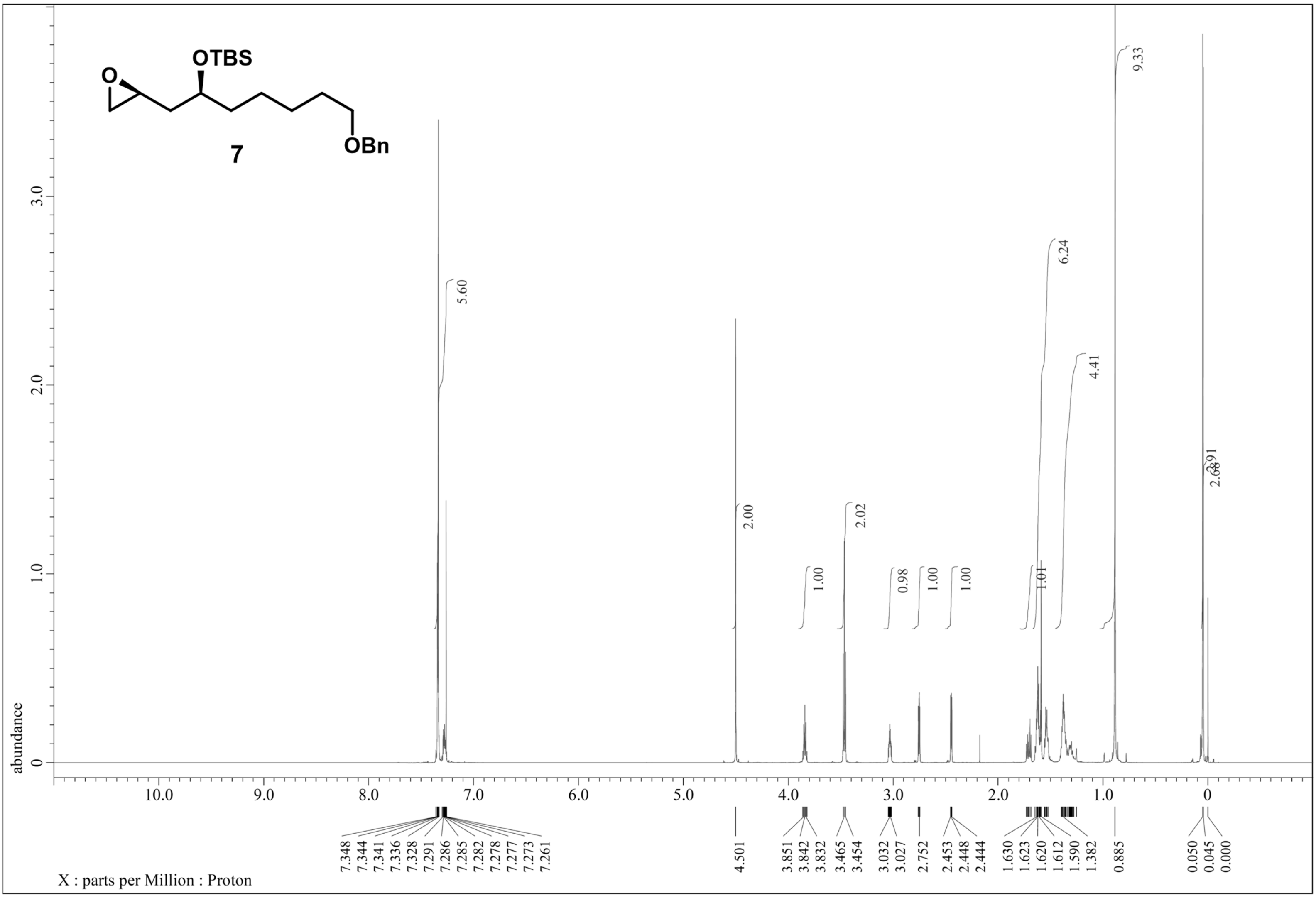

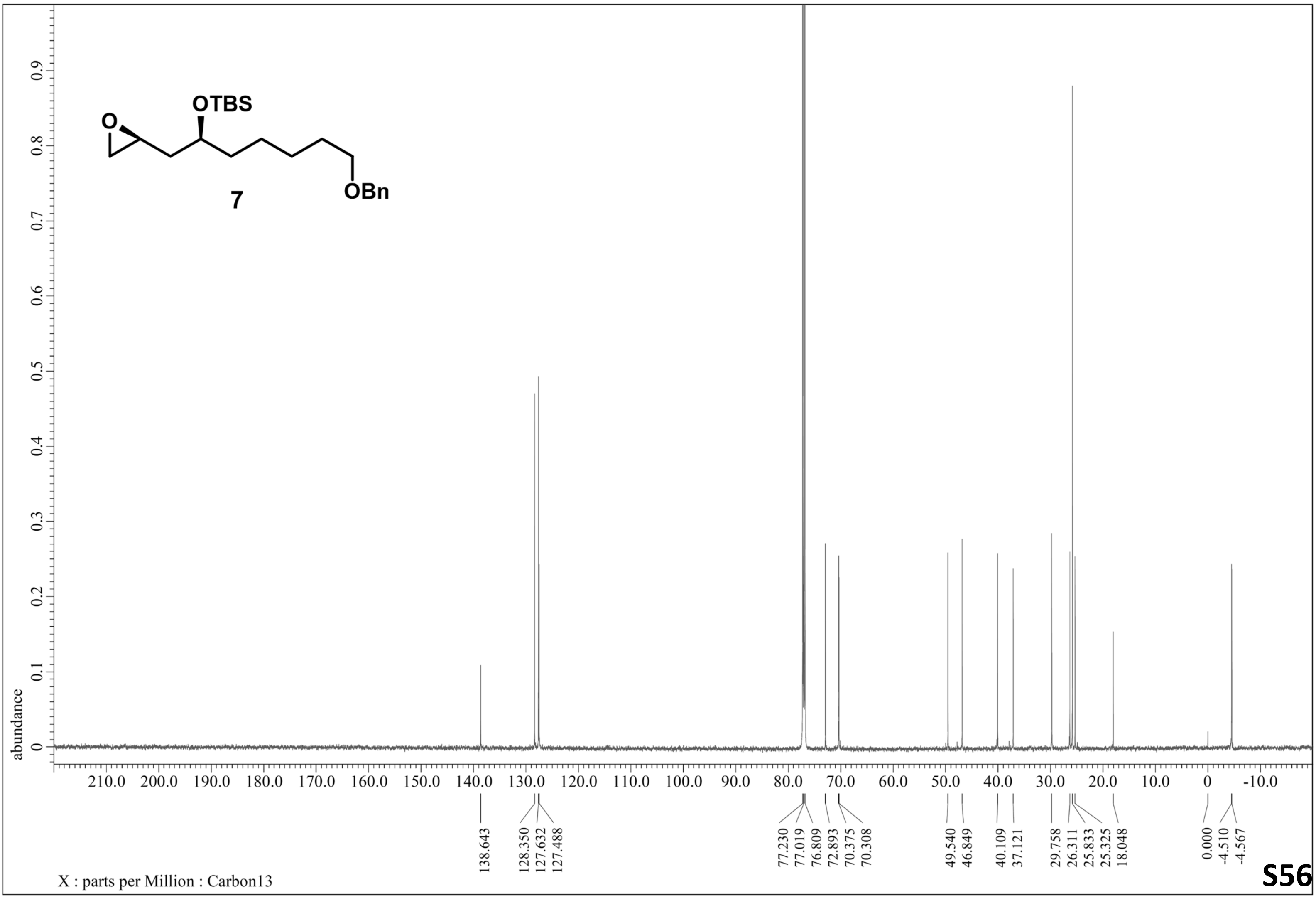

^1H NMR (600 MHz, $CDCl_3$) and ^{13}C NMR (151 MHz, $CDCl_3$) spectra of **S5**

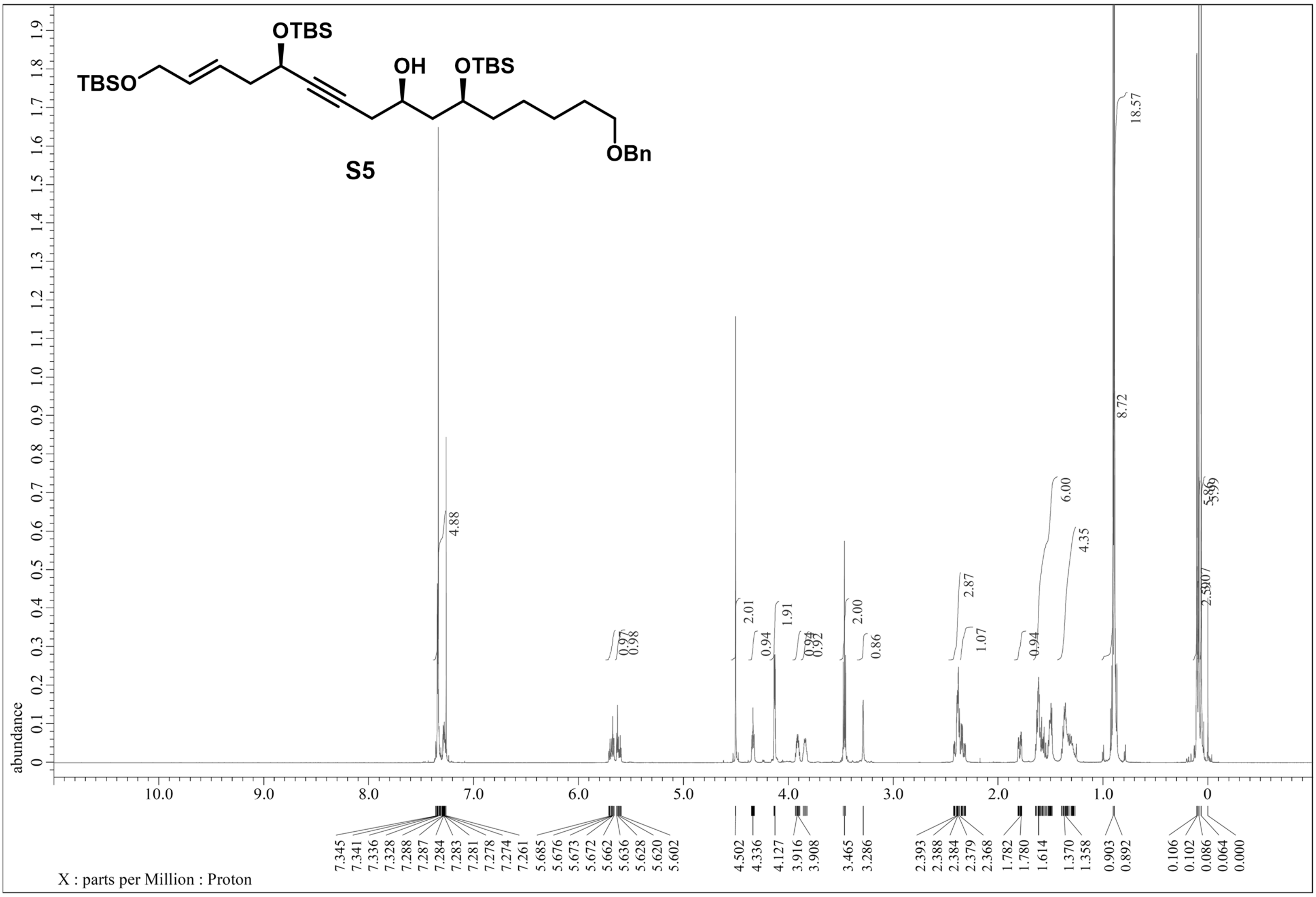

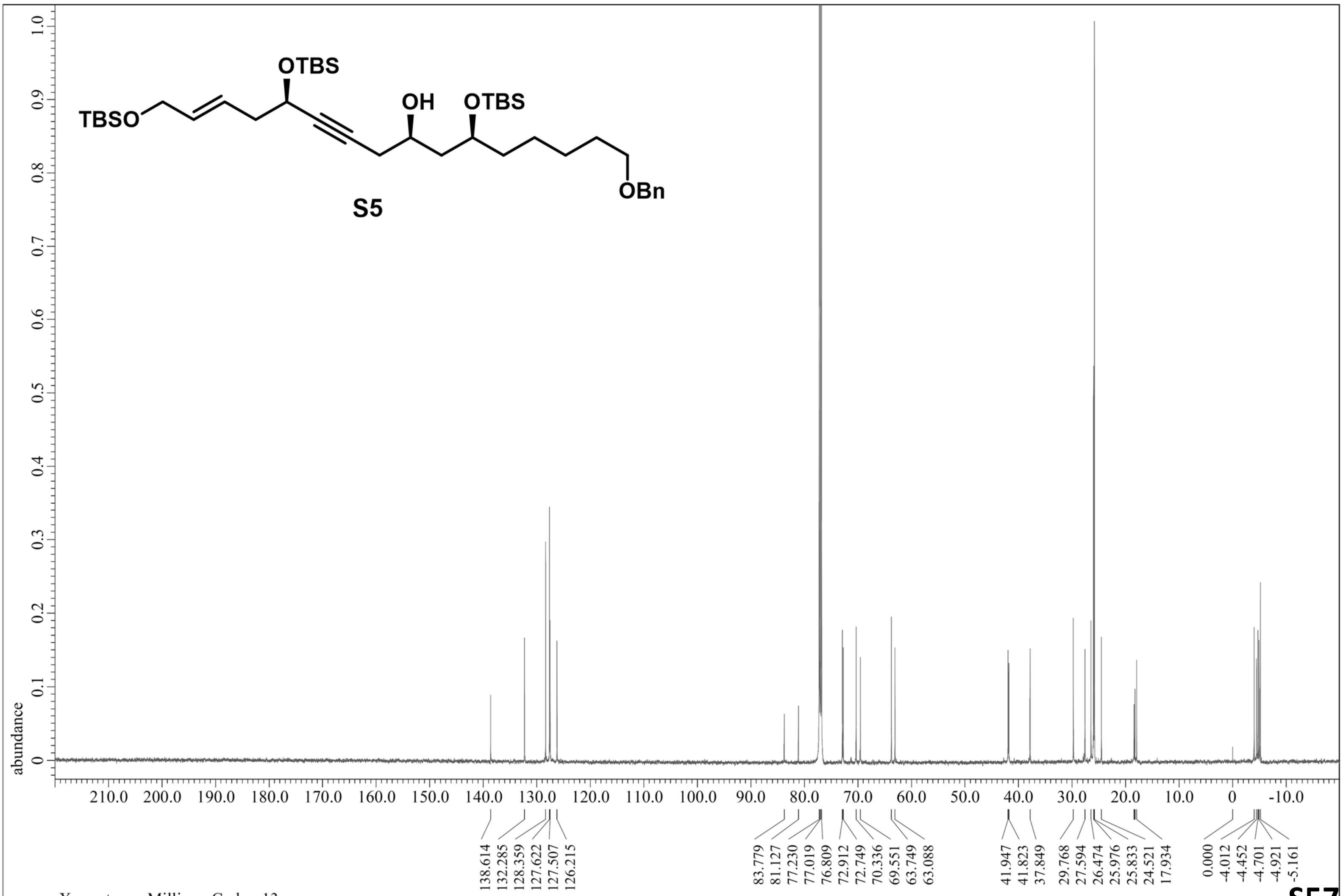

^{1}H NMR (600 MHz, $CDCl_3$) and ^{13}C NMR (151 MHz, $CDCl_3$) spectra of **14**

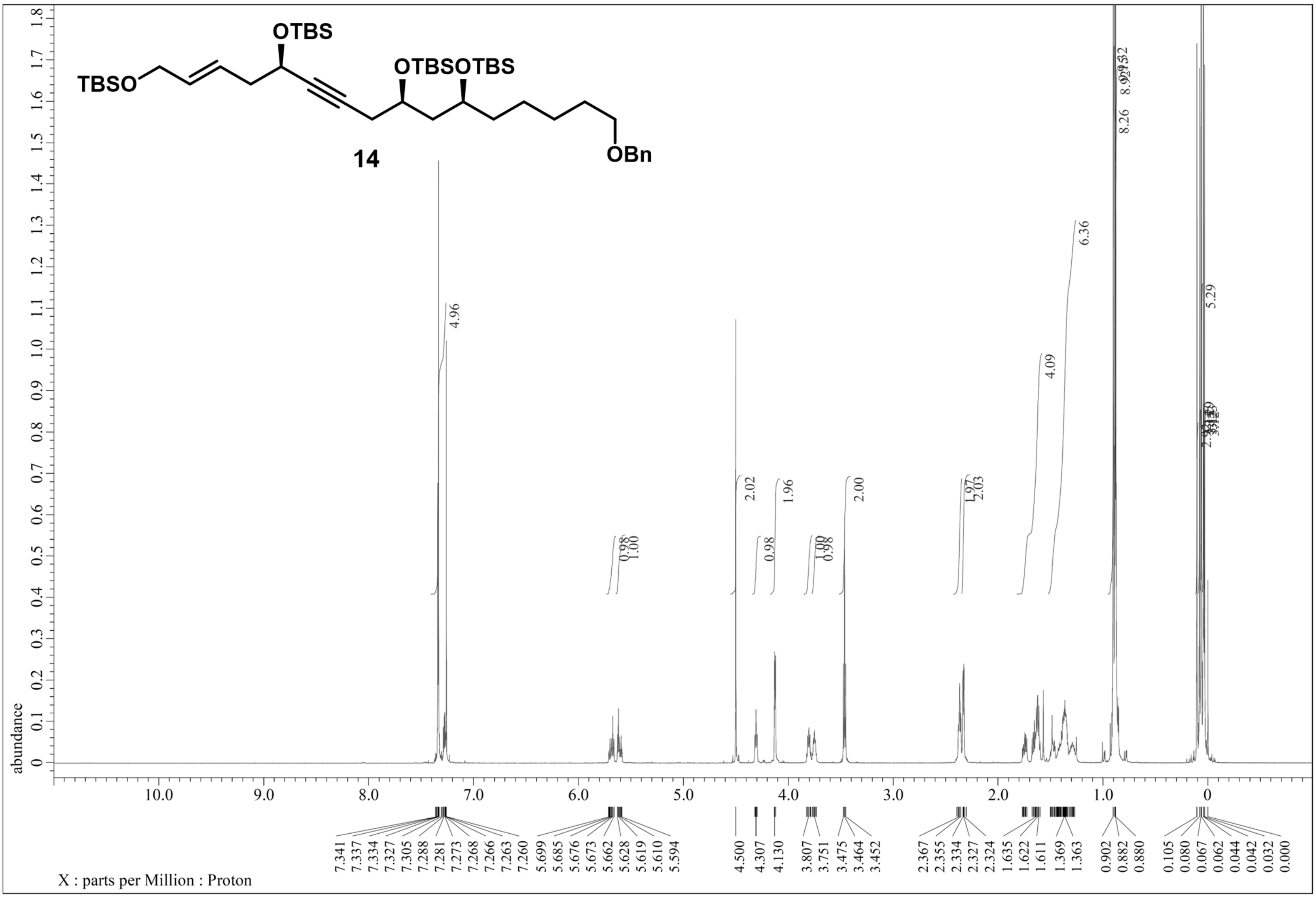

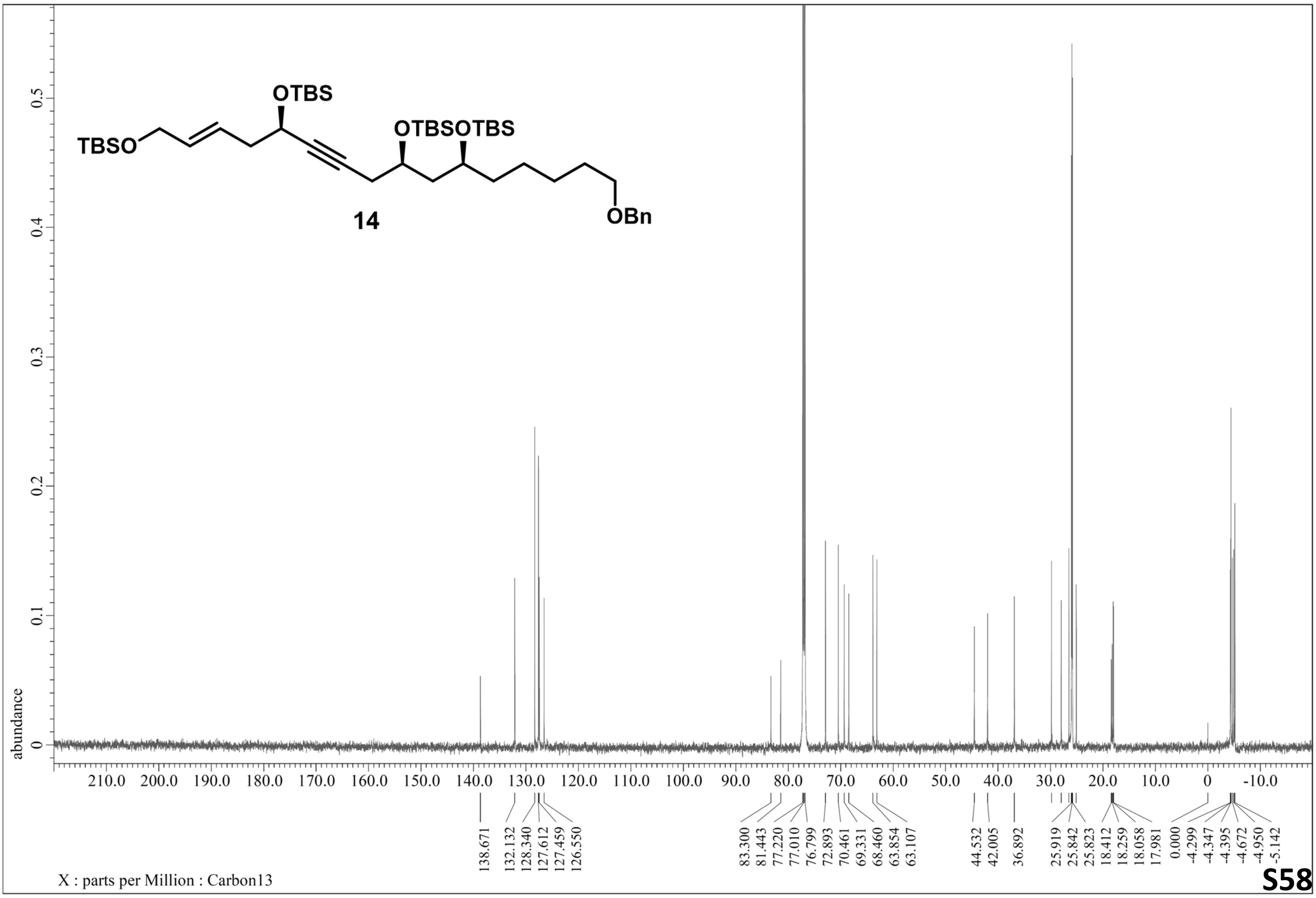

^{1}H NMR (600 MHz, $CDCl_3$) and ^{13}C NMR (151 MHz, $CDCl_3$) spectra of **15**

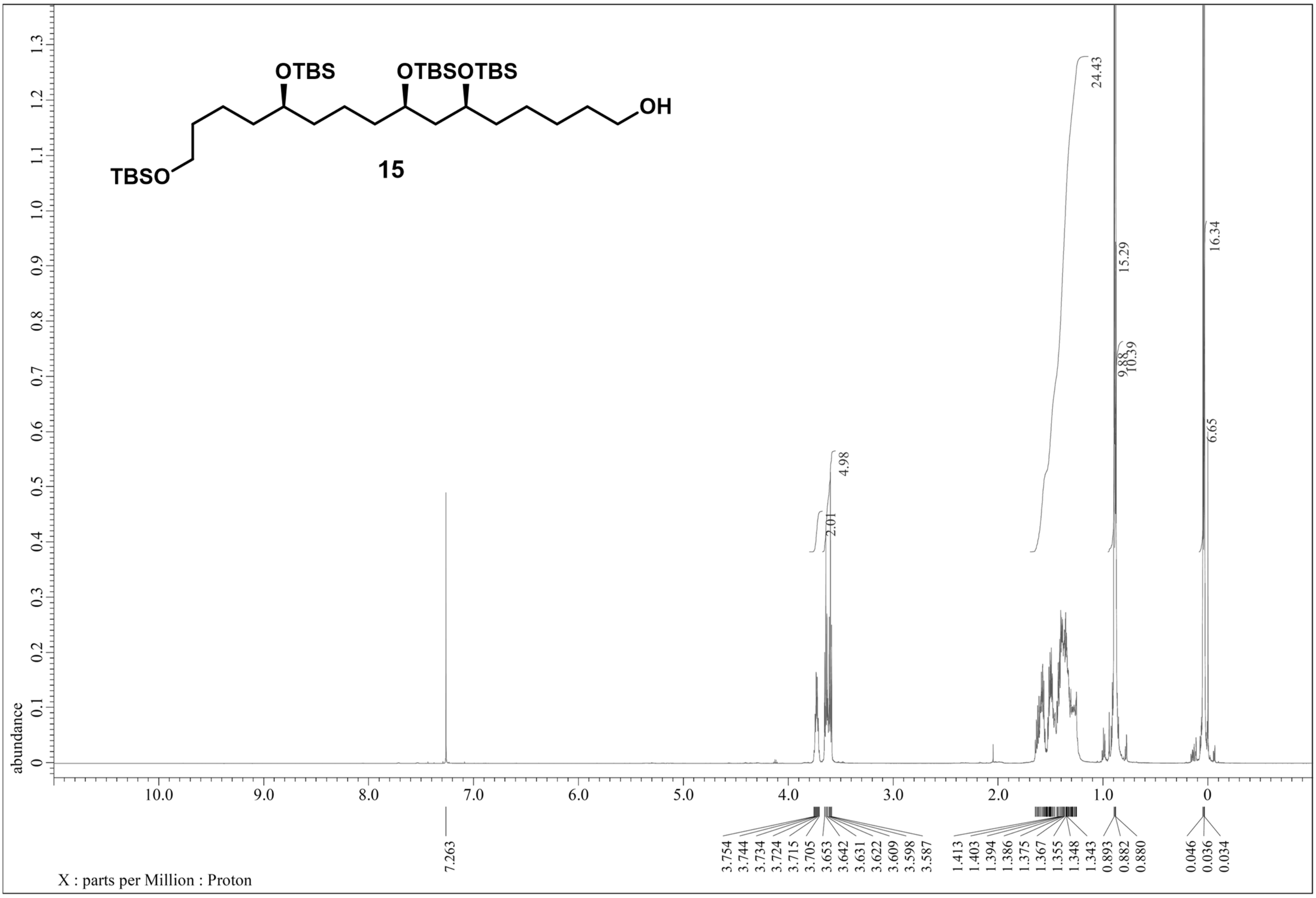

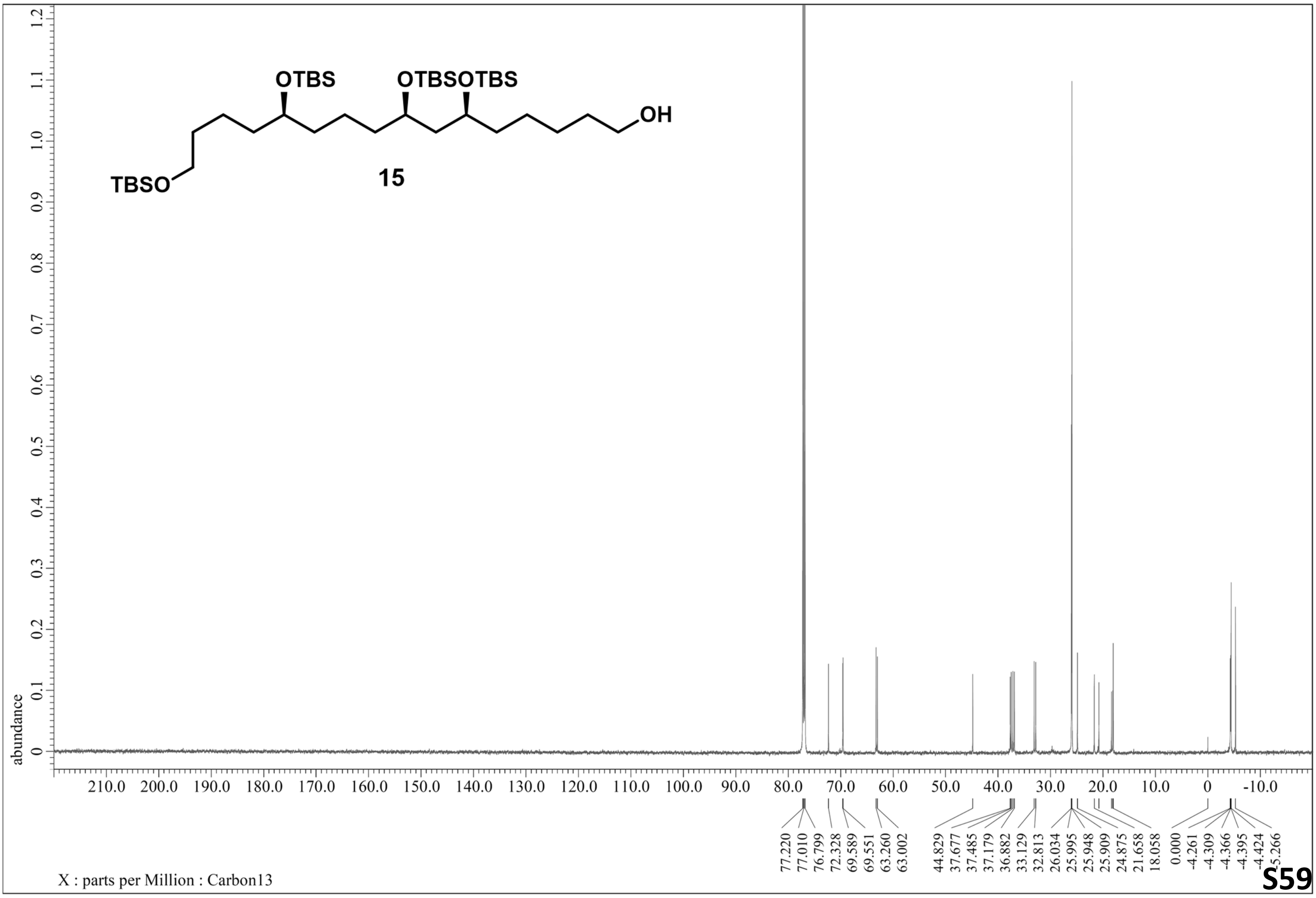

^{1}H NMR (600 MHz, $CDCl_3$) and ^{13}C NMR (151 MHz, $CDCl_3$) spectra of **S6**

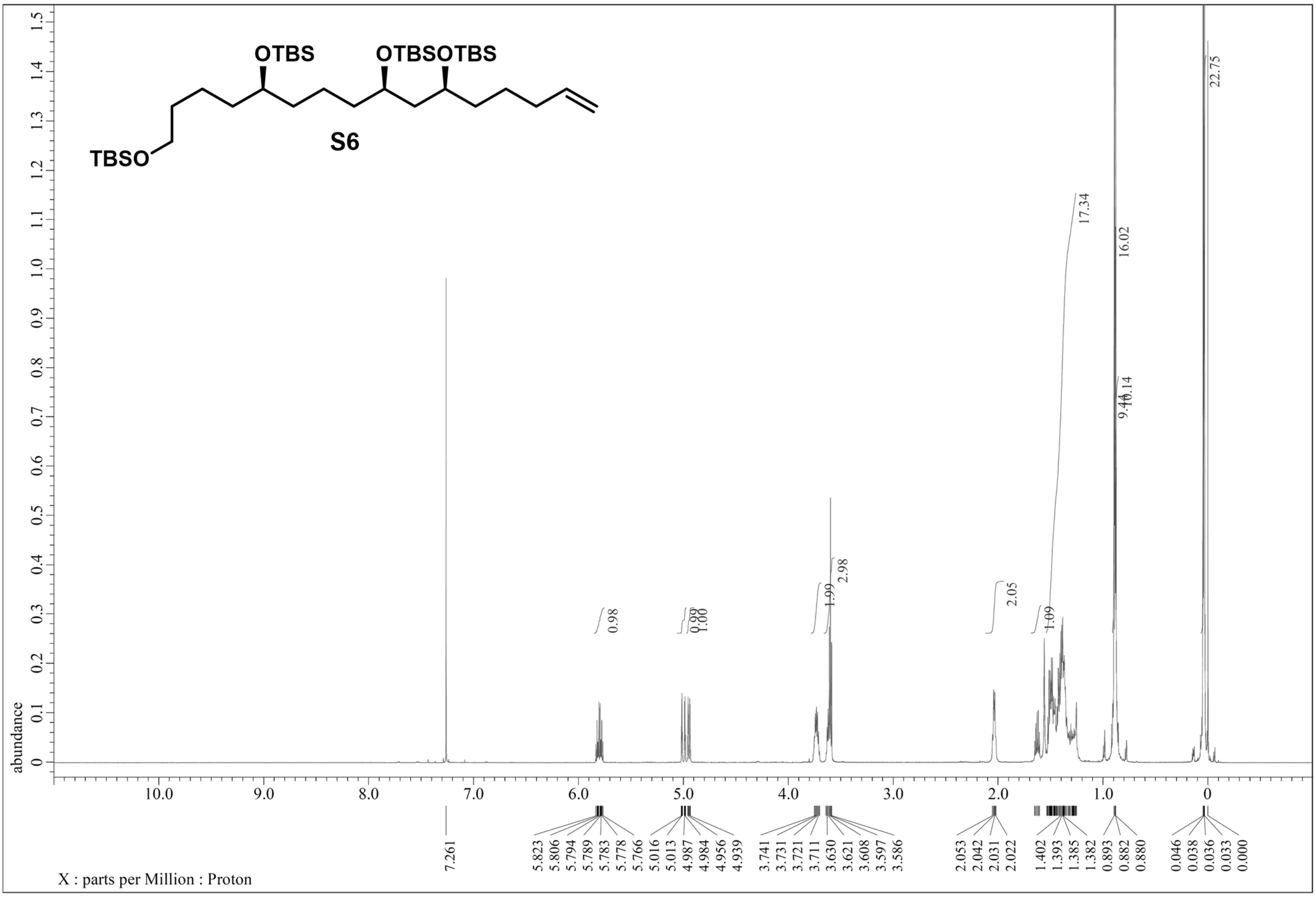

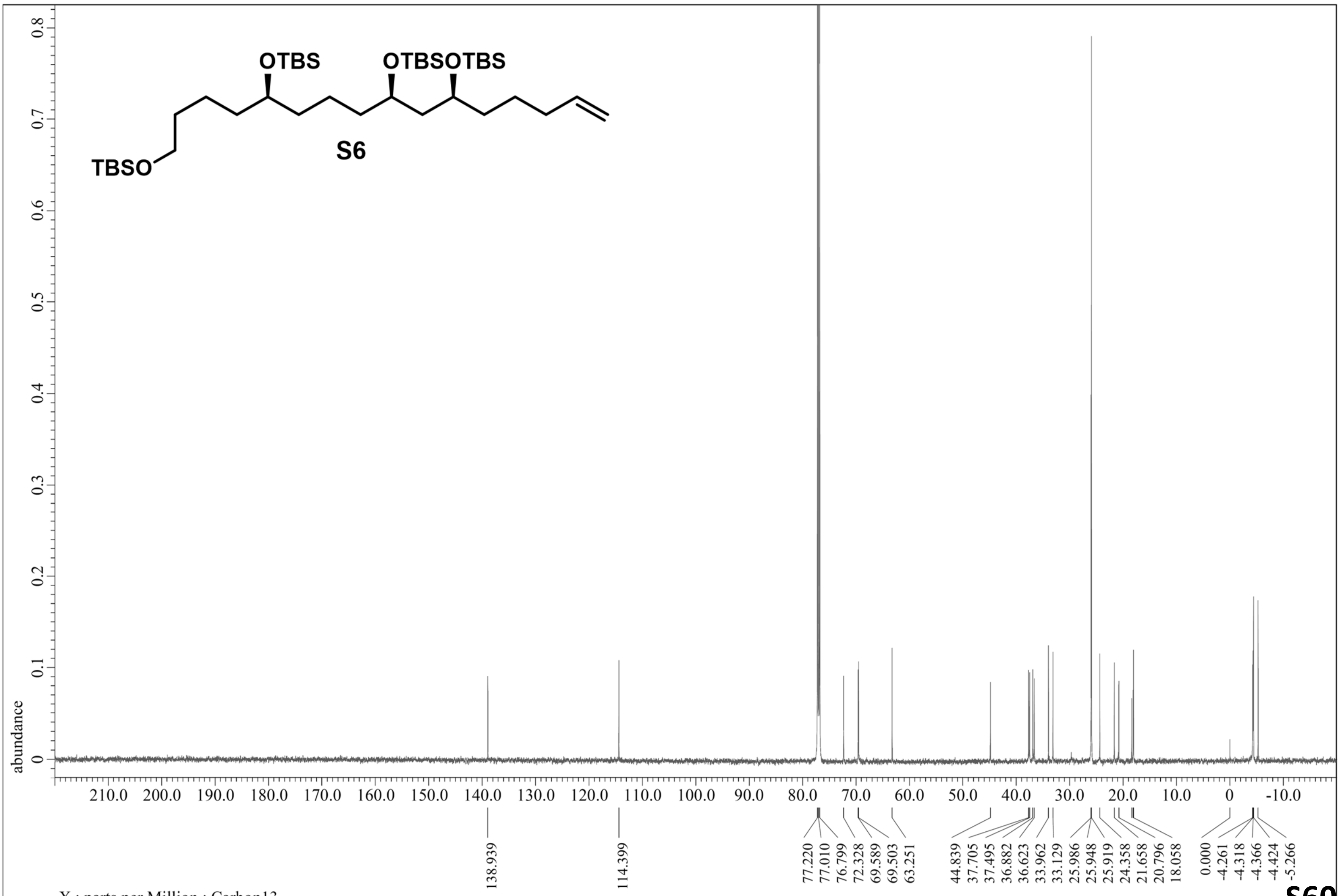

^{1}H NMR (600 MHz, $CDCl_3$) and ^{13}C NMR (151 MHz, $CDCl_3$) spectra of **S7**

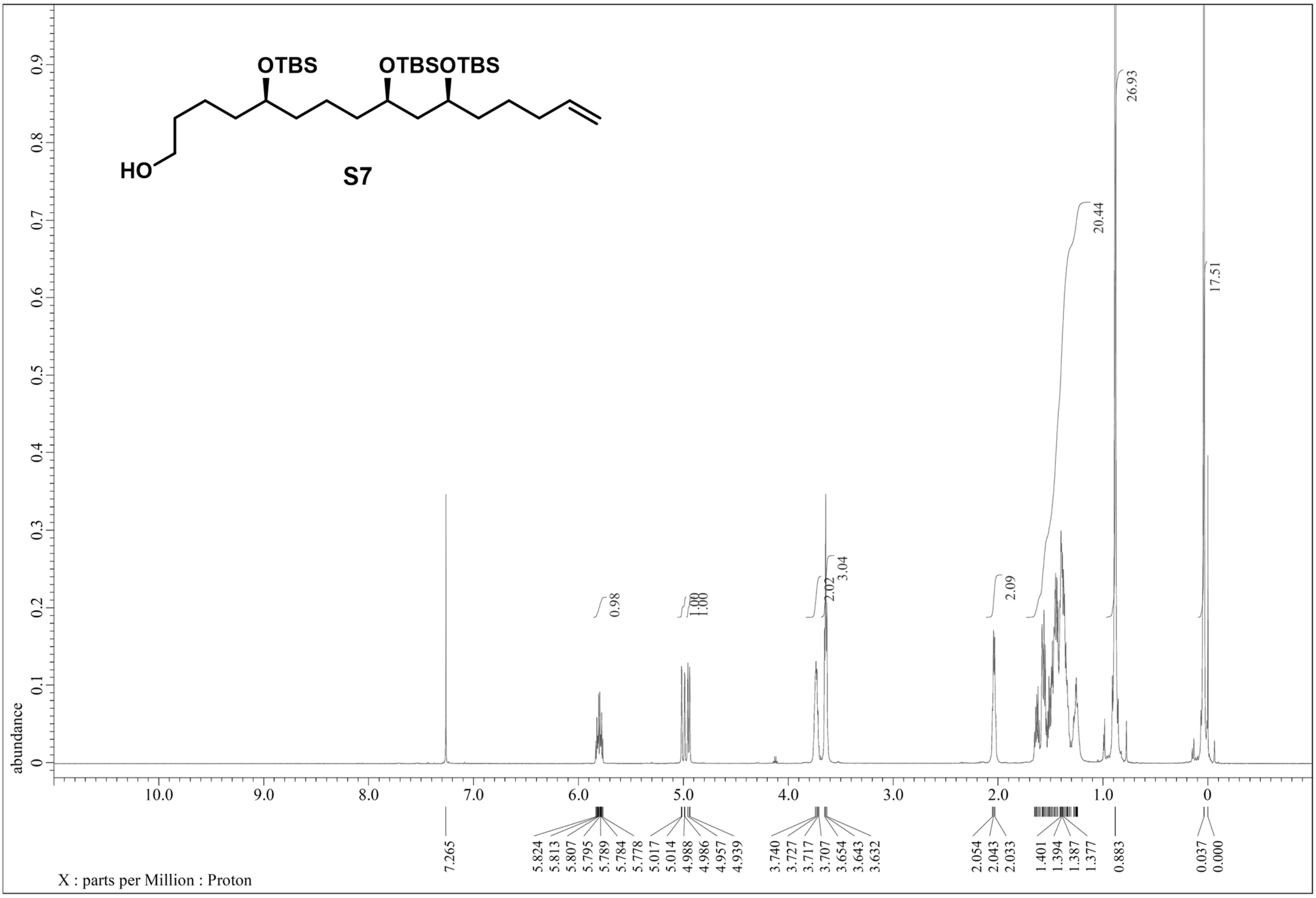

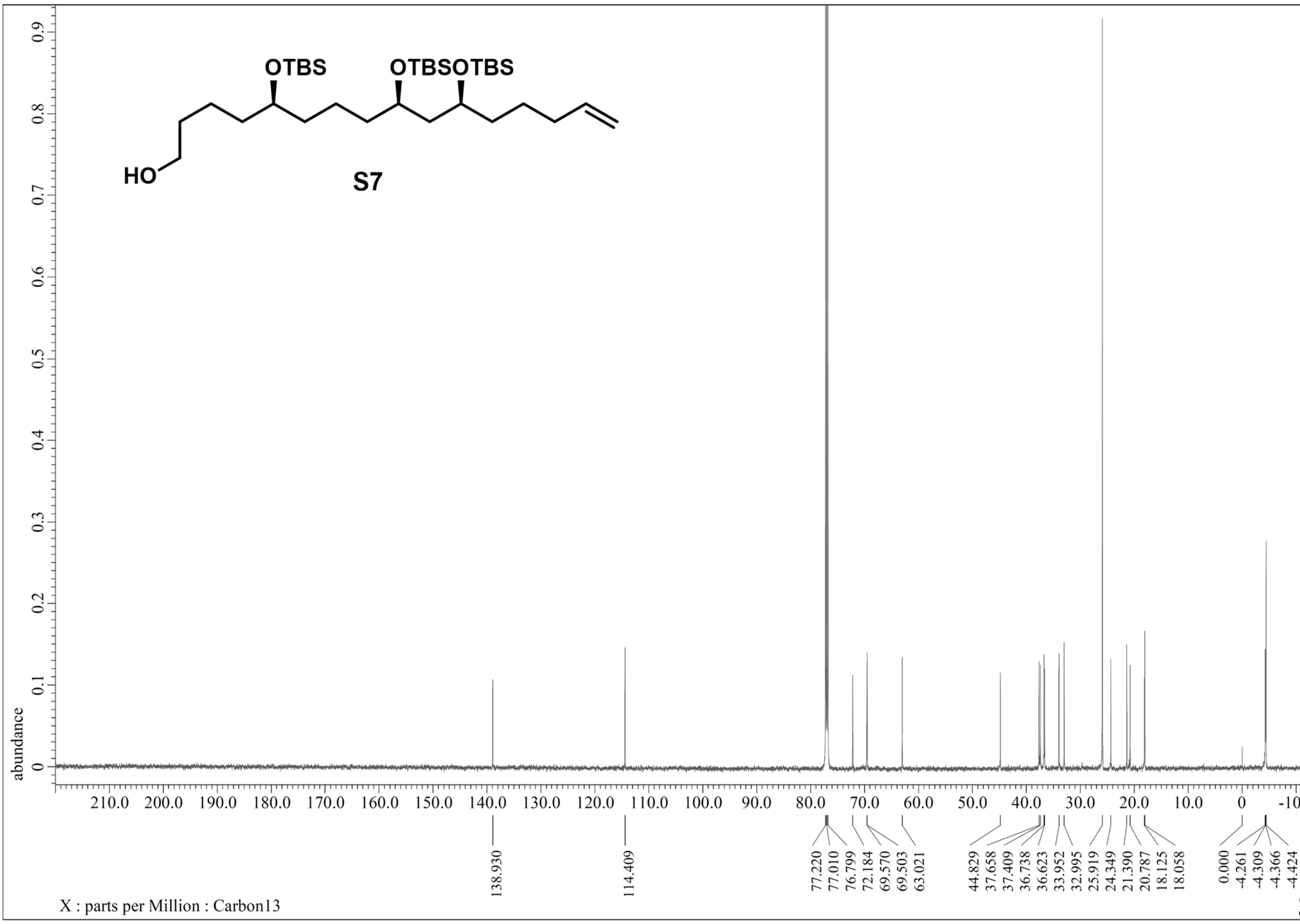

^1H NMR (600 MHz, $CDCl_3$) and ^{13}C NMR (151 MHz, $CDCl_3$) spectra of **4**

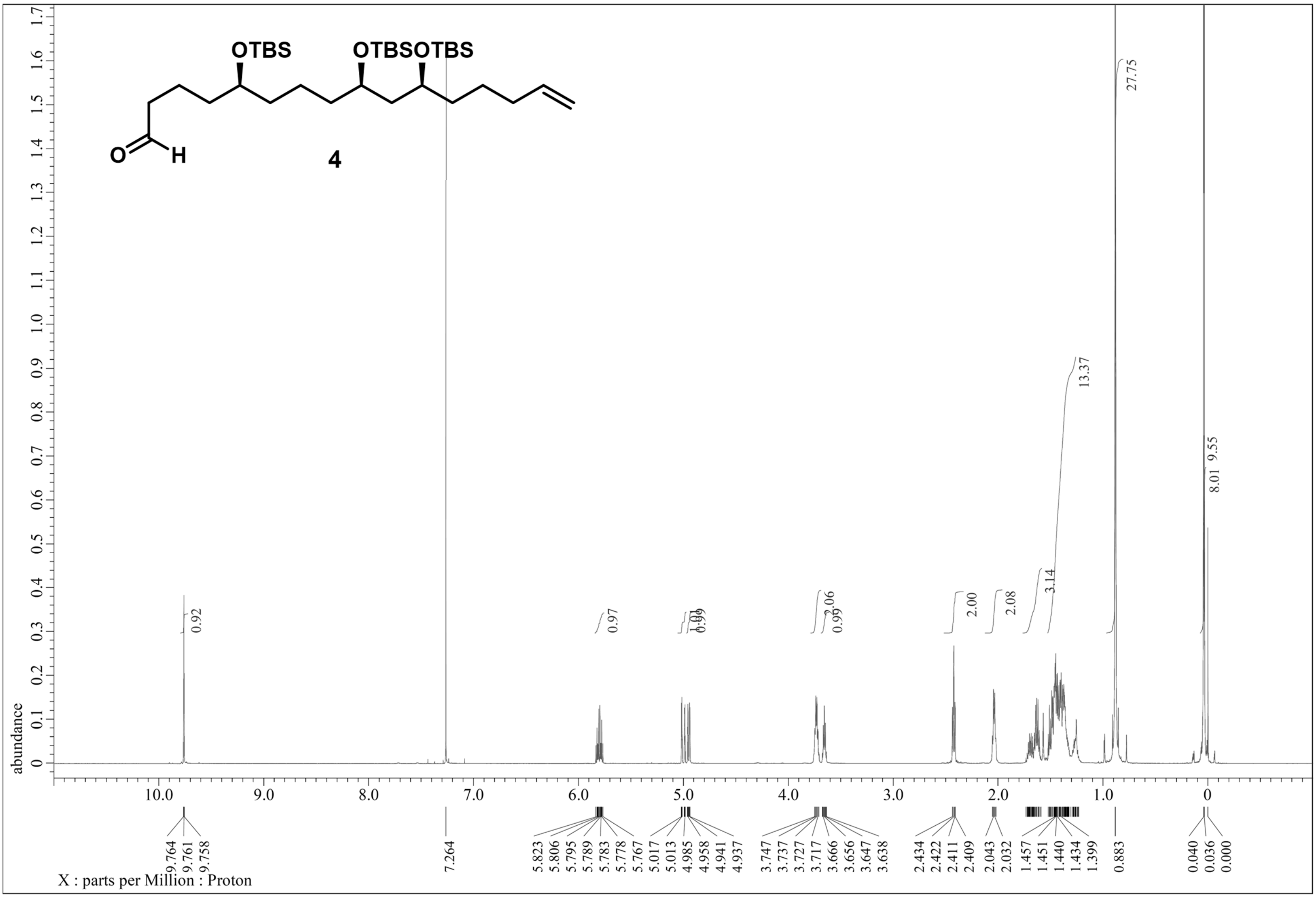

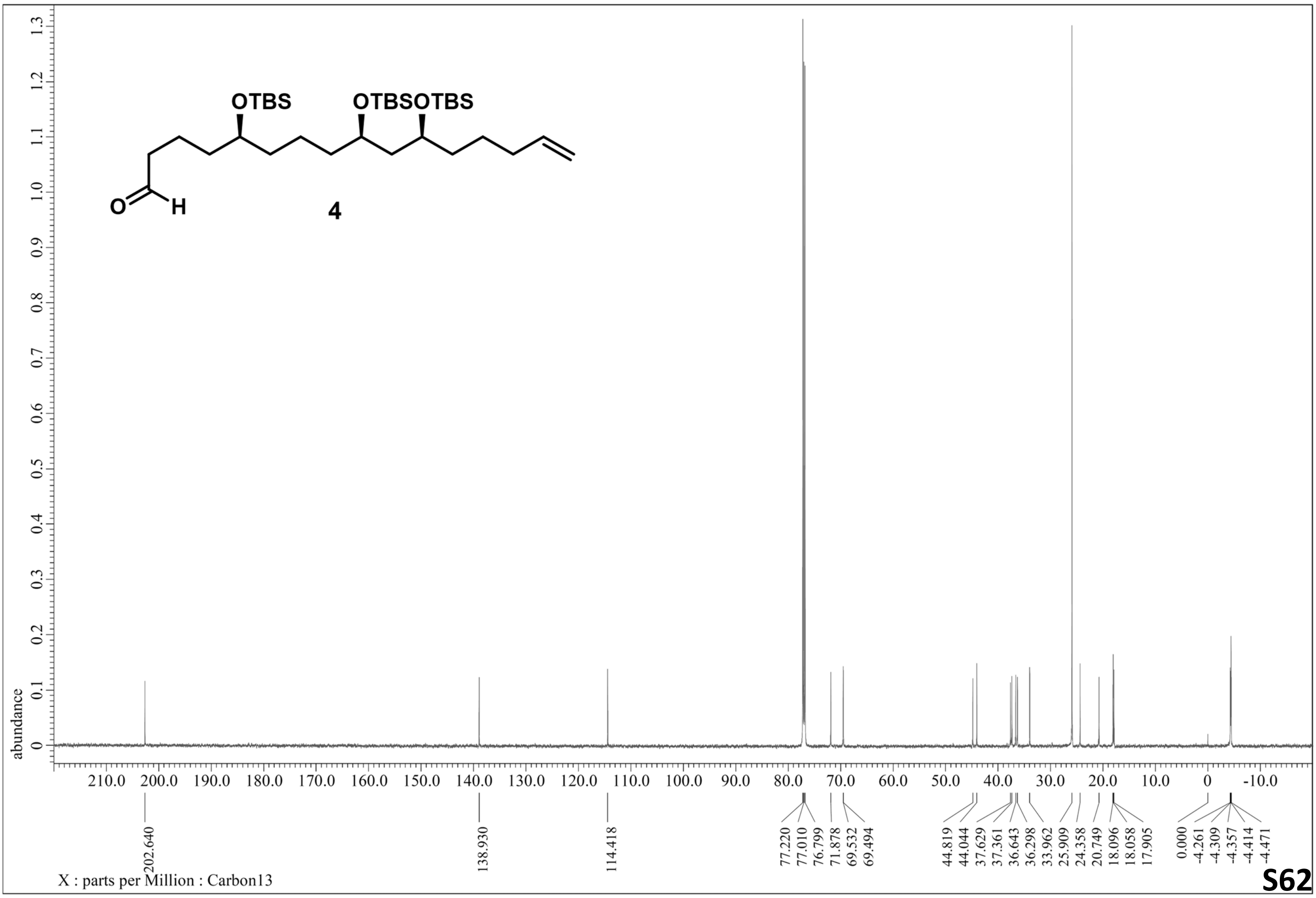

^{1}H NMR (600 MHz, $CDCl_3$) and ^{13}C NMR (151 MHz, $CDCl_3$) spectra of **S8**

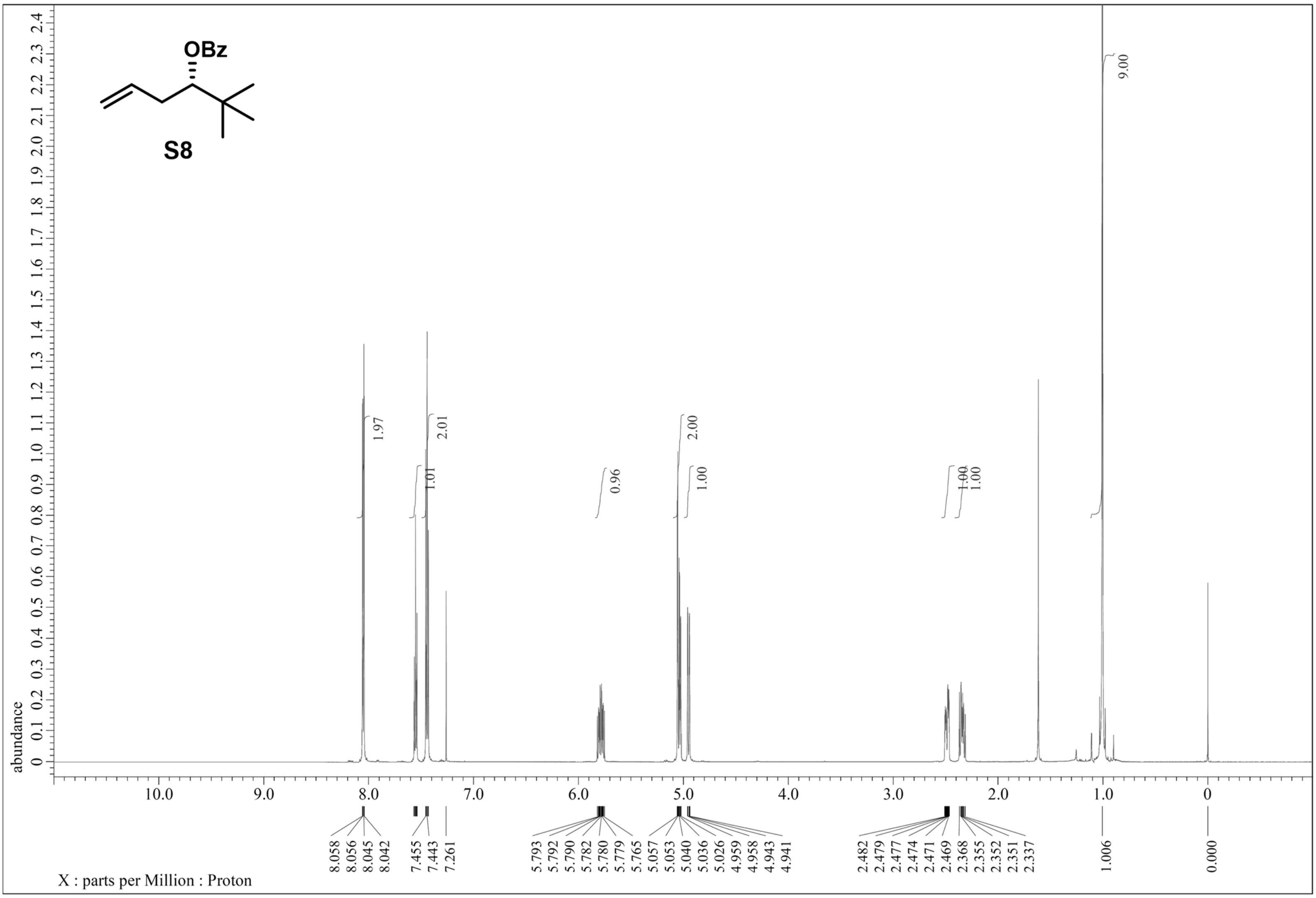

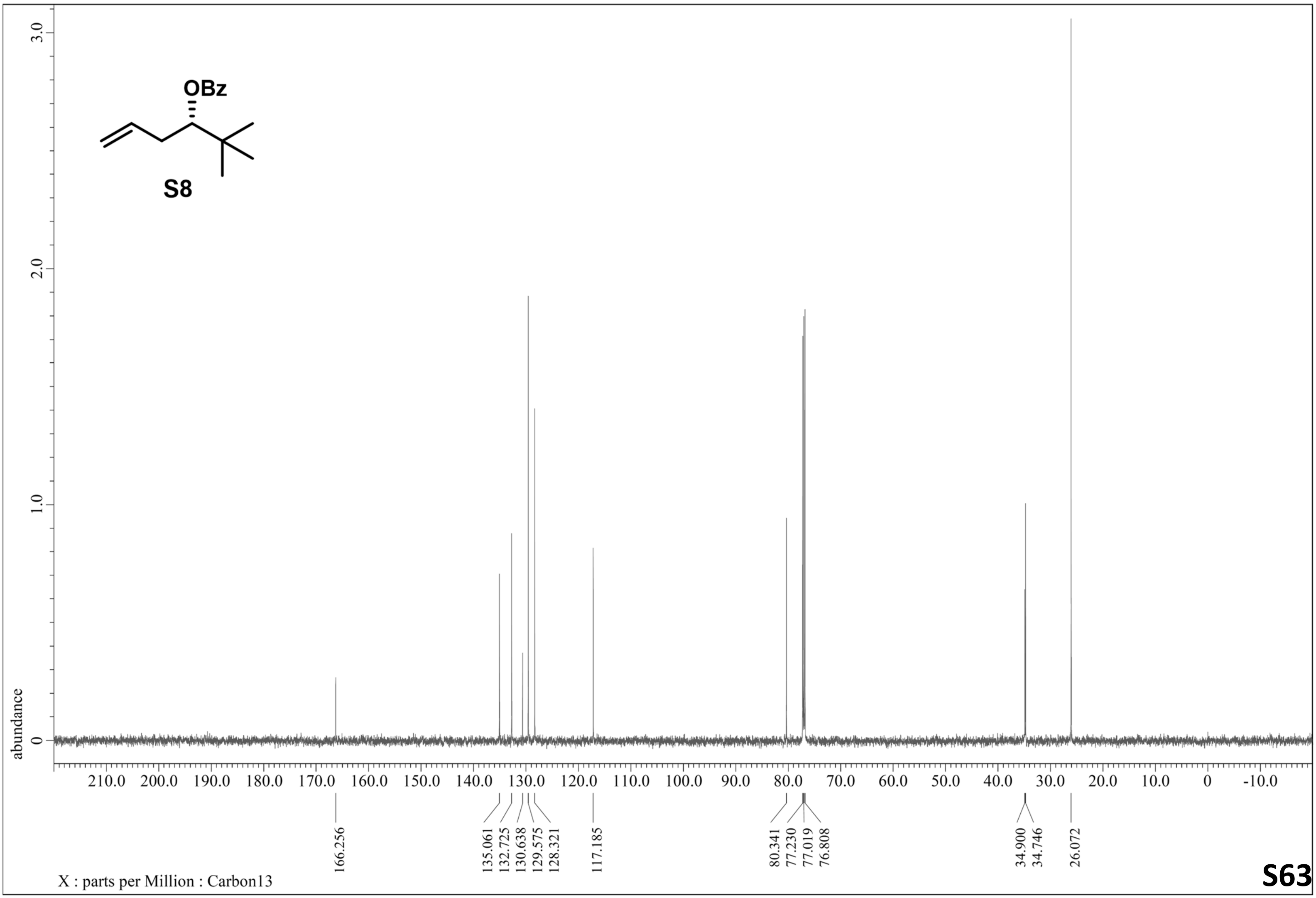

^1H NMR (600 MHz, $CDCl_3$) and ^{13}C NMR (151 MHz, $CDCl_3$) spectra of **S9**

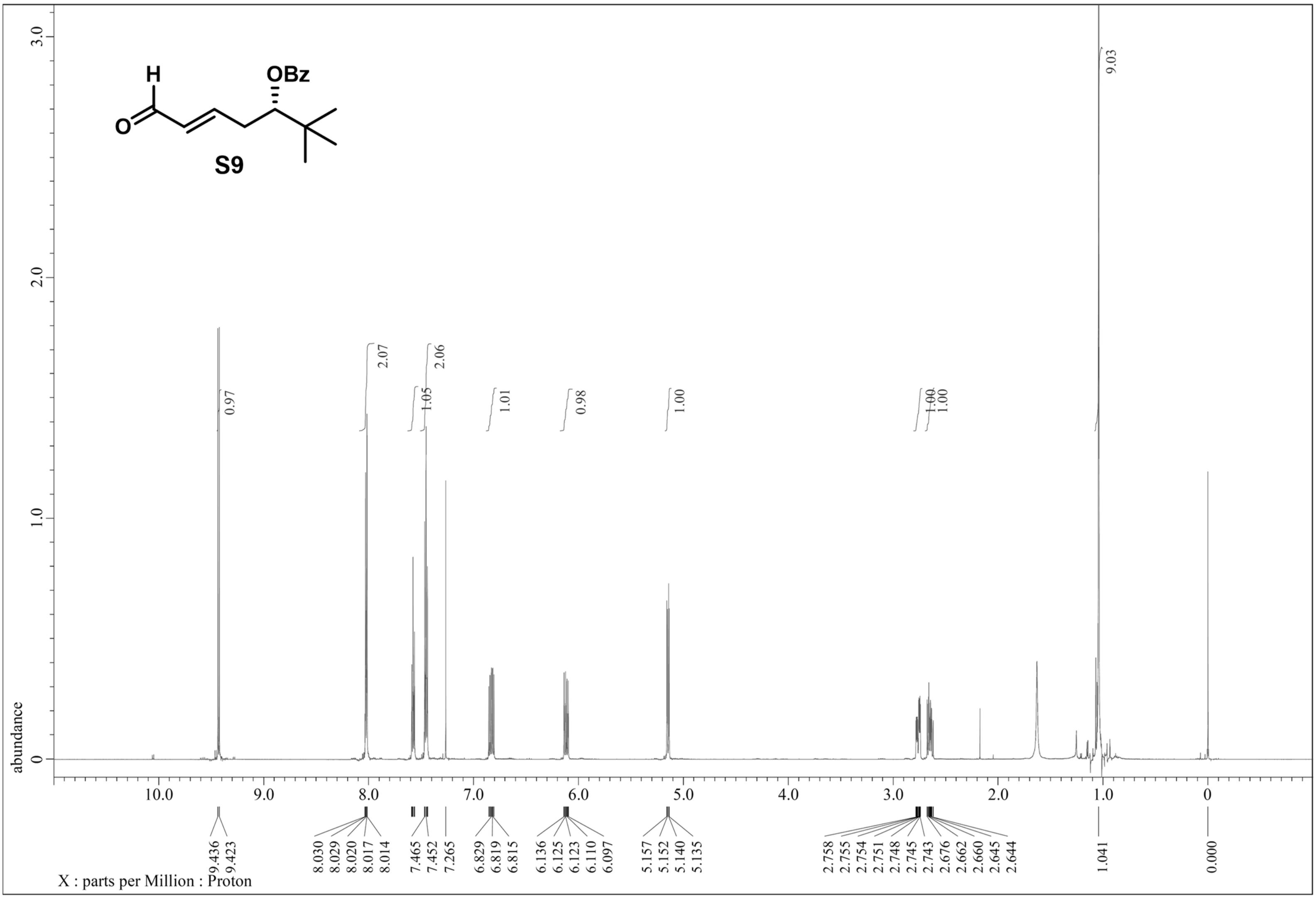

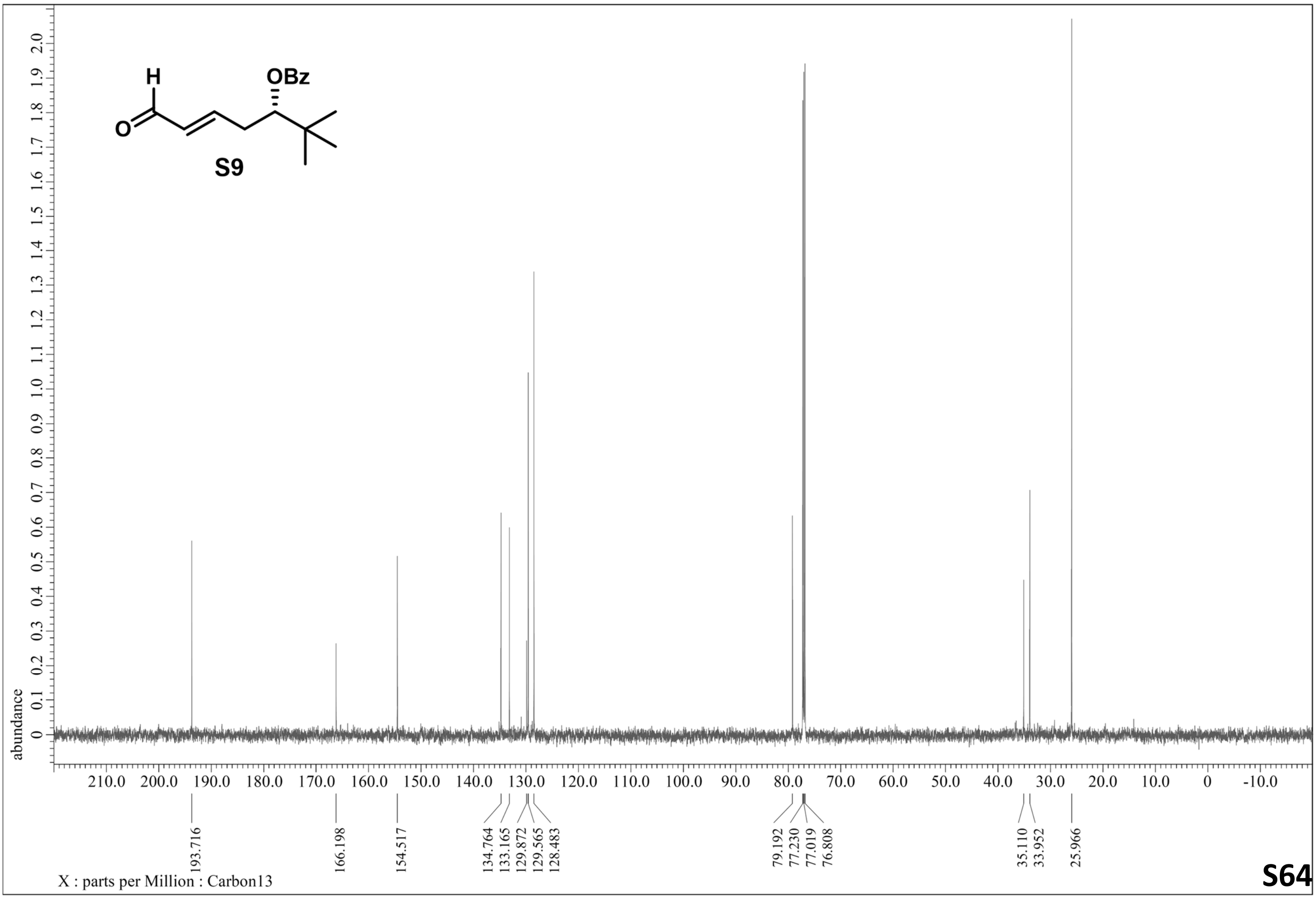

^{1}H NMR (600 MHz, $CDCl_3$) and ^{13}C NMR (151 MHz, $CDCl_3$) spectra of **9**

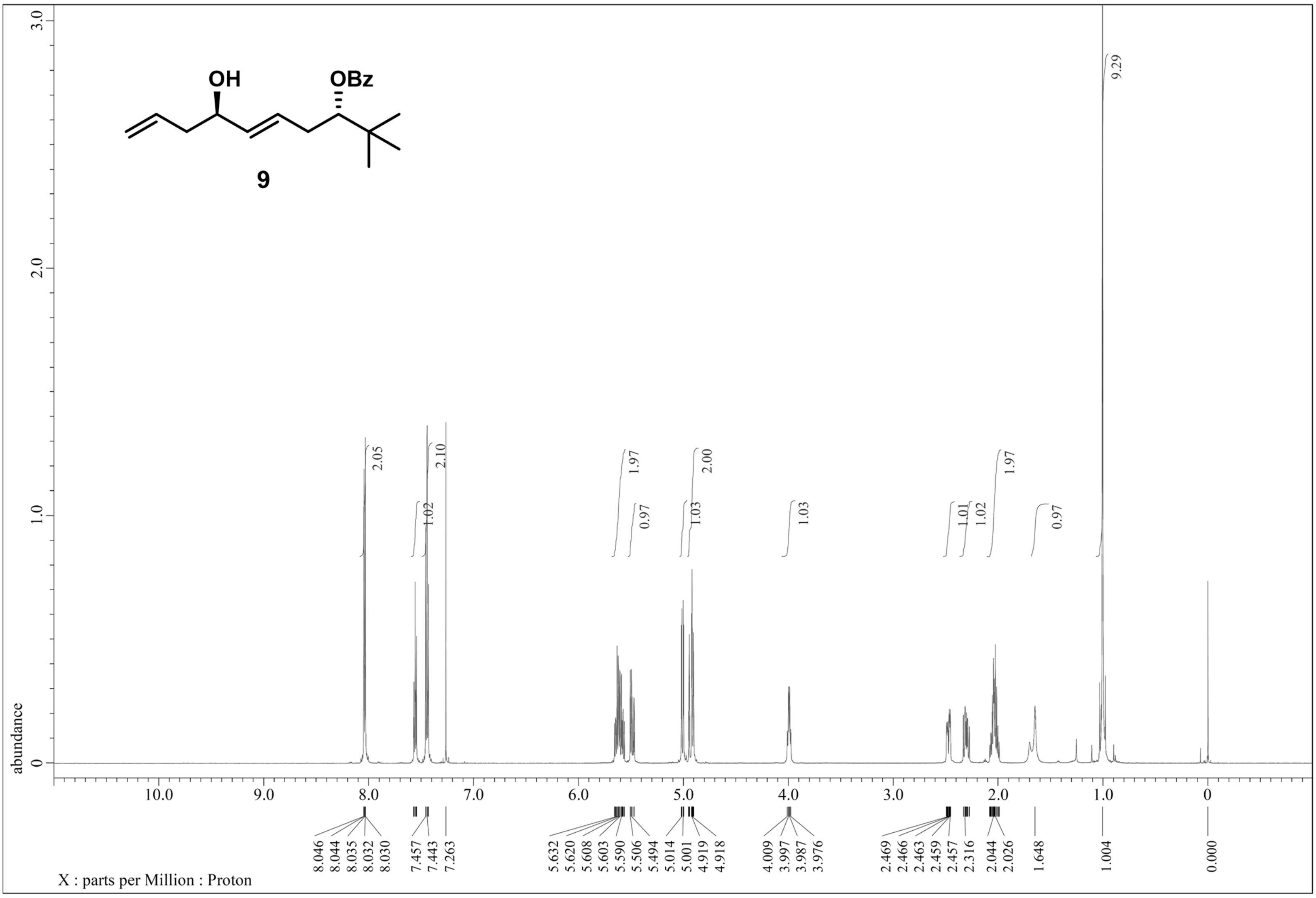

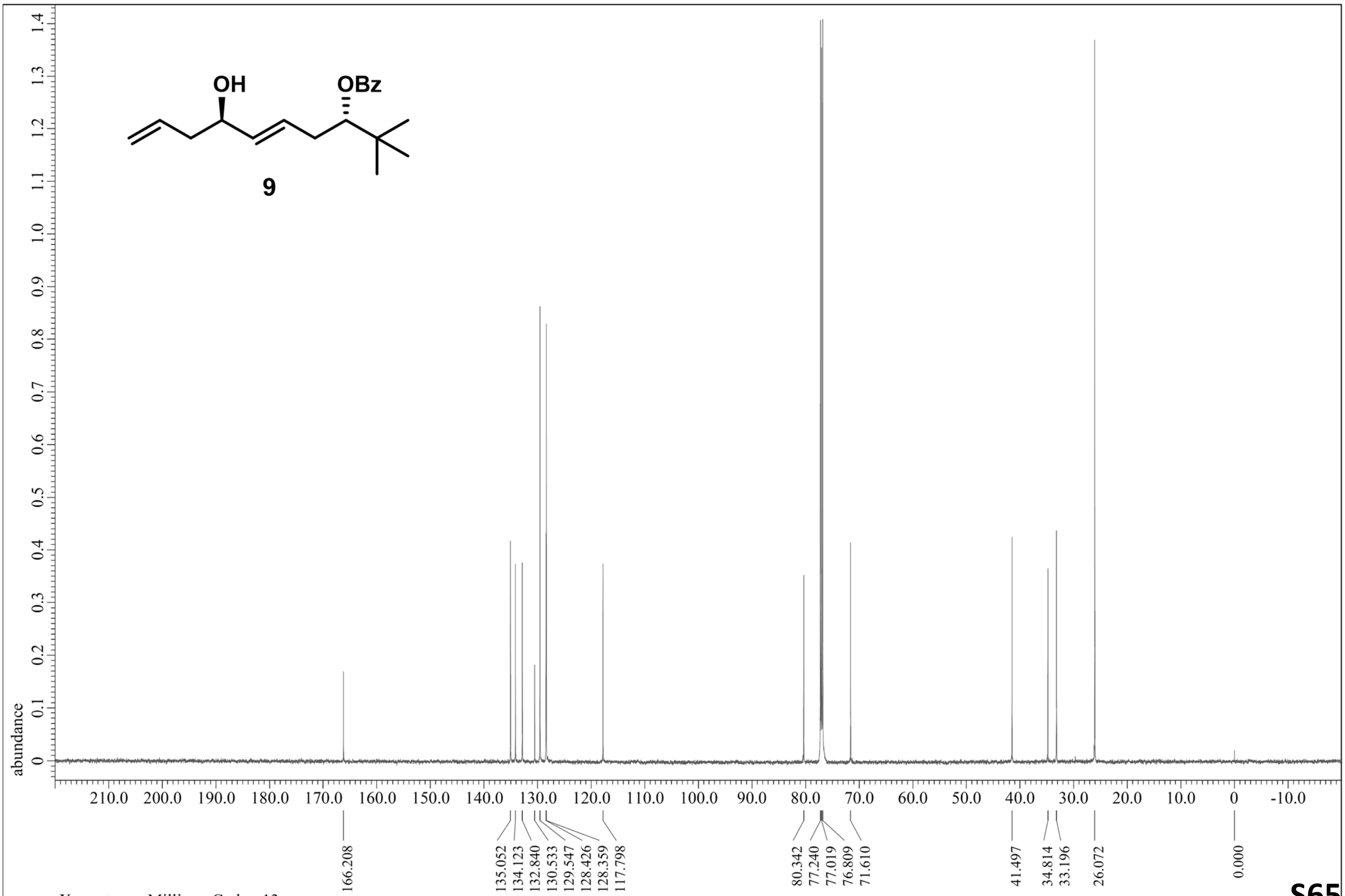

^{1}H NMR (600 MHz, $CDCl_3$) and ^{13}C NMR (151 MHz, $CDCl_3$) spectra of **S10**

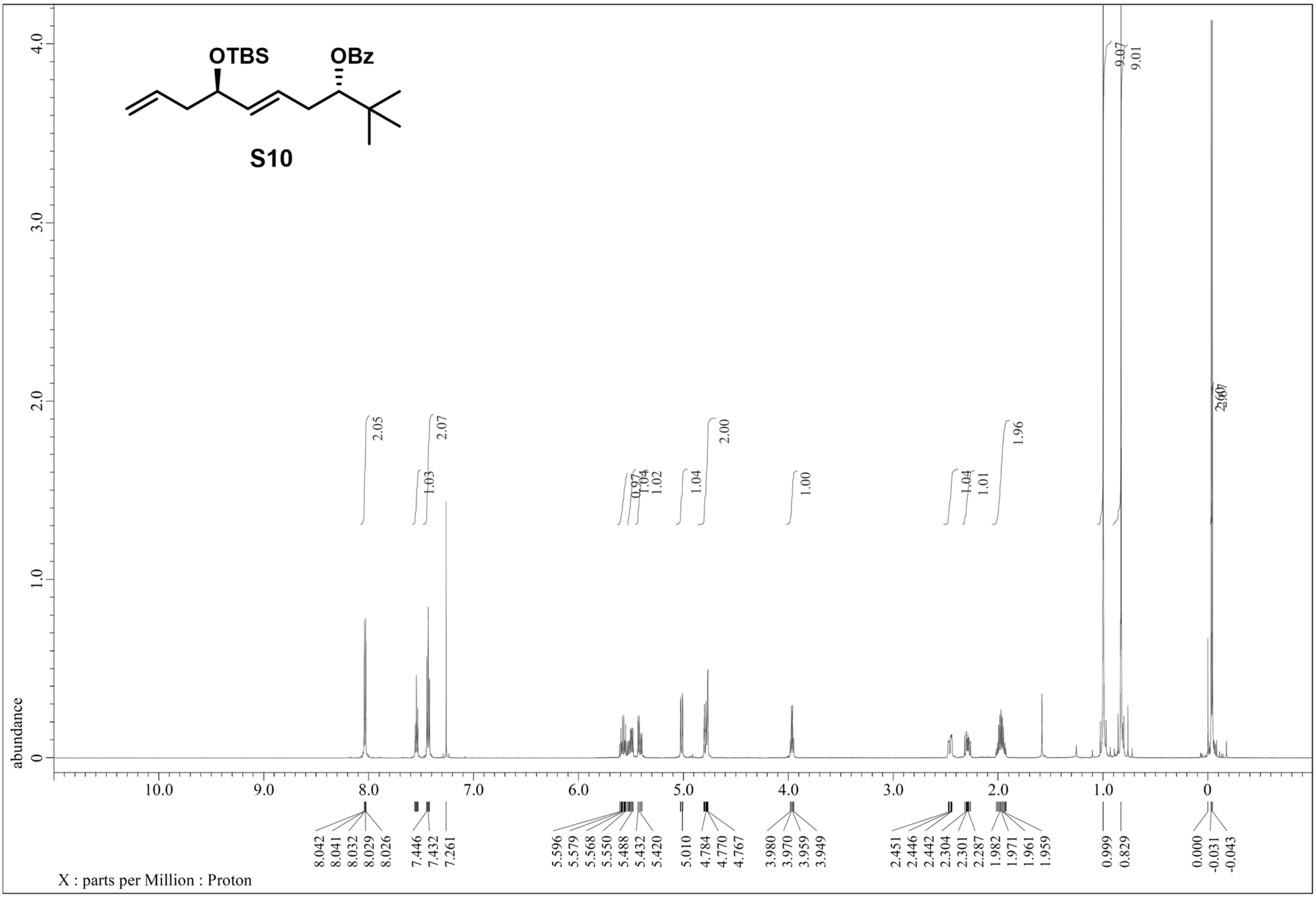

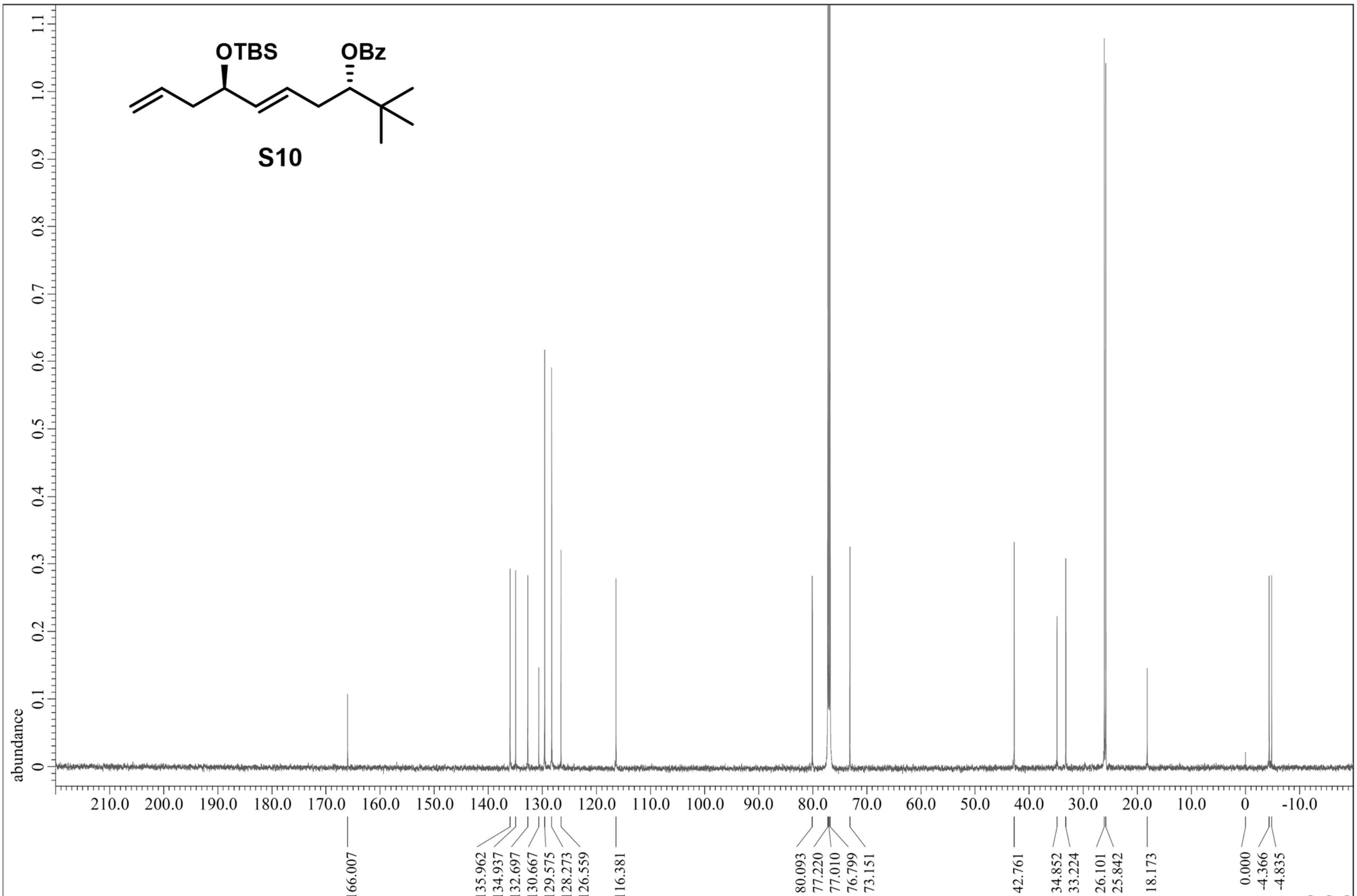

^{1}H NMR (600 MHz, $CDCl_3$) and ^{13}C NMR (151 MHz, $CDCl_3$) spectra of **S11**

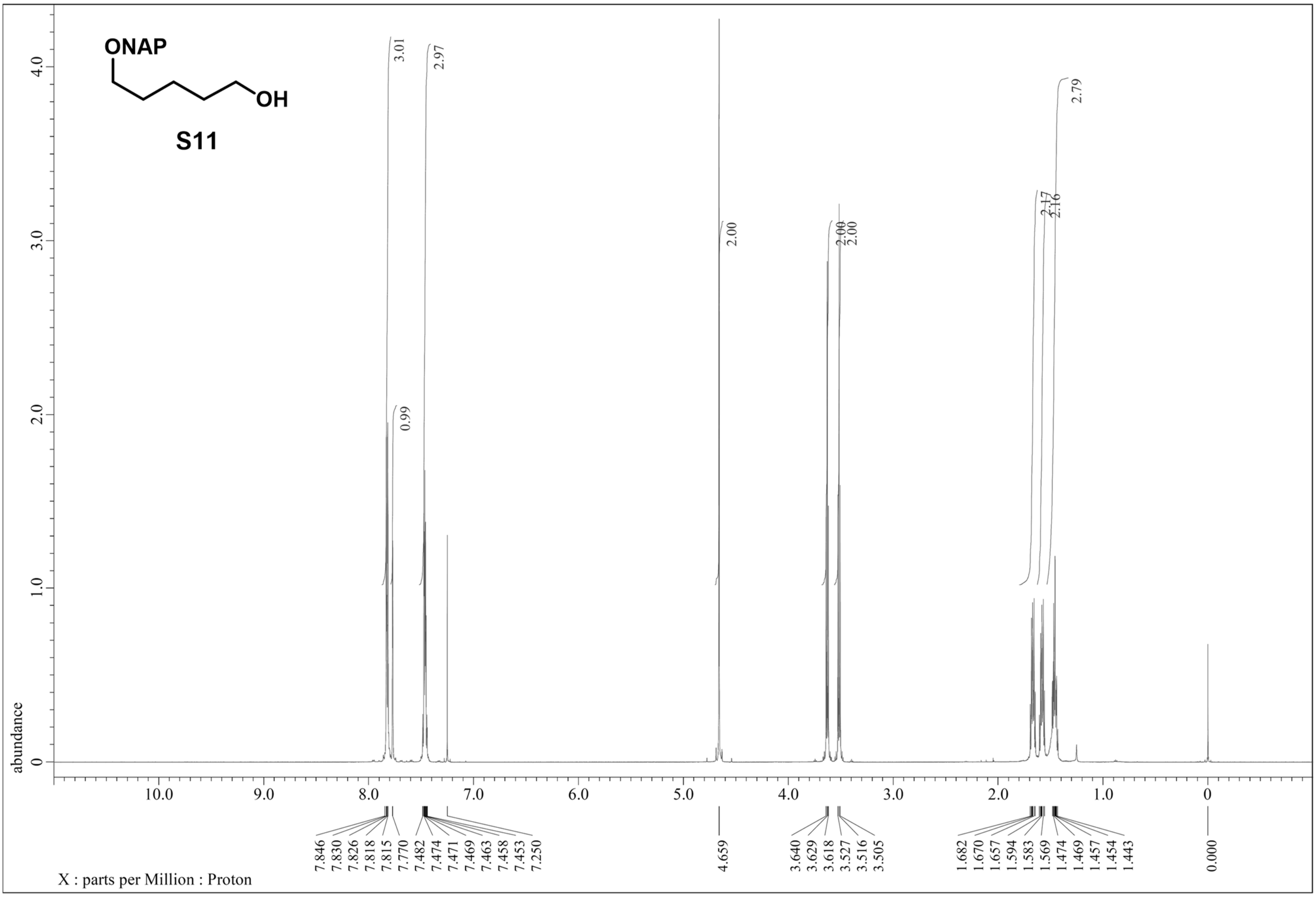

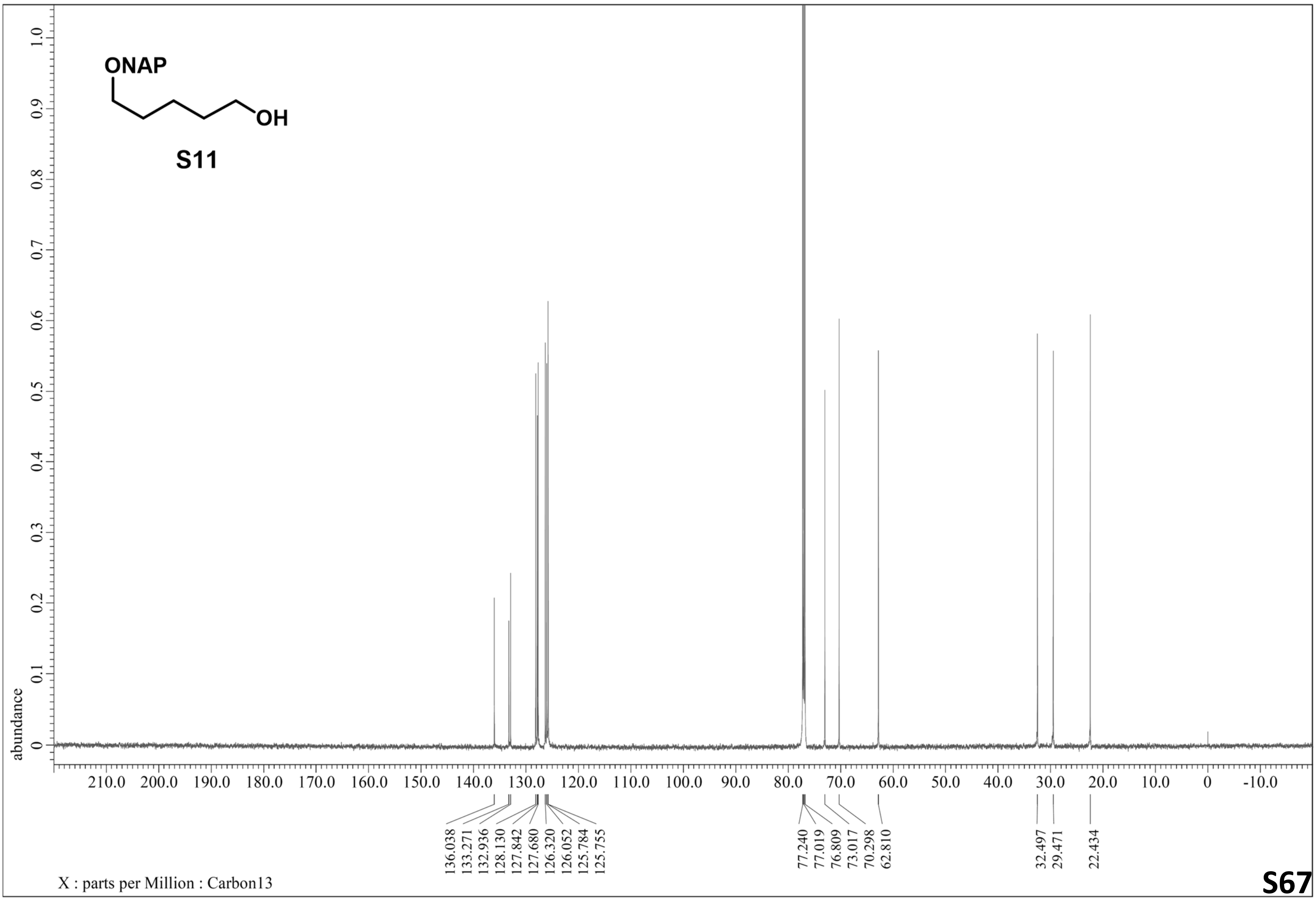

^1H NMR (600 MHz, $CDCl_3$) and ^{13}C NMR (151 MHz, $CDCl_3$) spectra of **23**

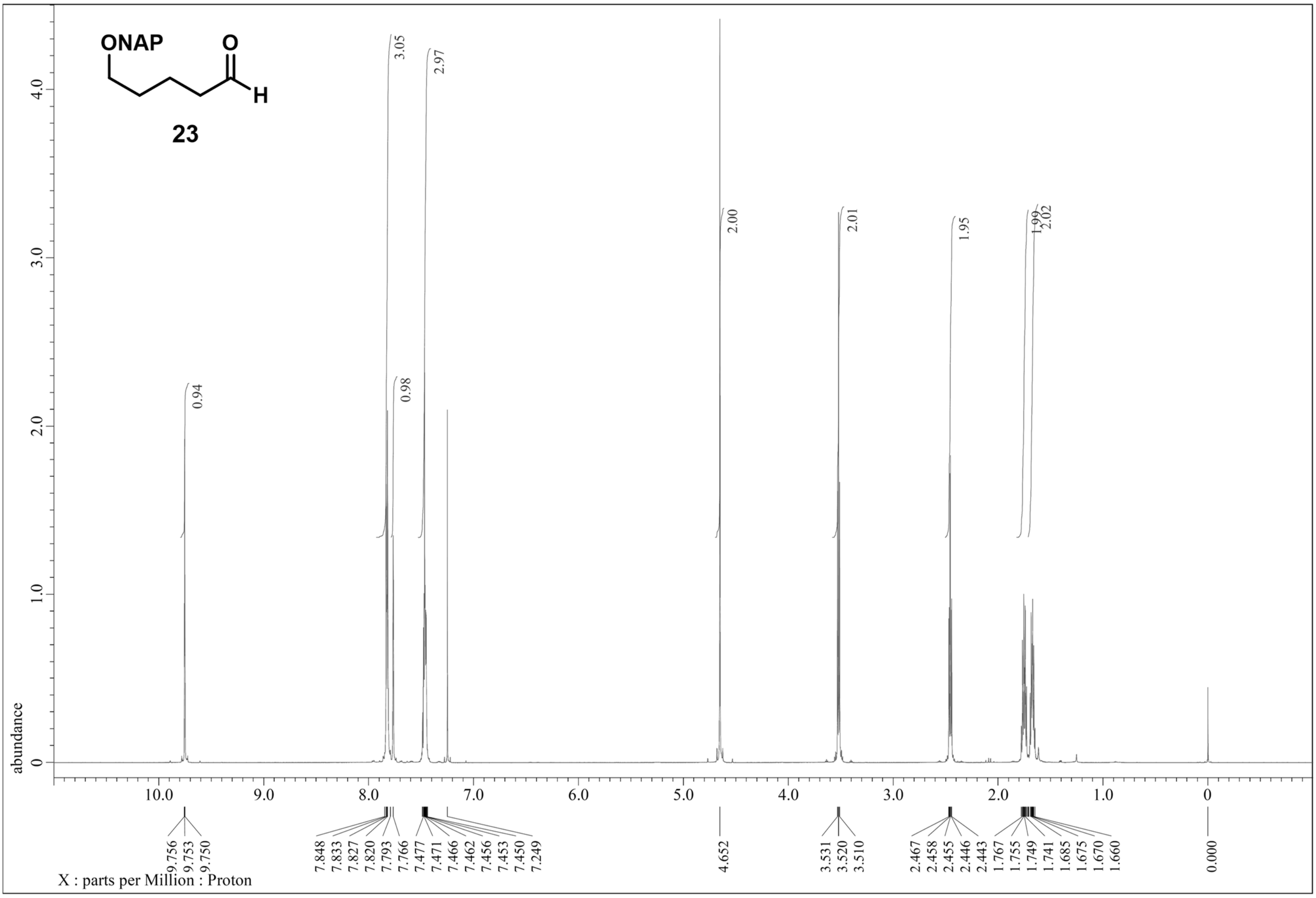

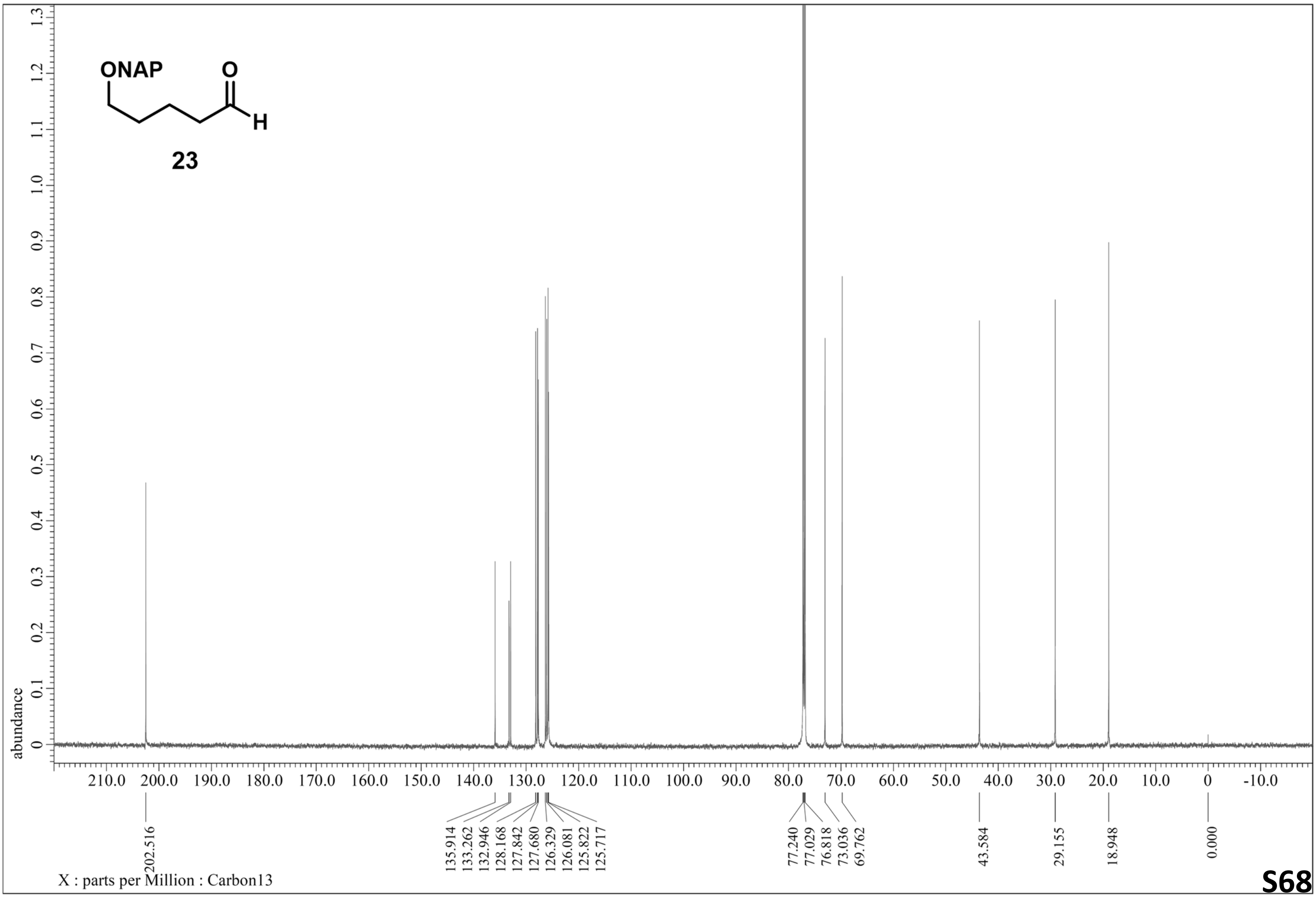

^{1}H NMR (600 MHz, $CDCl_3$) and ^{13}C NMR (151 MHz, $CDCl_3$) spectra of **S12**

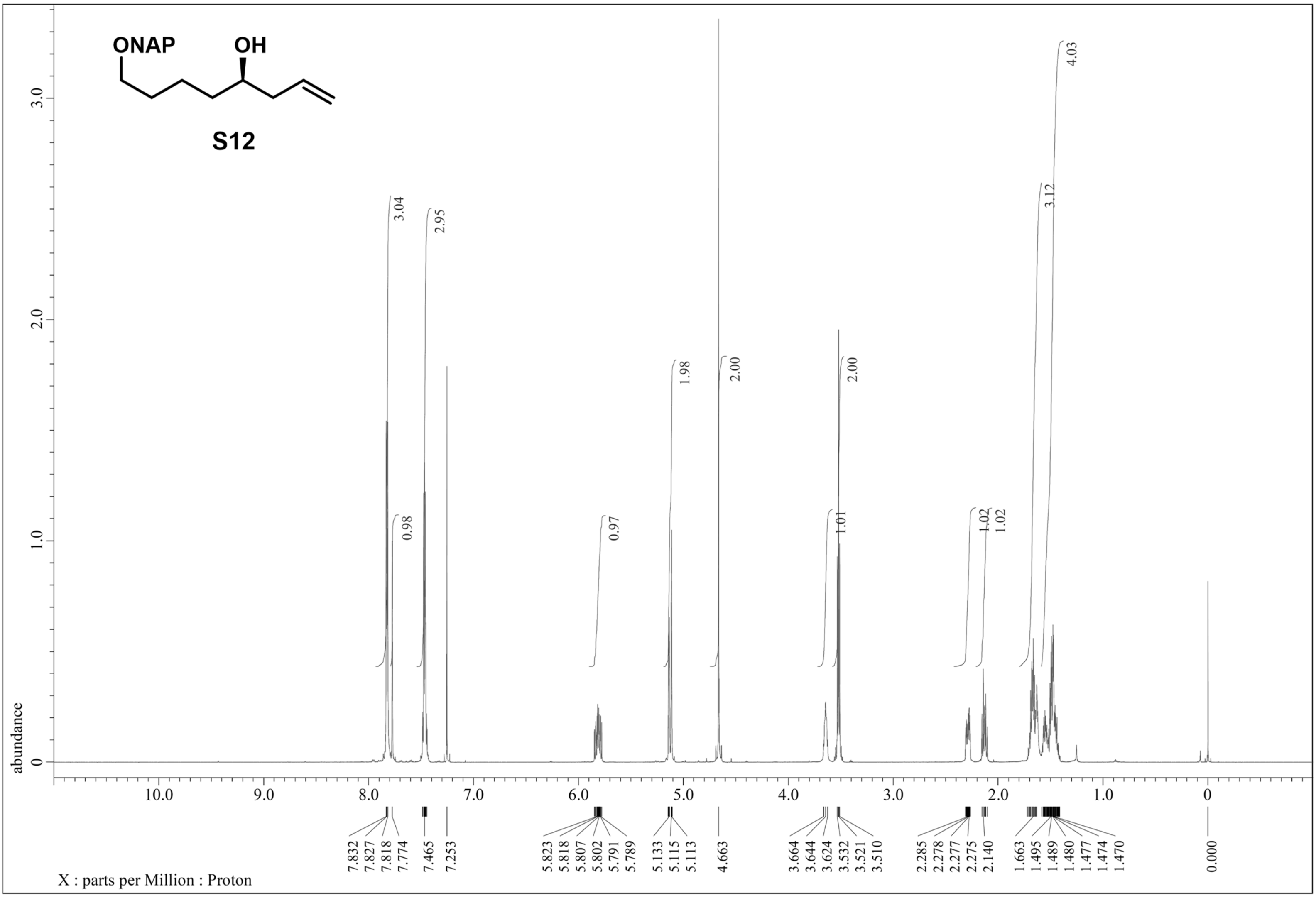

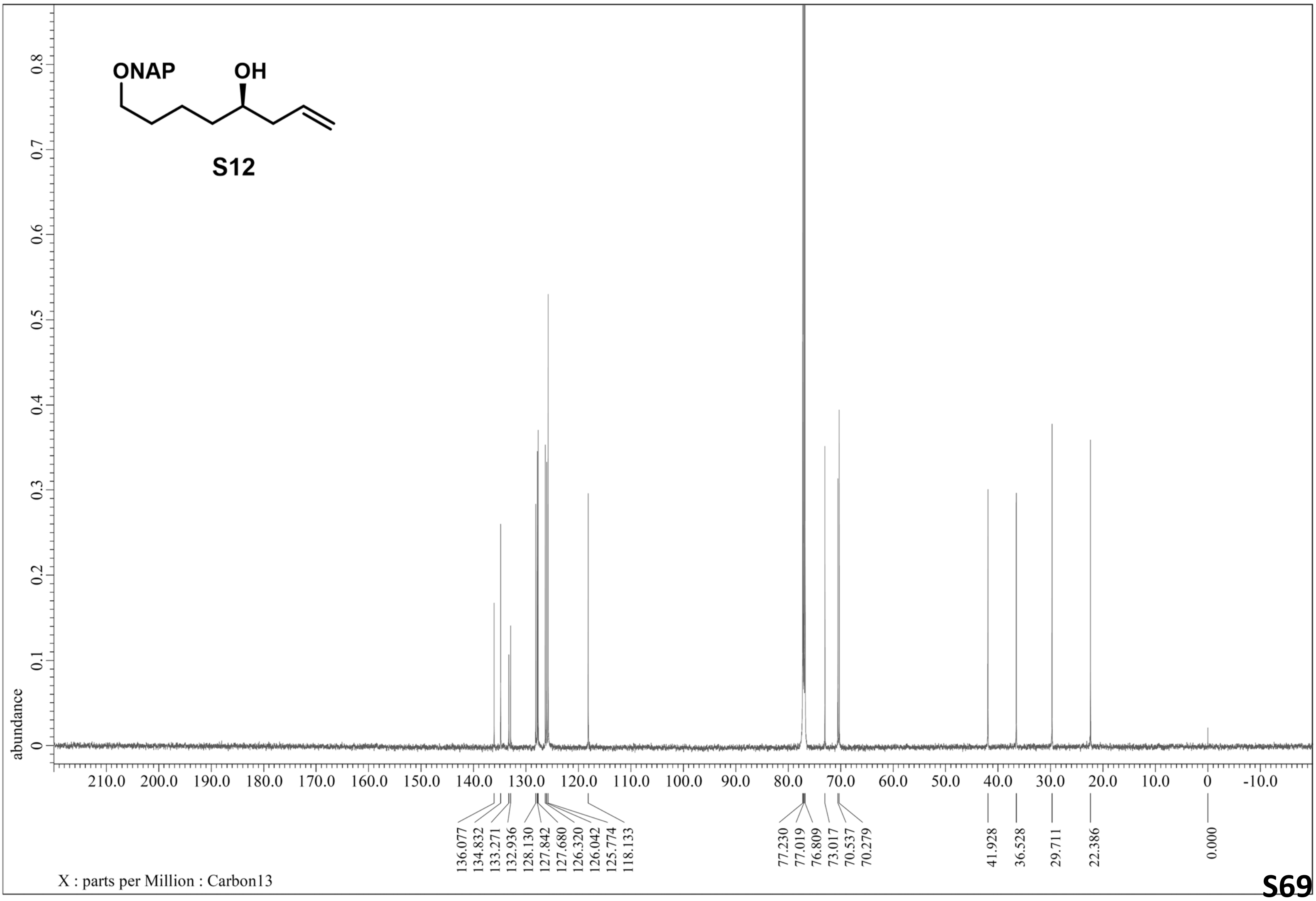

^{1}H NMR (600 MHz, $CDCl_3$) and ^{13}C NMR (151 MHz, $CDCl_3$) spectra of **24**

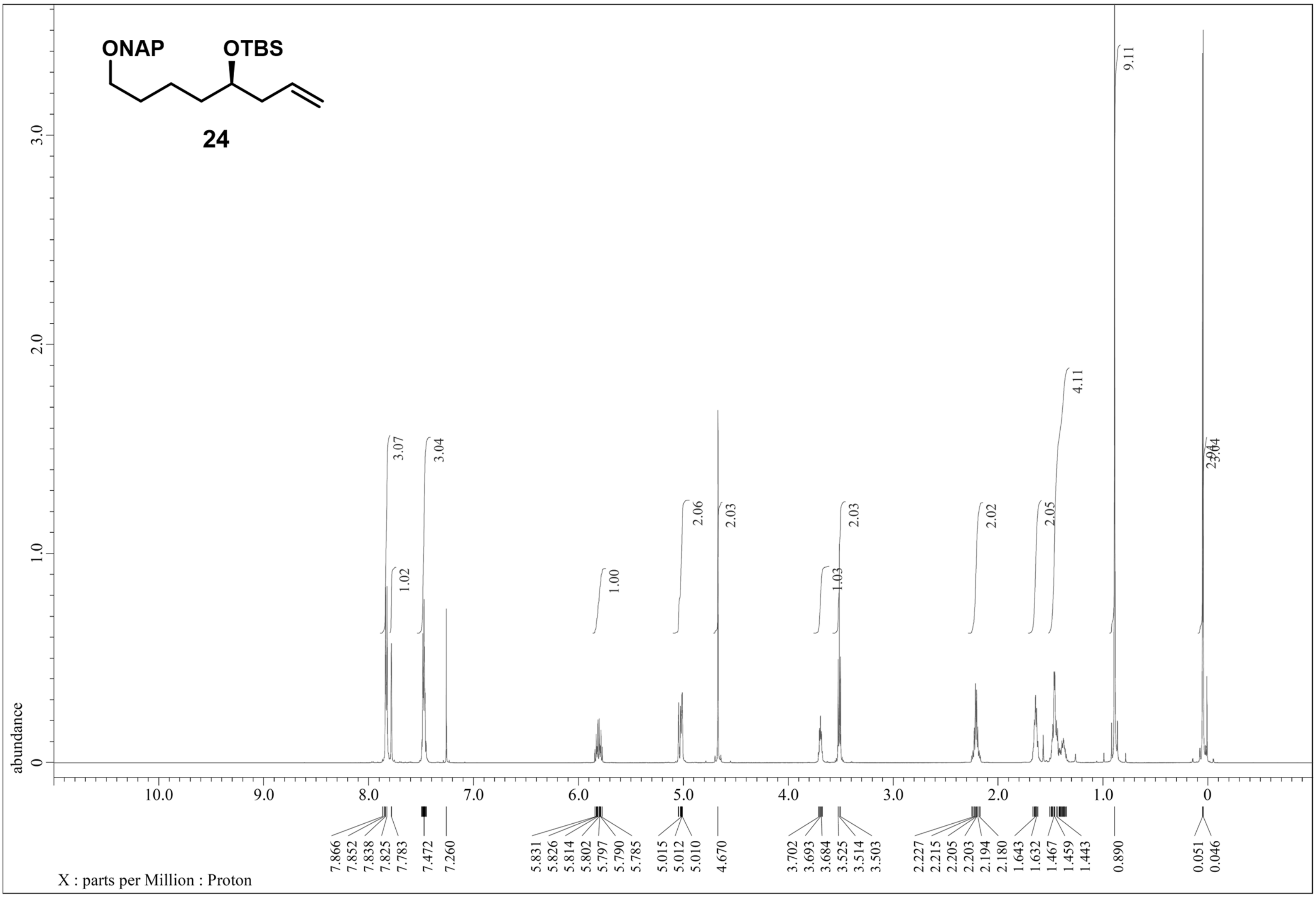

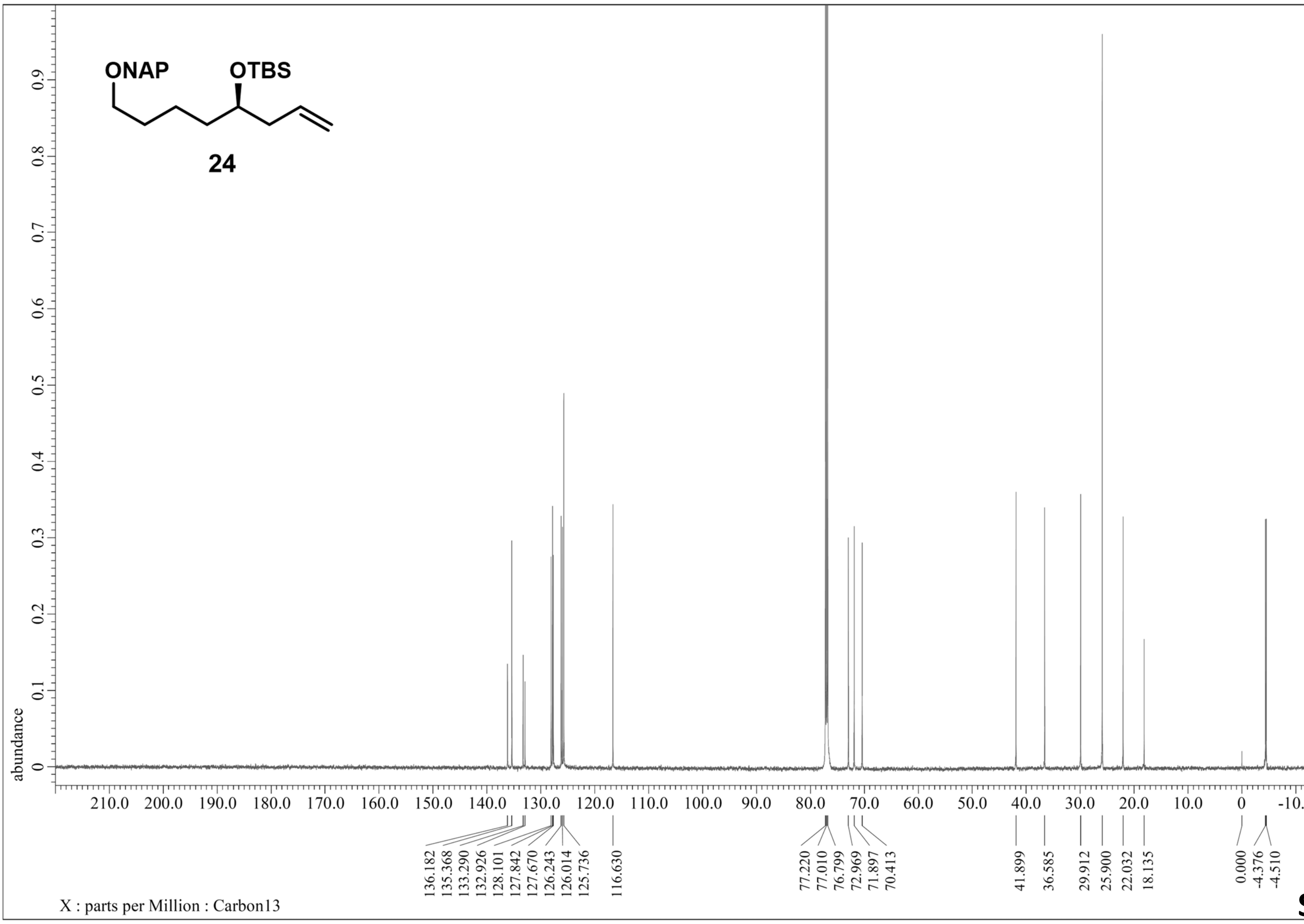

^{1}H NMR (600 MHz, $CDCl_3$) and ^{13}C NMR (151 MHz, $CDCl_3$) spectra of **25**

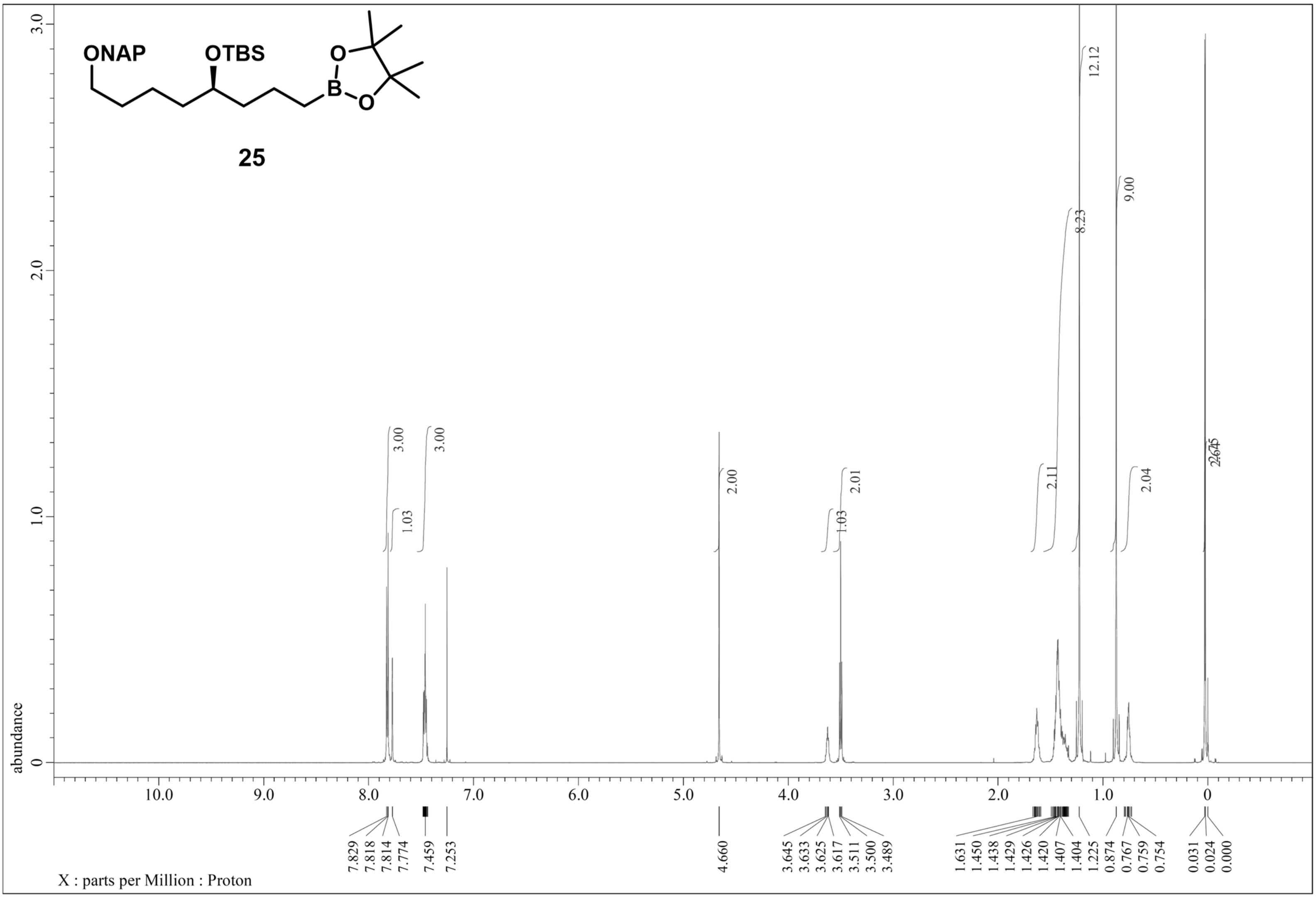

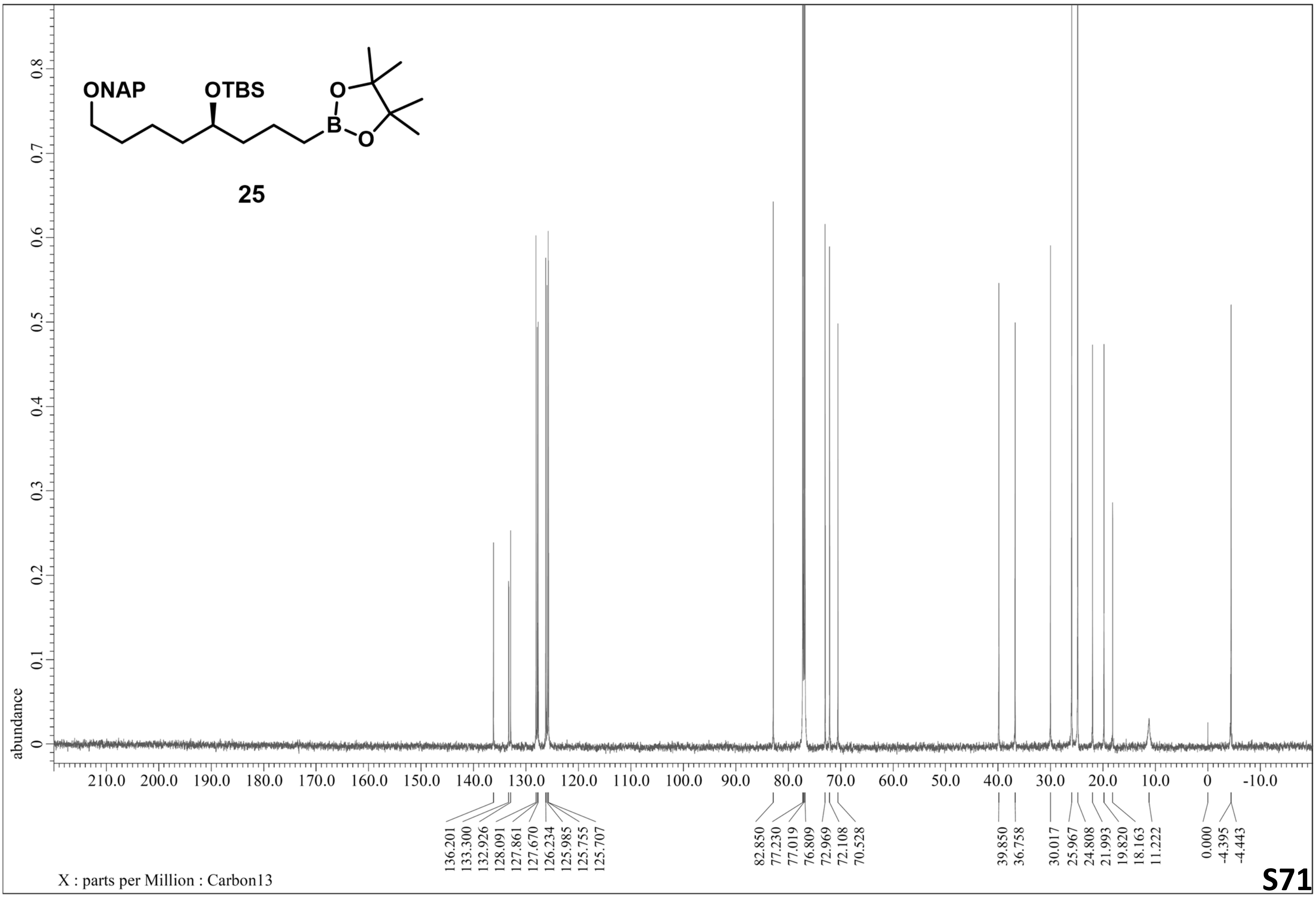

^1H NMR (600 MHz, $CDCl_3$) and ^{13}C NMR (151 MHz, $CDCl_3$) spectra of **8**

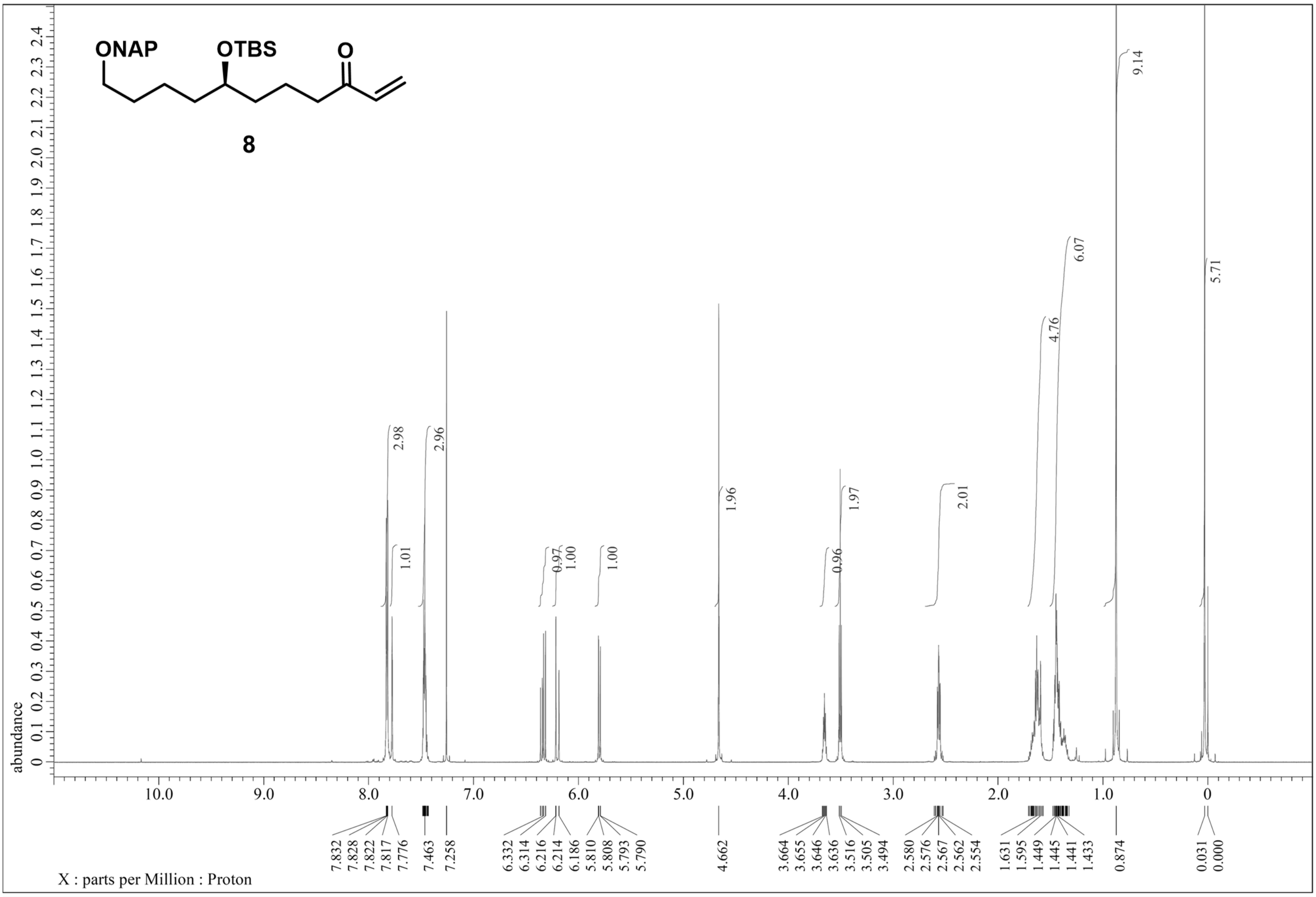

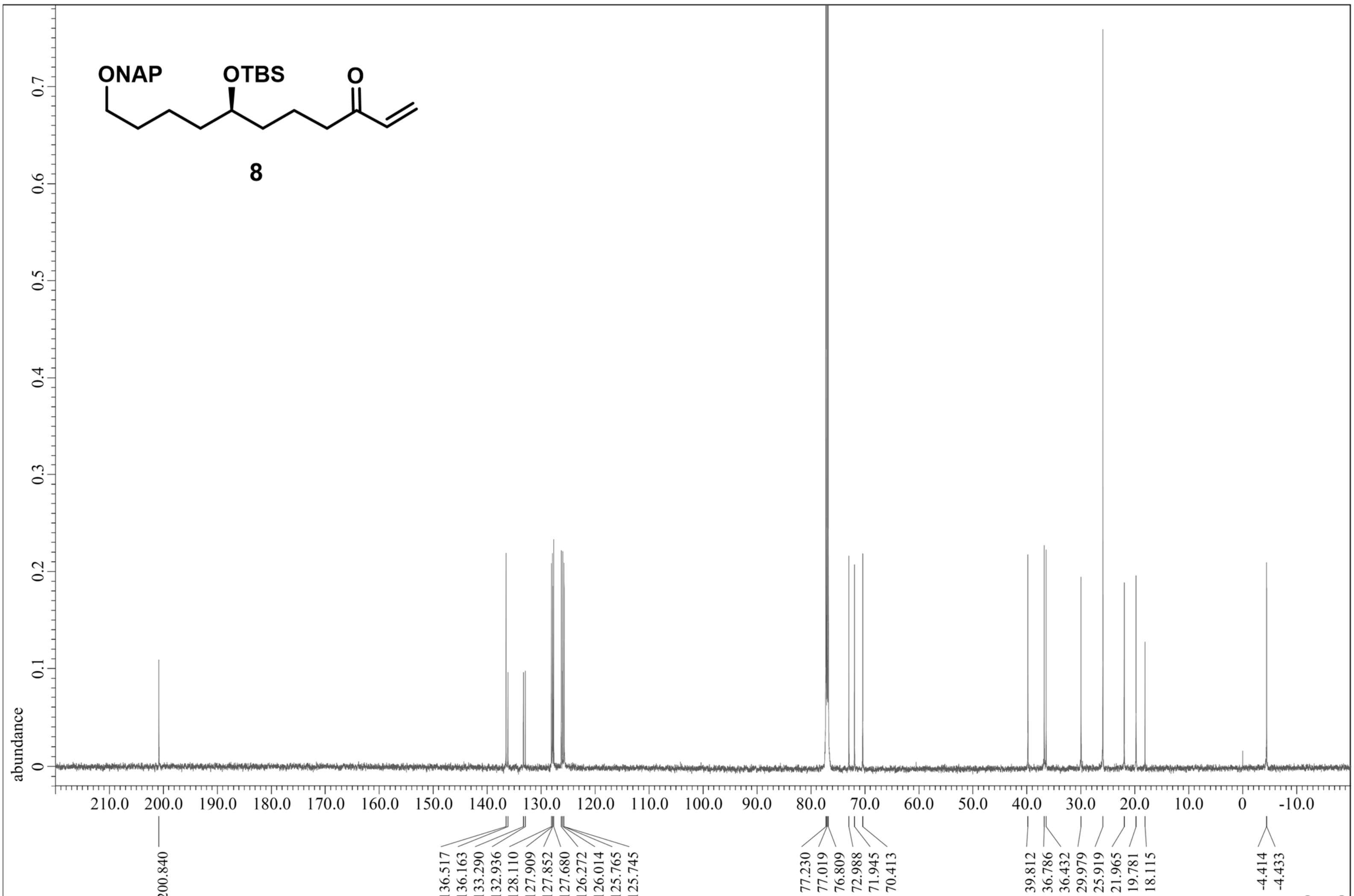

^{1}H NMR (600 MHz, $CDCl_3$) and ^{13}C NMR (151 MHz, $CDCl_3$) spectra of **20**

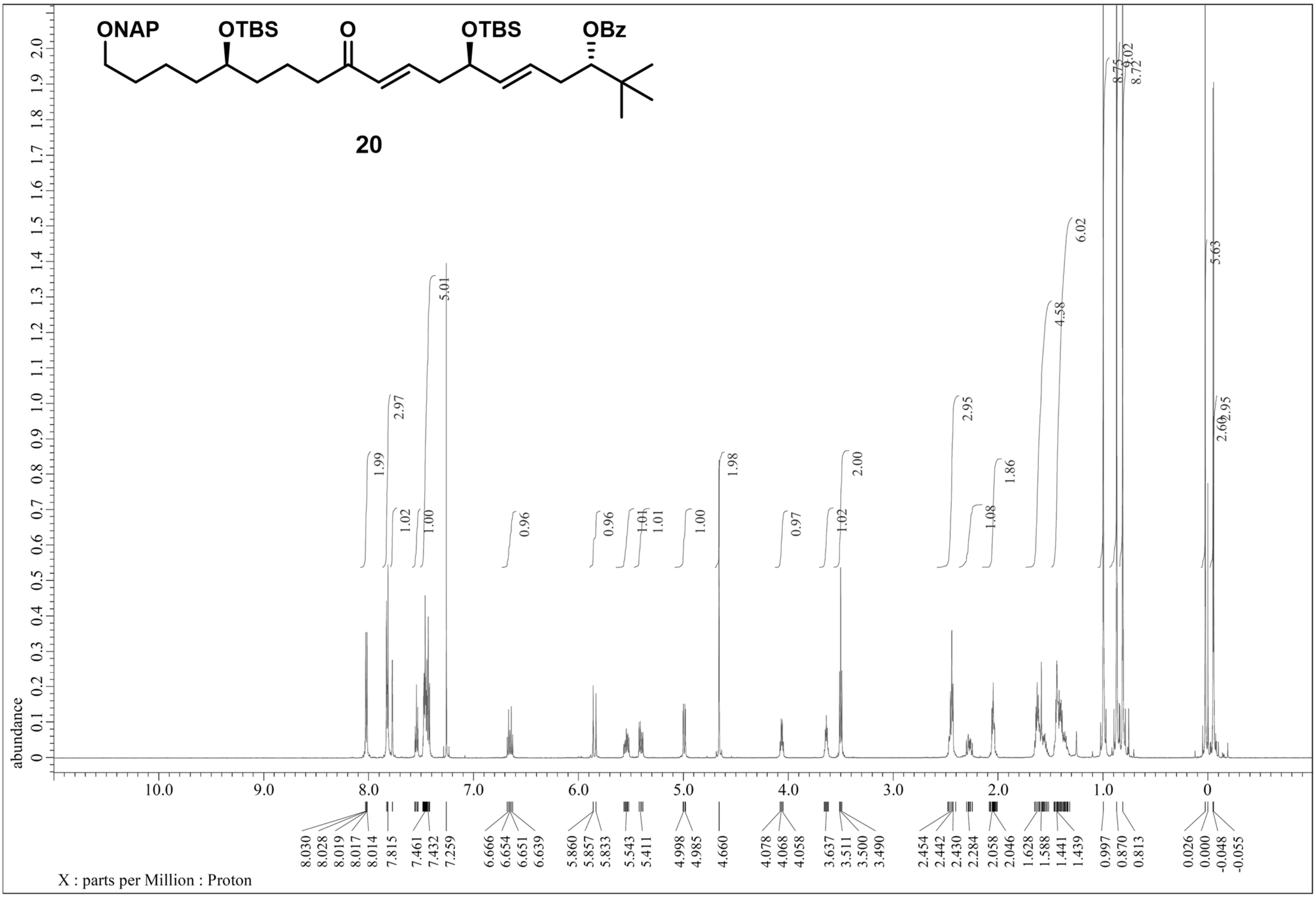

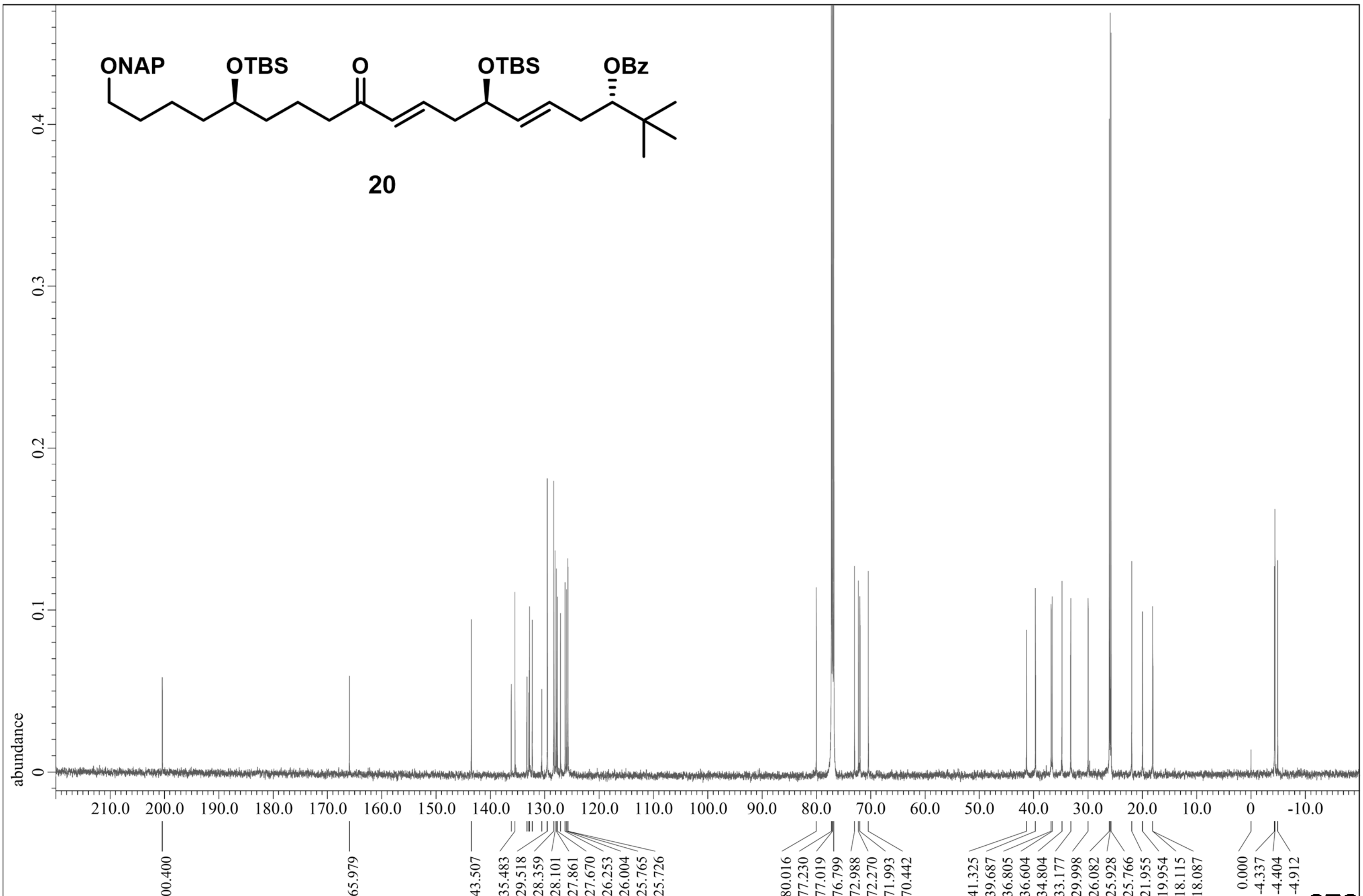

^{1}H NMR (600 MHz, $CDCl_3$) and ^{13}C NMR (151 MHz, $CDCl_3$) spectra of **S13**

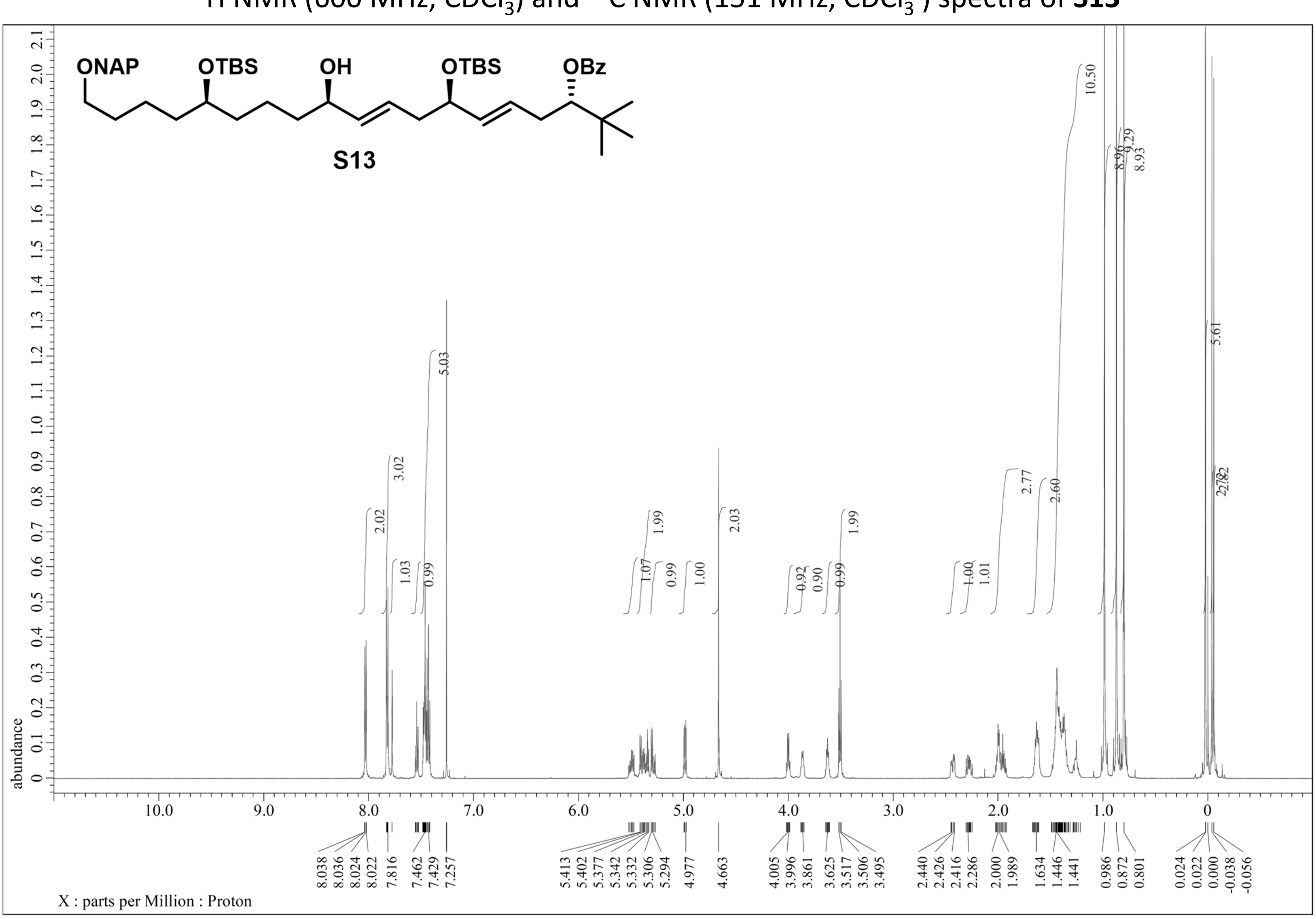

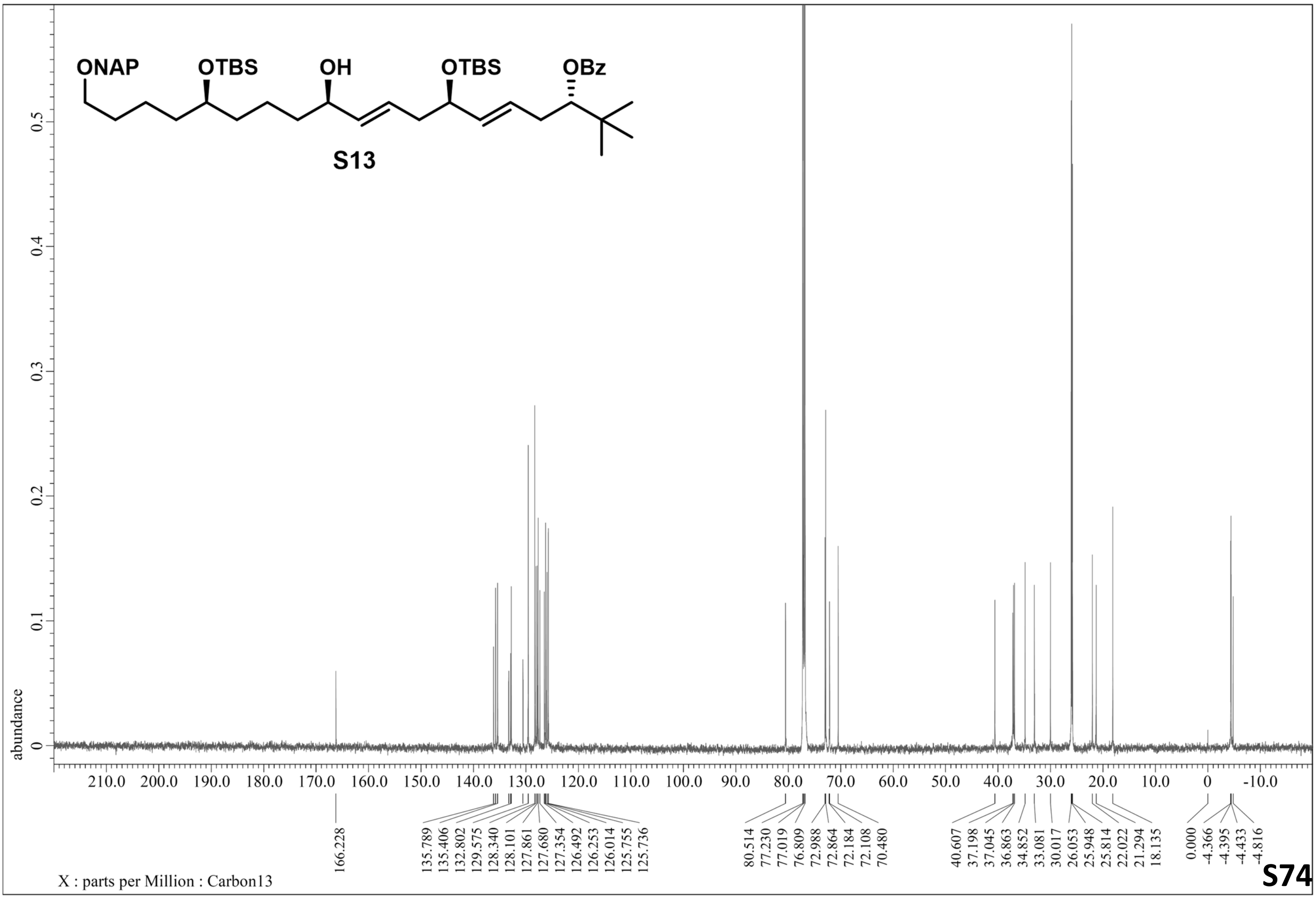

^{1}H NMR (600 MHz, $CDCl_3$) and ^{13}C NMR (151 MHz, $CDCl_3$) spectra of **S14**

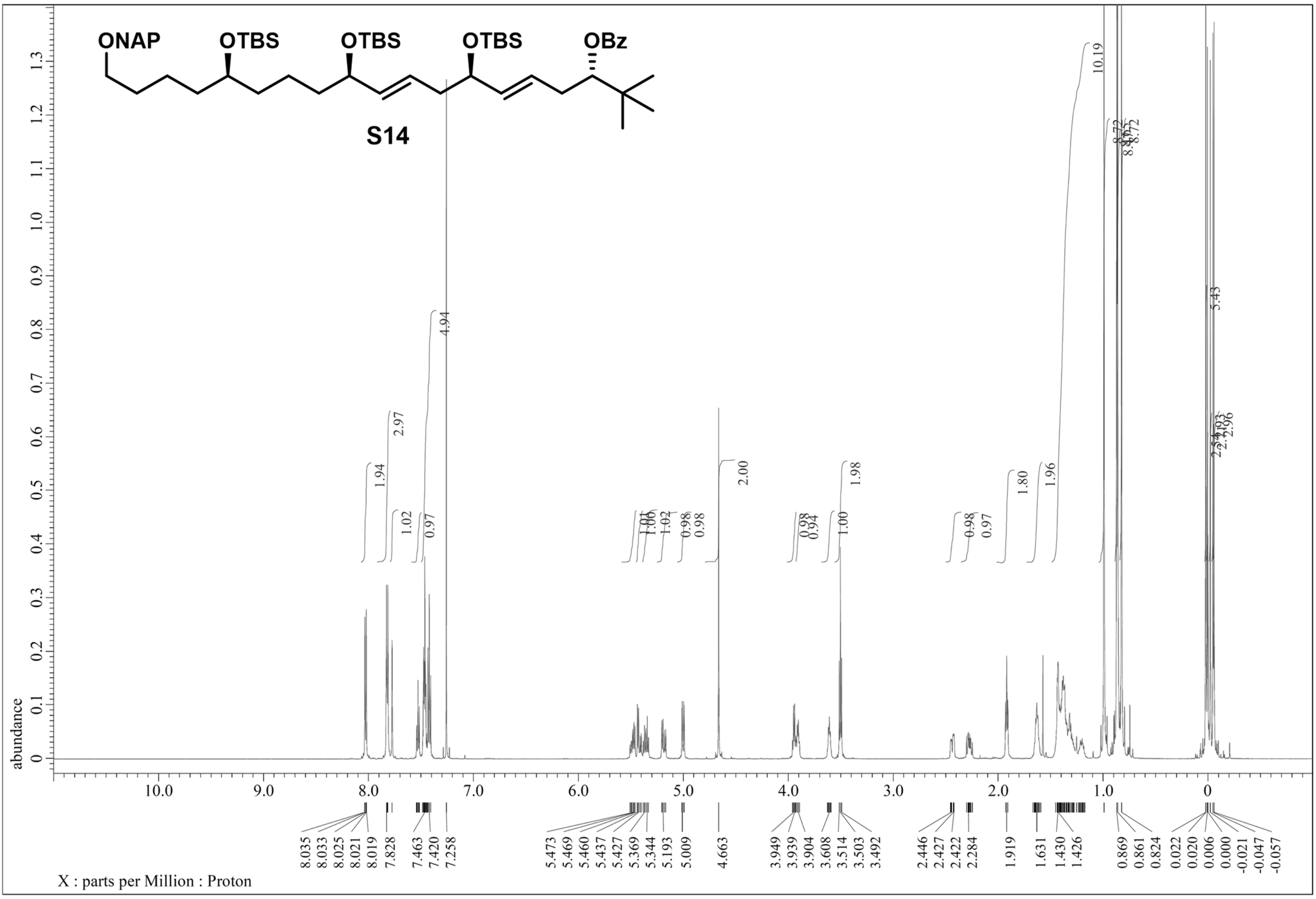

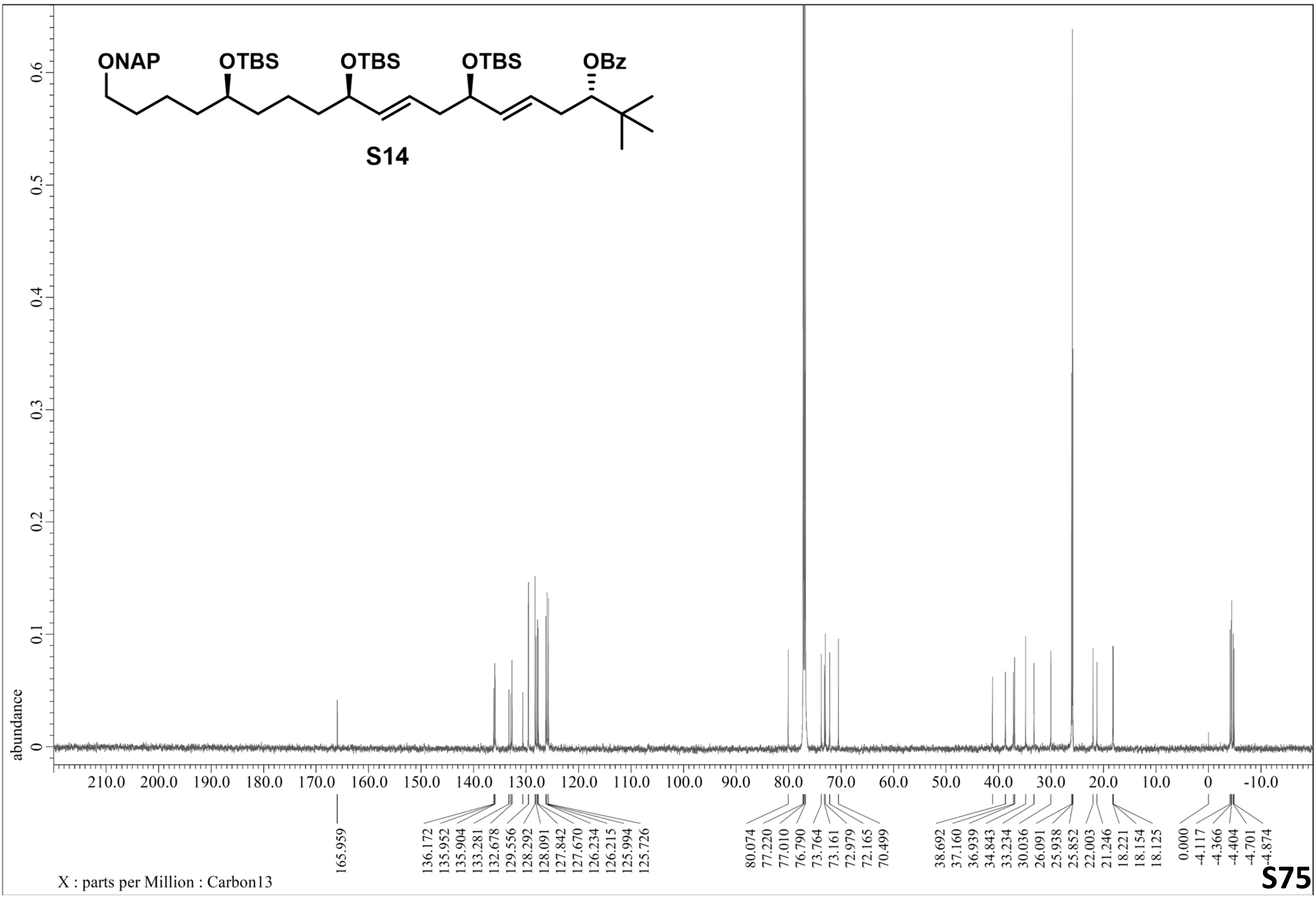

^{1}H NMR (600 MHz, $CDCl_3$) and ^{13}C NMR (151 MHz, $CDCl_3$) spectra of **21**

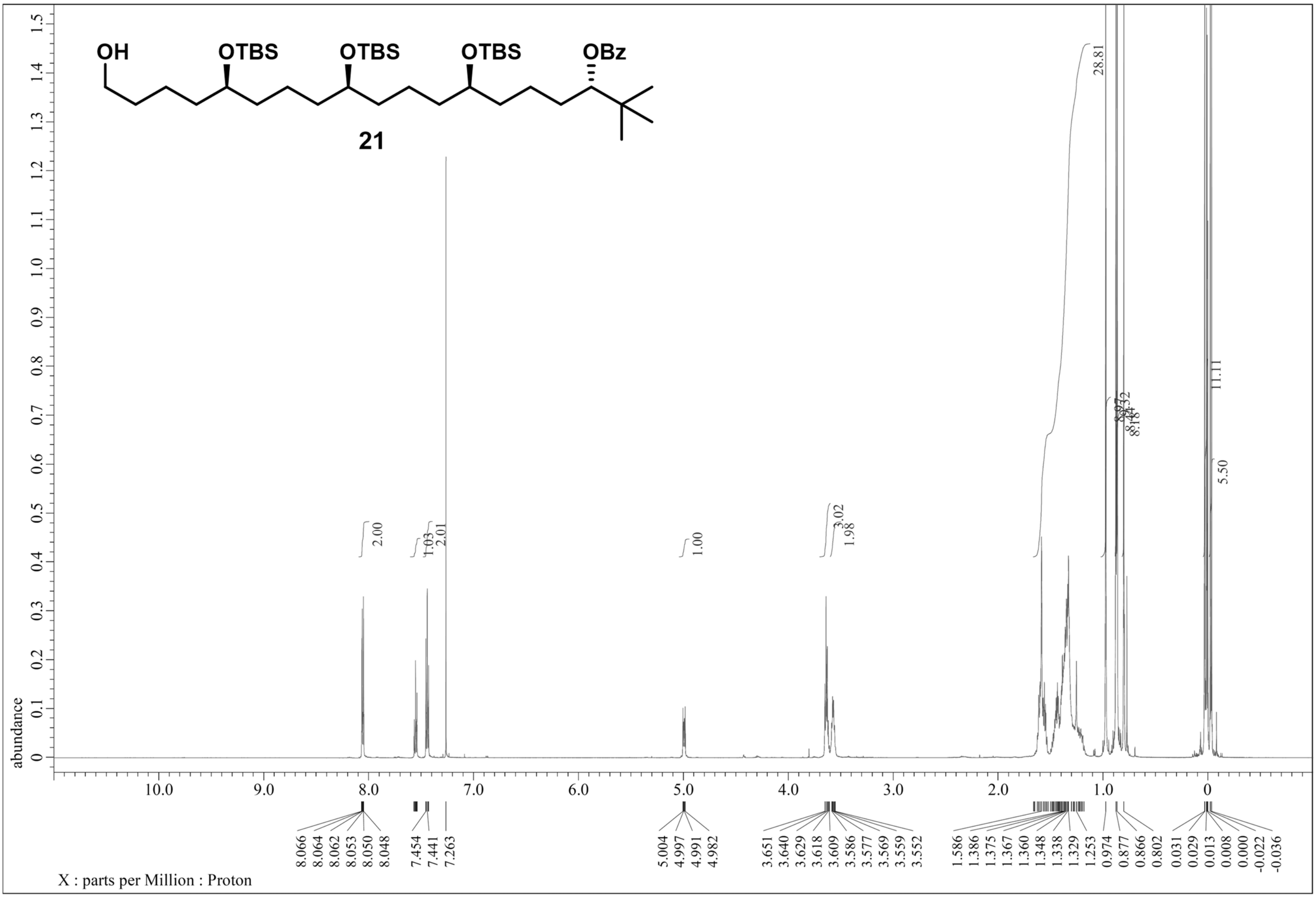

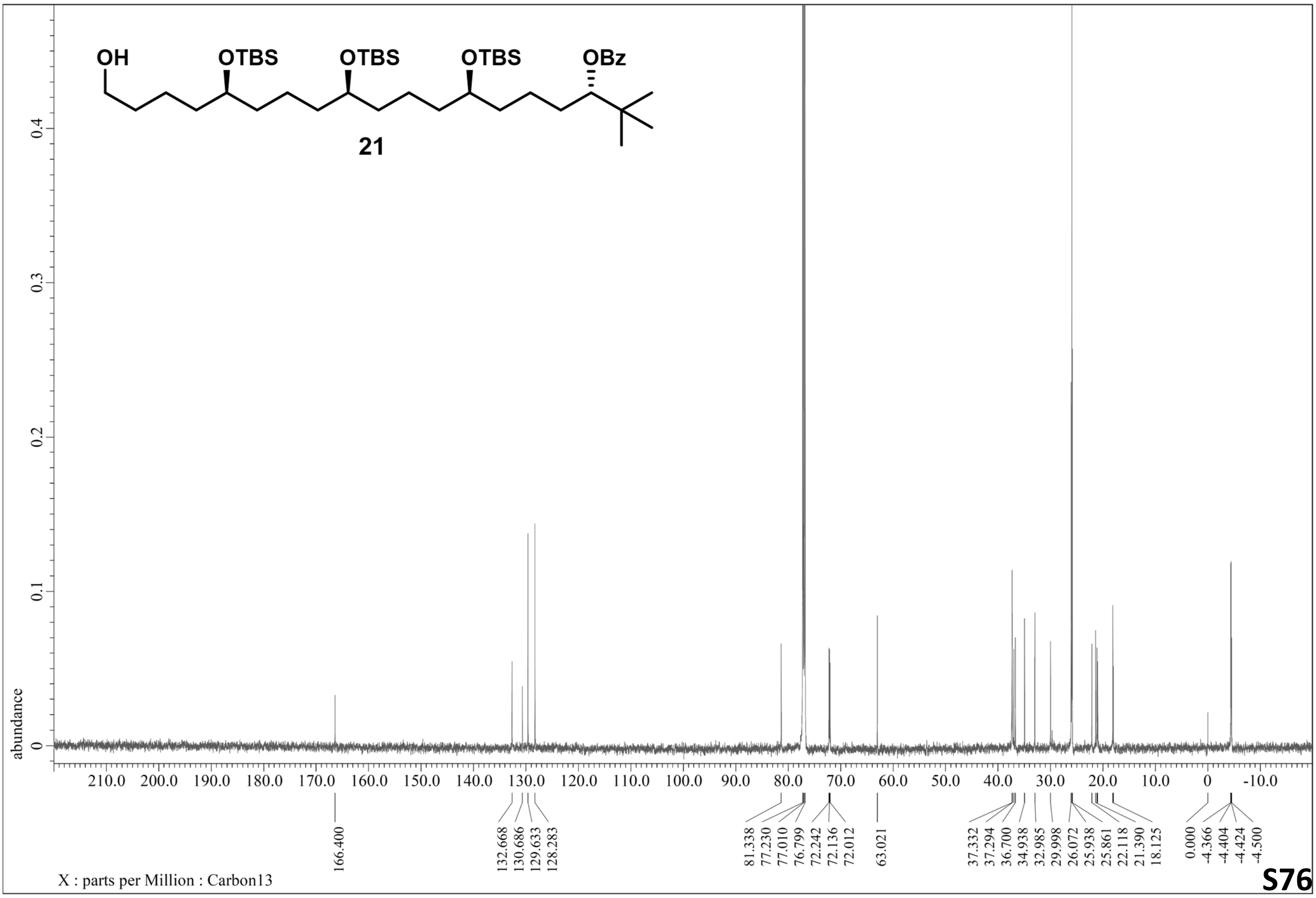

^1H NMR (600 MHz, $CDCl_3$) and ^{13}C NMR (151 MHz, $CDCl_3$) spectra of **22**

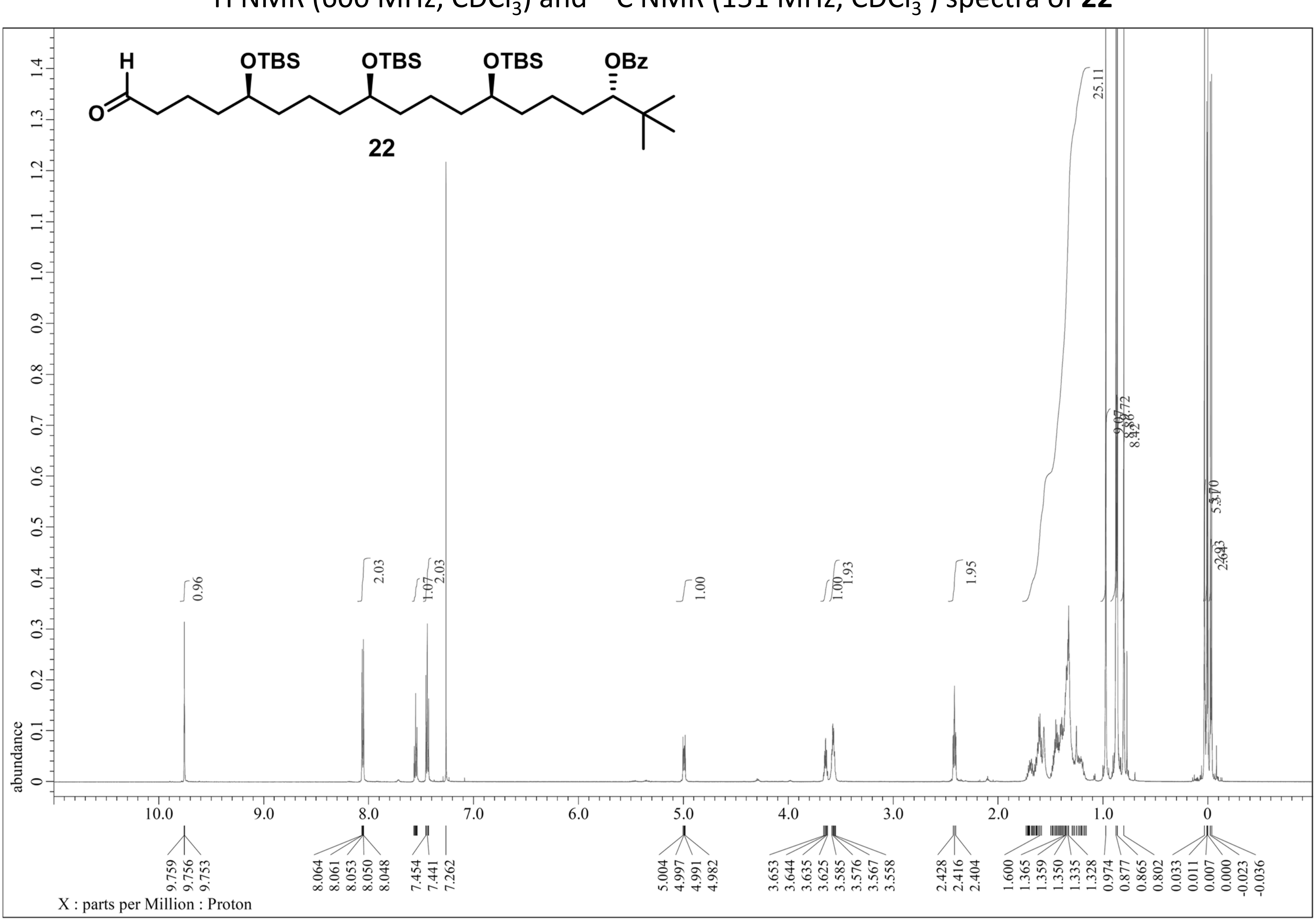

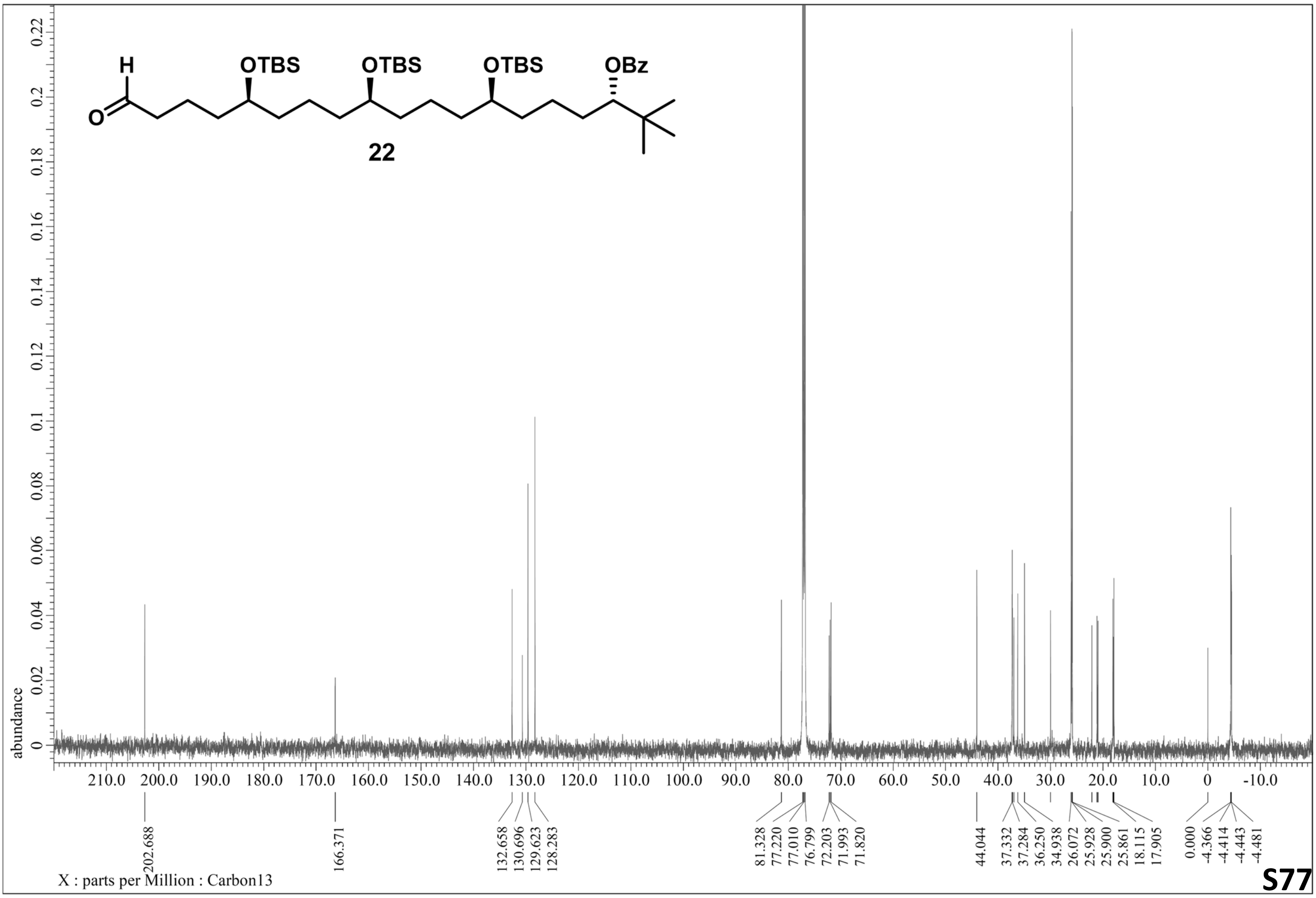

^1H NMR (600 MHz, $CDCl_3$) and ^{13}C NMR (151 MHz, $CDCl_3$) spectra of **S15**

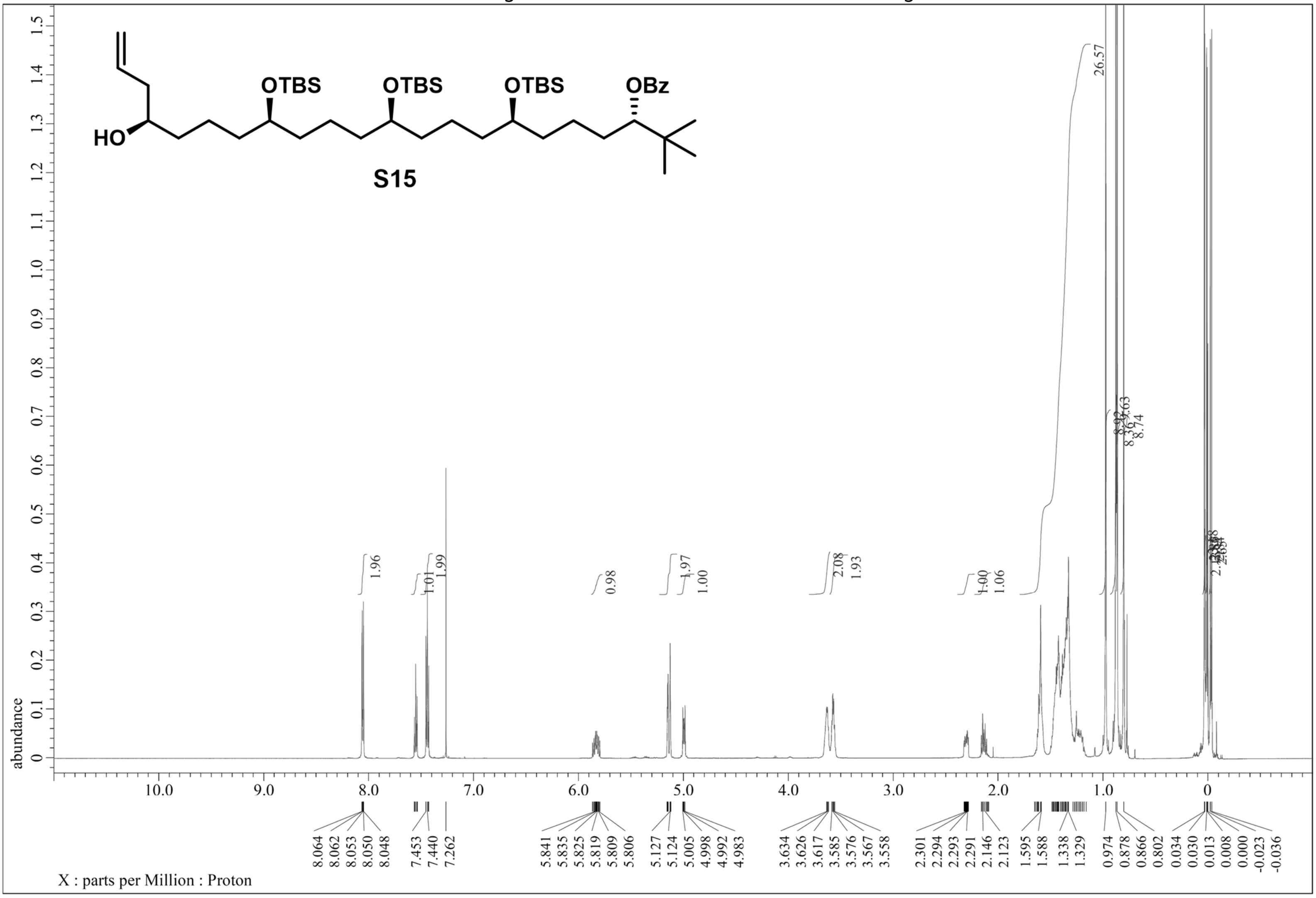

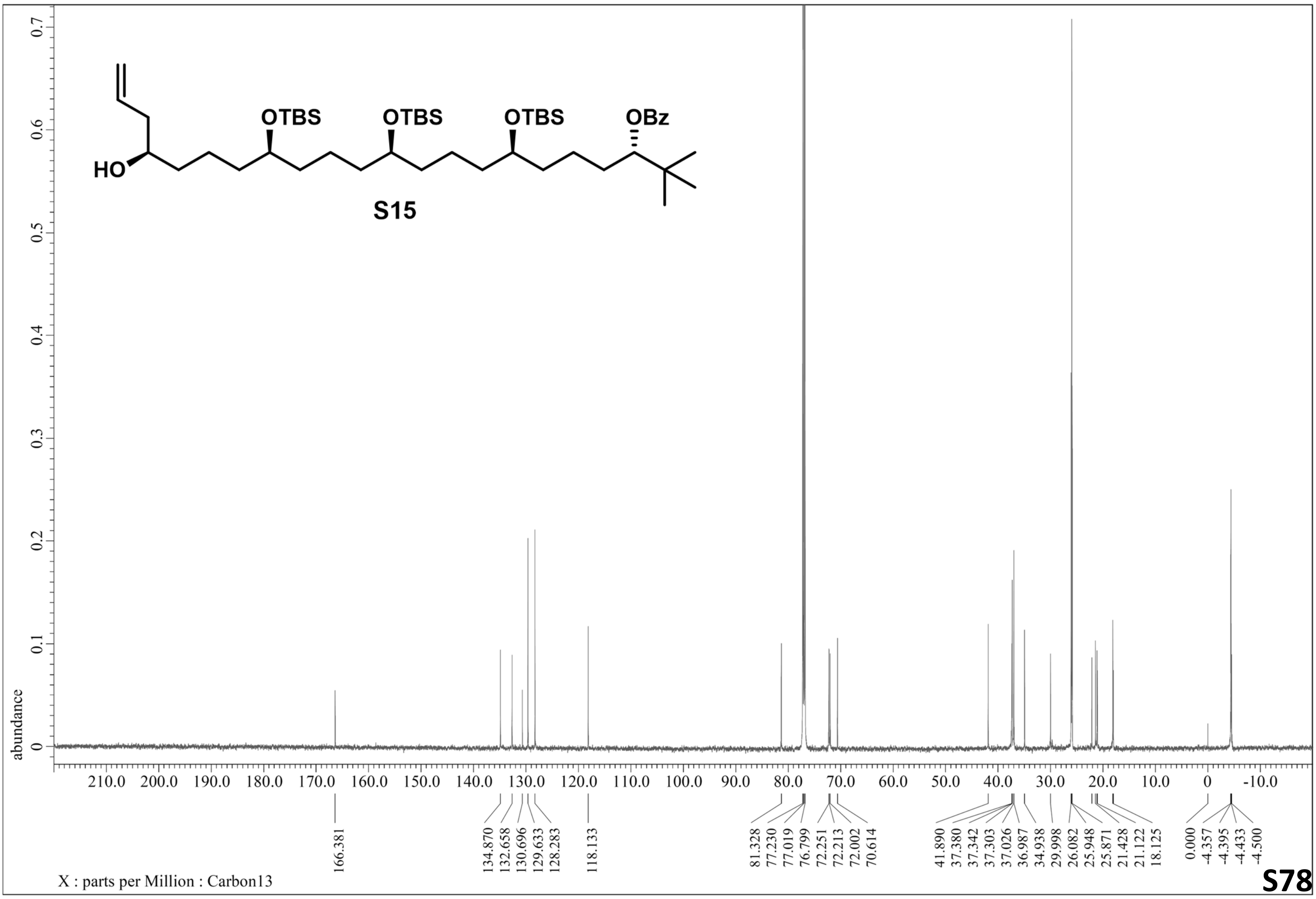

^{1}H NMR (600 MHz, $CDCl_3$) and ^{13}C NMR (151 MHz, $CDCl_3$) spectra of **S16**

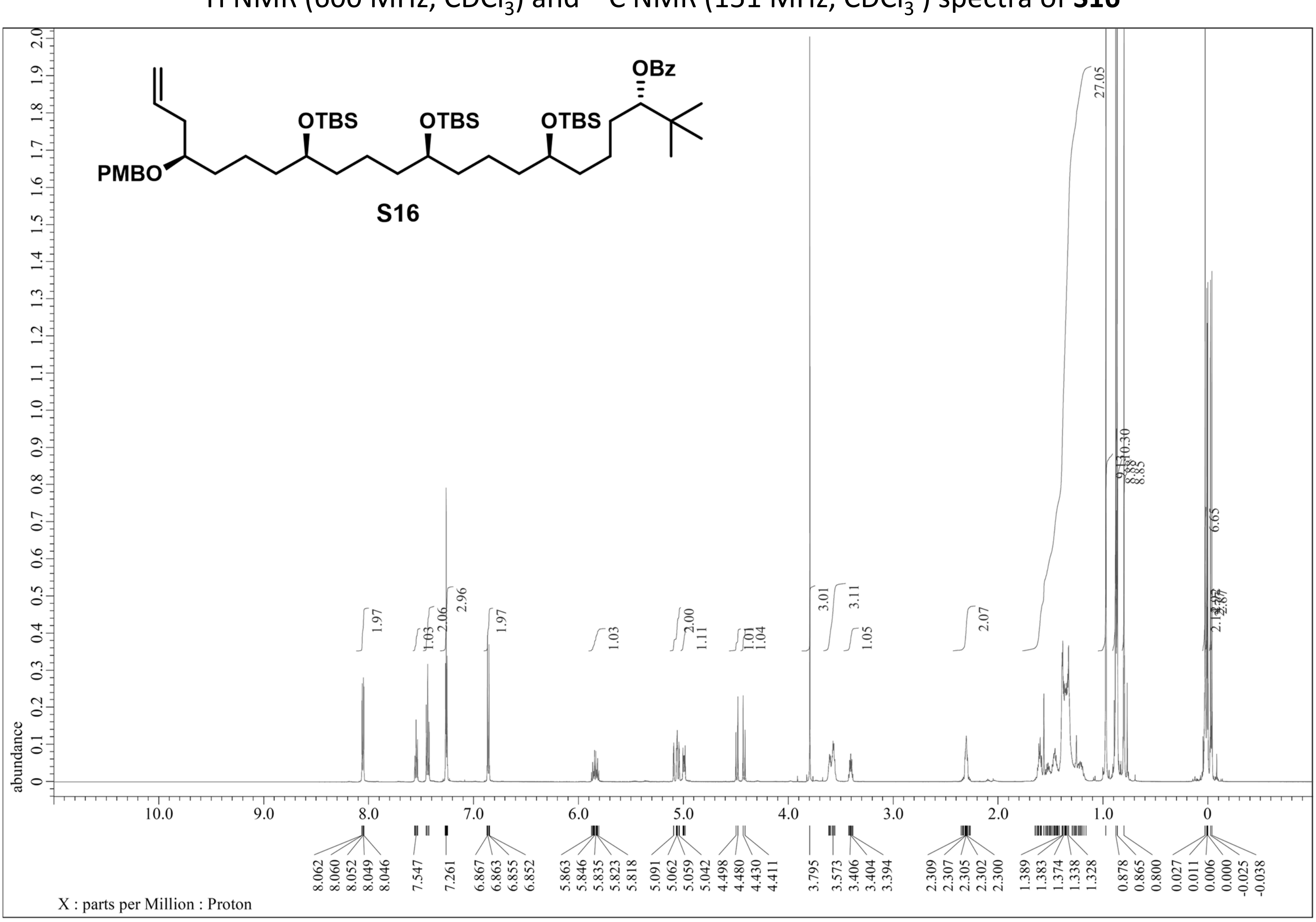

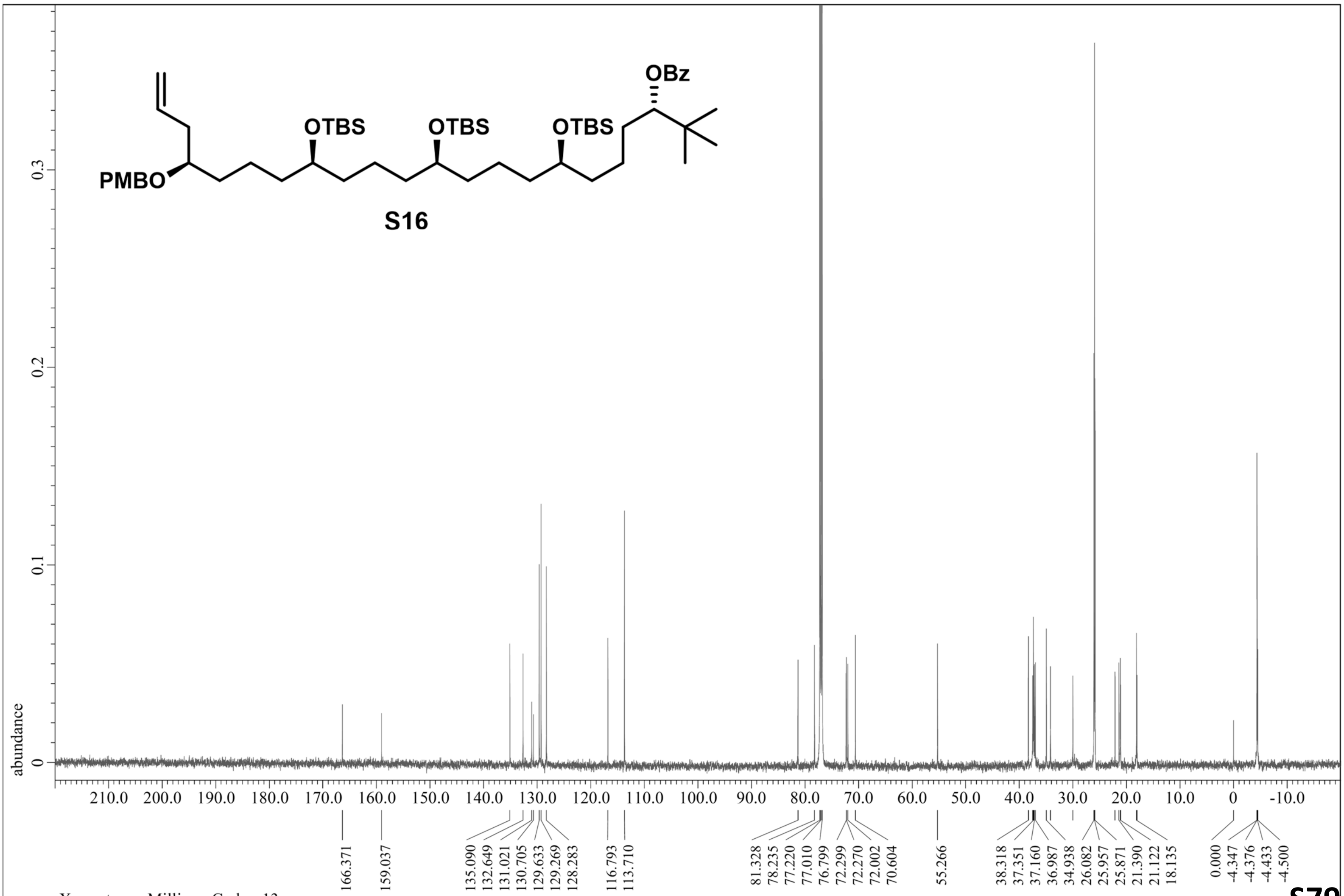

^{1}H NMR (600 MHz, $CDCl_3$) and ^{13}C NMR (151 MHz, $CDCl_3$) spectra of **5**

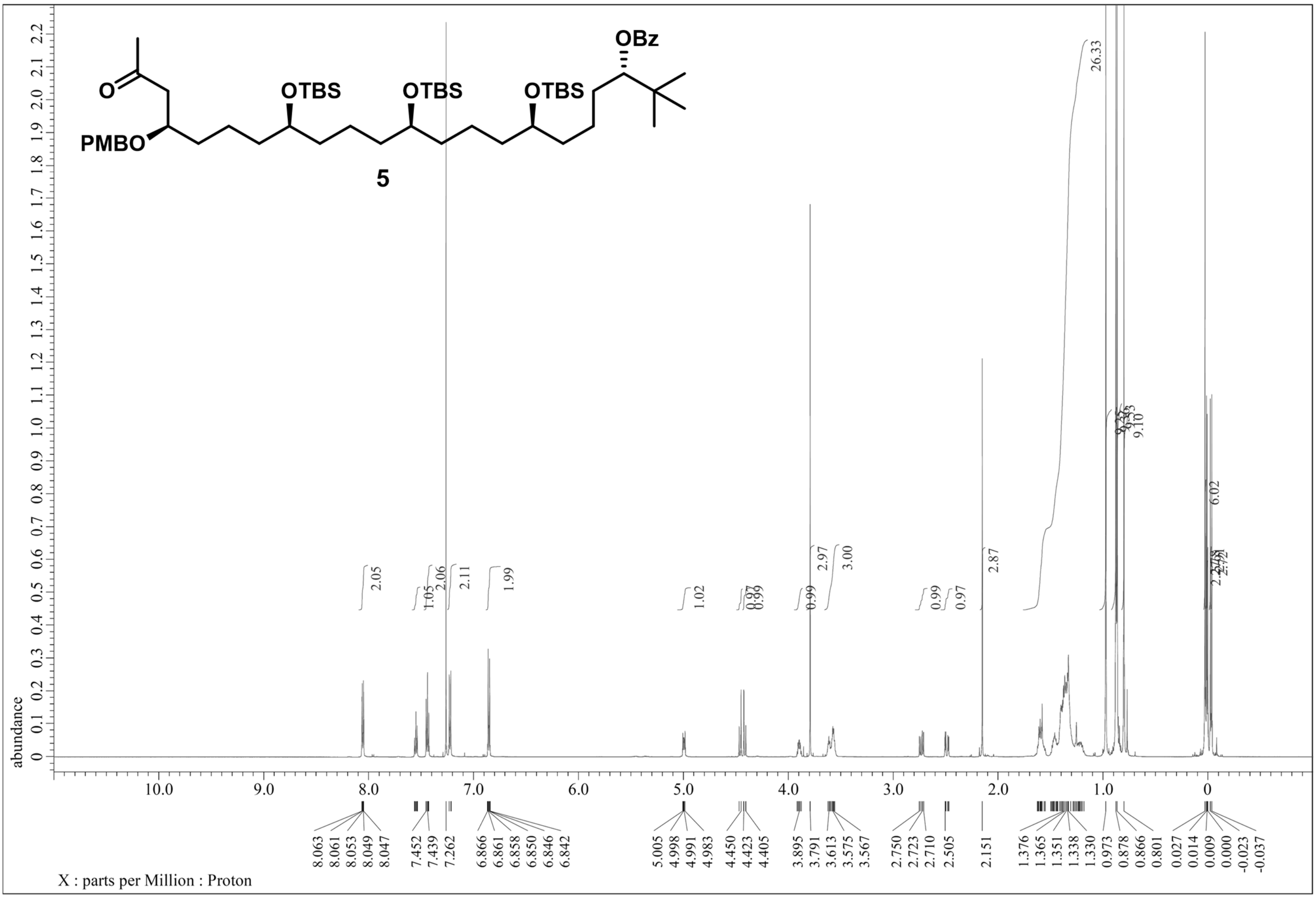

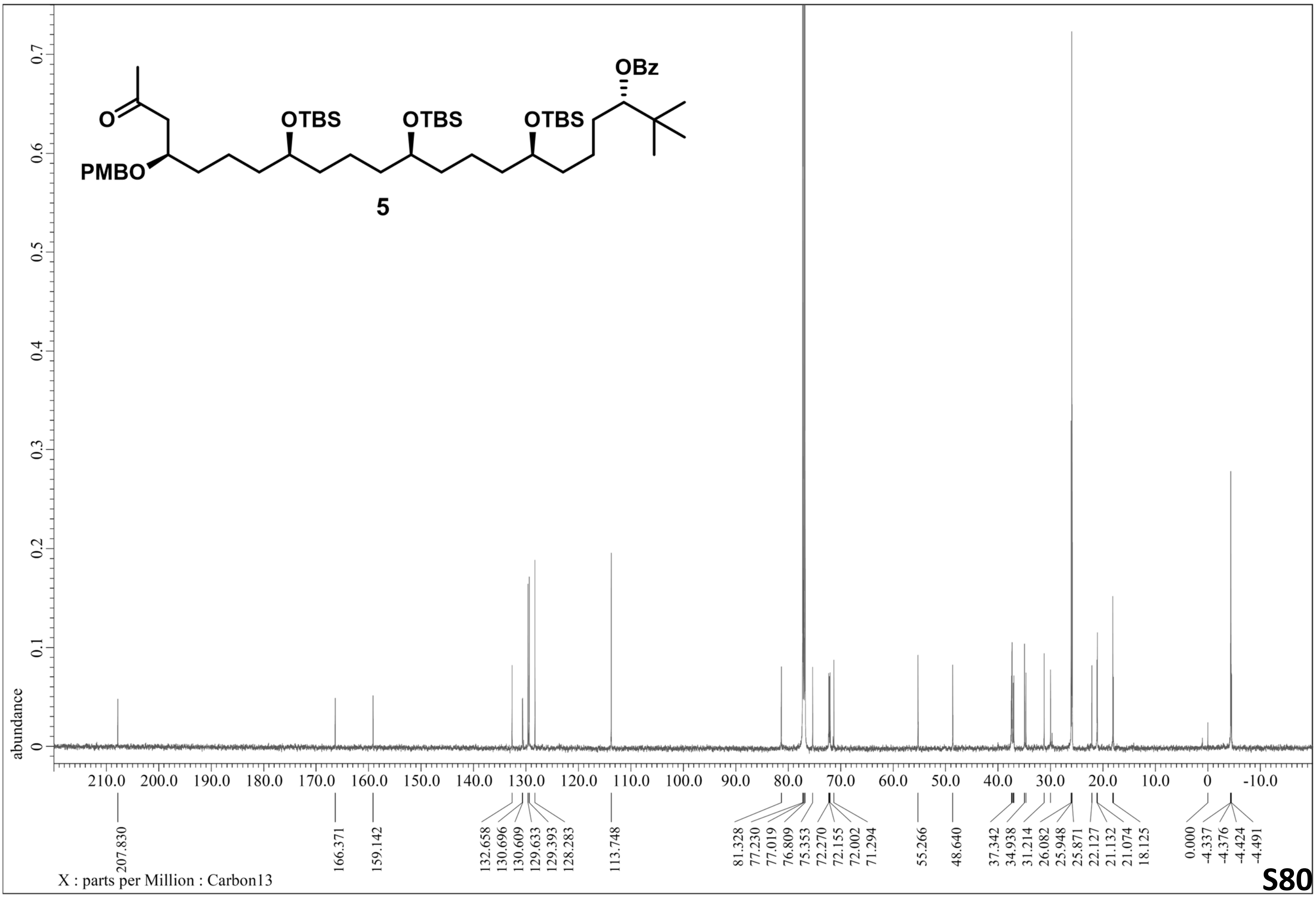

^1H NMR (600 MHz, $CDCl_3$) and ^{13}C NMR (151 MHz, $CDCl_3$) spectra of **26**

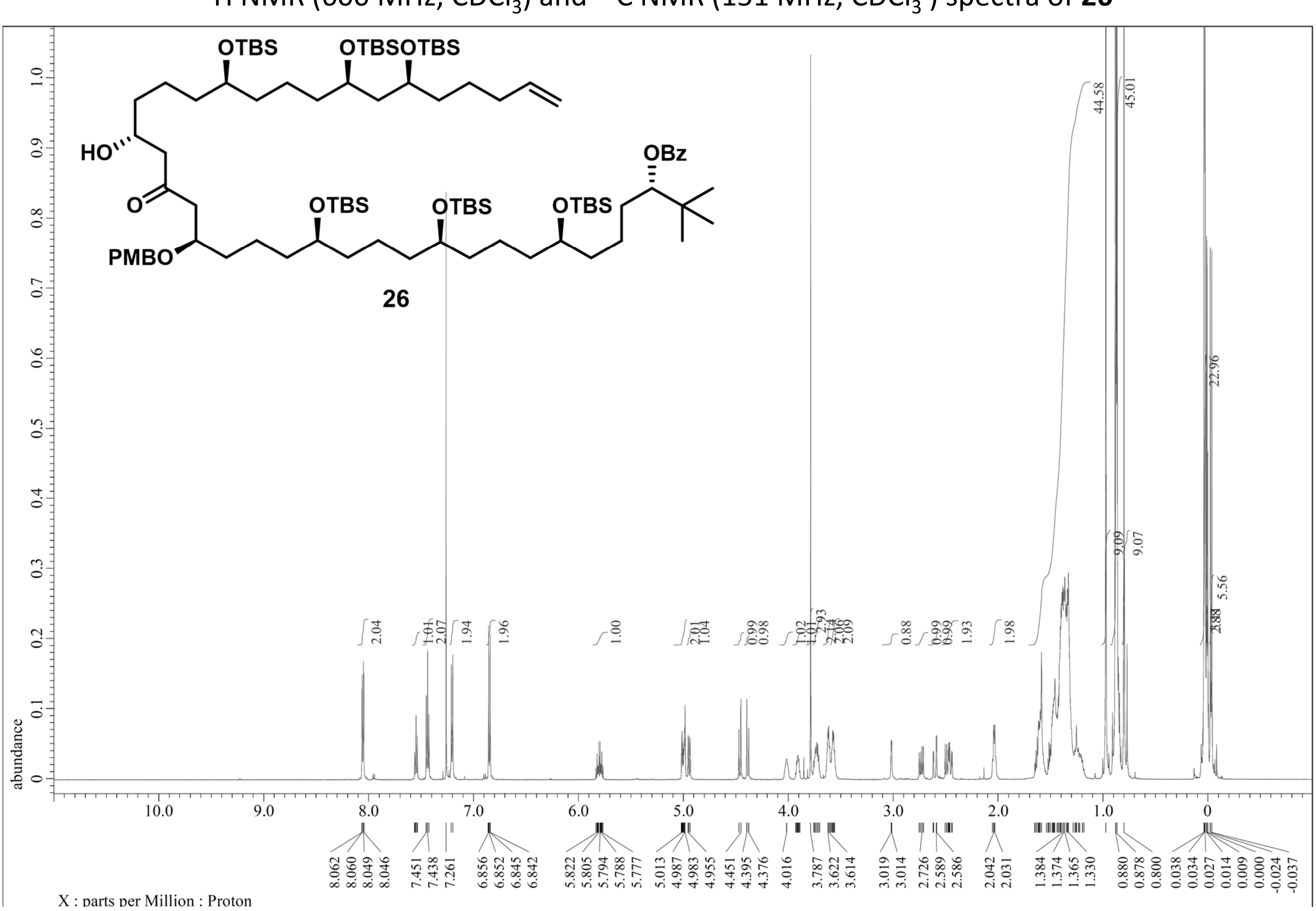

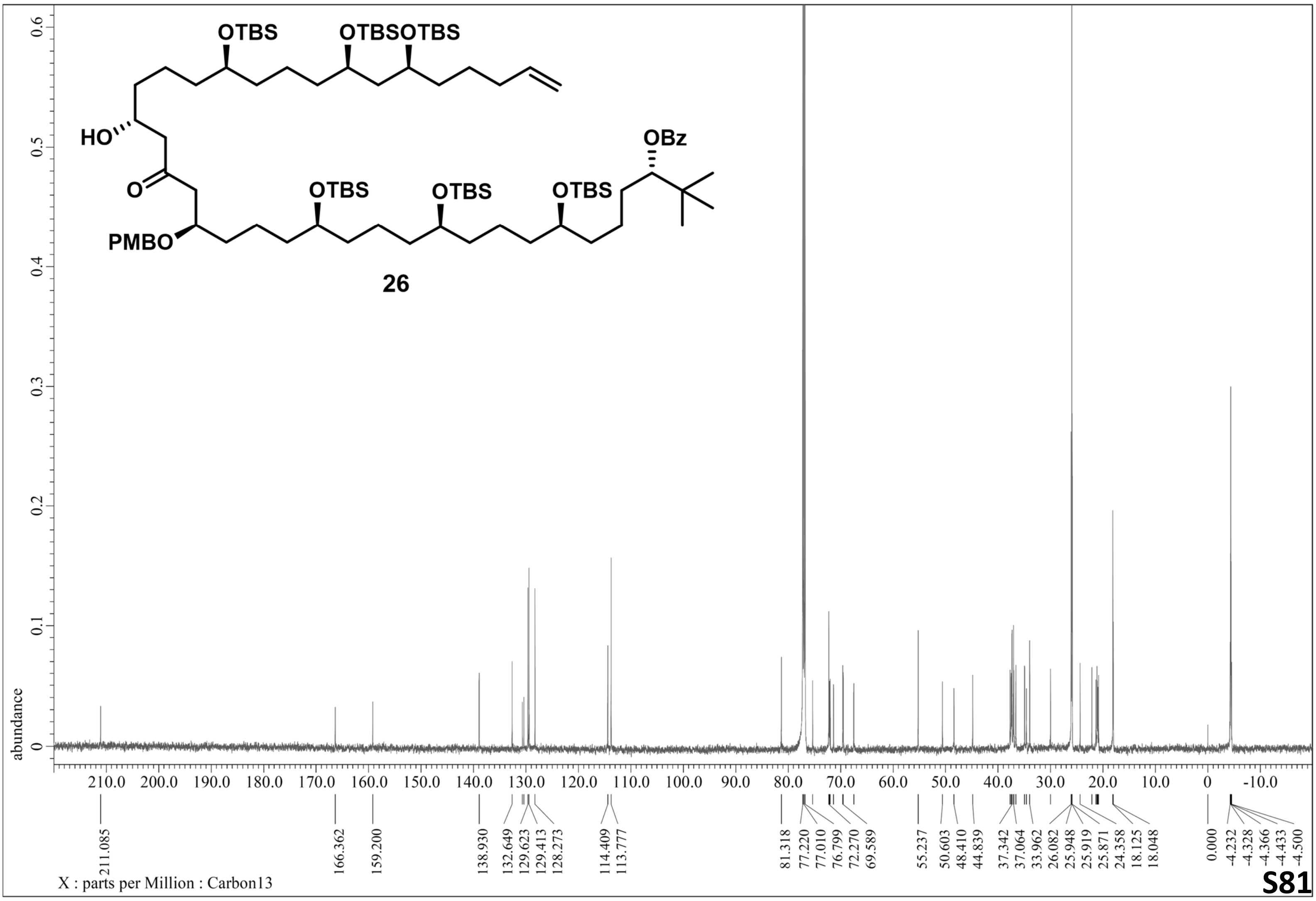

^{1}H NMR (600 MHz, $CDCl_3$) and ^{13}C NMR (151 MHz, $CDCl_3$) spectra of **S17**

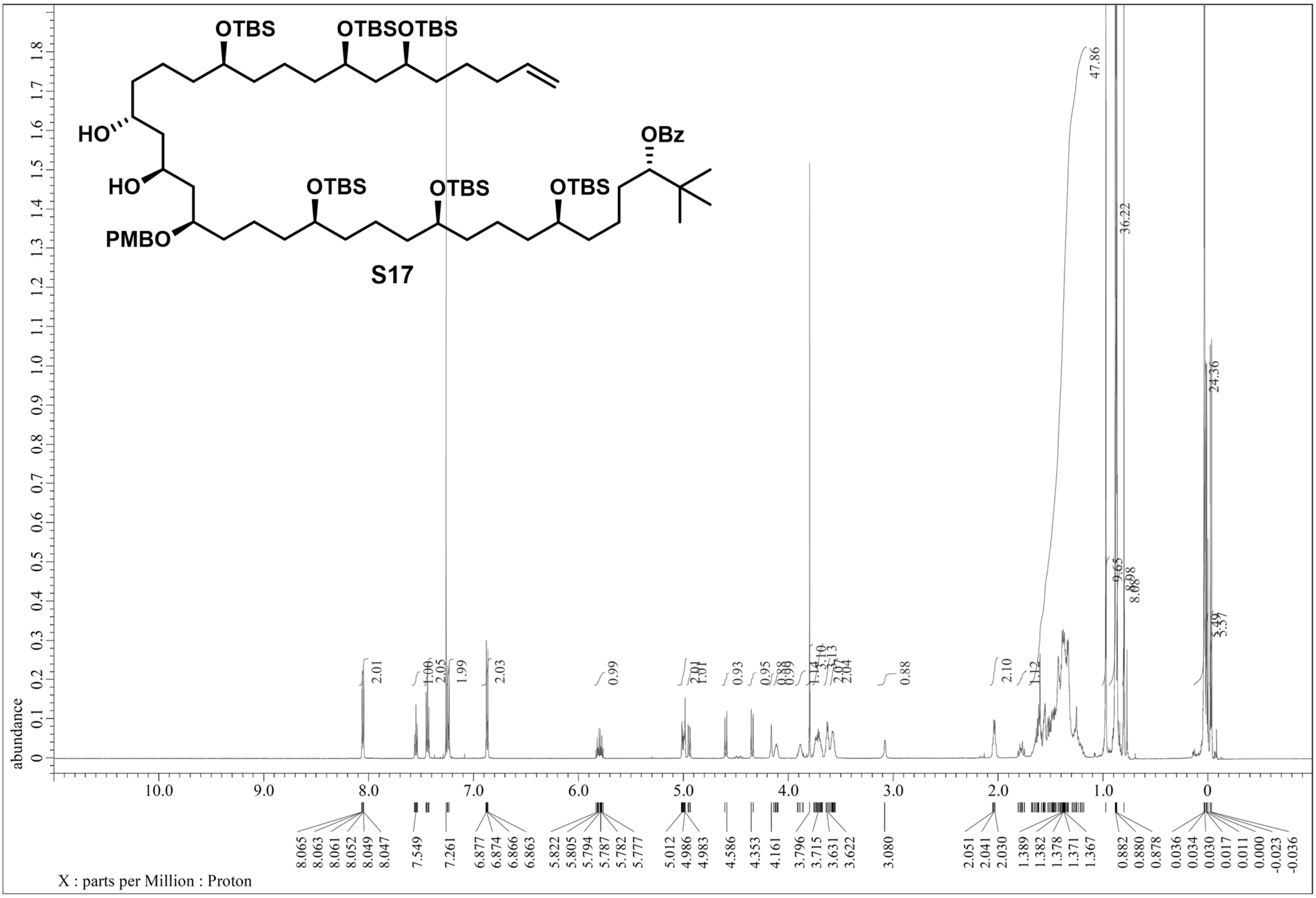

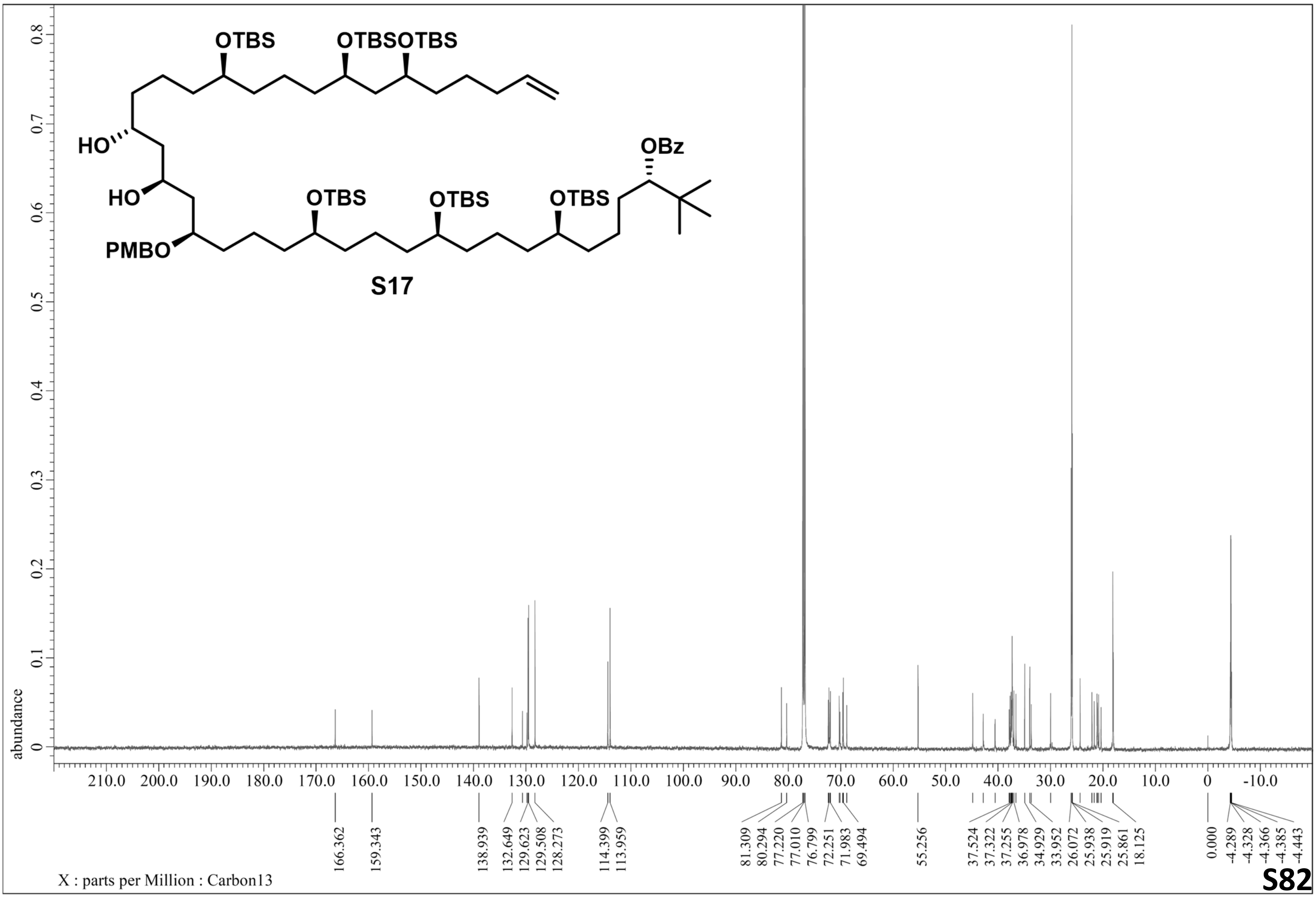

^{1}H NMR (600 MHz, $CDCl_3$) and ^{13}C NMR (151 MHz, $CDCl_3$) spectra of **27**

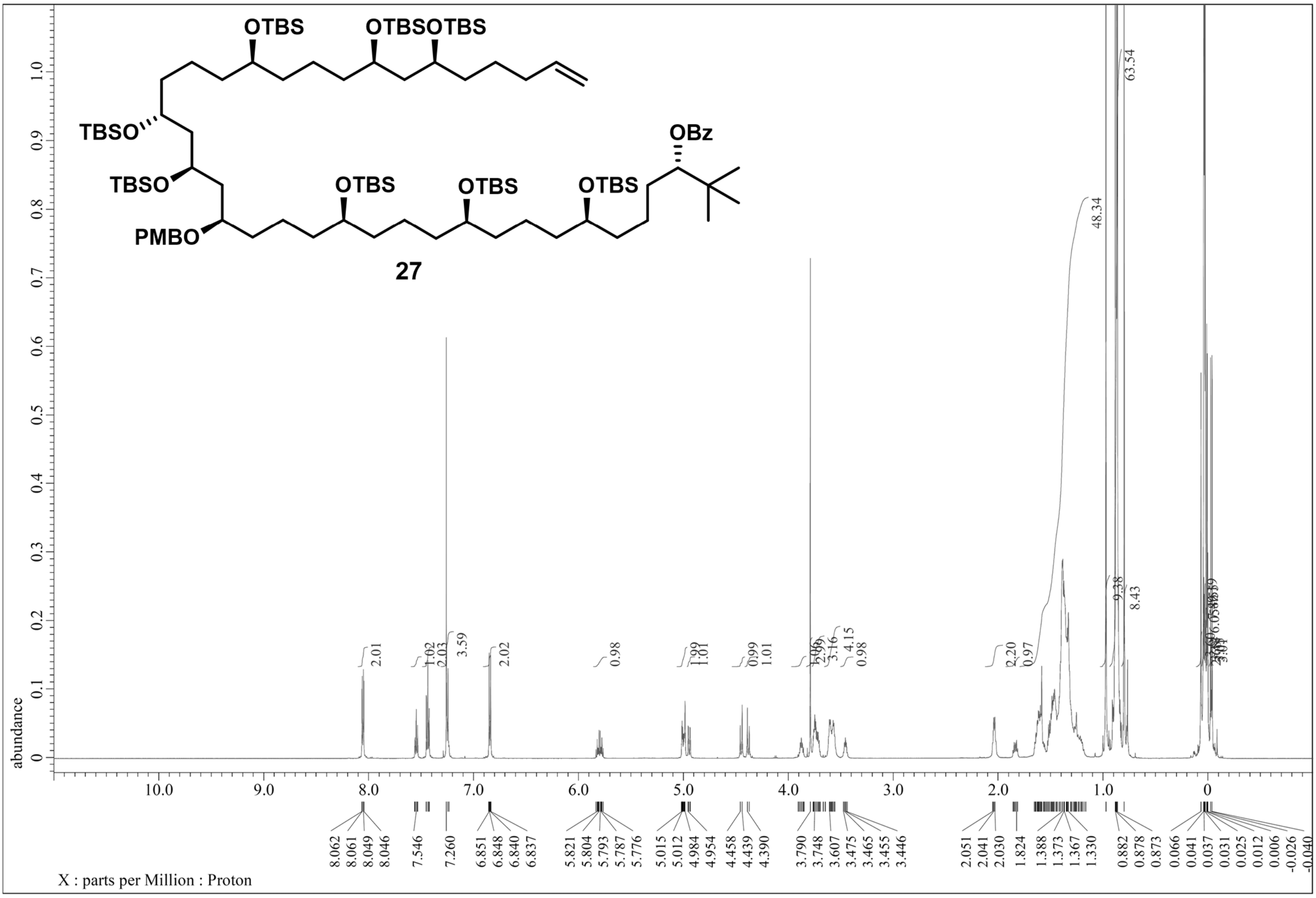

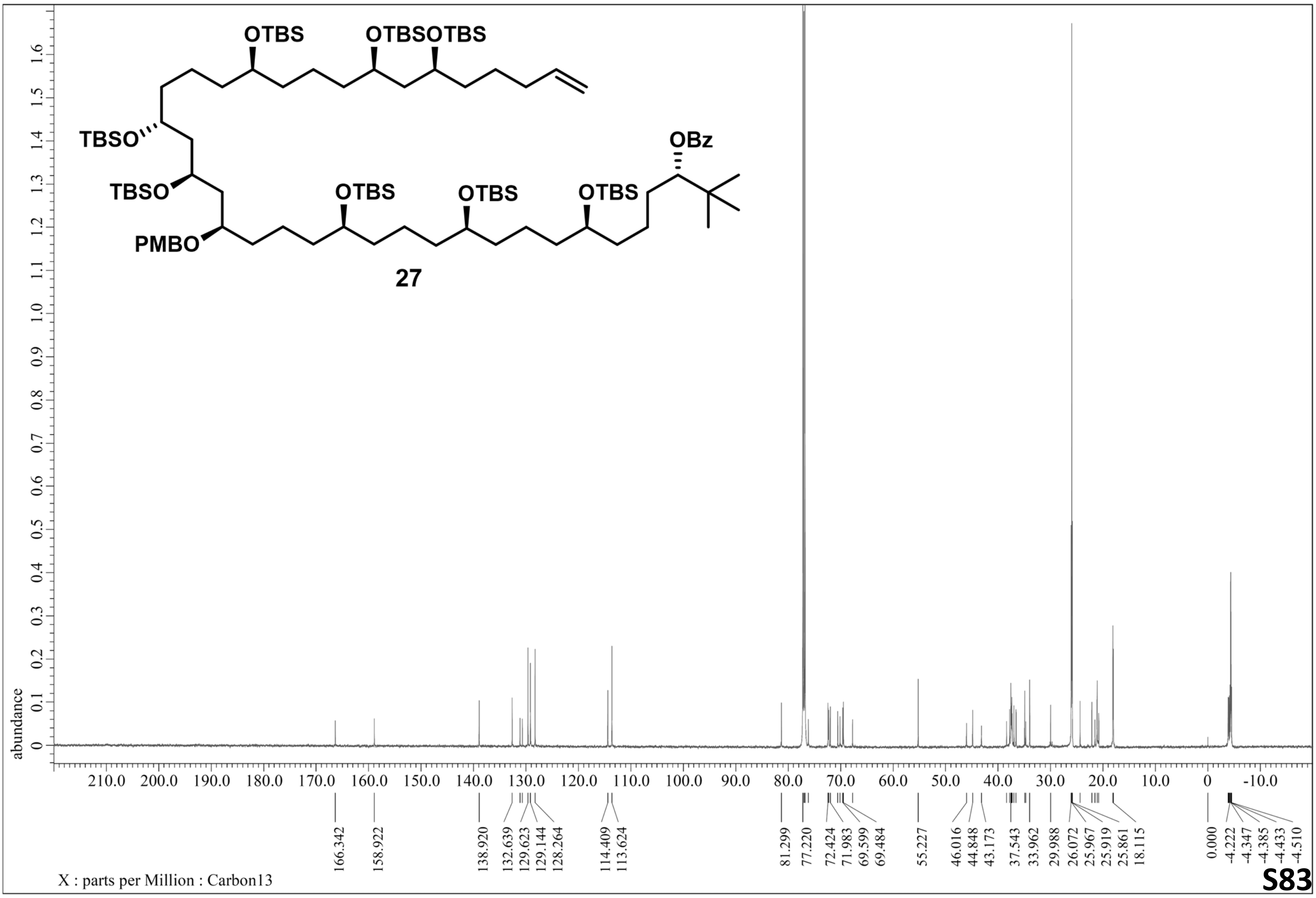

^{1}H NMR (600 MHz, $CDCl_3$) and ^{13}C NMR (151 MHz, $CDCl_3$) spectra of **S18**

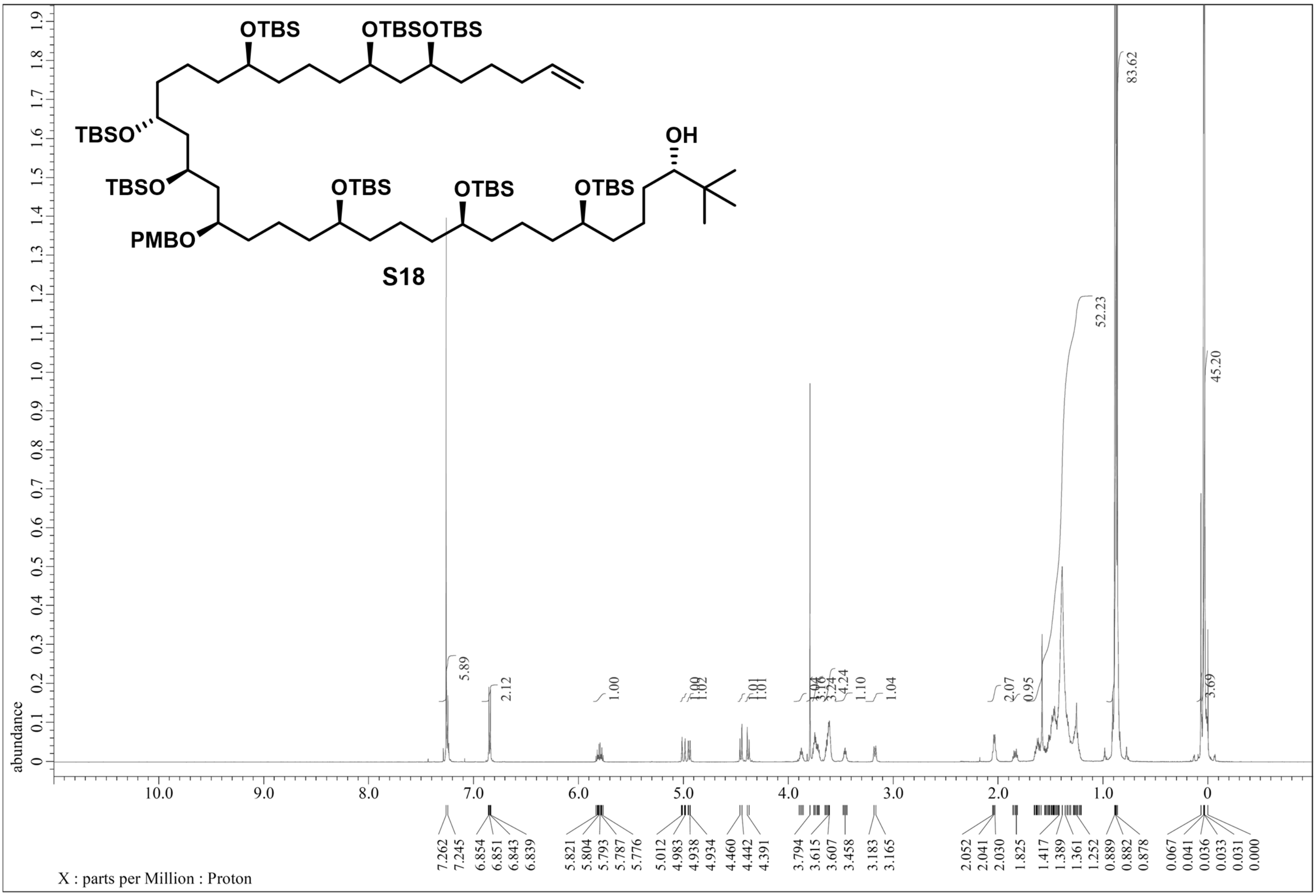

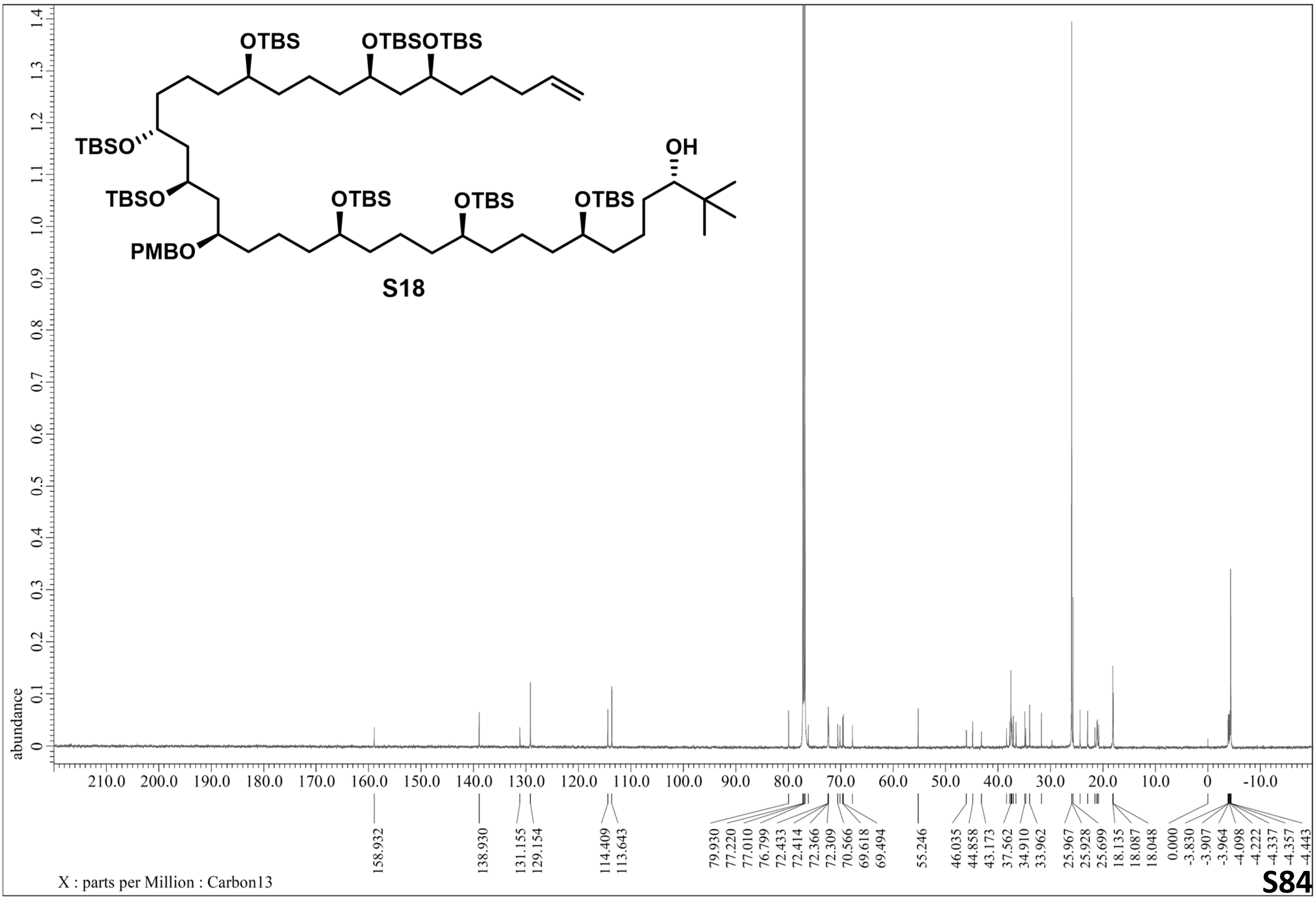

^{1}H NMR (600 MHz, $CDCl_3$) and ^{13}C NMR (151 MHz, $CDCl_3$) spectra of **29**

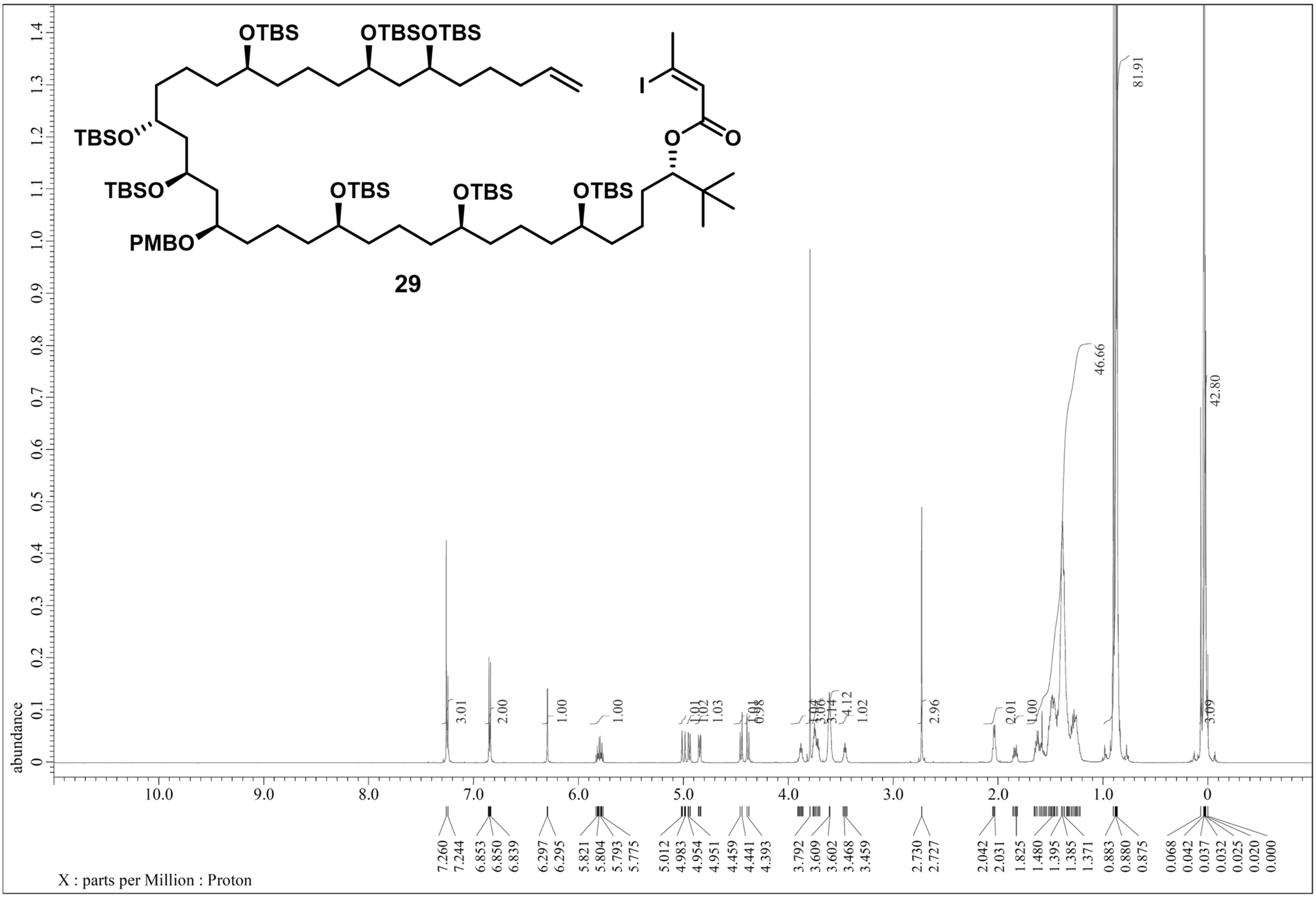

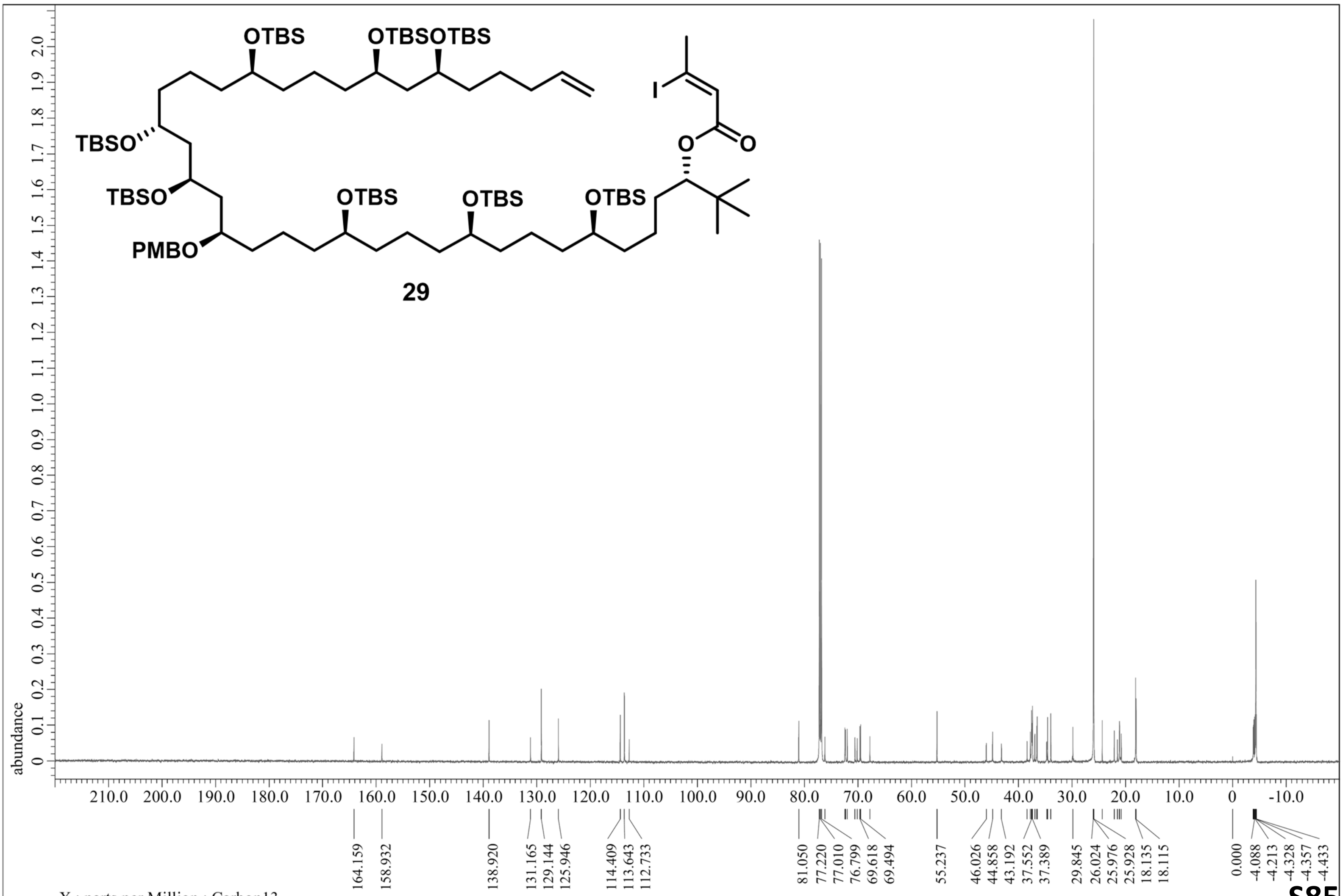

^{1}H NMR (600 MHz, $CDCl_3$) and ^{13}C NMR (151 MHz, $CDCl_3$) spectra of **30**

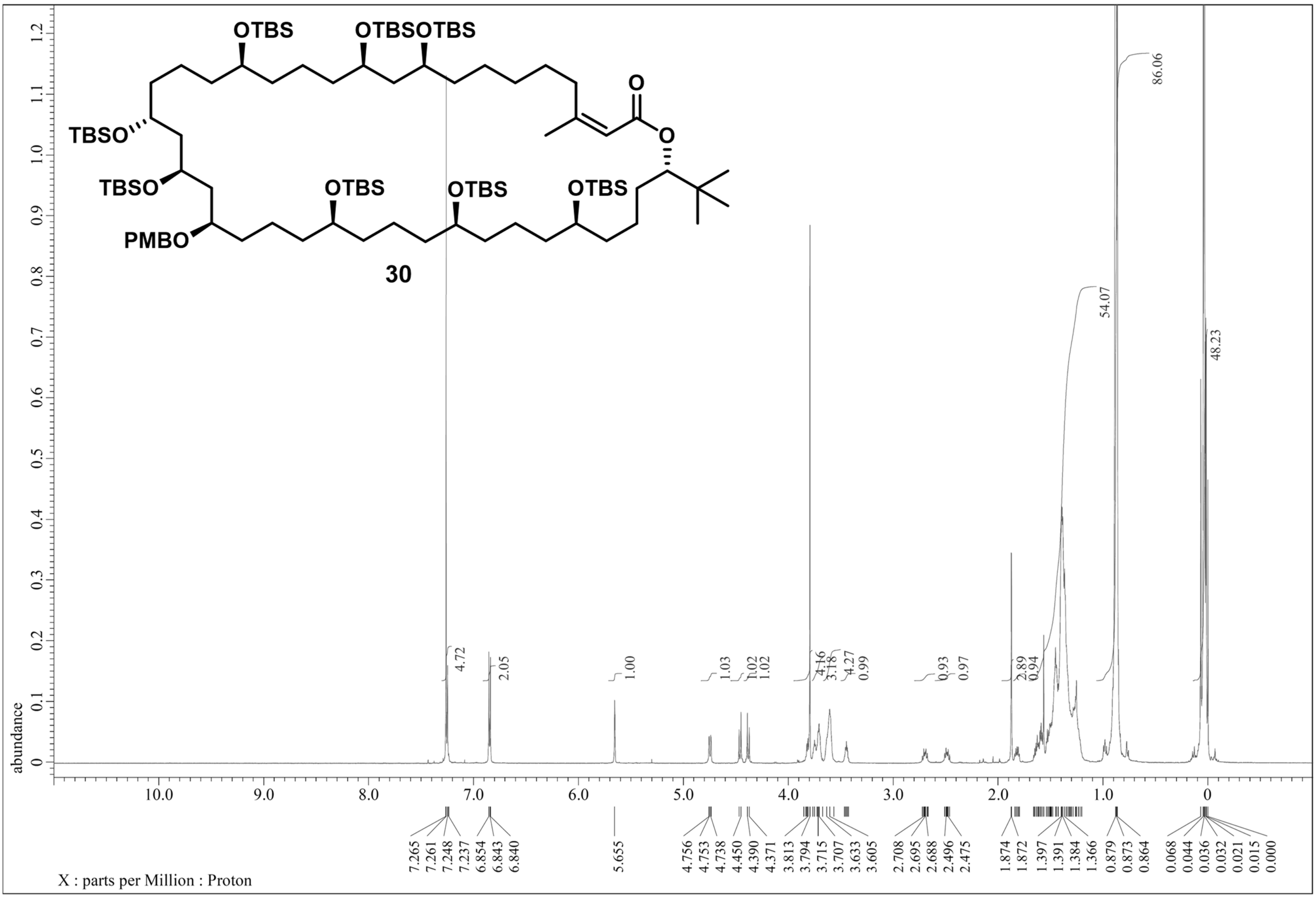

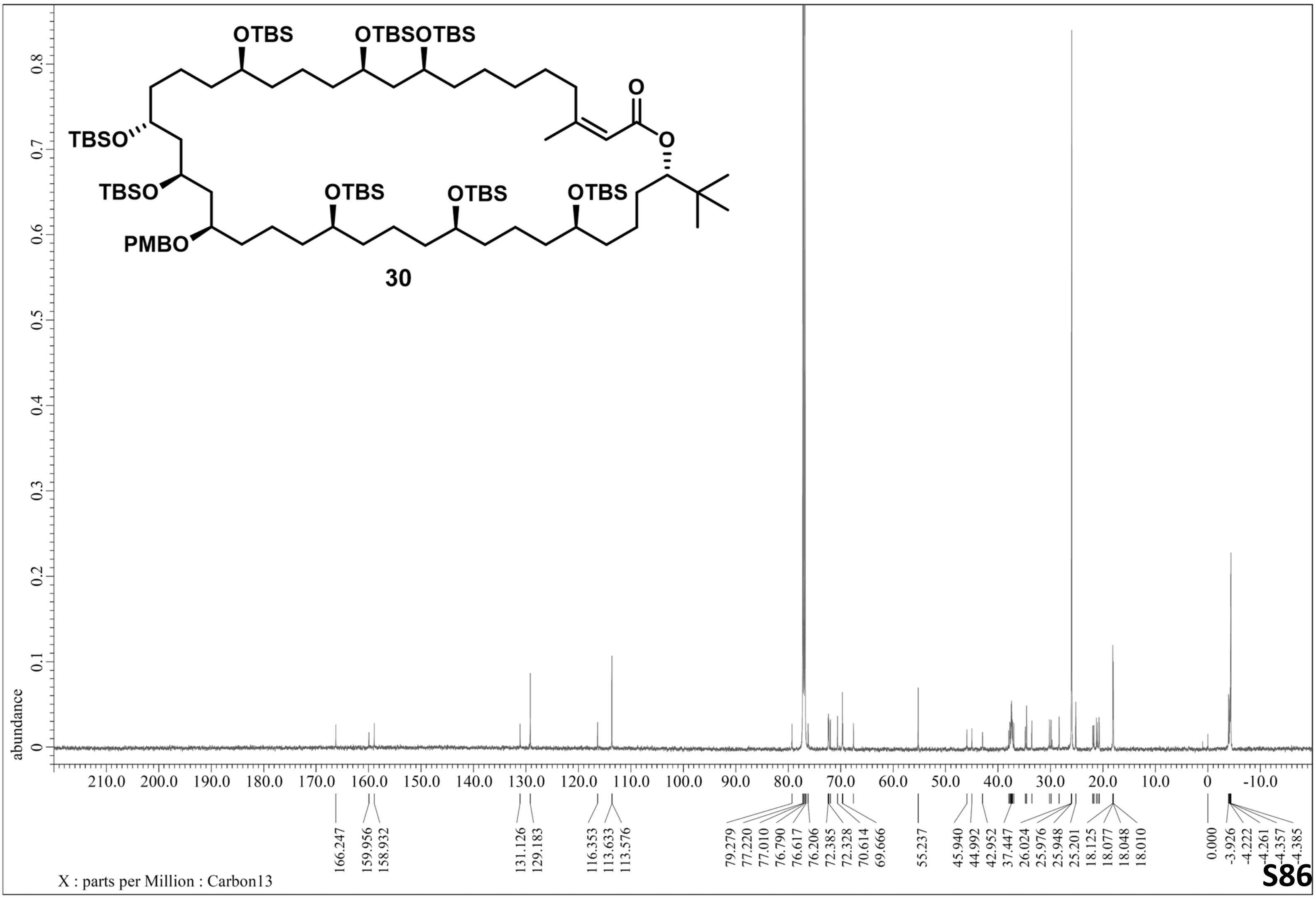

^1H NMR (600 MHz, $CDCl_3$) and ^{13}C NMR (151 MHz, $CDCl_3$) spectra of **S19**

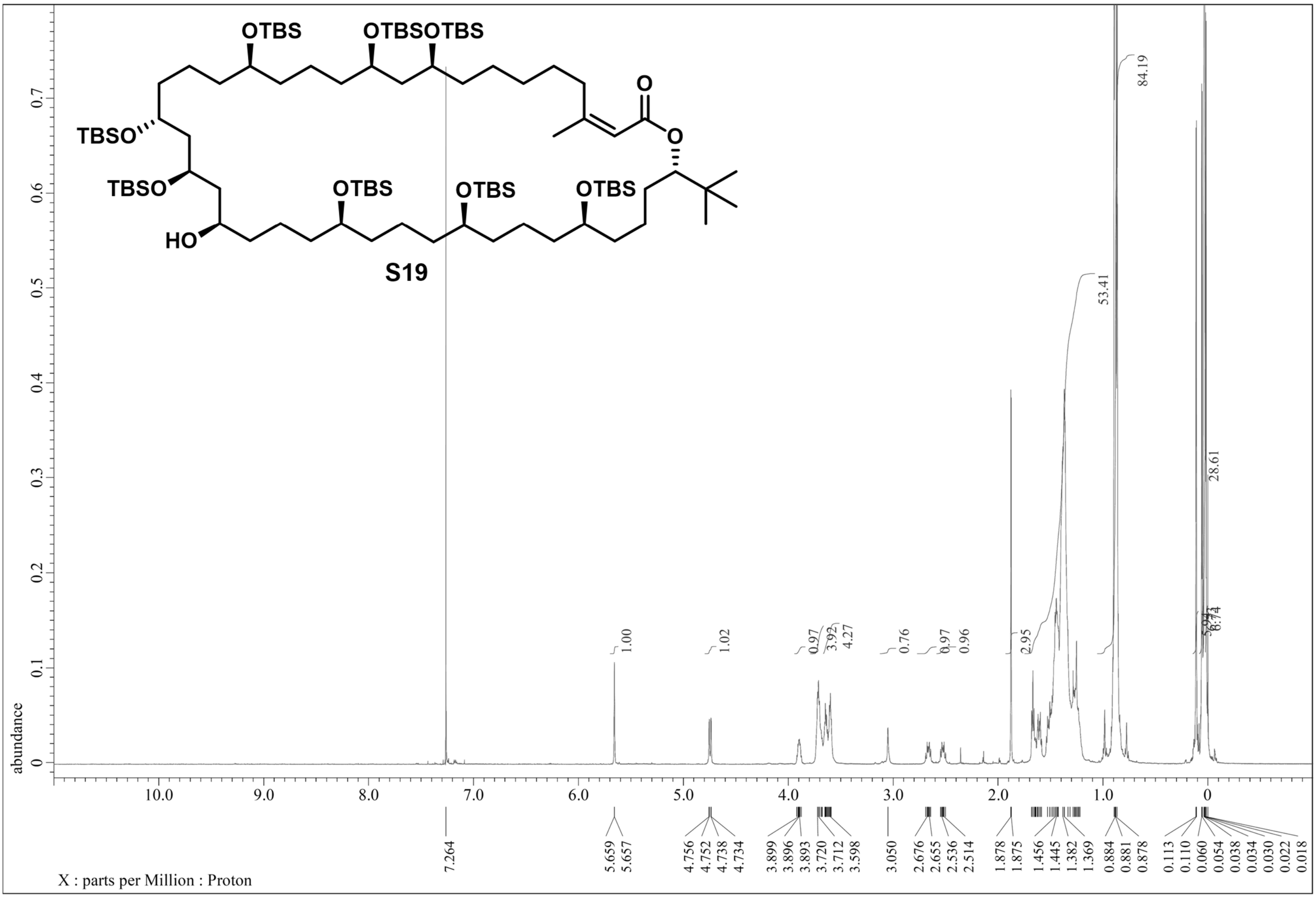

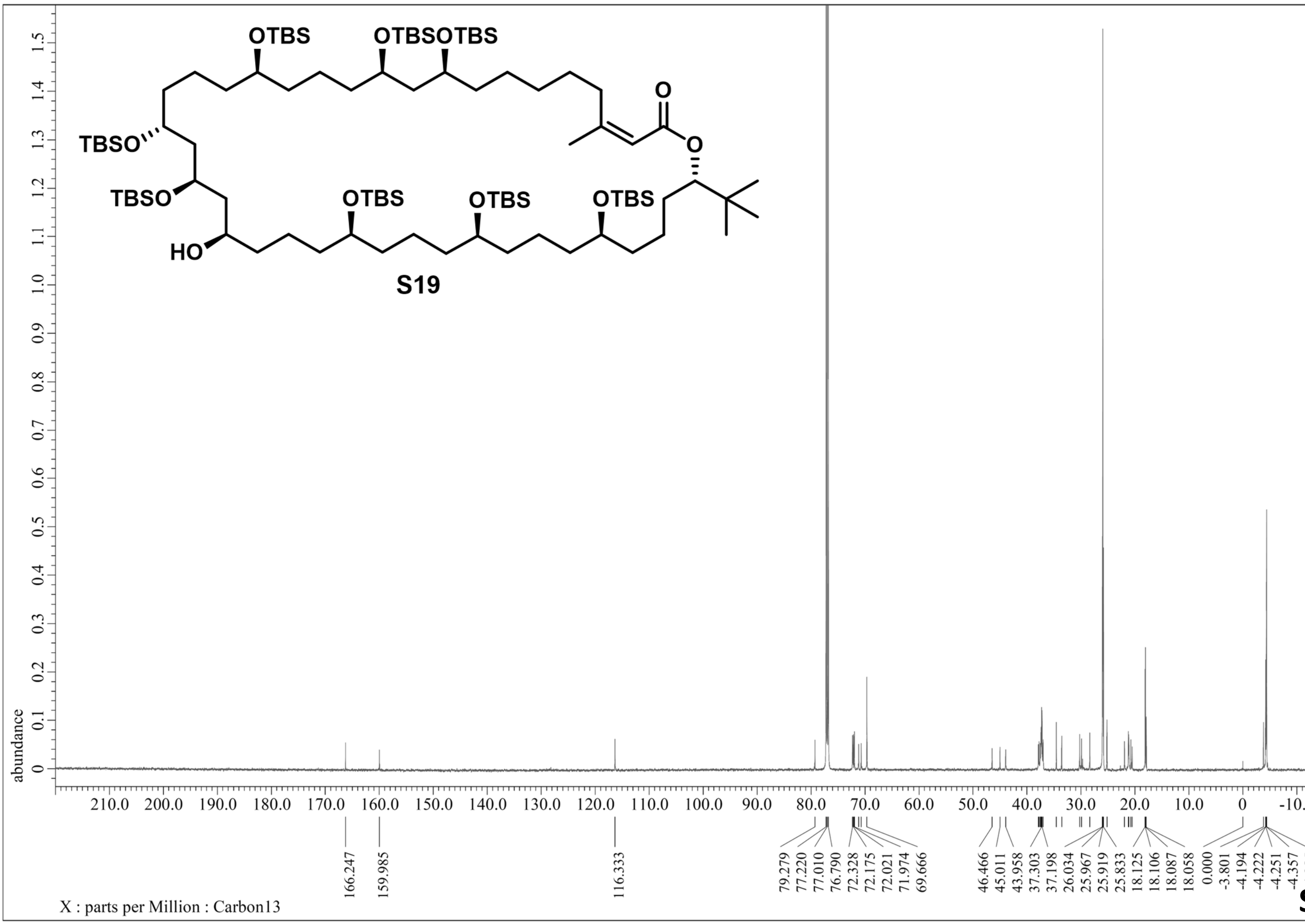

^{1}H NMR (600 MHz, MeOH-d4) and ^{13}C NMR (151 MHz, MeOH-d_4) spectra of **1**

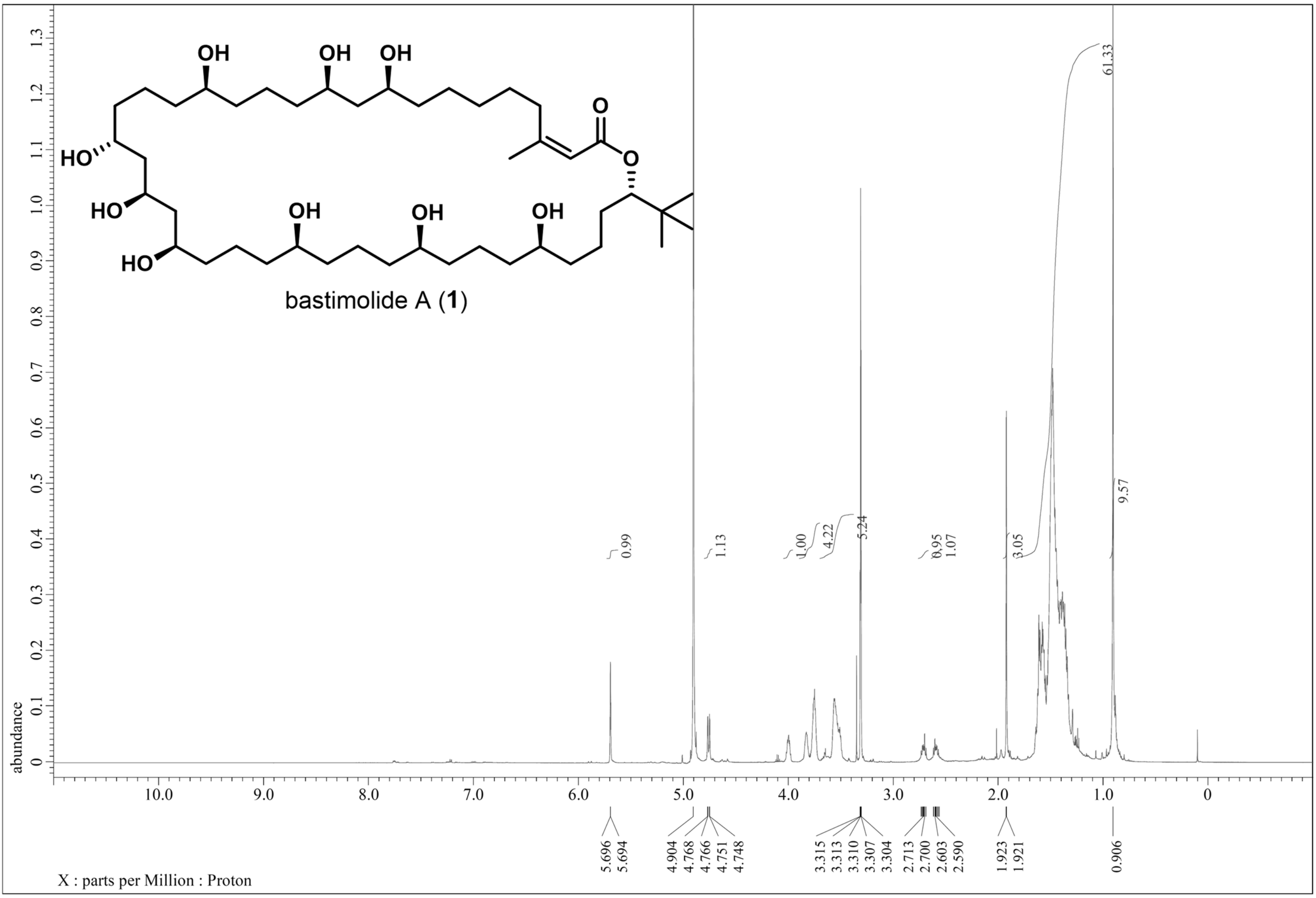

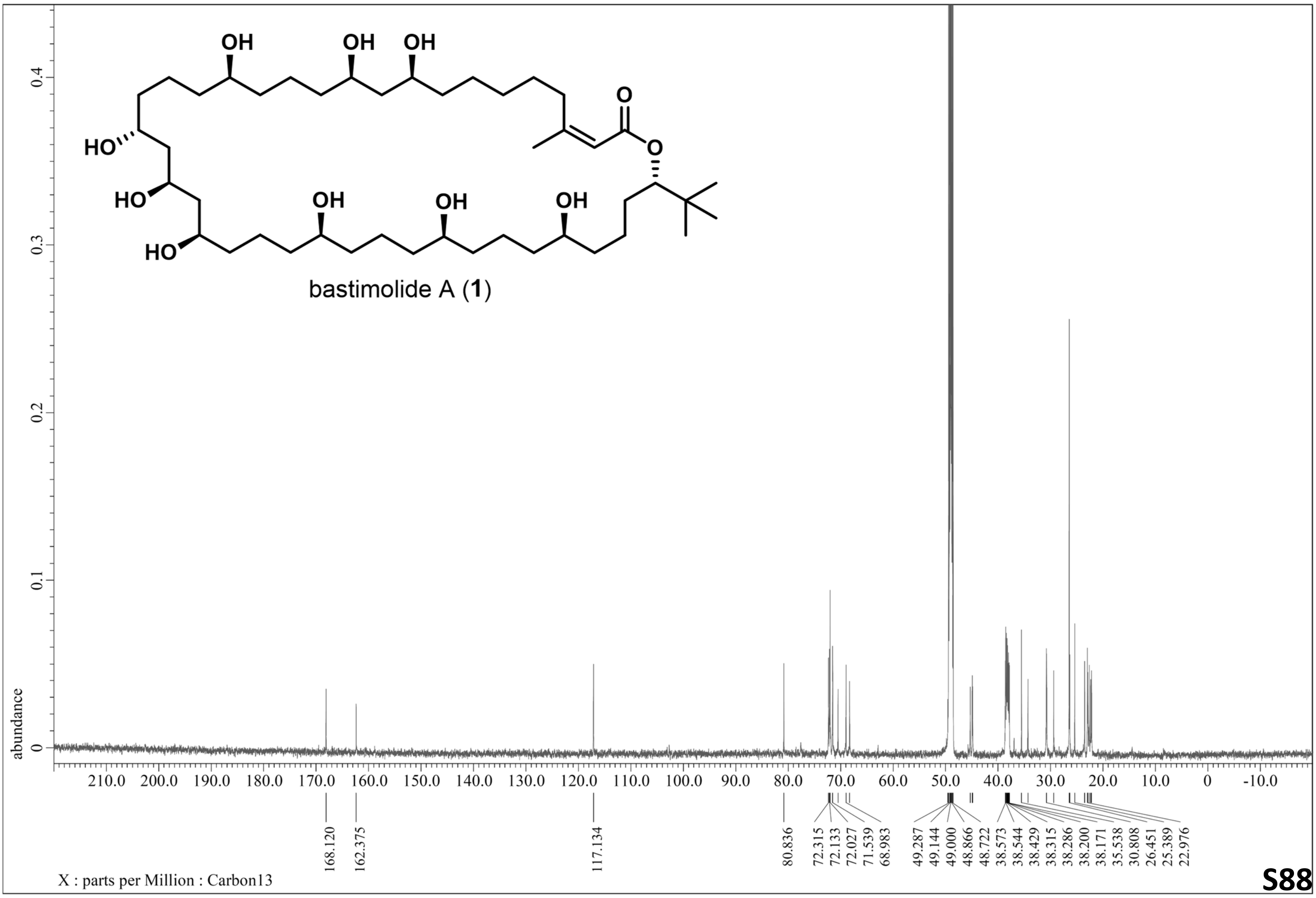

^1H NMR (600 MHz, pyridine-d5) and ^{13}C NMR (151 MHz, pyridine-d_5) spectra of **1**

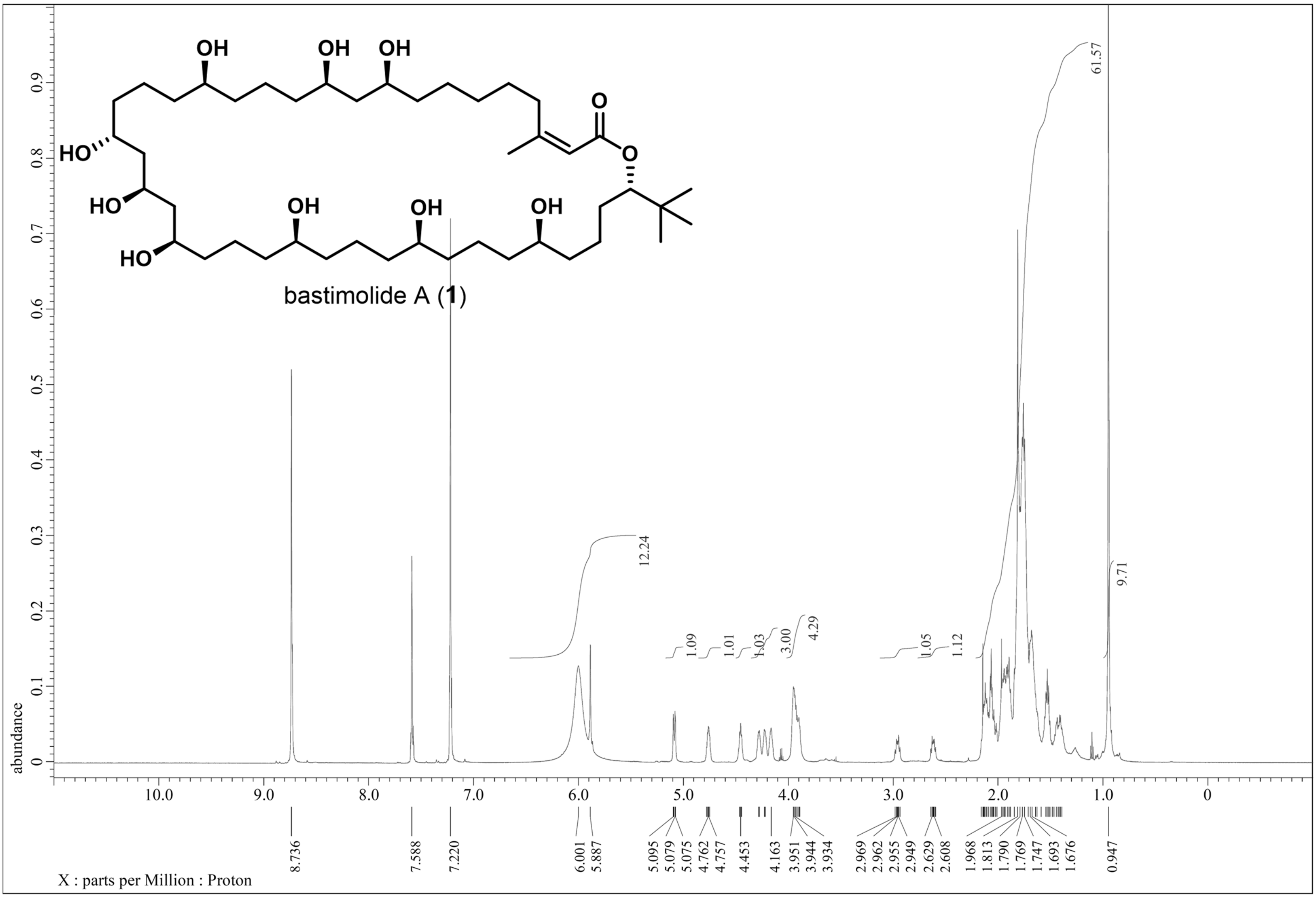

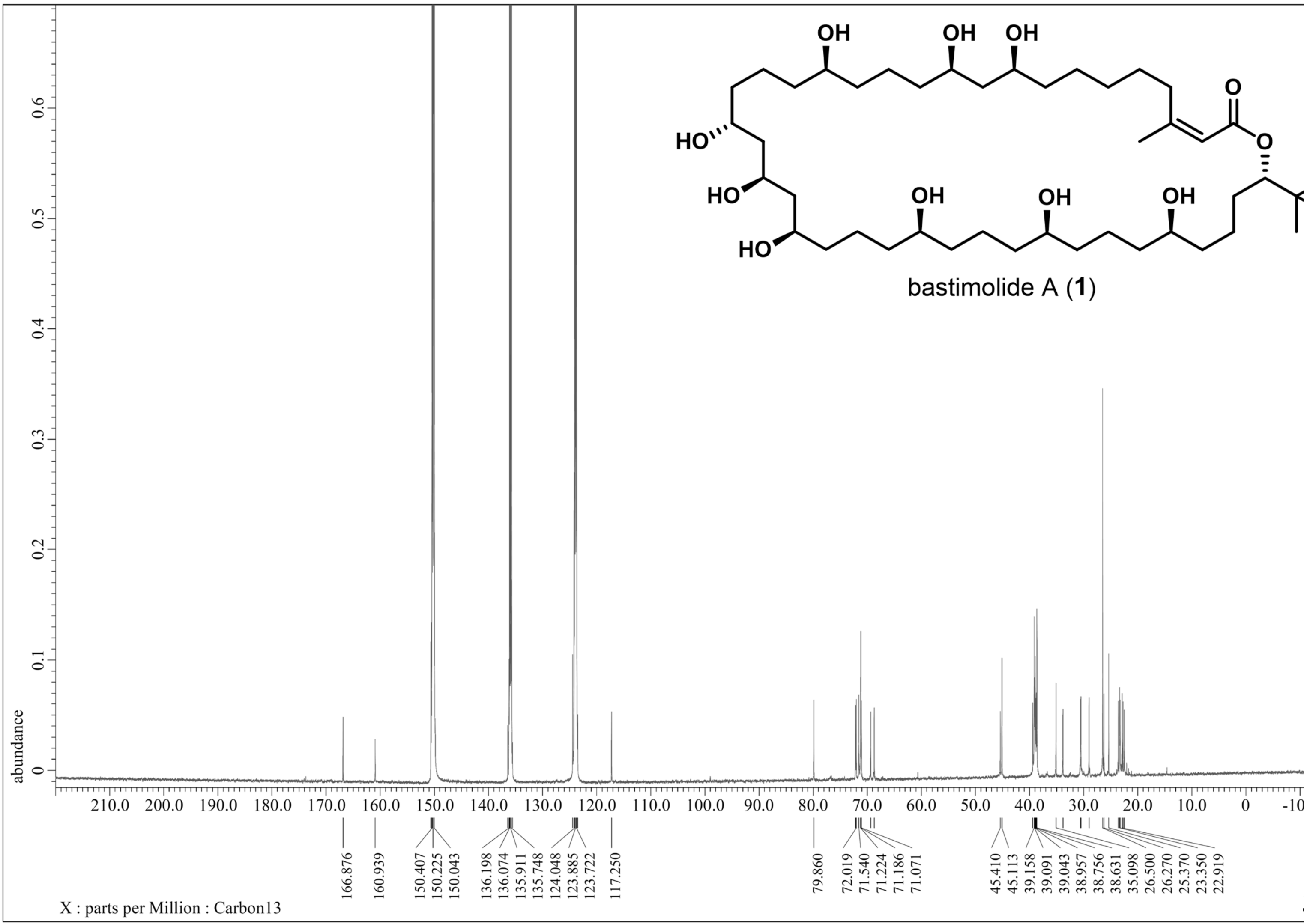

^{1}H NMR (600 MHz, $CDCl_3$) and ^{13}C NMR (151 MHz, $CDCl_3$) spectra of **(*Z*)-3-iodobut-2-enoic acid**

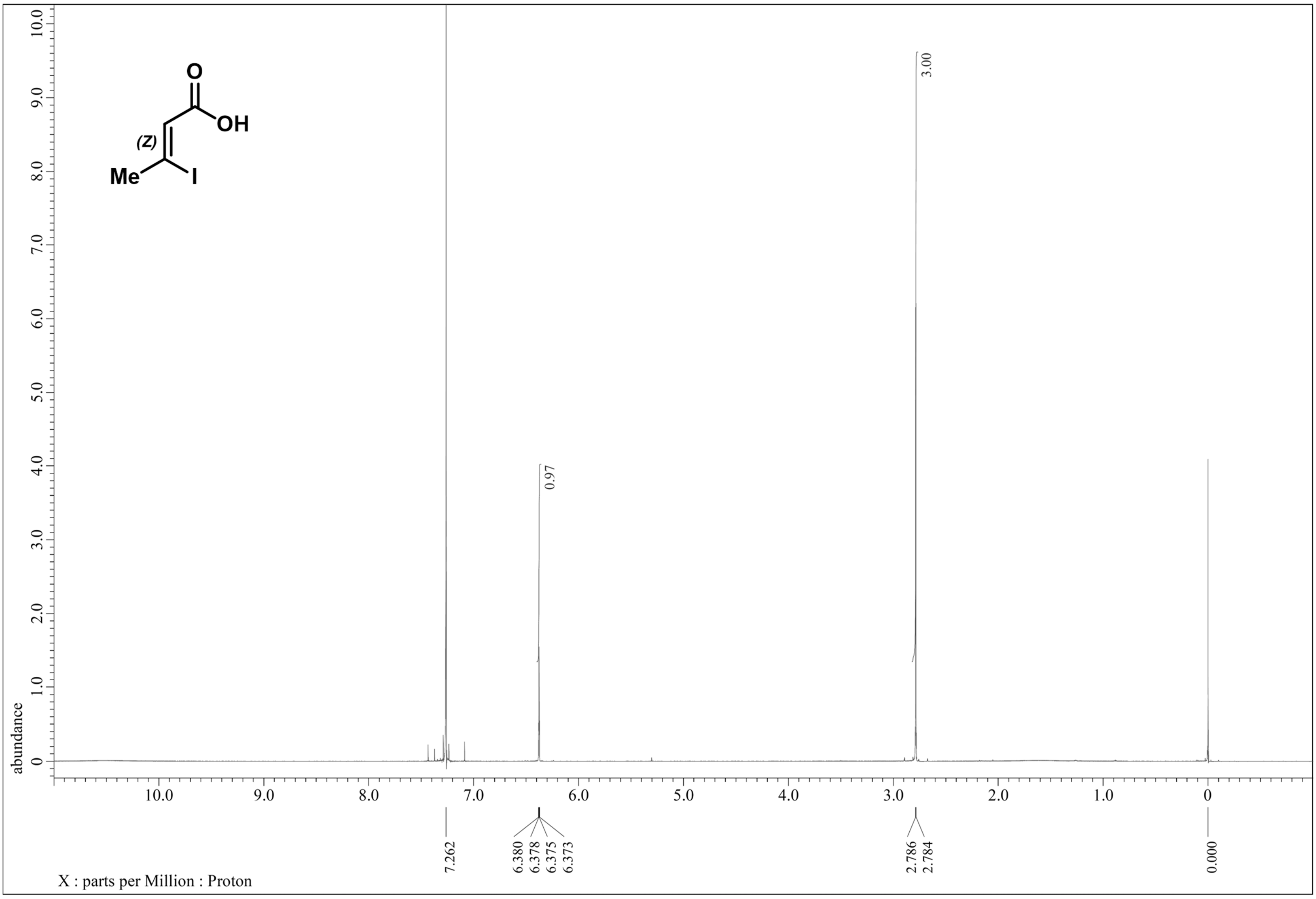

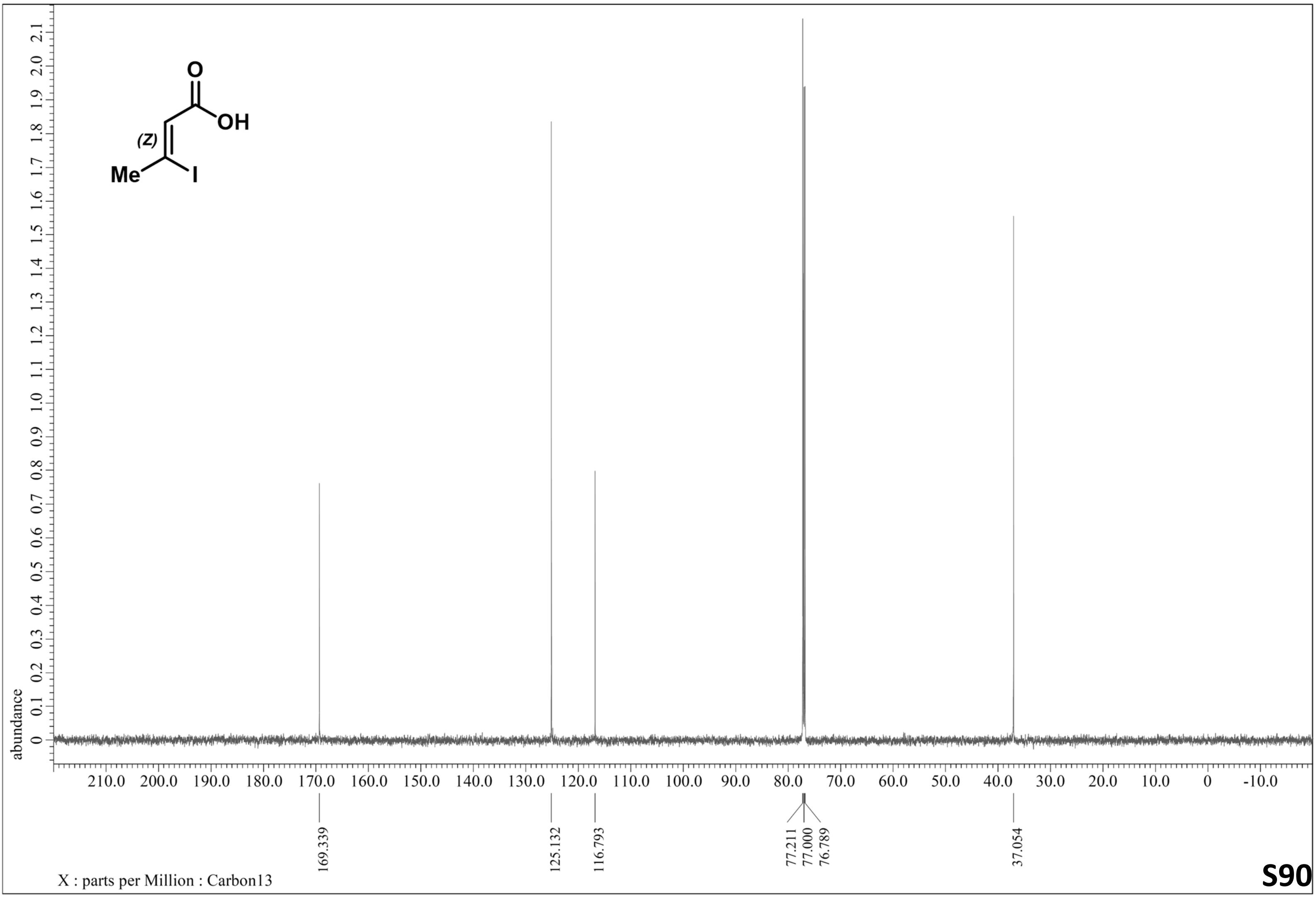

^{1}H NMR (600 MHz, $CDCl_3$) and ^{13}C NMR (151 MHz, $CDCl_3$) spectra of **28**

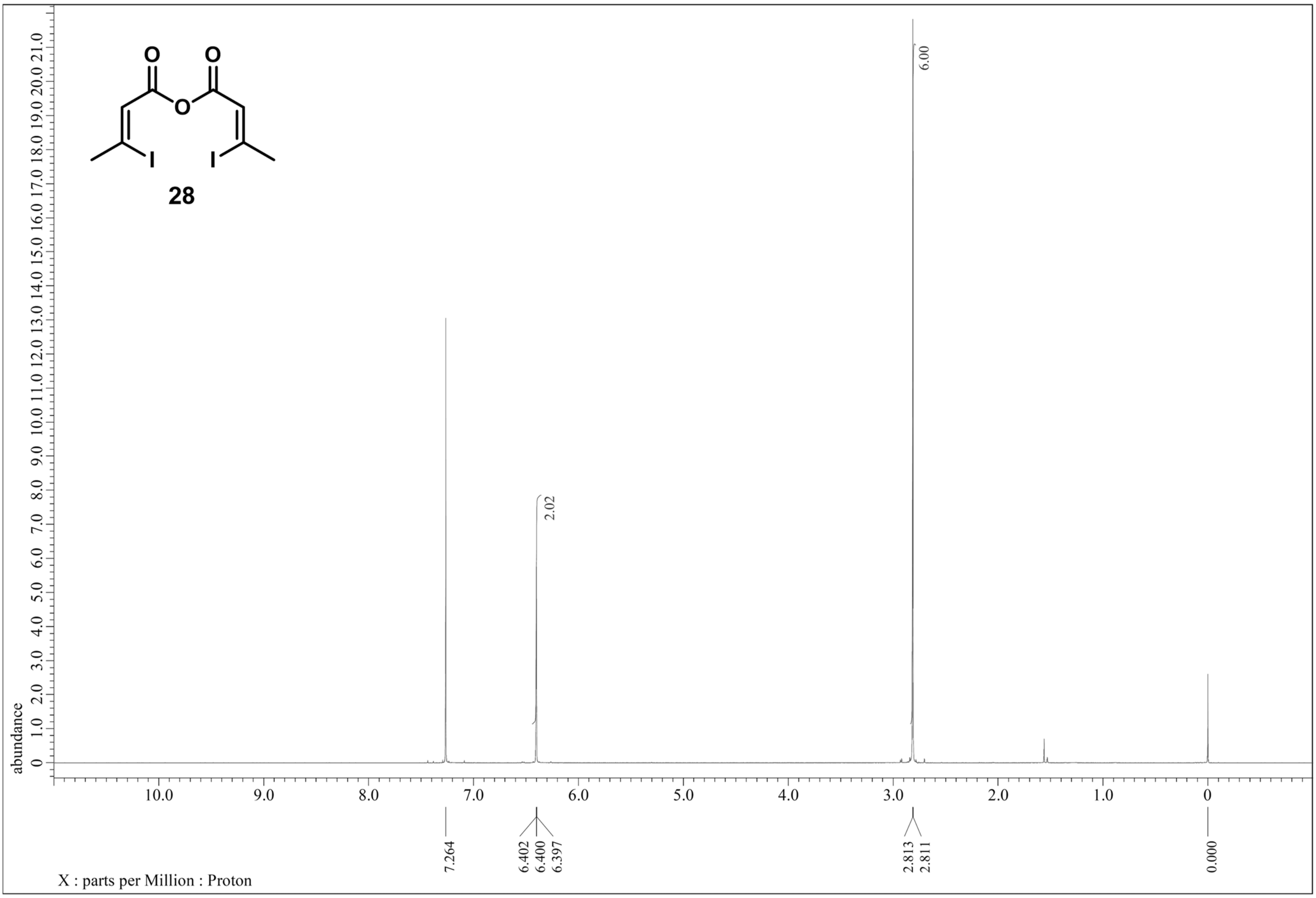

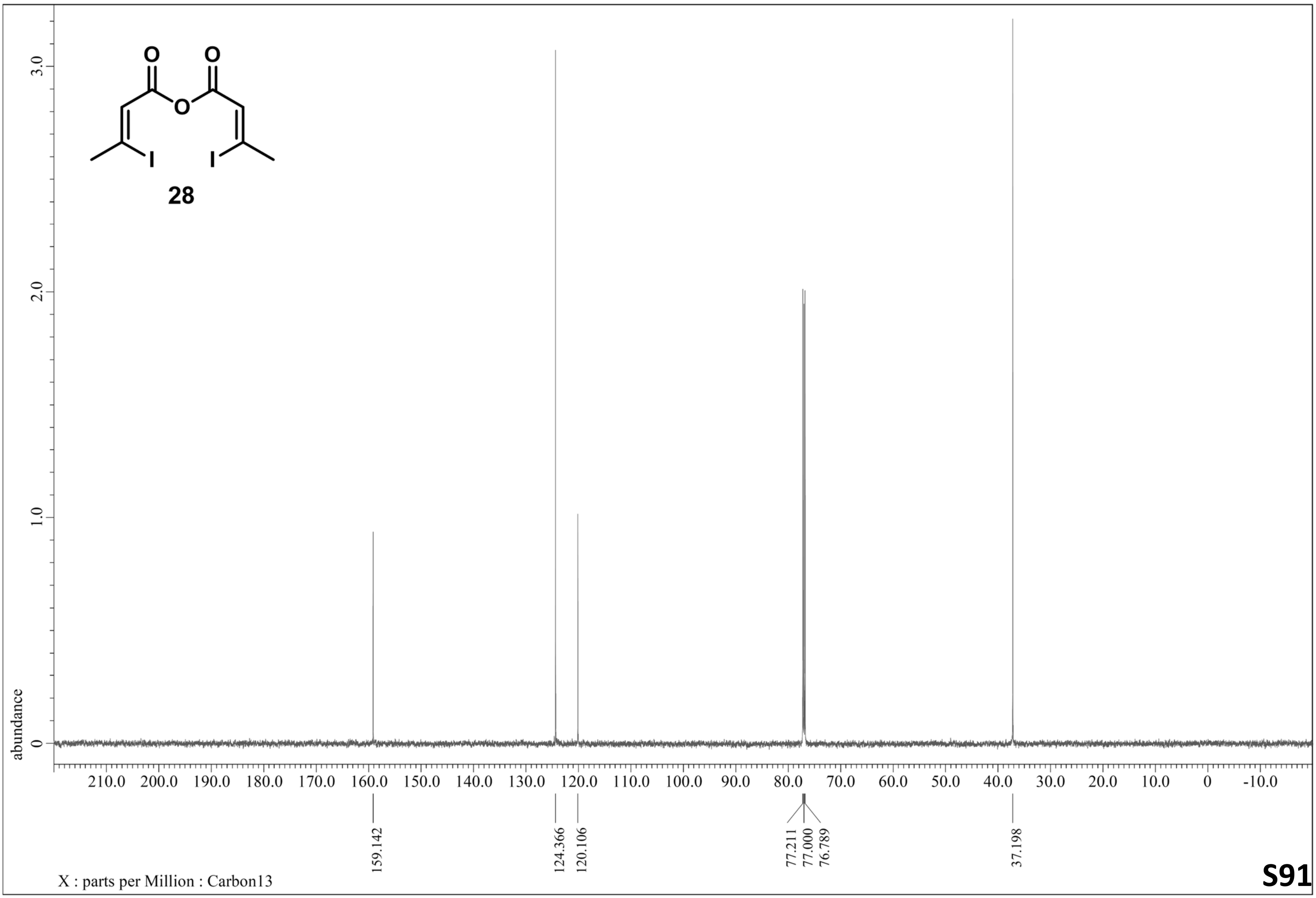

^{1}H NMR (600 MHz, $CDCl_3$) spectra of (*R*)-**S20** and (*S*)-**S21**

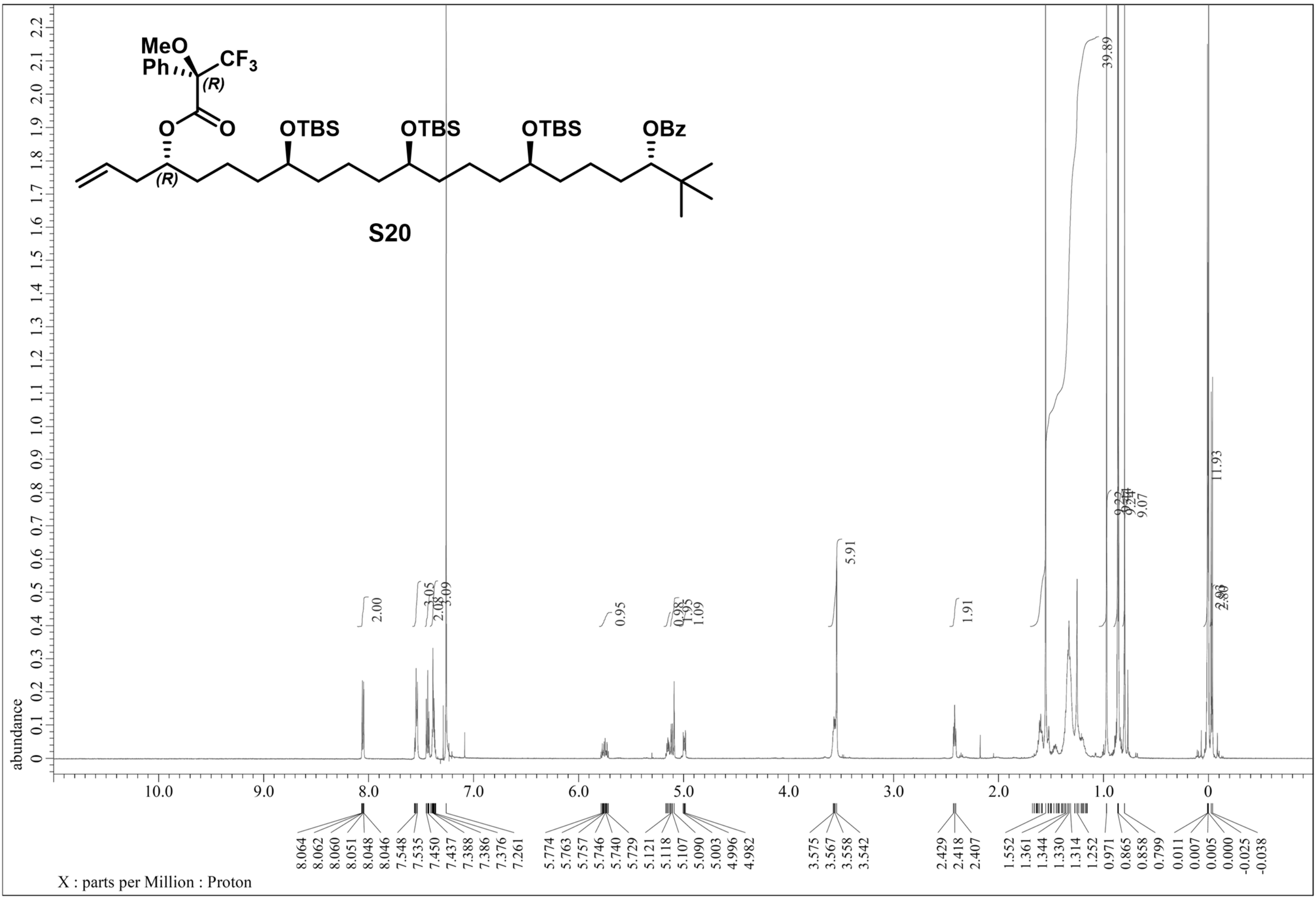

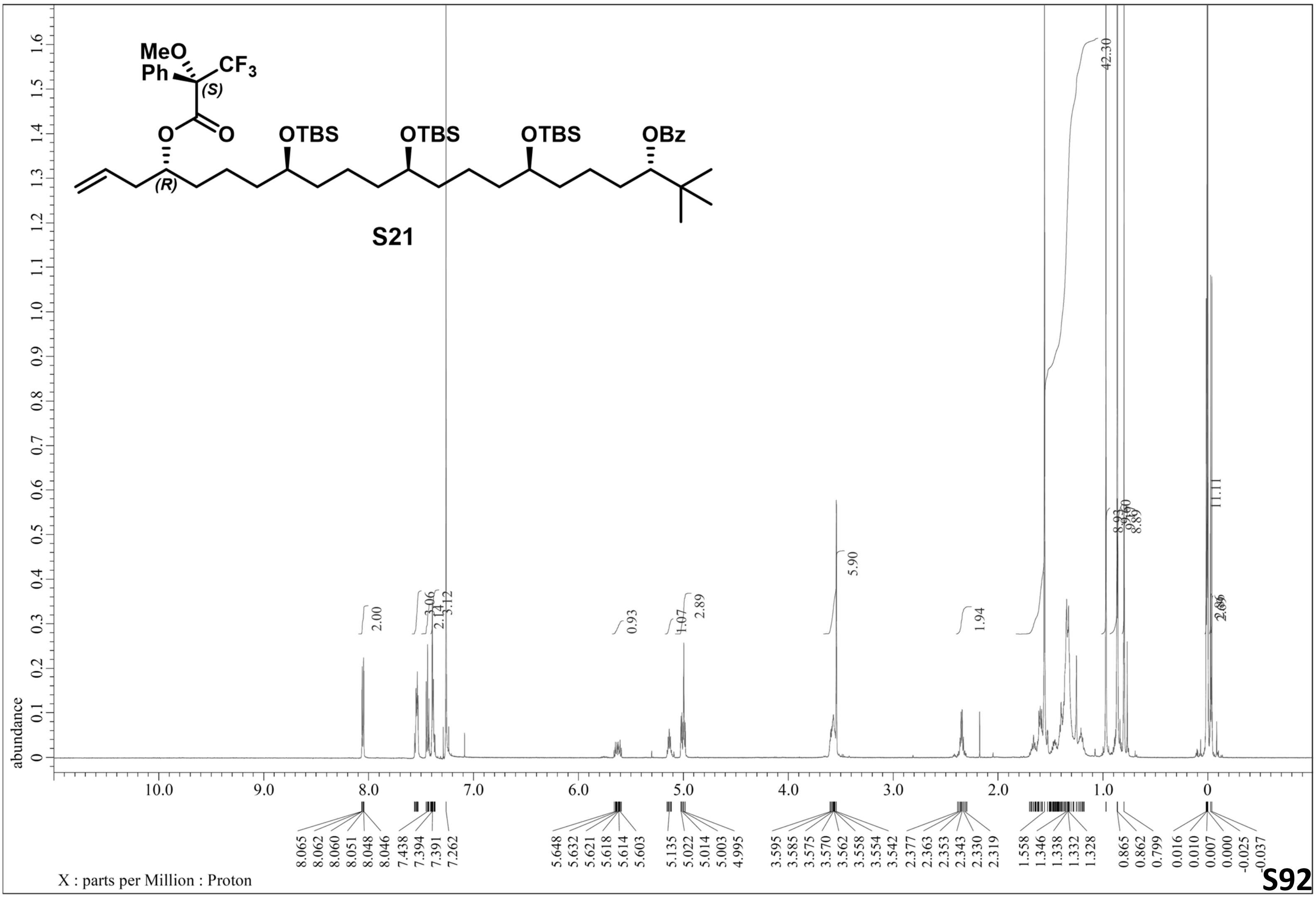

^{1}H NMR (600 MHz, $CDCl_3$) spectra of (*R*)-**S22** and (*S*)-**S23**

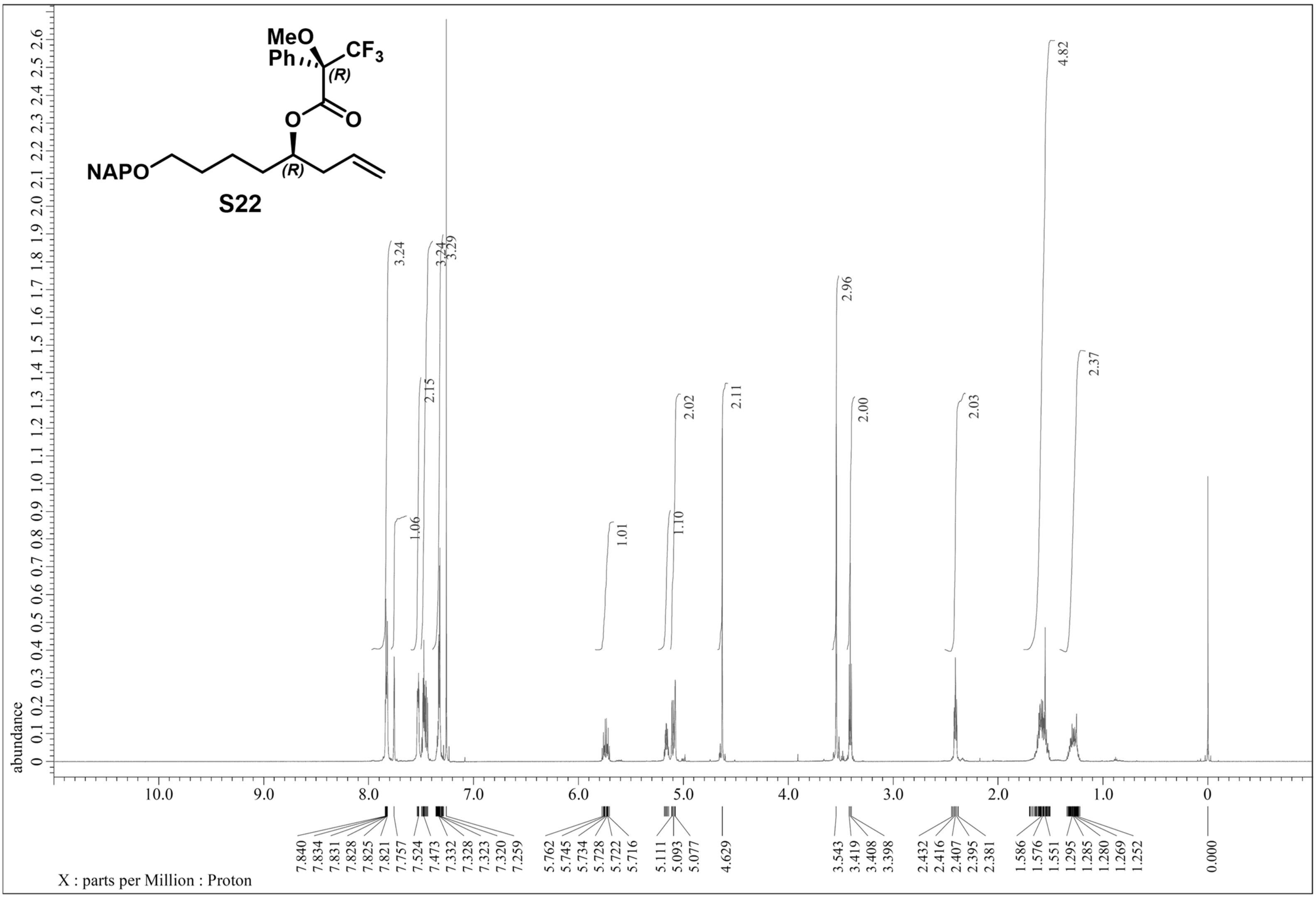

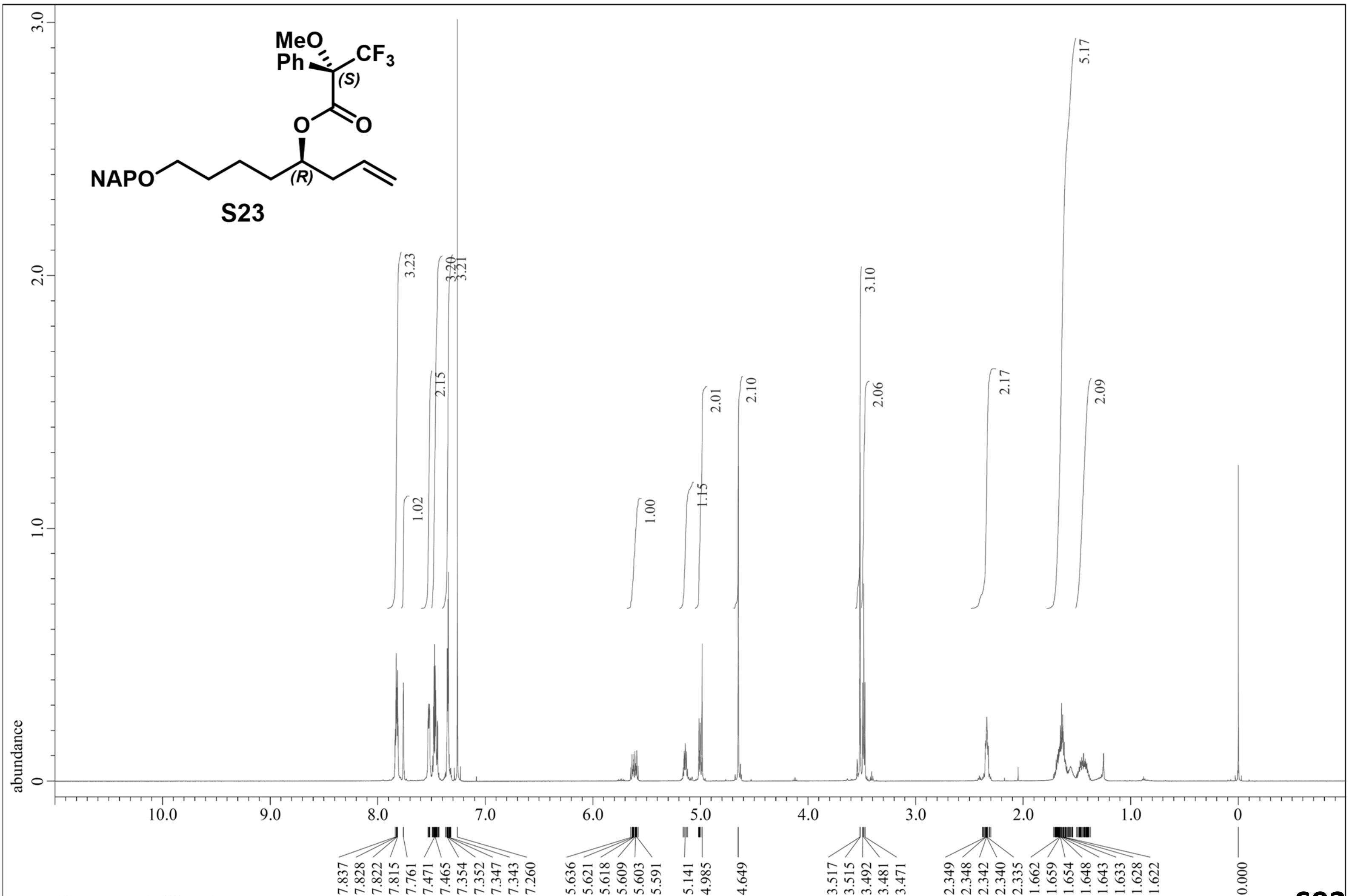

^{1}H NMR (600 MHz, $CDCl_3$) spectra of (*R*)-**S24** and (*S*)-**S25**

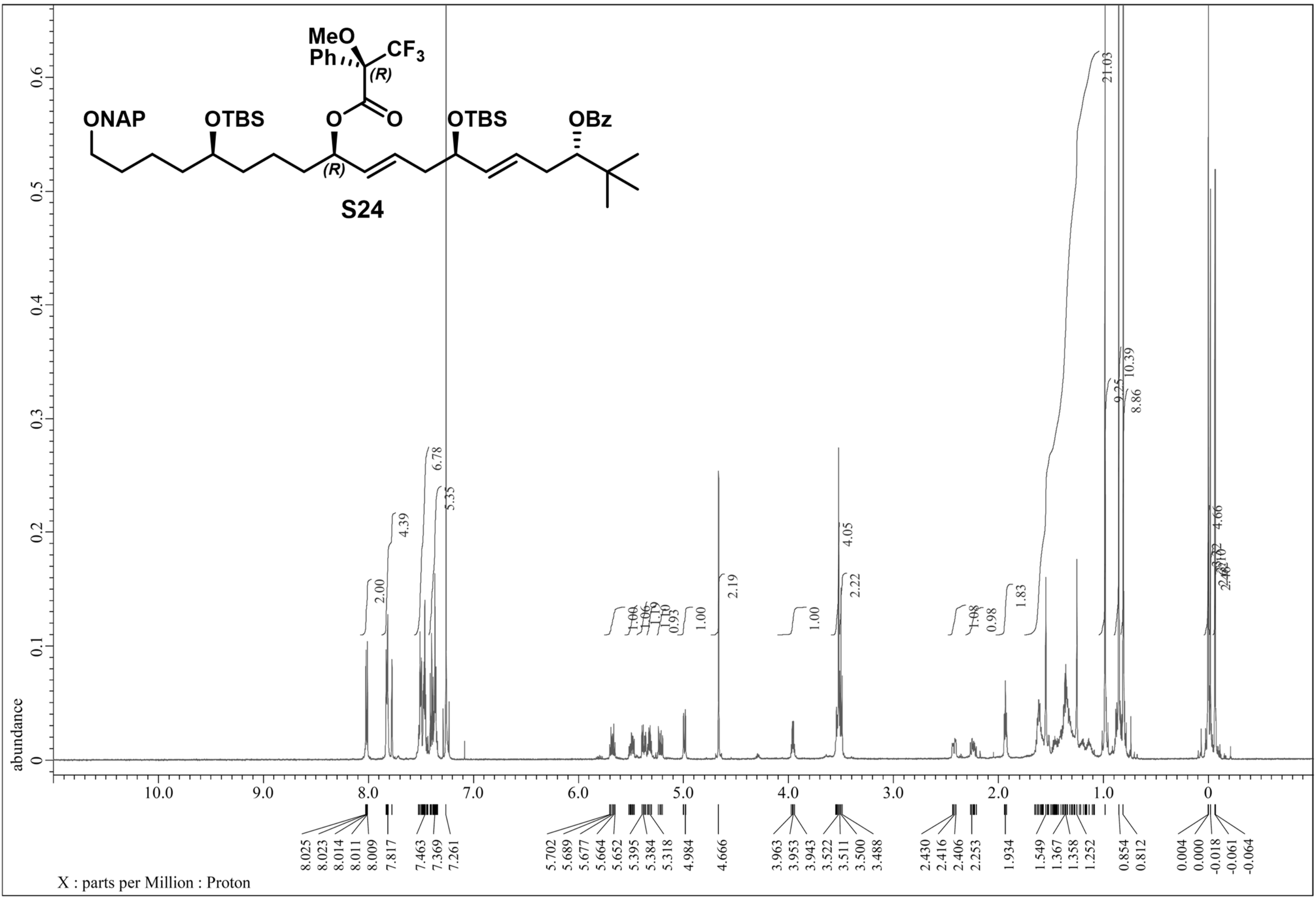

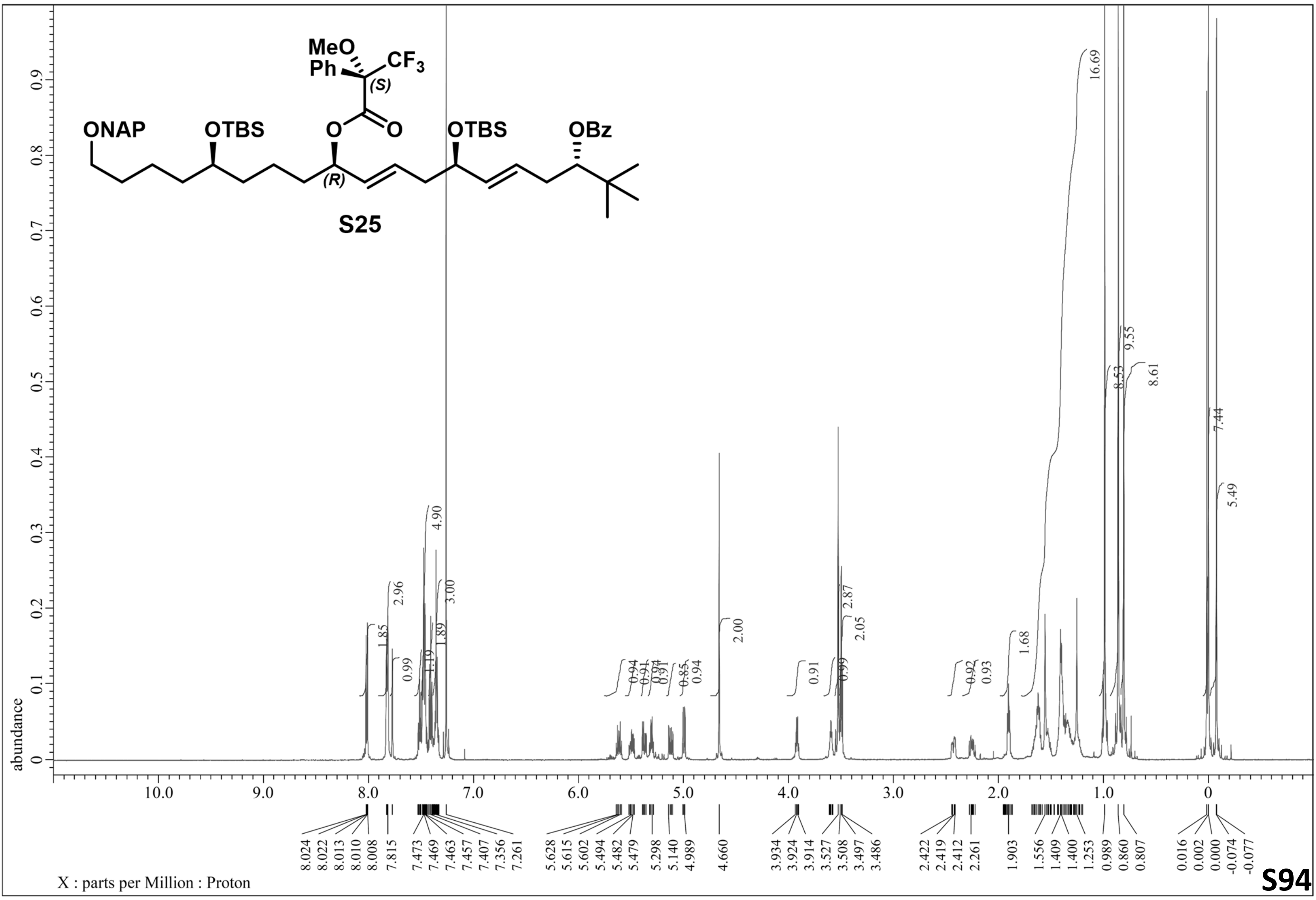

^{1}H NMR (600 MHz, $CDCl_3$) spectra of (*R*)-**S26** and (*S*)-**S27**

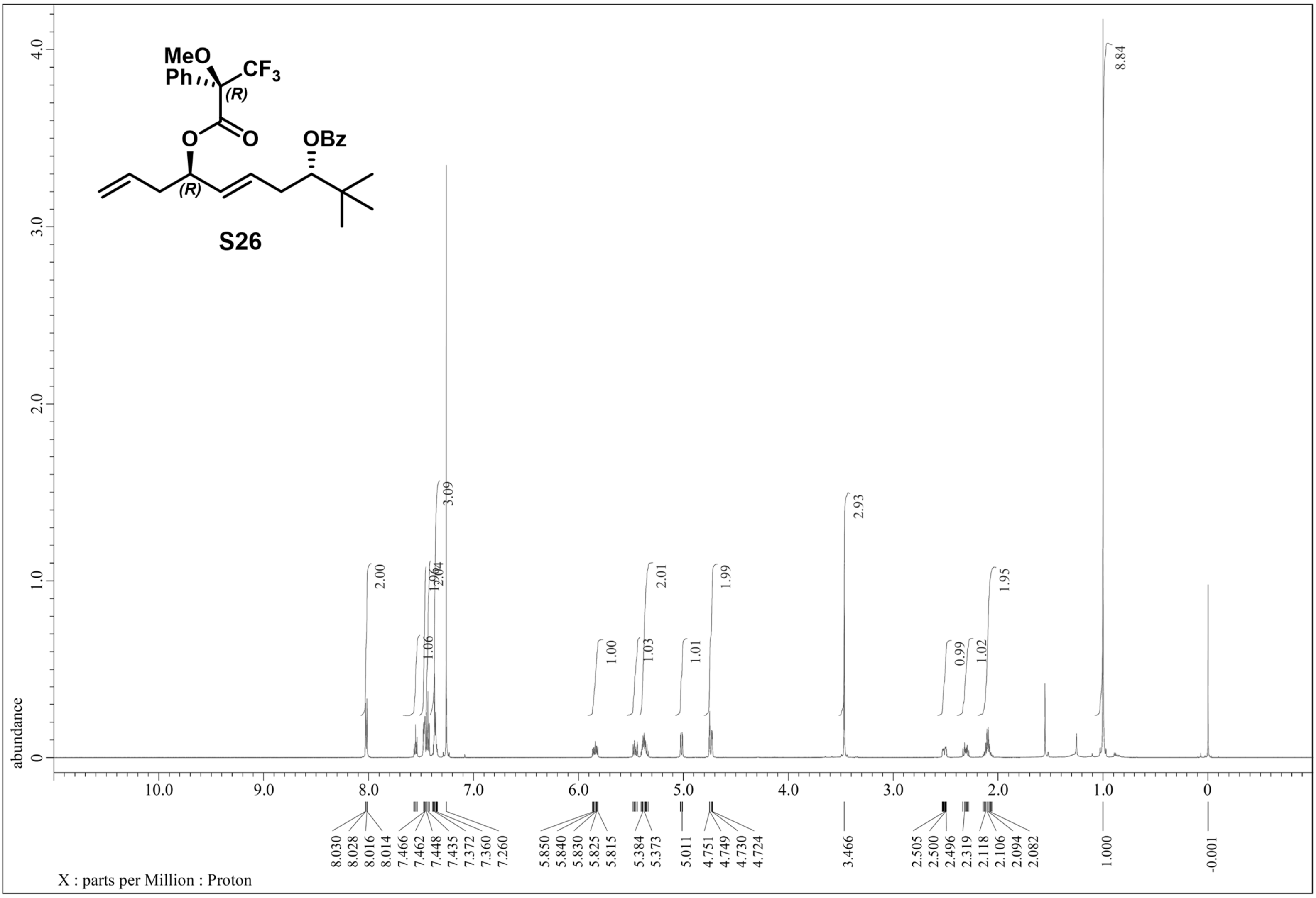

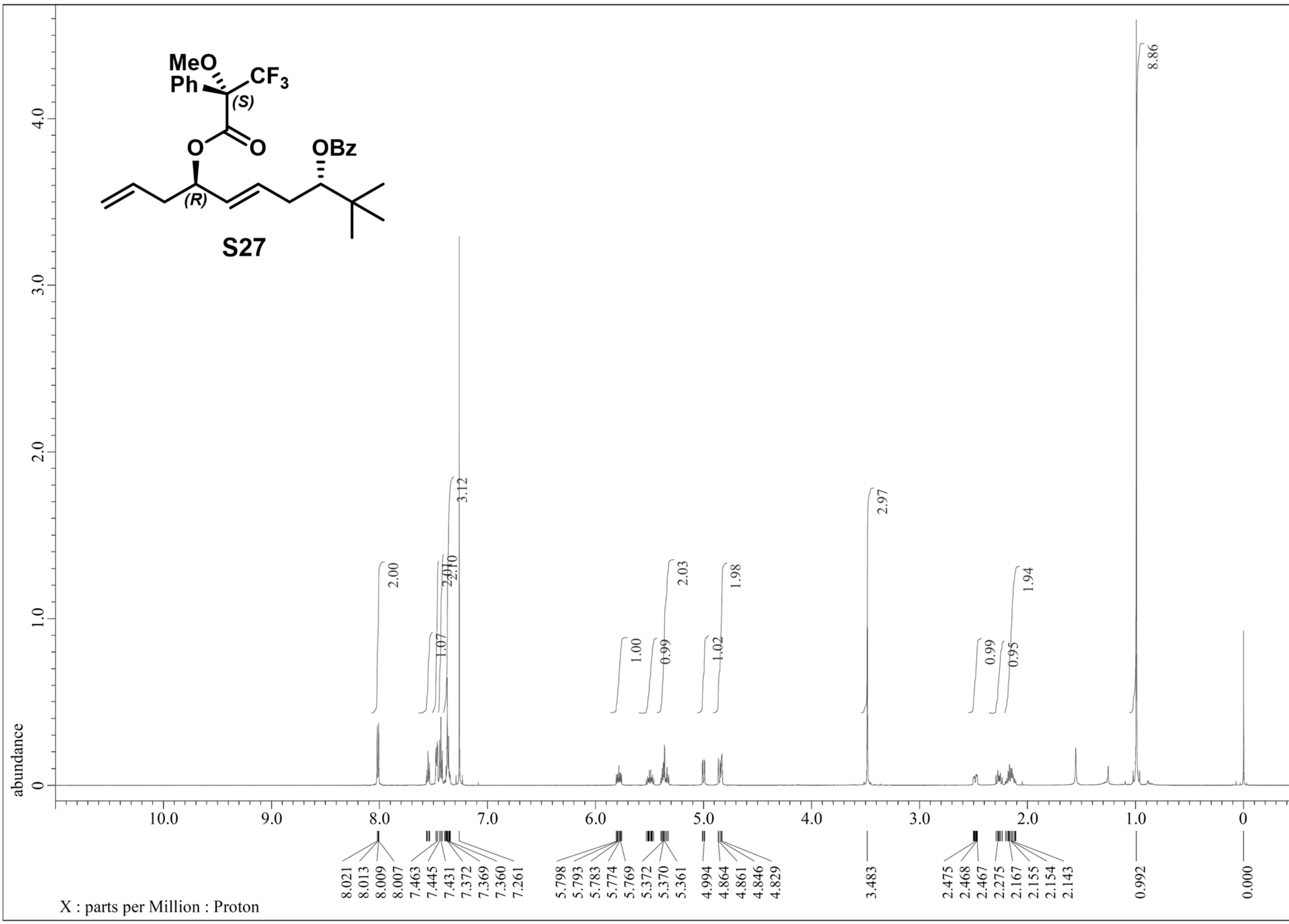

Supplement: Supplementary file 1 [file au5c00630_si_001.pdf]
